# Heterotrimetallic Assemblies with 1,2,4,5-Tetrakis(diphenylphosphino)benzene Bridges: Constructs for Controlling the Separation and Spatial Orientation of Redox-Active Metallodithiolene Groups

**DOI:** 10.1021/acs.inorgchem.2c03112

**Published:** 2022-10-25

**Authors:** Satyendra Kumar, Malathy Selvachandran, Che Wu, Robert A. Pascal, Xiaodong Zhang, Tod Grusenmeyer, Russell H. Schmehl, Stephen Sproules, Joel T. Mague, James P. Donahue

**Affiliations:** † Department of Chemistry, Tulane University, 6400 Freret Street, New Orleans, Louisiana 70118, United States; ‡ WestCHEM, School of Chemistry, University of Glasgow, Glasgow G12 8QQ, United Kingdom

## Abstract

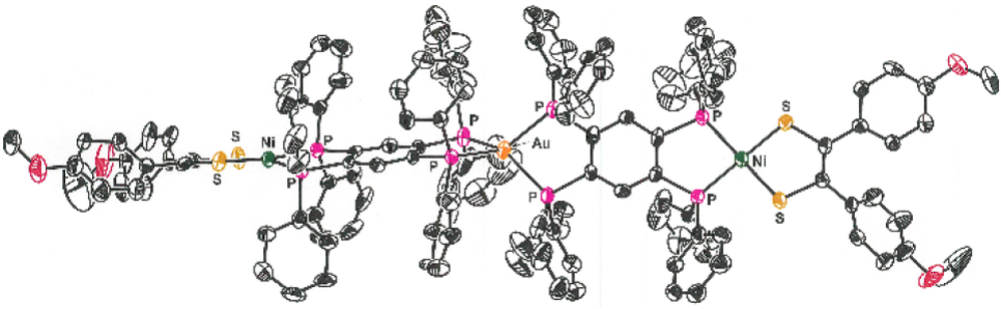

Metallodithiolene complexes of the type [(R_2_C_2_S_2_)M(η^2^-tpbz)] [R = CN, Ph, or *p*-anisyl; M = Ni^2+^, Pd^2+^, or Pt^2+^; tpbz = 1,2,4,5-tetrakis(diphenylphosphino)benzene] chelate transition metals ions to form trimetallic arrays [[(R_2_C_2_S_2_)M(tpbz)]_2_M′]^*n*+^, where M′ is square planar Pt^2+^, tetrahedral Cu^+^, Ag^+^, or Au^+^, or octahedral {ReBr(CO)}/{Re(CO)_2_}^+^. Forcing conditions (190 °C reflux in decalin, 72 h) are demanded for the Re^+^ compounds. With third-row metals at the nexus, the compounds are stable to air. Twelve members of the set have been characterized by X-ray diffraction and reveal dithiolene centroid–centroid distances ranging from 22.4 to 24.0 Å. Folding around each tpbz intrachelate P···P axis such that the MP_2_/M′P_2_ planes meet the tpbz P_2_C_6_P_2_ mean plane at non-zero values gives rise to core topologies that appear “S-like” or herringbone-like for M′ = Pt^2+^ or {ReBr(CO)}/{Re(CO)_2_}^+^. Calculations reveal that departure from idealized *D*_2*h*_/*D*_2*d*_/*C*_2*v*_ symmetries is induced by steric crowding between Ph groups and that dynamic, fluxional behavior is pertinent to the solution phase because multiple, lower-symmetry minima of comparable energy exist. Spectroscopically, the formation of the trimetallic arrays is marked by a shift of the open end ^31^P nuclear magnetic resonance signal from approximately −14.5 ppm to approximately +41, approximately +20.5, and approximately +28.5 ppm for M′ = Pt^2+^, Au^+^, and {ReBr(CO)}/{Re(CO)_2_}^+^, respectively. Electrochemically, dithiolene-based oxidations are observed for the R = Ph and M′ = Pt^2+^ or Au^+^ compounds but at potentials that are anodically shifted relative to charge-neutral [[(R_2_C_2_S_2_)M]_2_(μ-tpbz)]. The compounds reported clarify the possibilities for the synthesis of assemblies in which weakly coupled spins may be created in their modular (R_2_C_2_S_2_)M and M′ parts.

## Abstract 2

Metallodithiolene complexes [(R_2_C_2_S_2_)M(η^2^-tpbz)] [R = CN, Ph, or *p*-anisyl; M = Ni^2+^, Pd^2+^, or Pt^2+^; tpbz = 1,2,4,5-tetrakis(diphenylphosphino)benzene] form trimetallic arrays [[(R_2_C_2_S_2_)M(tpbz)]_2_M′]^*n*+^, where M′ is square planar Pt^2+^, tetrahedral Cu^+^, Ag^+^, or Au^+^, or octahedral {ReBr(CO)}/{Re(CO)_2_}^+^. Twelve members of the set have been crystallographically characterized. Electrochemically, dithiolene-based oxidations are observed for R = Ph and M′ = Pt^2+^ or Au^+^ at potentials anodically shifted relative to [[(R_2_C_2_S_2_)M]_2_(μ-tpbz)]^0^, with *J* ≈ 0 because of interspin separations of ∼23 Å.

## Introduction

The arena of quantum information sciences is now widely recognized as strategically significant ground for the economy and security of the future.^1^ This broadly ranging theme includes, *inter alia*, development of a robust and scalable physical platform that can support coherent quantum states that form the basis for memory and logic operations. Among the various scaffolds that have been considered for quantum bits (qubits) in quantum computing and quantum memory applications, electron spins hosted within discrete molecules or coordination complexes have some distinct advantages over other systems.^2^ One such advantage is the power of synthetic chemistry to vary the separation between spins such that the coupling between them is strong enough to support a coherent quantum state but weak enough to move coherence lifetimes into the time domain necessary for the execution of logic operations. Another advantage is a capacity to change the relative spatial orientation of two or more spins within a host molecule such that one spin might be manipulated selectively over another by an appropriately directed pulse.^3^ This selectivity is a basis for addressability.^4^

**Scheme 1:**
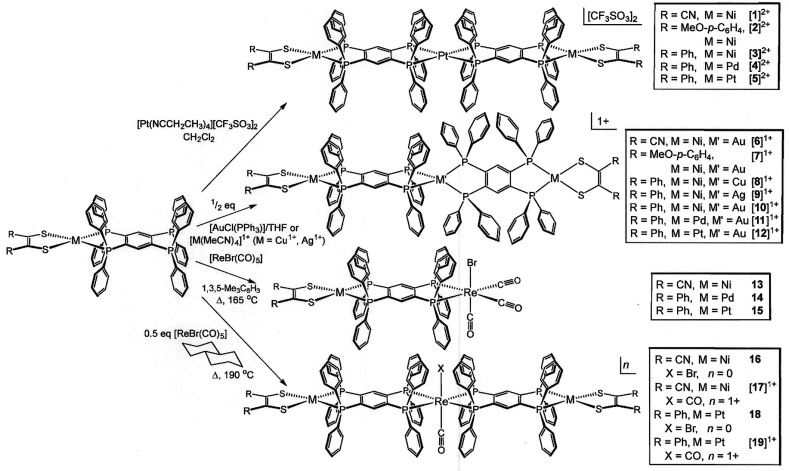
Trimetallic Assemblies with Metallodithiolene End Groups and tpbz Bridging Ligands

Bearing the forgoing general ideas in mind, we have elaborated upon the coordination chemistry of group 10 dithiolene diphosphine complexes, which generally sustain one, or sometimes two, reversible redox processes at the dithiolene ligand. When the diphosphine used is 1,2,4,5-tetrakis(diphenylphosphino)benzene (tpbz), centrosymmetric dimetallic compounds that support identical, very nearly concurrent oxidations at the dithiolene end groups can be prepared.^5,6^ The diradical thus created, with dipolar coupling of ∼16 cm^–1^, forms the basis for a diqubit system.^7^ In the course of these studies, we noted that the synthesis conditions can be modified to favor the formation of “open-ended” compounds (Scheme 1, left).^8^ Among other possibilities, these open-ended compounds provide for the introduction of asymmetry with different dithiolene ligands featuring different redox potentials. They also may be implemented as “ligands” in their own right, thereby arraying about some central ion two, or possibly three, metallodithiolene moieties in a well-defined geometry. Because such multimetal assemblies enable still further possibilities for weakly coupled distal spins, possibly but not necessarily involving the ensconced central ion, we have targeted the synthesis of a variety of such complexes and report herein the leading results of a broad synthetic foray into such compounds with attending structural, electrochemical, and spectroscopic characterization.

Work from the Freedman group has highlighted the great promise of dithiolene coordination compounds as hosts for electron spin qubits. In systems designed to minimize the presence of spin-active nuclei that contribute to the decoherence of qubit superposition states, coherence lifetimes of ∼1 ms have been achieved.^9,10^ The work reported here differs from that of the Freedman group in that the electron spins can be reversibly generated at the organic dithiolene ligands rather than existing within the d-electron manifold of the transition metal. More recently, possibilities for selective optical addressability of ground state spins in discrete molecules have been disclosed.^11^ The results described herein regarding both the incorporation of photoactive third-row transition metal ions into metallodithiolene arrays and the orthogonal disposition of dithiolene-based electron spins relate to this very powerful design concept in qubit engineering.

## Experimental Section

### Physical Methods

All ^1^H and ^31^P nuclear magnetic resonance (NMR) spectra were recorded at 25 °C with either a Bruker Avance spectrometer operating at 300.13 and 121.49 MHz for the ^1^H and ^31^P nuclei, respectively, or a Bruker Ascend 400 spectrometer operating at 162.11 MHz for ^31^P. The ^1^H NMR spectra were referenced to the solvent signal, while an external aqueous H_3_PO_4_ solution was employed as a reference for all ^31^P spectra. Ultraviolet–visible (UV–vis) spectra were recorded at ambient temperature with a Hewlett-Packard 8455A diode array spectrometer, while IR spectra were recorded as KBr pellets using a Thermo Nicolet Nexus 670 Fourier transform infrared instrument in absorption mode. Mass spectra (ESI+) were recorded with a Bruker micrOTOF II mass spectrometer. All observed and calculated masses represent the most intense peak in the mass envelope pattern. The [**8**]^3+^ and [**12**]^3+^ cation radicals were generated by chemical oxidation of [**8**]^+^ and [**12**]^+^ using 2 equiv of [(Br-*p*-C_6_H_4_)_3_N][SbCl_6_]. X-Band electron paramagnetic resonance (EPR) spectra were recorded on a Bruker ELEXSYS E500 spectrometer, and simulations were performed using *XSophe*.^12^ Electrochemical measurements were taken with a CH Instruments 620C electroanalyzer workstation using a Ag/AgCl reference electrode, a platinum disk working electrode, Pt wire as the auxiliary electrode, and [^*n*^Bu_4_N][PF_6_] as the supporting electrolyte. Under these conditions, the Cp_2_Fe^+^/Cp_2_Fe (Fc^+^/Fc) couple consistently occurred at 0.436 V in CH_2_Cl_2_ and at 0.500 V in *N*,*N*-dimethylformamide. Procedural details regarding crystal growth, X-ray diffraction data collection, data processing, and structure solution and refinement are available in the Supporting Information. Unit cell data and selected refinement statistics for the compounds that have been structurally identified are listed in Table 1; more complete crystallographic data are summarized in Tables S1–S4. A description of the methodologies employed, the theory level implemented, and other details pertinent to the computations is deposited in the Supporting Information.

**Table 1:** Unit Cell and Refinement Data for Crystallographically Characterized Trimetallic Compounds

	[**1**][CF_3_SO_3_]_2_	[**3**][CF_3_SO_3_]_2_	[**4**][CF_3_SO_3_]_2_	[**5**][CF_3_SO_3_]_2_
formula	C_144_H_119_N_7_O_14_F_6_P_8_S_6_Ni_2_Pt	C_147_H_129_N_3_O_9_F_6_P_8_S_6_Ni_2_Pt	C_148.5_H_128.5_N_3.5_O_11.5_F_6_P_8_S_6_Pd_2_Pt	C_150_H_136_N_4_O_12_F_6_P_8_S_6_Pt_3_
fw (g/mol)	3038.08	2944.12	3108.05	3326.01
crystal system	triclinic	monoclinic	monoclinic	monoclinic
space group	*P*1̅	*P*2_1_/*n*	*P*2_1_/*n*	*P*2_1_/*n*
*a* (Å)	13.592(3)	18.0711(13)	18.1595(15)	18.176(3)
*b* (Å)	14.064(3)	16.1273(12)	15.9710(13)	15.956(3)
*c* (Å)	21.188(5)	24.8605(18)	24.785(2)	24.813(4)
α (deg)	73.458(3)	90	90	90
β (deg)	89.326(3)	94.710(2)	92.570(2)	92.568(2)
γ (deg)	63.186(3)	90	90	90
volume (Å^3^)	3432.9(13)	7220.8(9)	7181.0(10)	7189(2)
*Z*	1	2	2	2
no. of independent reflections	14975	10390	11475	8843
goodness of fit^a^	1.015	1.028	1.028	1.054
R1,^b^,^c^ wR2^c^,^d^	0.0677, 0.1614	0.0761, 0.2093	0.0820, 0.2350	0.0790, 0.1671
R1,^b^,^e^ wR2^d^,^e^	0.1041, 0.1771	0.1327, 0.2575	0.1208, 0.2798	0.1365, 0.1992

	[**6**][CF_3_SO_3_]	[**7**][Cl]	[**10**][CF_3_SO_3_]	[**11**][CF_3_SO_3_]
formula	C_134_H_107.5_N_4.5_O_5_F_3_P_8_S_5_Ni_2_Au	C_142.5_H_117_NO_4.5_P_8_S_4_ClNi_2_Au	C_149_H_129_N_4_O_5.5_F_3_P_8_S_5_Ni_2_Au	C_137_H_104_O_3_F_3_P_8_S_5_Pd_2_Au
fw (g/mol)	2640.19	2641.20	2843.00	2673.02
crystal system	monoclinic	trigonal	monoclinic	tetragonal
space group	*C*2	*P*3_1_*c*	*P*2_1_/*n*	*I*4_1_/*a*
*a* (Å)	36.6060(12)	28.9658(4)	16.9691(7)	40.930(3)
*b* (Å)	20.5956(7)	28.9658(4)	26.4603(10)	40.930(3)
*c* (Å)	21.1856(7)	35.2990(15)	32.2338(12)	34.252(4)
α (deg)	90	90	90	90
β (deg)	123.408(1)	90	92.246(1)	90
γ (deg)	90	120	90	90
volume (Å^3^)	13333.2(8)	25648.6(18)	14462.1(10)	57382(11)
*Z*	4	6	4	16
no. of independent reflections	36107	26413	36255	24484
goodness of fit^a^	1.066	1.050	1.159	1.100
R1,^b^,^c^ wR2^c^,^d^	0.0463, 0.1036	0.0400, 0.1076	0.1198, 0.2876	0.0762, 0.1944
R1,^b^,^e^ wR2^d^,^e^	0.0614, 0.1105	0.0451, 0.1122	0.1504, 0.3049	0.1210, 0.2537

	**16**	[**17**][Br]	**18**	[**19**][Br]
formula	C_117_H_84_N_4_OP_8_S_4_Ni_2_BrRe	C_118_H_84_N_4_O_2_P_8_S_4_Ni_2_BrRe	C_161_H_124_N_4_O_9_P_8_S_4_BrRePt_2_	C_161_H_114_N_3_O_9_P_8_S_4_BrRePt_2_
fw (g/mol)	2321.41	2349.42	3290.92	3266.83
crystal system	monoclinic	monoclinic	monoclinic	monoclinic
space group	*P*2_1_/*n*	*P*2_1_/*n*	*P*2_1_/*c*	*P*2_1_/*c*
*a* (Å)	14.0537(10)	13.924(3)	14.2385(7)	14.0974(14)
*b* (Å)	24.4013(17)	23.264(5)	21.6579(11)	21.564(2)
*c* (Å)	20.3377(14)	19.354(4)	23.3488(12)	23.225(2)
α (deg)	90	90	90	90
β (deg)	108.650(2)	92.803(2)	96.601(2)	96.407
γ (deg)	90	90	90	90
volume (Å^3^)	6608.1(8)	6262(2)	7152.5(6)	7016.4(12)
*Z*	2	2	2	2
no. of independent reflections	8112	2180	14769	17463
goodness of fit^a^	1.011	1.072	1.119	1.009
R1,^b^,^c^ wR2^c^,^d^	0.0716, 0.2030	0.1056, 0.2776	0.0701, 0.1558	0.0789, 0.1596
R1,^b^,^e^ wR2^d^,^e^	0.1068, 0.2363	0.1265, 0.3023	0.1028, 0.1786	0.2013, 0.2086

a Goodness of fit = {∑[*w*(*F*_0_^2^ – *F*_c_^2^)^2^]/(*n* – *p*)}^1/2^, where *n* is the number of reflections and *p* is the total number of parameters refined.

b R1 = ∑||*F*_0_| – |*F*_c_||/∑|*F*_0_|.

c R indices for data cutoff at *I* > 2σ(*I*).

d wR2 = {∑[*w*(*F*_0_^2^ – *F*_c_^2^)^2^]/∑[*w*(*F*_0_^2^)^2^]}^1/2^; *w* = 1/[σ^2^(*F*_0_^2^) + (*xP*)^2^ + *yP*], where *P* = [2*F*_c_^2^ + max(*F*_0_^2^, 0)]/3.

e R indices for all data.

### General Considerations

Literature methods were implemented for the syntheses of the tpbz ligand,^13^ all [(R_2_C_2_S_2_)M(η^2^-tpbz)] complexes,^8^ [Pt(N≡CCH_2_CH_3_)_4_][CF_3_SO_3_]_2_,^14^ [(MeCN)_4_Cu][PF_6_],^15a^ and [(MeCN)_4_Ag][BF_4_].^15b^ All other reagents were purchased from commercial sources and used as received. Solvents were either dried with a system of drying columns from the Glass Contour Company (CH_2_Cl_2_, *n*-pentane, hexanes, Et_2_O, THF, C_6_H_6_, and toluene) or freshly distilled according to standard procedures (MeOH, CH_3_CN, and 1,2-dichloroethane).^16^ All reactions described below were conducted under an atmosphere of N_2_, unless otherwise indicated, while silica columns were run in the open air using 60–230 μm silica (Dynamic Adsorbents). Abbreviations used throughout the text are as follows: mnt = maleonitriledithiolate(2−) = [(NC)_2_C_2_S_2_]^2–^; pdt = [Ph_2_C_2_S_2_]^2–^ = 1,2-diphenyl-1,2-ethylenedithiolate(2−); adt = [(MeO-*p*-C_6_H_4_)_2_C_2_S_2_]^2–^ = 1,2-di-*p*-anisyl-1,2-ethylenedithiolate(2−); dppb = 1,2-bis(diphenylphosphino)benzene; [BArF_24_]^−^ = tetrakis[3,5-bis(trifluoromethyl)phenyl]borate(1−).

## Syntheses

### [((NC)_2_C_2_S_2_)Ni(μ-tpbz)Pt(μ-tpbz)Ni(S_2_C_2_(CN)_2_)][CF_3_SO_3_]_2_, [**1**][CF_3_SO_3_]_2_

Under a N_2_ atmosphere, a solution of [(mnt)Ni(η^2^-tpbz)] (0.100 g, 0.0986 mmol) in CH_2_Cl_2_ (20 mL) was transferred dropwise via cannula to a solution of [Pt(NCCH_2_CH_3_)_4_][CF_3_SO_3_]_2_ (0.0352 g, 0.0493 mmol) dissolved in CH_2_Cl_2_ (5 mL). This reaction mixture was stirred for 12 h at 25 °C. The light brown precipitate that was produced was separated by filter cannulation from the colorless supernatant, washed with CH_2_Cl_2_ (2 × 5 mL) and then Et_2_O (2 × 5 mL), and dried *in vacuo*. Crystallization was accomplished by diffusion of Et_2_O vapor into a filtered solution of [**1**][CF_3_SO_3_]_2_ in a minimal volume of C_6_H_5_NO_2_. Yield: 0.103 g, 84%. ^1^H NMR (δ, DMSO-*d*_6_): 7.75–7.72 (m, 7 H, aromatic C–H), 7.65–7.56 (m, 19 H, aromatic C–H), 7.48–7.43 (m, 32 H, aromatic C–H), 7.24–7.19 (m, 14 H, aromatic C–H), 7.10–7.05 (m, 12 H, aromatic C–H). ^31^P{^1^H} NMR (δ, DMSO-*d*_6_): 59.15 (s), 42.41 (s). UV–vis [DMF, λ_max_, nm (ε)]: 369 (7170), 423 (2330). IR (KBr, cm^–1^): 2203 (asym *v*_C≡N_), 2216 (sym *v*_C≡N_). MS (ESI^+^) calcd for [C_116_H_84_N_4_Ni_2_P_8_PtS_4_]^2+^: *m*/*z* 1111.0928. Observed: *m*/*z* 1111.0855. Error (δ): 6.57 ppm.

### [((MeO-*p*-C_6_H_4_)_2_C_2_S_2_)Ni(μ-tpbz)Pt(μ-tpbz)Ni(S_2_C_2_(C_6_H_4_-*p*-OMe)_2_)][CF_3_SO_3_]_2_, [**2**][CF_3_SO_3_]_2_

The same procedure and scale as described for the synthesis of [**3**][CF_3_SO_3_]_2_ (*vide infra*) were employed but with [((MeO-*p*-C_6_H_4_)_2_C_2_S_2_)Ni(tpbz)] (0.117 g, 0.0995 mmol) used in place of [(Ph_2_C_2_S_2_)Ni(tpbz)]. Yield: 0.095 g of brown solid, 75%. ^1^H NMR (δ, DMSO-*d*_6_): 7.73–7.38 (m, 57 H, aromatic C–H), 7.23–7.12 (m, 15 H, aromatic C–H), 7.05–7.00 (m, 7 H, aromatic C–H), 6.90–6.85 (m, 12 H, aromatic C–H), 6.63–6.60 (m, 9 H, aromatic C–H), 3.63 (s, 12 H, -OCH_3_). ^31^P{^1^H} NMR (δ, DMSO-*d*_6_): 54.59 (s), 41.03 (s). MS (ESI^+^) calcd for [C_140_H_112_Ni_2_O_4_P_8_PtS_4_]^2+^: *m*/*z* 1273.1855. Observed: *m*/*z* 1273.1641. Error (δ): 16.84 ppm.

### [(Ph_2_C_2_S_2_)Ni(μ-tpbz)Pt(μ-tpbz)Ni(S_2_C_2_Ph_2_)][CF_3_SO_3_]_2_, [**3**][CF_3_SO_3_]_2_

Under an atmosphere of N_2_, a 25 mL Schlenk flask was charged with [(Ph_2_C_2_S_2_)Ni(tpbz)] (0.111 g, 0.0995 mmol) in 20 mL of CH_2_Cl_2_, while a solution of [Pt(NCCH_2_CH_3_)_4_][CF_3_SO_3_]_2_ (0.035 g, 0.050 mmol) in 5 mL of CH_2_Cl_2_ was composed in a separate 50 mL Schlenk flask. The green solution of [(Ph_2_C_2_S_2_)Ni(tpbz)] was transferred dropwise via cannula to the colorless [Pt(NCCH_2_CH_3_)_4_][CF_3_SO_3_]_2_ solution. The resulting reaction mixture was stirred overnight (14 h) at ambient temperature, during which time a dark brown solution developed. The reaction mixture was thereupon reduced to a volume of ∼3 mL, and hexanes (∼10 mL) were added via syringe to precipitate [**3**][CF_3_SO_3_]_2_ as a brown powder. The supernatant was removed by cannula filtration, and the solid residue was then washed with Et_2_O (2 × 8 mL) and dried under vacuum. Purification of [**3**][CF_3_SO_3_]_2_ was accomplished by layering a dry 0.040 g sample onto a 0.50 in. thick Celite pad in a filter pipet. Dichloromethane (2 × 3 mL) was passed through the crude complex to remove any unreacted open-ended [(Ph_2_C_2_S_2_)Ni(tpbz)], dimetallic [(Ph_2_C_2_S_2_)Ni(μ-tpbz)Ni(S_2_C_2_Ph_2_)], and other soluble impurities until the filtrate was colorless. The settled thin brown layer of trimetallic [**3**][CF_3_SO_3_]_2_ on the Celite pad was recovered by extraction into *N*,*N*-dimethylformamide. X-ray quality crystals of [**3**][CF_3_SO_3_]_2_·4DMF·2Et_2_O were grown by diffusion of Et_2_O vapor into a filtered concentrate in *N*,*N*-dimethylformamide. Yield: 0.100 g of brown solid, 79%. ^1^H NMR (δ, DMSO-*d*_6_): 7.55–7.38 (m, 52 H, aromatic C–H), 7.27–7.17 (m, 20 H, aromatic C–H), 7.07–7.01 (m, 20 H, aromatic C–H), 6.95–6.90 (m, 12 H, aromatic C–H). ^31^P{^1^H} NMR (δ, DMSO-*d*_6_): 55.87 (s), 41.05 (s). MS (ESI^+^) calcd for [C_136_H_104_Ni_2_P_8_PtS_4_]^2+^: *m*/*z* 1213.1486. Observed: *m*/*z* 1213.1643. Error (δ): 12.95 ppm.

### [(Ph_2_C_2_S_2_)Pd(μ-tpbz)Pt(μ-tpbz)Pd(S_2_C_2_Ph_2_)][CF_3_SO_3_]_2_, [**4**][CF_3_SO_3_]_2_

The same procedure and scale as implemented for the synthesis of [**3**][CF_3_SO_3_]_2_ were employed but with [(Ph_2_C_2_S_2_)Pd(tpbz)] used in place of [(Ph_2_C_2_S_2_)Ni(tpbz)]. The same purification protocol as given for [**3**][CF_3_SO_3_]_2_ was also followed. Yield: 0.089 g of purple solid, 70%. ^1^H NMR (δ, DMSO-*d*_6_): 7.53–7.44 (m, 52 H, aromatic C–H), 7.24–7.16 (m, 17 H, aromatic C–H), 7.04–6.93 (m, 35 H, aromatic C–H). ^31^P{^1^H} NMR (δ, DMSO-*d*_6_): 48.92 (s), 40.75 (s). MS (ESI^+^) calcd for [C_136_H_104_P_8_Pd_2_PtS_4_]^2+^: *m*/*z* 1260.6343. Observed: *m*/*z* 1260.6213. Error (δ): 10.36 ppm.

### [(Ph_2_C_2_S_2_)Pt(μ-tpbz)Pt(μ-tpbz)Pt(S_2_C_2_Ph_2_)][CF_3_SO_3_]_2_, [**5**][CF_3_SO_3_]_2_

The same procedure and scale as implemented for the synthesis of [**3**][CF_3_SO_3_]_2_ were employed but with [(Ph_2_C_2_S_2_)Pt(tpbz)] (0.125 g, 0.0998 mmol) used in place of [(Ph_2_C_2_S_2_)Ni(tpbz)]. The same purification protocol as given for [**3**][CF_3_SO_3_]_2_ was also followed. Yield: 0.081 g of red solid. ^1^H NMR (δ, DMSO-*d*_6_): 7.97–7.95 (m, 4 H, aromatic C–H), 7.56–7.44 (m, 51 H, aromatic C–H), 7.25–7.18 (m, 17 H, aromatic C–H), 7.07–7.02 (m, 24 H, aromatic C–H), 6.96–6.92 (m, 8 H, aromatic C–H). ^31^P{^1^H} NMR (δ, DMSO-*d*_6_): 43.69 (s, *J*_Pt–P_ = 2744 Hz), 40.50 (s, *J*_Pt–P_ = 2345 Hz). UV–vis [DMF, λ_max_, nm (ε)]: 437 (3150), 510 (2850). MS (ESI^+^) calcd for [C_136_H_104_P_8_Pt_3_S_4_]^2+^: *m*/*z* 1349.1956. Observed: *m*/*z* 1349.1987. Error (δ): 2.32 ppm.

### [((NC)_2_C_2_S_2_)Ni(μ-tpbz)Au(μ-tpbz)Ni(S_2_C_2_(CN)_2_)][CF_3_SO_3_], [**6**][CF_3_SO_3_]

Under an atmosphere of N_2_, a red solution of [(mnt)Ni(tpbz)] (0.102 g, 0.101 mmol) in dry THF (5 mL) was transferred dropwise via cannula to a colorless solution of Au(PPh_3_)Cl (0.025 g, 0.051 mmol) in THF (5 mL). During the course of this addition, immediate formation of a yellow brown precipitate was observed. The reaction mixture was stirred for 8 h at ambient temperature, after which time the solvent was removed by cannula filtration, and the solid residue was dried under vacuum. To this dried crude solid was added Ag[CF_3_SO_3_] (0.0128 g, 0.0498 mmol) in a 20:1 CH_2_Cl_2_/MeOH solvent (21 mL). This mixture was stirred overnight (12 h) at ambient temperature in the dark. The solution was filtered through a 1 in. Celite pad on a glass frit to remove AgCl. The filtrate was reduced to a volume of ∼3 mL, and 10 mL of hexanes were then added via syringe to precipitate [**6**][CF_3_SO_3_] as a yellow-brown solid. The supernatant was removed by cannula filtration, and the residual solid was washed with hexanes (8 mL) and then Et_2_O (2 × 8 mL) and dried under vacuum. Further purification was achieved on a silica chromatography column eluted with a 5:95 THF/CH_2_Cl_2_ mixture, which moved [**6**][CF_3_SO_3_] as a yellow-brown band. Recrystallization was accomplished by the diffusion of Et_2_O into a filtered PhNO_2_ or 1,3-dimethyl-2-imidazolidinone solution of the complex. Yield: 0.093 g of yellow-brown solid, 79%. ^1^H NMR (δ, CD_2_Cl_2_): 7.51–7.40 (m, 14 H, aromatic C–H), 7.34–7.29 (m, 10 H, aromatic C–H), 7.35–7.21 (m, 30 H, aromatic C–H), 7.03–6.98 (m, 15 H, aromatic C–H), 6.86–6.83 (m, 15 H, aromatic C–H). ^31^P{^1^H} NMR (δ, CD_2_Cl_2_): 56.30 (s), 21.21 (s). UV–vis [CH_2_Cl_2_, λ_max_, nm (ε)]: 365 (sh, 18600), 405 (sh, 9680), ∼575 (210). IR (KBr, cm^–1^): 2127 (asym *v*_C≡N_), 2156 (sym *v*_C≡N_). MS (ESI^+^) calcd for [C_116_H_84_AuN_4_Ni_2_P_8_S_4_]^+^: *m*/*z* 2223.1871. Observed: *m*/*z* 2223.1795. Error (δ): 3.45 ppm.

### [((MeO-*p*-C_6_H_4_)_2_C_2_S_2_)Ni(μ-tpbz)Au(μ-tpbz)Ni(S_2_C_2_(C_6_H_4_-*p*-OMe)_2_)][Cl], [**7**][Cl]

The same procedure and scale as described for the synthesis of [**10**][CF_3_SO_3_] (*vide infra*) were employed but with [((MeO-*p*-C_6_H_4_)_2_C_2_S_2_)Ni(tpbz)] (0.117 g, 0.0995 mmol) implemented in place of [(Ph_2_C_2_S_2_)Ni(μ-tpbz)]. The purification procedure described for [**10**][CF_3_SO_3_] was also applied. Although [CF_3_SO_3_]^−^ was the intended counteranion, the anion in the isolated product was Cl^–^, as determined by X-ray crystallography. Yield: 0.093 g of brown solid, 69%. *R*_*f*_ = 0.19 (9:1 DCM/THF). ^1^H NMR (δ, CD_2_Cl_2_): 7.51–7.37 (m, 42 H, aromatic C–H), 7.35–7.30 (m, 16 H, aromatic C–H), 7.23–7.03 (m, 24 H, aromatic C–H), 6.96–6.83 (m, 12 H, aromatic C–H), 6.57–6.54 (m, 6 H, aromatic C–H), 3.65 (s, 12 H, -OCH_3_). ^31^P{^1^H} NMR (δ, CD_2_Cl_2_): 53.76 (s), 20.13 (s). UV–vis [CH_2_Cl_2_, λ_max_, nm (ε)]: 490 (3070), ∼625 (1090). MS (ESI^+^) calcd for [C_140_H_112_AuNi_2_O_4_P_8_S_4_]^+^: *m*/*z* 2547.3724. Observed: *m*/*z* 2547.3735. Error (δ): 0.43 ppm.

### [(Ph_2_C_2_S_2_)Ni(μ-tpbz)Cu(μ-tpbz)Ni(S_2_C_2_Ph_2_)][BArF_24_], [**8**][BArF_24_]

In a scintillation vial, [(Ph_2_C_2_S_2_)Ni(tpbz)] (0.100 g, 0.0896 mmol) was combined with [Cu(N≡CCH_3_)_4_][PF_6_] (0.018 g, 0.048 mmol) in CH_2_Cl_2_ (10 mL) and stirred at ambient temperature for 1 h. The dark green reaction mixture was treated with NaBArF_24_ (0.043 g, 0.054 mmol) and left to stir overnight. The mixture was then filtered to remove the insoluble residue. The dropwise addition of MeOH induced precipitation of [**8**][BArF_24_], which was collected on a frit, washed with MeOH and then Et_2_O, and dried under vacuum. Yield: 0.113 g, 80%. ^31^P{^1^H} NMR (δ, CD_2_Cl_2_): 54.46 (s), 7.46 (br, s). UV–vis [CH_2_Cl_2_, λ_max_, nm (ε)]: 464 (3480). 620 (950). MS (ESI^+^) calcd for [C_136_H_104_CuNi_2_P_8_S_4_]^+^: *m*/*z* 2294.2937. Observed: *m*/*z* 2294.3171. Error (δ): 10.2 ppm. MS (ESI^+^) calcd for [(Ph_2_C_2_S_2_)Ni(μ-tpbz)Cu]^+^, [C_68_H_52_CuNiP_4_S_2_]^+^: *m*/*z* 1179.1089. Observed: *m*/*z* 1179.1023. Error (δ): 5.6 ppm. MS (ESI^+^) calcd for [(Ph_2_C_2_S_2_)Ni(tpbz)]^+^, [C_68_H_52_NiP_4_S_2_]^+^: *m*/*z* 1114.1809. Observed: *m*/*z* 1114.1501. Error (δ): 27.6 ppm.

### [(Ph_2_C_2_S_2_)Ni(μ-tpbz)Ag(μ-tpbz)Ni(S_2_C_2_Ph_2_)][BF_4_], [**9**][BF_4_]

In a 20 mL glass scintillation vial, [(Ph_2_C_2_S_2_)Ni(tpbz)] (0.117 g, 0.105 mmol) was dissolved in CH_2_Cl_2_ (5 mL). To this solution was added solid [(MeCN)_4_Ag][BF_4_] (0.0188 g, 0.052 mmol), and the reaction mixture was then diluted with MeCN (5 mL). After the mixture had been stirred for 2 h at ambient temperature, all volatiles were removed under reduced pressure. The residual solid was triturated with MeOH (∼20 mL), collected as an olive-green powder by filtration onto a fine porosity glass frit, and dried in the open air. Yield: 0.0823 g, 33%. ^31^P{^1^H} NMR (δ, CD_2_Cl_2_): 54.74 (s), −0.68 (d, *J*_^31^P-^107^Ag_ = 234 Hz), −0.69 (d, *J*_^31^P-^109^Ag_ = 262 Hz). MS (ESI^+^) calcd for [C_272_H_208_Ag_2_Ni_4_P_16_S_8_]^2+^: *m*/*z* 2339.2686. Observed: *m*/*z* 2339.2595. Error (δ): 3.87 ppm. The ESI-MS fit is best described as a mixture of a monocation and a dimeric dication.

### [(Ph_2_C_2_S_2_)Ni(μ-tpbz)Au(μ-tpbz)Ni(S_2_C_2_Ph_2_)][CF_3_SO_3_], [**10**][CF_3_SO_3_]

Under N_2_, a green solution of [(Ph_2_C_2_S_2_)Ni(μ-tpbz)] (0.111 g, 0.0995 mmol) in dry THF (5 mL) was added dropwise via cannula to a colorless solution of [AuCl(PPh_3_)] (0.025 g, 0.051 mmol) in THF (5 mL), which immediately induced formation of a dark brown color. This reaction mixture was stirred for 8 h at 25 °C and then reduced to dryness. To the crude solid residue were added Ag[CF_3_SO_3_] (0.0128 g, 0.0498 mmol) and a 20:1 CH_2_Cl_2_/MeOH mixture (21 mL). Stirring was continued for 12 h at 25 °C in the dark. The resulting suspension of AgCl was removed by filtration through a Celite pad, and the filtrate was reduced *in vacuo* to a volume of 3 mL. Addition of hexanes (10 mL) induced precipitation of [**10**][CF_3_SO_3_] as a brown solid, which then was separated by filtration, washed with hexanes (8 mL) followed by Et_2_O (2 × 8 mL), and dried *in vacuo*. Further purification was accomplished on a silica column eluted with (95:5 CH_2_Cl_2_/THF) followed by crystallization by diffusion of Et_2_O vapor into a 1,3-dimethyl-2-imidazolidinone solution. Yield: 0.096 g, 75%. *R*_*f*_ = 0.26 (9:1 CH_2_Cl_2_/THF). ^1^H NMR (δ, CD_2_Cl_2_): 7.52–7.50 (m, 4 H, aromatic C–H), 7.42–7.31 (m, 33 H, aromatic C–H), 7.25–7.20 (m, 16 H, aromatic C–H), 7.08–6.99 (m, 36 H, aromatic C–H), 6.88–6.82 (m, 15 H, aromatic C–H). ^31^P{^1^H} NMR (δ, CD_2_Cl_2_): 54.15 (s), 20.57 (s). UV–vis [CH_2_Cl_2_, λ_max_, nm (ε)]: 470 (4280), ∼625 (1020). MS (ESI^+^) calcd for [C_136_H_104_AuNi_2_P_8_S_4_]^1+^: *m*/*z* 2427.3323. Observed: *m*/*z* 2427.3263. Error (δ): 2.5 ppm.

### [(Ph_2_C_2_S_2_)Pd(μ-tpbz)Au(μ-tpbz)Pd(S_2_C_2_Ph_2_)][CF_3_SO_3_], [**11**][CF_3_SO_3_]

The same procedure and scale as described for the synthesis of [**10**][CF_3_SO_3_] were employed but with [(Ph_2_C_2_S_2_)Pd(tpbz)] (0.116 g, 0.0997 mmol) used in place of [(Ph_2_C_2_S_2_)Ni(tpbz)]. The purification procedure described for [**10**][CF_3_SO_3_] was also applied. Yield: 0.098 g of purple-brown solid, 74%. *R*_*f*_ = 0.32 (9:1 CH_2_Cl_2_/THF). ^1^H NMR (δ, CD_2_Cl_2_): 7.63–7.58 (m, 4 H, aromatic C–H), 7.42–7.31 (m, 35 H, aromatic C–H), 7.25–7.20 (m, 16 H, aromatic C–H), 7.07–6.99 (m, 35 H, aromatic C–H), 6.87–6.84 (m, 14 H, aromatic C–H). ^31^P{^1^H} NMR (δ, CD_2_Cl_2_): 47.59 (s), 20.48 (s). UV–vis [CH_2_Cl_2_, λ_max_, nm (ε)]: 480 (3950). MS (ESI^+^) calcd for [C_136_H_104_AuP_8_Pd_2_S_4_]^+^: *m*/*z* 2523.2453. Observed: *m*/*z* 2523.2707. Error (δ): 10.07 ppm.

### [(Ph_2_C_2_S_2_)Pt(μ-tpbz)Au(μ-tpbz)Pt(S_2_C_2_Ph_2_)][CF_3_SO_3_], [**12**][CF_3_SO_3_]

The same procedure and scale as described for the synthesis of [**10**][CF_3_SO_3_] were employed but with [(Ph_2_C_2_S_2_)Pt(tpbz)] (0.125 g, 0.0998 mmol) used in place of [(Ph_2_C_2_S_2_)Ni(μ-tpbz)]. The purification procedure described for [**10**][CF_3_SO_3_] was also applied. Yield: 0.118 g of orange-red solid, 83%. *R*_*f*_ = 0.31 (9:1 CH_2_Cl_2_/THF), ^1^H NMR (δ, CD_2_Cl_2_): 7.68–7.65 (m, 4 H, aromatic C–H), 7.45–7.32 (m, 45 H, aromatic C–H), 7.25–7.16 (m, 20 H, aromatic C–H), 7.04–6.99 (m, 24 H, aromatic C–H), 6.88–6.86 (m, 11 H, aromatic C–H). ^31^P{^1^H} NMR (δ, CD_2_Cl_2_): 42.61 (s, *J*_Pt–P_ = 2746 Hz), 20.48 (s). UV–vis [CH_2_Cl_2_, λ_max_, nm (ε)]: 465 (5290). MS (ESI^+^) calcd for [C_136_H_104_AuP_8_Pt_2_S_4_]^+^: *m*/*z* 2701.3687. Observed: *m*/*z* 2701.3918. Error (δ): 8.53 ppm.

### [((NC)_2_C_2_S_2_)Ni(μ-tpbz)Re(CO)_3_Br], **13**

Under a N_2_ atmosphere, a 50 mL Schlenk flask was charged with solid [(mnt)Ni(tpbz)] (0.111 g, 0.109 mmol) and [Re(CO)_5_Br] (0.040 g, 0.098 mmol). Dry mesitylene (10 mL) was transferred to the reaction mixture via syringe. The reaction mixture was heated to reflux for 48 h, during which time it assumed a dark brown-red color. Once the mixture had cooled, a solid residue was deposited. The solvent was removed by cannula filtration, and the solid thus isolated was washed with hexanes (8 mL) followed by Et_2_O (2 × 8 mL) and then dried *in vacuo*. This crude product was further purified on a silica column eluted with a 90:10 CH_2_Cl_2_/THF mixture, which moved **13** as a brown-orange band. Single crystals of diffraction quality were grown by the diffusion of Et_2_O vapor into a solution in CH_2_Cl_2_. Yield: 0.091 g of yellow-brown solid, 67%. ^31^P{^1^H} NMR (δ, CDCl_3_): 54.81 (s), 28.91 (s). UV–vis [CHCl_3_, λ_max_, nm (ε)]: 425 (sh, 3890). IR (KBr, cm^–1^): 2038 (asym *v*_C≡N_), 2053 (sym *v*_C≡N_). MS (ESI^+^) calcd for [C_61_H_42_BrN_2_NiO_3_P_4_ReS_2_ + H^+^]^+^: *m*/*z* 1364.9735. Observed: *m*/*z* 1364.9630. Error (δ): 7.66 ppm.

### [(Ph_2_C_2_S_2_)Pd(μ-tpbz)Re(CO)_3_Br], **14**

The same procedure and scale as described for the synthesis of **13** were employed but with [(Ph_2_C_2_S_2_)Pd(tpbz)] (0.116 g, 0.0997 mmol) used in place of [(mnt)Ni(μ-tpbz)]. Compound **14** was purified on a silica column eluted with a 90:10 CH_2_Cl_2_/hexanes mixture and collected as a brown-red band. Crystallization was accomplished by diffusion of Et_2_O vapor into a CH_2_Cl_2_ solution. Yield: 0.080 g of brown solid, 53%. ^31^P{^1^H} NMR (δ, CDCl_3_): 46.65 (s), 28.51 (s). MS (ESI^+^) calcd for [C_71_H_52_BrO_3_P_4_PdReS_2_]^+^: *m*/*z* 1514.0144. Observed: *m*/*z* 1514.0112. Error (δ): 2.1 ppm.

### [(Ph_2_C_2_S_2_)Pt(μ-tpbz)Re(CO)_3_Br], **15**

The same procedure and scale as described for the synthesis of **13** were employed but with [(Ph_2_C_2_S_2_)Pt(tpbz)] (0.125 g, 0.0998 mmol) in place of [(mnt)Ni(tpbz)]. Compound **15** was purified on a silica column eluted with a 70:10 CH_2_Cl_2_/hexanes mixture and collected as a dark orange-yellow band. Crystallization was accomplished by the diffusion of Et_2_O vapor into a CH_2_Cl_2_ solution. Yield: 0.098 g of brown solid, 62%. ^31^P{^1^H} NMR (δ, CDCl_3_): 38.06 (s), 28.65 (s), 28.57 (s). MS (ESI^+^) calcd for [C_71_H_52_BrO_3_P_4_PtReS_2_]^+^: *m*/*z* 1602.0690. Observed: *m*/*z* 1602.0676. Error (δ): 0.82 ppm. Anal. Calcd for C_71_H_52_O_3_P_4_S_2_BrRePt: C, 53.22; H, 3.27. Found: C, 53.02; H, 3.29.

### [((NC)_2_C_2_S_2_)Ni(μ-tpbz)ReBr(CO)(μ-tpbz)Ni(S_2_C_2_(CN)_2_)], **16**, and [((NC)_2_C_2_S_2_)Ni(μ-tpbz)Re(CO)_2_(μ-tpbz)Ni(S_2_C_2_(CN)_2_)]Br, [**17**]Br

The same procedure and scale as described for the synthesis of **18** and [**19**]Br (*vide infra*) were employed but with [(mnt)Ni(tpbz)] used in place of [(Ph_2_C_2_S_2_)Pt(μ-tpbz)]. Following extraction of the crude solid with CH_2_Cl_2_ and filtering of the extract through a Celite pad, the filtrate was reduced to dryness under vacuum to afford a light red-orange residue. Diffusion of Et_2_O vapor into a 1,3-dimethyl-2-imidazolidinone solution of this crude solid produced orange plate crystals of **16**. After the crystals of **16** had been separated, the remaining crude material was reduced to dryness and further washed with Et_2_O. Crystallization by the same method that was used for **16** yielded [**17**]Br as orange-brown column-shaped crystals. Yield of **16**: 0.080 g, 68%. ^31^P{^1^H} NMR (δ, CD_2_Cl_2_): 58.08 (s), 28.71 (s). IR (KBr, cm^–1^): 2125 (asym *v*_C≡N_), 2156 (sym *v*_C≡N_). MS (ESI^+^) calcd for [C_117_H_84_BrN_4_Ni_2_OP_8_ReS_4_]^+^: *m*/*z* 2320.0861. Observed: *m*/*z* 2320.1266. Error (δ): 17.47 ppm. Yield of [**17**]Br: 0.023 g. ^31^P{^1^H} NMR (δ, CD_2_Cl_2_): 54.98 (s), 29.10 (s). MS (ESI^+^) calcd for [C_118_H_84_N_4_Ni_2_O_2_P_8_ReS_4_]^+^: *m*/*z* 2269.1639. Observed: *m*/*z* 2269.1619. Error (δ): 0.87 ppm.

### [(Ph_2_C_2_S_2_)Pt(tpbz)Re(CO)Br(μ-tpbz)Pt(S_2_C_2_Ph_2_)], **18**, and [(Ph_2_C_2_S_2_)Pt(μ-tpbz)Re(CO)_2_(μ-tpbz)Ph(S_2_C_2_Ph_2_)][Br], [**19**][Br]

Under an atmosphere of N_2_, a 50 mL Schlenk flask was charged with [(Ph_2_C_2_S_2_)Pt(μ-tpbz)] (0.125 g, 0.0998 mmol) and [Re(CO)_5_Br] (0.022 g, 0.054 mmol). Dry *cis*,*trans*-decalin (10 mL) was transferred to the reaction mixture via syringe. The reaction mixture was heated to reflux for 72 h. At an early stage (∼30 min), the reaction mixture turned a dark brown-red color, and solid material was observed around the wall of the flask. After the reaction mixture had cooled to ambient temperature, the solvent was removed by cannula filtration, and the solid residue was washed with hexanes (5 mL) and Et_2_O (2 × 5 mL) and then dried *in vacuo*. The crude product was partially dissolved in CH_2_Cl_2_ (10 mL) and filtered through a Celite pad. The filtrate was reduced to a volume of 3 mL whereupon 10 mL of hexanes was added via syringe to precipitate the product as an orange powder. The supernatant was removed by cannula filtration, and the solid residue was washed with Et_2_O (2 × 8 mL) and dried under vacuum. Mass spectrometric analysis (ESI) of the crude solid indicated the presence of both **18** and [**19**][Br]. Diffusion of Et_2_O vapor into a solution of this product mixture in nitrobenzene produced an initial crop of **18**·4(PhNO_2_) as orange plate crystals. Following the collection of **18**·4(PhNO_2_), the residual solid was purified on a silica column eluted with a 4:1 CH_2_Cl_2_/hexanes mixture and collected as a yellow-orange band. Orange column crystals of [**19**][Br] were grown by the same PhNO_2_/Et_2_O vapor diffusion method. Yield of **18**: 0.030 g of yellow brown solid, 22%. ^31^P{^1^H} NMR (δ, CDCl_3_): 41.92 (s), 28.31 (s). UV–vis [CHCl_3_, λ_max_, nm (ε)]: 434 (140). MS (ESI^+^) calcd for [C_137_H_104_BrOP_8_Pt_2_ReS_4_]^+^: *m*/*z* 2798.2917. Observed: 2798.2405. Error (δ): 18.28 ppm. Yield of [**19**][Br]: 0.018 g of yellow-brown solid. ^31^P{^1^H} NMR (δ, CDCl_3_): 42.03 (s, *J*_Pt–P_ = 2734 Hz), 28.47 (s). MS (ESI^+^) calcd for [C_138_H_104_O_2_P_8_Pt_2_ReS_4_]^+^: *m*/*z* 2746.3694. Observed: *m*/*z* 2746.3220. Error (δ): 17.28 ppm.

## Discussion

### Syntheses and Structures

Well-defined coordination compounds featuring two chelating diphosphine ligands have broad precedent among the group VI–XI elements. In general, when implemented using open-ended [(R_2_C_2_S_2_)M(tpbz)] complexes (M = Ni^2+^, Pd^2+^, or Pt^2+^; R = CN, Ph, or *p*-anisyl) as “ligands,” modifications of these procedures are effective in placing two such metallodithiolene diphosphine complexes around a third, central ion. Hexacationic [(phen)_2_Os(tpbz)Ni(tpbz)Pd(dppb)]^6+^ [dppb = 1,2-bis(diphenylphosphino)benzene], designed by Fox and Zahavy as a redox-gated, long-range photoelectron transfer device, is the only related trimetallic assembly thus far reported.^17^ This cation was not characterized structurally.

As its triflate salt, the tetrakis(propionitrile)platinum(II) cation provides clean substitution reactions in yields of 75–85% with [(R_2_C_2_S_2_)M(tpbz)], where the identity of R and M can be independently varied as CN, Ph, or *p*-anisyl and Ni^2+^, Pd^2+^, or Pt^2+^, respectively (Scheme 1). Once prepared, these cations are stable to air and moisture. Use of a Ni^2+^ or Pd^2+^ precursor in place of [Pt(NCEt)_4_]^2+^ for the central ion was less successful in providing tractable products. Although subject to some degree of static disorder in the crystalline state, the triflate anion (OTf^−^) repeatedly proved to be more amenable than anions such as PF_6_^–^ or [BArF_24_]^−^ for the formation of diffracting crystals.

**Figure 1:**
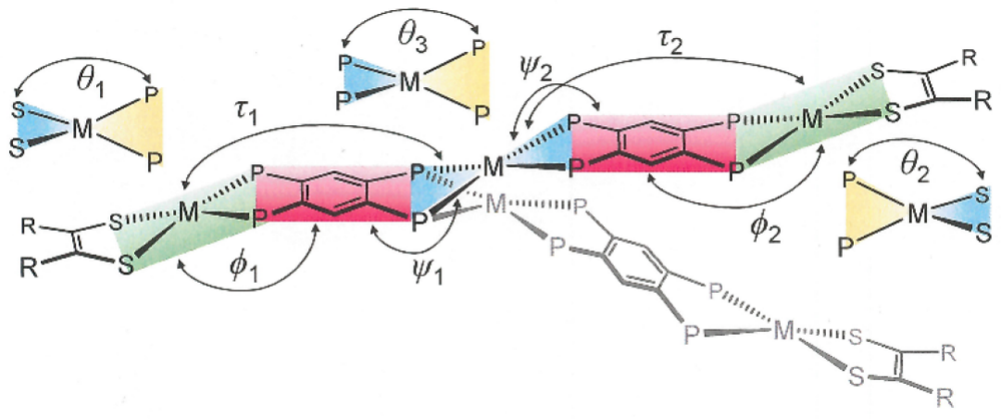
Graphical definitions of the interplanar angles that decide the core topology of the trimetallic, tpbz-linked assemblies.

The crystalline state structures of these trimetallic compounds composed of divalent group 10 ions are subject to two distinctive distortions that operate independently but with cumulative effects that can produce rather different core topologies. The first of these distortions is minor tetrahedralization at the square planar sites, which is quantified as the angle, θ, between the two planes defined by M with the donor atoms of each ligand chelate (Figure 1 and Table 2). The second deformation is folding of the tpbz ligand along an intrachelate P···P axis such that the MP_2_ plane is not coincident with the P_2_C_6_P_2_ mean plane. The folding along the P···P axis on the two different ends of each tpbz ligand, designated as φ and ψ (Figure 1), may bend the appended fragments to the same side, or to opposite sides, of the P_2_C_6_P_2_ mean plane, thereby conferring a “boat-like” or “chair-like” local geometry at the tpbz ligand. Analogous structural features with similar variation are seen in [(tdpttf)_2_M]^2+^ [tdpttf = tetrakis(diphenylphosphino)tetrathiafulvalene;^18^ M = Pd^2+^ or Pt^2+^], which, although monometallic species, are comparable in size because of the greater length of the tdpttf ligand.^19^

**Table 2:** Selected Angles (degrees) and Distances (angstroms) of All Structurally Characterized Compounds

		θ_1_^a^	θ_2_^b^	θ_3_^c^	ϕ_1_^d^	ϕ_2_^e^	ψ_1_^f^	ψ_2_^g^	τ_1_^h^	τ_2_^i^	tpbz conformation	*d*^j^
M–Pt–M compounds	[**1**]^2+^	1.1	–k	0.0	33.3	–k	28.3	–k	61.6	–k	boat	22.38
	[**3**]^2+^	16.6	–k	0.0	19.4	–k	18.8	–k	7.1	–k	chair	23.58
	[**4**]^2+^	13.3	–k	0.0	19.7	–k	17.9	–k	5.9	–k	chair	23.95
	[**5**]^2+^	13.3	–k	0.0	19.7	–k	18.2	–k	5.7	–k	chair	23.95
M–Au–M compounds	[**6**]^+^^l^	6.5	–k	83.4	5.5	–k	3.9	–k	3.0	–k	–	23.77
		10.4		87.1	8.8		3.5		6.6			23.66
	[**7**]^+^	13.0	9.2	87.5	8.3	13.1	9.2	4.9	5.3	8.2	–	23.72
	[**10**]^+^	8.1^m^	5.0	83.9	16.0^m^	32.7	13.8^m^	11.8	4.6	21.5	–	23.25
	[**11**]^+^	5.7	1.1	86.3	18.4	21.1	12.2	19.3	8.9	5.5	–	23.86
M–Re compounds	**13**^n^	2.8	–	–	18.5	–	50.6	–	32.1	–	chair	–
	**13**^o^	1.6^p^	–	–	19.9^p^	–	50.8	–	31.4^p^	–	chair	–
	**14**	6.1	–	–	12.4	–	25.1	–	37.5	–	boat	–
	**15**	5.1	–	–	13.1	–	25.3	–	38.4	–	boat	–
M–Re–M compounds	**16**	3.5	–k	0.0	18.1	–k	21.6	–k	39.6	–k	boat	23.46
	[**17**]^+^	8.9	–k	0.0	21.2	–k	34.9	–k	55.6	–k	boat	22.89
	**18**	4.7	–k	0.0	22.5	–k	25.4	–k	4.2	–k	chair	23.97
	[**19**]^+^	2.0	–k	0.0	23.9	–k	22.6	–k	3.3	–k	chair	24.02

a θ_1_ is the angle between the S_2_M and P_2_M planes, left side.

b θ_2_ is the angle between the S_2_M(2) and P_2_M(2) planes, right side.

c θ_3_ is the angle between the P_2_M planes at the central ion.

d ϕ_1_ is the angle between the S_2_MP_2_ (left side) and P_2_C_6_P_2_ (left side) mean planes.

e ϕ_2_ is the angle between the S_2_MP_2_ (right side) and P_2_C_6_P_2_ (right side) mean planes.

f ψ_1_ is the angle between the P_2_C_6_P_2_ mean plane (left side) and P_2_M plane (central ion or Re).

g ψ_2_ is the angle between the P_2_C_6_P_2_ (right side) and P_2_M plane (central ion).

h τ_1_ is the angle between S_2_MP_2_ (left side) and P_2_M (central ion).

i τ_2_ is the angle between S_2_MP_2_ (right side) and P_2_M (central ion).

j *d* is the distance between the centroids of the dithiolene C=C bonds.

k The right half of the compound is symmetry-related to the left half.

l Two independent half cations of [**6**]^+^ occur in the asymmetric unit.

m One P atom of the tpbz ligand is disordered over two sites. The position giving the smaller distortion from square planarity is used to evaluate the angle.

n Pseudopolymorph 1, data set JPD979.

o Pseudopolymorph 2, data set JPD1046.

p These values were determined using the positional variant of the disordered mnt(2−) ligand with a higher site occupancy.

**Figure 2:**
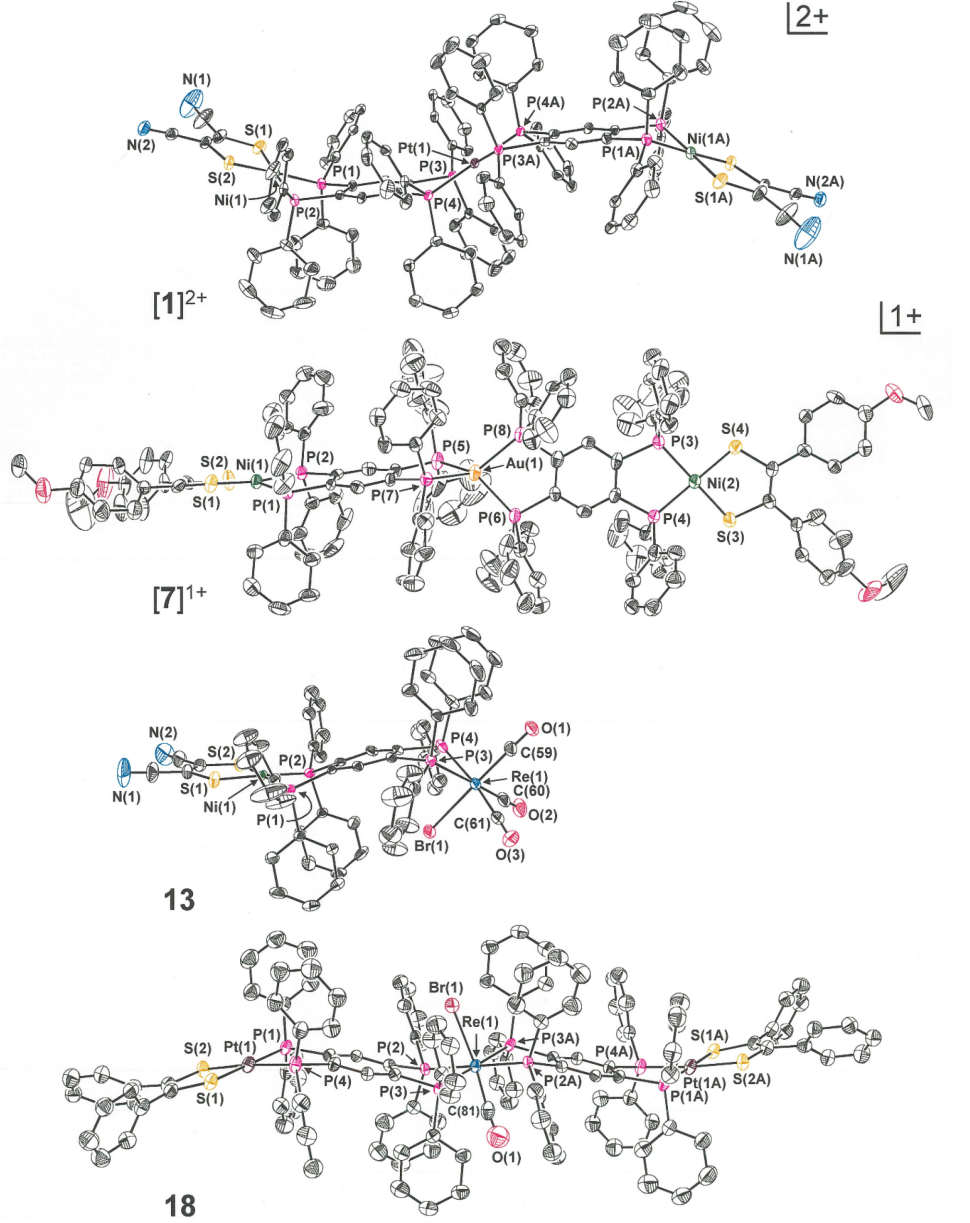
Thermal ellipsoid plots of [**1**]^2+^ (50%), [**7**]^+^ (50%), **13** (40%), and **18** (35%). For the sake of maximum clarity, all H atoms, counteranions, and interstitial solvent molecules have been omitted and disordered phenyl groups have been edited to present only one positional variant.

In [**1**]^2+^, while the local coordination geometry at each group 10 M^2+^ ion is square planar, the tpbz ligand displays a rotation about the intrachelate P(1)···P(2) axis such that the S_2_NiP_2_ mean plane presents an angle of 33.3° (φ_1_) to the P_2_C_6_P_2_ mean plane (Figure 2 and Table 1). A similar fold in the same direction between the P_2_C_6_P_2_ mean plane and the P_2_Pt plane disposes them at an angle of 28.3° (ψ_1_) such that the S_2_NiP_2_ and P_2_Pt planes meet at 61.6° (τ_1_), and an accentuated concave shape results for each half of [**1**]^2+^. The dication occurs on a crystallographic inversion center that is coincident with Pt(1) and thus causes the cation to undulate with a pronounced S-shaped core topology. The OTf^–^ counteranions are ensconced within the cavities defined by this particular shape (Figure S7), suggesting that the exaggerated conformation displayed by [**1**]^2+^ arises from felicitous charge-pairing/ion-packing effects for the crystalline state. The distance between the centroids of the dithiolene C=C bonds in [**1**]^2+^ is 22.38 Å.

**Figure 3:**
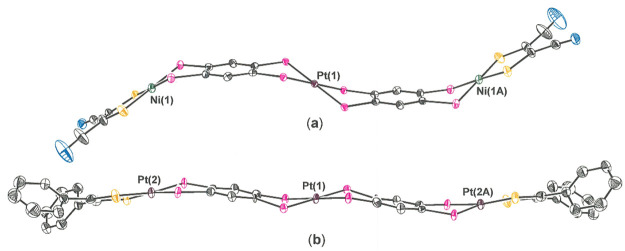
Core topologies found in [**1**]^2+^ and [**5**]^2+^, which are illustrative of the “S-like” and “herringbone-like” conformations, respectively. Ellipsoids are shown at the 50% level.

The series [(Ph_2_C_2_S_2_)M(μ-tpbz)Pt(μ-tpbz)M(S_2_C_2_Ph_2_)]^2+^ (M = Ni^2+^, Pd^2+^, or Pt^2+^) was prepared in a fashion analogous to that of [**1**]^2+^ and similarly crystallized by diffusion of Et_2_O vapor into solutions composed with a strongly polar organic solvent. While similar to [**1**]^2+^ in crystallizing upon an inversion center, [**3**]^2+^–[**5**]^2+^ contrast with [**1**]^2+^ by assuming the “chair” conformation at the tpbz ligands, rather than the “boat” conformation, such that their geometry has a staircase-like or herringbone-like appearance (Figure 3). Possibly because of the greater steric profile projected by the [Ph_2_C_2_S_2_]^2–^ ligands, the OTf^–^ anions are not as closely associated with these dications as in [**1**]^2+^.

Introduction of [Cu(MeCN)_4_][PF_6_] or [Ag(MeCN)_4_][BF_4_] to [(Ph_2_C_2_S_2_)Ni(tpbz)] in a 1:2 ratio induced an immediate reaction, as marked by a distinctive darkening in color. Formation of the intended trimetallic monocations [**8**]^+^ and [**9**]^+^ was readily corroborated by ^31^P NMR spectroscopy and mass spectrometry (*vide infra*). However, despite considerable effort, these compounds were not amenable to the formation of crystals suited for X-ray diffraction because of an apparent limited solution stability.

Treatment of [AuCl(PPh_3_)] with [(R_2_C_2_S_2_)M(tpbz)] in a 1:2 ratio led to the immediate formation of the trimetallic assembly, again as manifested by a change in the solution color. Possibly because of a greater M′–P_phosphine_ bond dissociation energy for the heavier metal, the trimetallic constructs with Au^+^ at the nexus ([**6**]^+^, [**7**]^+^, and [**10**]^+^–[**12**]^+^) were decisively more tractable to crystallization. As with [**1**]^2+^ and [**3**]^2+^–[**5**]^2+^, triflate proved to be the anion most conducive to formation of X-ray diffraction quality crystals, although for [**7**]^+^, chloride that presumably originated from [AuCl(PPh_3_)] was the counteranion identified by crystallography. Four of the trimetallic M–Au–M monocations have been structurally characterized (Tables 1 and 2), the principal difference between them and the M–Pt–M set being the orthogonal disposition of the [(R_2_C_2_S_2_)M(tpbz)] end groups that is enforced by the d^10^ Au^+^ ion with its preference for the tetrahedral geometry. The incompatibility of this geometry with a crystallographic inversion center brought most of these cations onto a general position, but [**6**]^+^ notably occurred on a crystallographic *C*_2_ axis in *C*2 (No. 5). When the conformations of peripheral substituents and the twist (θ) and fold angles (φ and ψ) are neglected, the idealized point group symmetry in these M–Au–M compounds is *D*_2*d*_.

The structure of [**7**]^+^, which is representative of the M–Au–M set in general respects, is presented in Figure 2. A modest 8.3° angle defines the juncture between the S_2_NiP_2_ and P_2_C_6_P_2_ mean planes at its left half (φ_1_), but its effect is offset by a 9.2° fold of the P(5)–Au(1)–P(7) plane in the opposite direction (downward) across the P(5)···P(7) axis with the result that a slight chair conformation to the left-side tpbz ligand results. A qualitatively similar description is pertinent to the right half of [**7**]^+^, as presented in Figure 2. The slight distortion from *D*_2*d*_ local symmetry at Au(1), as expressed by the modest departure from orthogonality between the P(5)–Au(1)–P(7) and P(6)–Au(1)–P(8) planes by 2.5°, is similar in magnitude to distortions from ideal *D*_2*d*_ symmetry observed in a variety of mononuclear [Au(dppb)_2_]^+^ complexes.^20−22^ This minor distortion therefore appears to be unrelated to the size of [**7**]^+^ and any packing forces to which it is subject.

Bis(diphosphine) complexes of Re(I) can be directly accessed from [ReX(CO)_5_] (X = Cl or Br) but demand forcing conditions, such as refluxing for 7 days in mesitylene (∼165 °C). With dppb itself, the organic analogue of open-ended [(R_2_C_2_S_2_)M(tpbz)] complexes, these conditions are reported to produce a *cis*/*trans* mixture of [Re(CO)_2_(dppb)_2_]^+^, as deduced from spectroscopic data.^23^ However, when used as the medium for reaction between [(R_2_C_2_S_2_)M(tpbz)] and [ReBr(CO)_5_], mesitylene enables only the formation of [(R_2_C_2_S_2_)M(μ-tpbz)ReBr(CO)_3_], several examples of which (**13–15**) have been isolated and structurally identified (Figure 2 and Table 2). Compound **13** reveals a fold angle (ψ_1_) between the P_2_C_6_P_2_ and P_2_Re planes that is twice the magnitude (∼51°) of the corresponding value in **14** and **15** (∼25°). A further difference is that **13** holds a chair conformation about its tpbz bridge such that τ_1_ (∼31°) is essentially the difference between ψ_1_ and φ_1_ (∼20°), while a boat conformation at the tpbz bridge in **14** and **15** causes φ_1_ (∼13°) and ψ_1_ to be additive in producing τ_1_ (∼38°). Similarly sharp fold angles observed between the tpbz bridge and M(CO)_4_ (when M = Mo, ψ_ave_ = 20.3°; when M = W, ψ = 21.3°)^24^ and ReBr(CO)_3_ (ψ_ave_ = 32.9°)^25^ end groups in symmetric dimetallic compounds suggest that this structural feature is a lower-energy configuration arising from enhanced M → CO π overlap at the expense of the M–tpbz interaction.

The somewhat more elevated reflux temperature offered by decalin (∼190 °C), when used as the medium for reactions of [(R_2_C_2_S_2_)M(tpbz)] with 0.5 equiv of [ReBr(CO)_5_], leads to separable mixtures of [(R_2_C_2_S_2_)M(tpbz)ReBr(CO)(tpbz)M(S_2_C_2_R_2_)] and [(R_2_C_2_S_2_)M(tpbz)Re(CO)_2_(tpbz)M(S_2_C_2_R_2_)]Br in a time frame of 72 h (Scheme 1). The thermal stability demonstrated by these compounds under such forcing conditions is striking. Successful preparation and isolation, including identification by X-ray crystallography, of two representative sets (for **16** and [**17**]Br, R = CN and M = Ni^2+^; for **18** and [**19**]Br, R = Ph and M = Pt) support the presumption that other permutations of the identity of R and M would be similarly effective. All four compounds crystallize upon inversion centers in monoclinic space group 14 (*P*2_1_/*c*, *P*2_1_/*n*), a situation that imposes disorder between the *trans*-disposed CO and Br^–^ ligands in **16** and **18**. In **18**, despite the disorder, the two ligands were clearly distinguishable in the electron density map and amenable to refinement with minimal restraints. The tpbz conformations within both **18** and [**19**]^+^ are chairlike, with τ_1_ essentially being the difference between ψ_1_ and φ_1_, which produces a core topology similar to that illustrated for [**5**]^2+^ in Figure 3. The opposite situation pertains to **16** and [**17**]^+^, which both have the boat conformations at the tpbz bridges and an overall S-like core topology similar to that of [**1**]^2+^ (Figure 3) but less pronounced.

**Figure 4:**
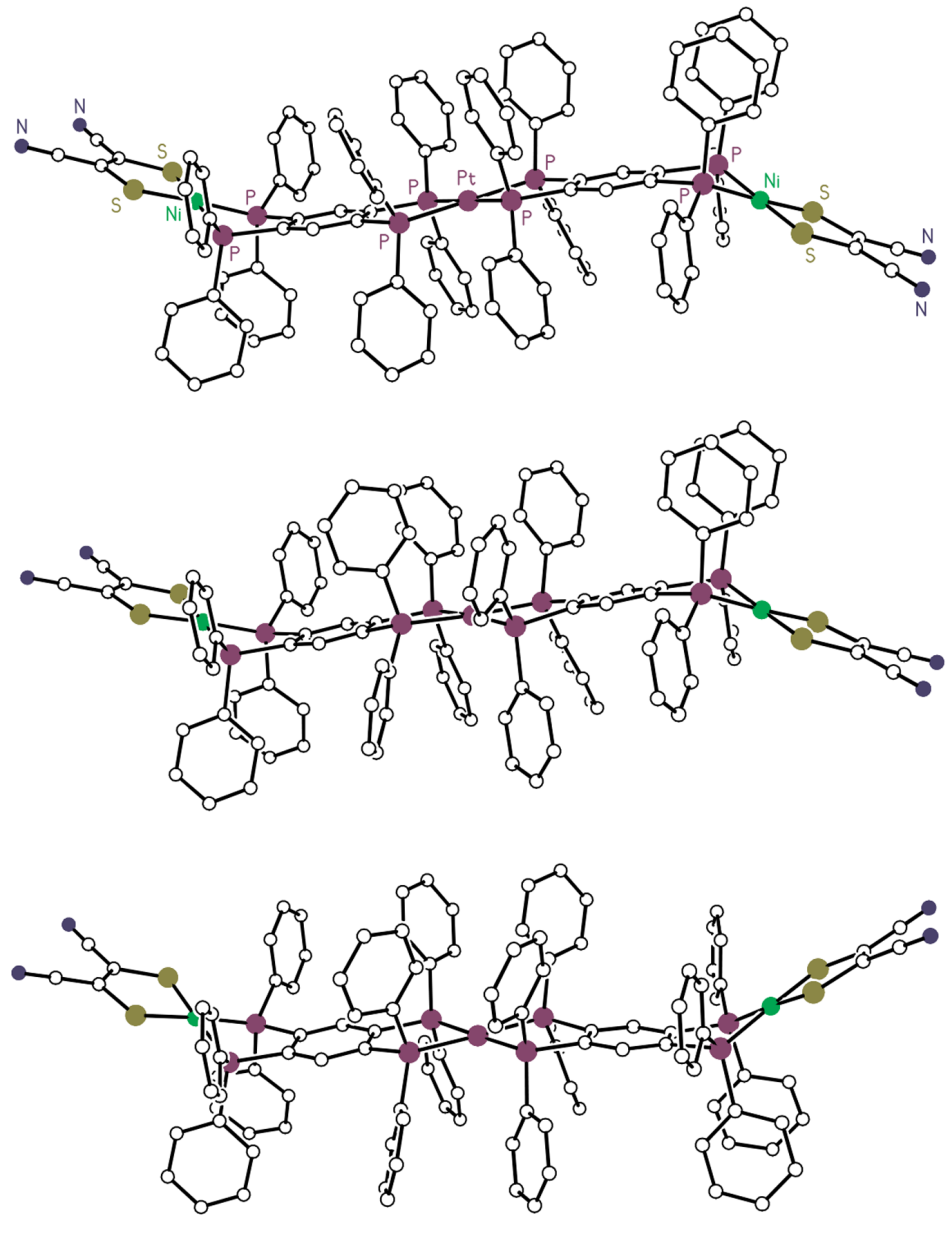
B3PW91/LANL2DZ-calculated low-energy conformations of compound [**1**]^2+^. Hydrogen atoms have been omitted for the sake of clarity. *C*_*i*_-[**1**]^2+^, *C*_1_-[**1**]^2+^, and *C*_2_-[**1**]^2+^ from top to bottom, respectively.

The range of structural variation among these sets of trimetallic compounds implies conformational complexity with only modest differences in the energies of the various geometries. A casual inspection of chemical drawings of these compounds suggests that complexes [**1**]^2+^ and [**5**]^2+^ possess low-energy conformations of *D*_2*h*_ symmetry, but such structures exhibit severe steric conflicts between the many phenyl substituents. A computational exploration of the energy landscape for [**1**]^2+^ at the B3PW91/LANL2DZ and B3PW91/Def2SVP levels of theory^26−29^ finds that *D*_2*h*_-[**1**]^2+^ is a high-order saddle point, more than 50 kcal/mol above any potential minimum (Table 3). Lowering the symmetry to *D*_2_ relieves most of the strain, but even this structure is a transition state 3–7 kcal/mol higher in energy than various lower-symmetry structures. Low-energy potential minima of *C*_*i*_, *C*_2_, and *C*_1_ symmetry were located (Figure 4), with the *C*_1_ structure being the ground state by a fraction of a kilocalorie per mole. The B3PW91/LANL2DZ-calculated *C*_*i*_-symmetric conformation closely resembles the experimental molecular structure, but the small range of energy differences (∼2 kcal/mol) between the local minima, and the likely low barriers between these conformations, suggest a dynamic solution behavior in which the trimetallic core of the molecule fluctuates between broad S-shaped and U-shaped conformations.

**Table 3:**
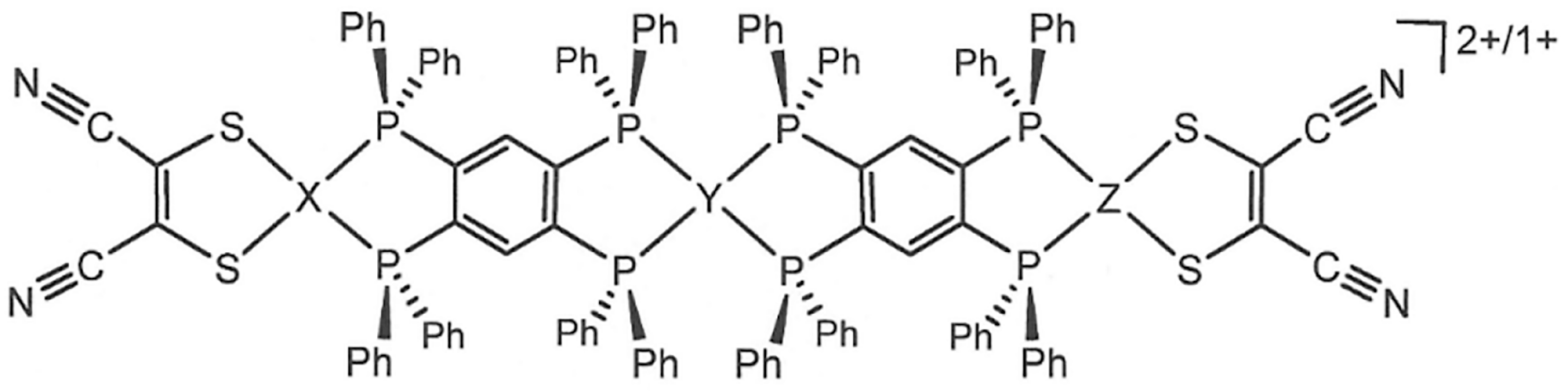
Calculated Energies of the Conformations of Selected Trimetallic Complexes

compound	symmetry	core shape^a^	*E* (au)^b^	Δ*E* (kcal/mol)	*E*+ZPE (au)^b^	Δ(*E*+ZPE) (kcal/mol)	*n*I^c^	*d*_*xz*_ (Å)
At the B3PW91/LANL2DZ Level								
X = Z = Ni^2+^; Y = Pt^2+^	*D*_2*h*_	F	–5237.507053	+60.0	–5235.828275	+58.2	11	18.28
	*D*_2_	F	–5237.595433	+4.5	–5235.913860	+4.5	1	18.24
	*C*_*i*_ (∼*C*_2*h*_)	S	–5237.600267	+1.5	–5235.918662	+1.5	0	17.90
	*C*_1_ (∼*C*_2_)	S	–5237.602645	0.0	–5235.921097	0.0	0	17.94
	*C*_2_	U	–5237.602268	+0.2	–5235.920752	+0.2	0	17.88
X = Z = Ni^2+^; Y = Au^+^	*D*_2_	T	–5254.128435	+5.1	–5252.450013	+5.2	0	18.44
	*C*_1_ (∼*C*_2_)	T	–5254.130774	+3.6	–5252.452486	+3.7	0	18.21
	*C*_2_	T	–5254.136544	0.0	–5252.458358	0.0	0	17.70
At the B3PW91/def2SVP Level								
X = Z = Ni^2+^; Y = Pt^2+^	*D*_2*h*_	F	–12142.070835	+65.7	–12140.405196	+64.3	12	17.98
	*D*_2_	F	–12142.164330	+7.1	–12140.496690	+6.9	1	17.91
	*C*_*i*_ (∼*C*_2*h*_)	S	–12142.172096	+2.2	–12140.504095	+2.3	0	17.58
	*C*_1_	S	–12142.175578	0.0	–12140.507703	0.0	0	17.50
	*C*_2_	U	–12142.174724	+0.5	–12140.507090	+0.4	0	17.53

a Legend: F, flat; S, S-shaped; U, U-shaped; T, tetrahedral center.

b 1 au = 627.503 kcal/mol.

c *n*I is the number of imaginary frequencies.

When the central platinum is replaced with tetrahedral gold, the situation is less complex. At the B3PW91/LANL2DZ level, three low-energy conformations were found, with the apparent *C*_2_ ground state resembling the experimental structure of [**6**]^+^, although the molecule in the crystal lies at a general position and thus does not have the strict *C*_2_ symmetry of the calculated minimum. The calculated *D*_2_ and *C*_1_ minima are significantly higher in energy (5.2 and 3.7 kcal/mol, respectively), and it is likely that fewer than 1 in 100 molecules adopt these conformations in solution.

### Spectroscopy

Clean formation of the intended trimetallic arrays was affirmed unequivocally in their ^31^P NMR spectra, which revealed a substantial downfield shift of the signal attributed to the open phosphines in [(R_2_C_2_S_2_)M(η^2^-tpbz)] to positions that depended upon the identity of the central metal ion: approximately +55 ppm for central Pt^2+^, approximately +35 ppm for Au^+^, and approximately +43 ppm for Re^+^ (Table 4). The Cu^+^- and Ag^+^-linked compounds revealed peak positions and, in the case of Ag^+^, coupling constants very similar to those of the analogous mononuclear [(dppb)_2_M]^+^ (M = Cu^+^^22,30,31^ or Ag^+^^32−34^) cations (cf. Figures S85 and S91). The more downfield-shifted ^31^P signal in [**8**]^+^, which is due to the phosphines that are chelating Ni^2+^, has the sharp character typical of most of these complexes (Figure S85). The ^31^P NMR signal for the phosphines chelating to Cu^+^ in [**8**]^+^, however, is broad to a degree that is not observed for [(P-P)_2_Cu]^+^ complexes until temperatures of ≤190 K are imposed. This effect may be due to hindered fluxionality about Cu^+^ arising from the appreciably greater size of the [(Ph_2_C_2_S_2_)Ni(tpbz)] “ligands” compared to the smaller, purely organic diphosphines. In all cases, the phosphine signal attributable to tpbz bound to the metallo-dithiolene end groups occurred downfield of the phosphine signal arising from the tpbz end chelating the central ion.

**Table 4:** Summary of ^31^P NMR Data for tpbz Compounds

compound	^31^P NMR signals (solvent)
[(mnt)Ni(tpbz)]	57.2, – 4.5^a^ (CDCl_3_)
[(adt)Ni(tpbz)]	55.2, −14.2^a^ (CDCl_3_)
[(pdt)Ni(tpbz)]	55.0, −14.8^a^ (CDCl_3_)
[(pdt)Pd(tpbz)]	49.4, −14.7^a^ (CDCl_3_)
[(pdt)Pt(tpbz)]	42.7, −14.7^a^ (CDCl_3_)
[(mnt)Ni(tpbz)Pt(tpbz)Ni(mnt)]^2+^, [**1**]^2+^	59.14, 42.40^b^ (DMSO-*d*_6_)
[(adt)Ni(tpbz)Pt(tpbz)Ni(adt)]^2+^, [**2**]^2+^	54.59, 41.03^b^ (DMSO-*d*_6_)
[(pdt)Ni(tpbz)Pt(tpbz)Ni(pdt)]^2+^, [**3**]^2+^	55.87, 41.05^b^ (DMSO-*d*_6_)
[(pdt)Pd(tpbz)Pt(tpbz)Pd(pdt)]^2+^, [**4**]^2+^	48.92, 40.75^b^ (DMSO-*d*_6_)
[(pdt)Pt(tpbz)Pt(tpbz)Pt(pdt)]^2+^, [**5**]^2+^	43.69, 40.50^b^ (DMSO-*d*_6_)
[(dppb)_2_Cu]^+^	8.12 (CDCl_3_)^c^
[(dppb)_2_Ag]^+^	0.28 (CD_2_Cl_2_)^c^
	(d, *J*_^31^P-^107^Ag_ = 230 Hz)
	(d, *J*_^31^P-^107^Ag_ = 265 Hz)
[(dppb)_2_Au]^+^	21.43 (CD_2_Cl_2_)^c^
[(mnt)Ni(tpbz)Au(tpbz)Ni(mnt)]^+^, [**6**]^+^	56.30, 21.21^b^ (CD_2_Cl_2_)
[(adt)Ni(tpbz)Au(tpbz)Ni(adt)]^+^, [**7**]^+^	53.76, 20.13^b^ (CD_2_Cl_2_)
[(pdt)Ni(tpbz)Cu(tpbz)Ni(pdt)]^+^, [**8**]^+^	54.46, 7.46^b^ (br) (CD_2_Cl_2_)
[(pdt)Ni(tpbz)Ag(tpbz)Ni(pdt)]^+^, [**9**]^+^	54.74 (CD_2_Cl_2_)
	–0.68^b^ (d, *J*_^31^P-^107^Ag_ = 234 Hz)
	–0.69^b^ (d, *J*_^31^P-^109^Ag_ = 262 Hz)
[(pdt)Ni(tpbz)Au(tpbz)Ni(pdt)]^+^, [**10**]^+^	54.15, 20.57^b^ (CD_2_Cl_2_)
[(pdt)Pd(tpbz)Au(tpbz)Pd(pdt)]^+^, [**11**]^+^	47.59, 20.48^b^ (CD_2_Cl_2_)
[(pdt)Pt(tpbz)Au(tpbz)Pt(pdt)]^+^, [**12**]^+^	42.61, 20.48^b^ (CD_2_Cl_2_)
[(mnt)Ni(tpbz)ReBr(CO)_3_], **13**	54.81, 28.91^d^ (CDCl_3_)
[(pdt)Pd(tpbz)ReBr(CO)_3_], **14**	46.65, 28.51^d^ (CDCl_3_)
[(pdt)Pt(tpbz)ReBr(CO)_3_], **15**	38.06, 28.65, 28.57^d^,^e^ (CDCl_3_)
[(mnt)Ni(tpbz)ReBr(CO)(tpbz)Ni(mnt)], **16**	58.08, 28.71^b^ (CD_2_Cl_2_)
[(mnt)Ni(tpbz)Re(CO)_2_(tpbz)Ni(mnt)]^+^, [**17**]^+^	54.08, 29.10^b^ (CD_2_Cl_2_)
[(pdt)Pt(tpbz)ReBr(CO)(tpbz)Pt(pdt)], **18**	41.92, 28.31^b^ (CDCl_3_)
[(pdt)Pt(tpbz)Re(CO)_2_(tpbz)Pt(pdt)]^+^, [**19**]^+^	42.03, 28.47^b^ (CDCl_3_)

a Signal due to the open end of tpbz.

b Signal arising from the end of tpbz coordinated to the central ion.

c From ref (22).

d Signal assigned to the end of tpbz coordinated to Re(I).

e The slight splitting in the signal for the Re-bound end is presumed to arise from the presence of both ^185^Re (*I* = ^5^/_2_, 37%) and ^187^Re (*I* = ^5^/_2_, 63%).

Mass spectrometry (ESI^+^) unequivocally identifies the trimetallic compounds with their parent masses and their distinctive isotope distribution profiles. Fragment masses arising from the trimetallic assembly {e.g., [(Ph_2_C_2_S_2_)Ni(tpbz)Cu]^+^ from [(Ph_2_C_2_S_2_)Ni(tpbz)Cu(tpbz)Ni(S_2_C_2_Ph_2_)]^+^ (Figure S90)} are, in some instances, observable. Generally darker colors mark the formation of the trimetallic arrays from their monometallic fragments; where absorption maxima are discernible and shoulders estimable, they are indicated (*vide supra*). The electronic absorption spectra of the trimetallic compounds generally show shifts to higher energy for the assemblies comprised of third-row metals compared to the analogues with lighter metals of the same group. In many instances, multiple broad unresolved bands that absorb across the visible spectrum complicate simple comparisons and interpretation. Rigorous spectral deconvolution and assignment of these transitions with the aid of time-dependent density functional theory calculations have not been attempted here.

**Figure 5:**
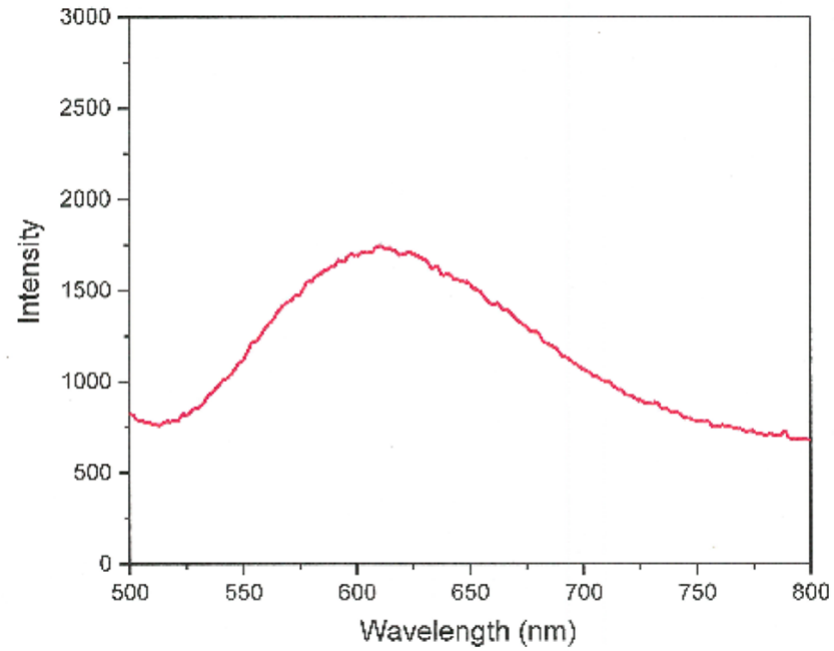
Emission spectrum of [(Ph_2_C_2_S_2_)Pt(tpbz)Au(tpbz)Pt(S_2_C_2_Ph_2_)]^+^ following 401 nm excitation (77 K, 4:1 EtOH/MeOH).

When probed with 325–355 nm light, [(dppb)_2_Au]^+^ is very brightly luminescent in the solid state with λ_em_ values that are highly dependent on the presence or absence of an interstitial solvent and the nature and/or identity of the counteranion.^20−22,35^ However, its trimetallic homologue, [(pdt)Ni(tpbz)Au(tpbz)Ni(pdt)]^+^, shows no emission under the same conditions. We attribute this observation to intramolecular energy transfer to states localized on the (phosphine)_2_Ni(dithiolene) fragments, which are likely lower in energy than the emitting state of the Au center. However, the isostructural [(Ph_2_C_2_S_2_)Pt(tpbz)Au(tpbz)Pt(S_2_C_2_Ph_2_)]^+^ cation displays a relatively long-lived emission of ∼5 μs at ∼610 nm in a frozen 4:1 EtOH/MeOH solution (Figure 5). This emission likely arises from the (phosphine)_2_Pt(dithiolene) end groups, as mononuclear complexes of this general type are known to be luminescent.^36,37^ The solid state, low-temperature matrix luminescence of these monoplatinum complexes is assigned as arising from a Pt (d π) to dithiolene (π*) transition, with the phosphine ligands influencing the relative energy of the Pt d π levels.^37^ A similar assignment should apply to [(Ph_2_C_2_S_2_)Pt(tpbz)Au(tpbz)Pt(S_2_C_2_Ph_2_)]^+^ because the dithiolene ligand can serve as an acceptor.

**Figure 6:**
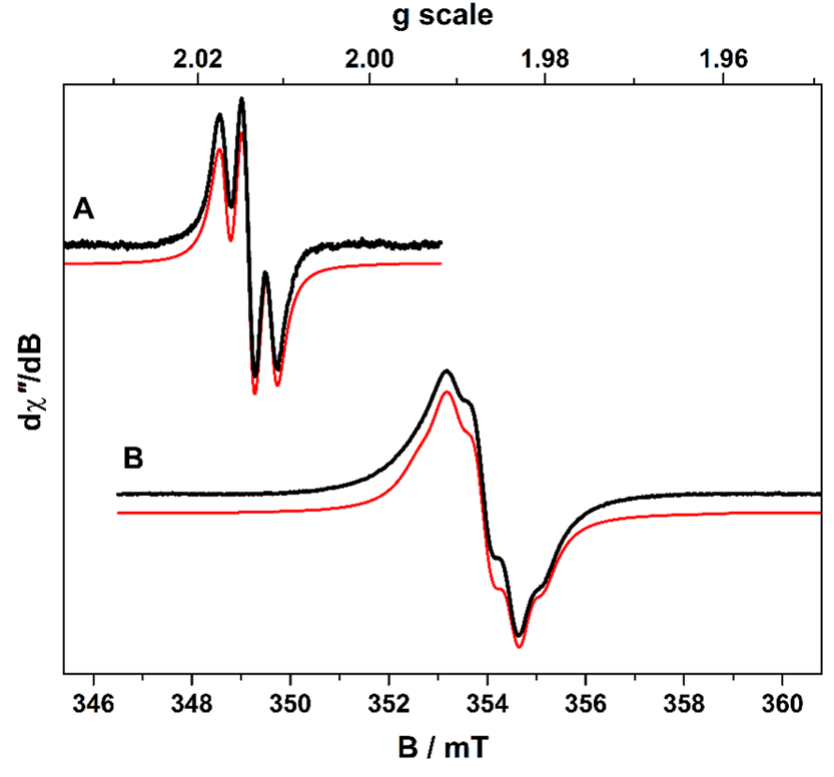
Comparison of the X-band EPR spectra of [**8**]^3+^ (**A**) and [**12**]^3+^ (**B**) recorded in a CH_2_Cl_2_ solution at 293 K. Experimental data are shown by the black lines, and simulations are depicted by the dashed red traces. Spin Hamiltonian parameters are given in the text.

The application of these multimetal assemblies as prototype two-qubit gates was investigated using EPR spectroscopy. Samples of [**8**]^+^ and [**12**]^+^ were oxidized *in situ* converting each terminal dithiolene ligand into its monoanionic radical form. This action generates tricationic spin-triplet species, whose spectra recorded at ambient temperature provide a measure of the exchange interaction between the ligand radicals. Most notable is the absence of exchange coupling, as the spectra are indicative of uncoupled *S* = ^1^/_2_ entities. The three-line spectrum of [**8**]^3+^ was simulated with *g* = 2.0131 and *A*_P_ = 4.5 × 10^–4^ cm^–1^ for coupling of the dithiolene radical with the two ^31^P (*I* = ^1^/_2_, 100%) nuclei of the μ-tpbz ligand (Figure 6). These parameters are identical to those of [(dppb)Ni(S_2_C_2_Ph_2_)]^+^ [dppb = 1,2-bis(diphenylphosphino)benzene].^7^ The absence of additional hyperfine lines demonstrates *J* ≈ 0 because of the sizable interspin distance exceeding 20 Å and the orthogonal orientation of each dithiolene spin center caused by the Cu(I) tetrahedral node (*vide supra*). The spectrum of [**12**]^3+^ gave a similar result with isolated *S* = ^1^/_2_ spin centers localized to the terminal dithiolene ligands with negligible exchange coupling. Simulation gave *g* = 1.9911 and includes additional ^195^Pt (*I* = ^1^/_2_, 33.4%) coupling of *A*_Pt_ = 12.0 × 10^–4^ cm^–1^ commensurate with a coordinated dithiolene radical (Figure S109).^7^

### Electrochemistry

**Figure 7:**
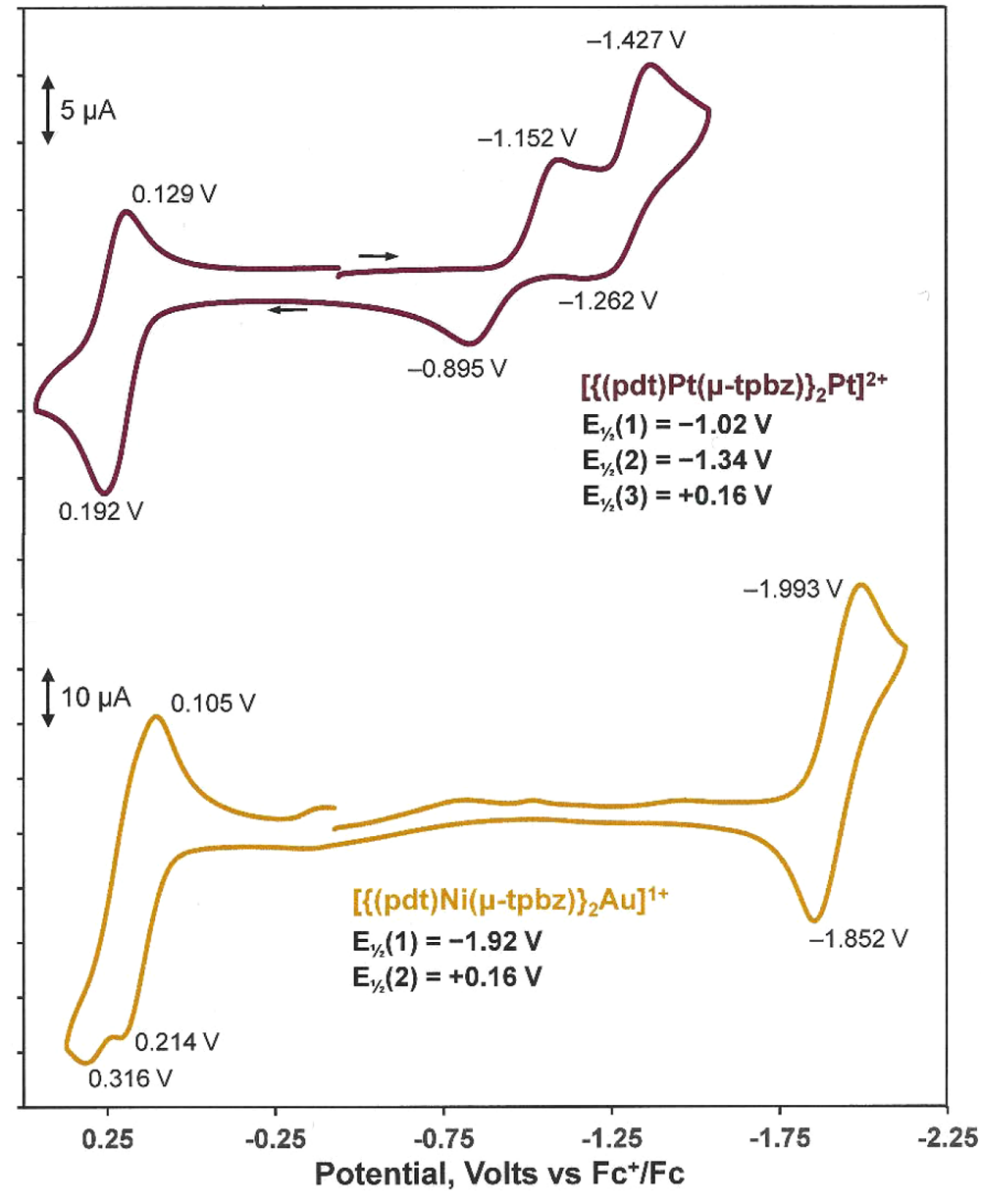
Cyclic voltammograms of [**5**]^2+^ in DMF (top) and [**10**]^+^ in CH_2_Cl_2_ (bottom) with [Et_4_N][PF_6_] as the supporting electrolyte and a Pt disk as the working electrode.

When R = alkyl or aryl, dimetallic compounds of the type [(R_2_C_2_S_2_)M(tpbz)M(S_2_C_2_R_2_)] support reversible, concurrent oxidations of the terminal dithiolene ligands ([R_2_C_2_S_2_]^2–^ – e^–^ → [R_2_C_2_S^–^S^•^]^−^) at ∼0 V versus Fc^+^/Fc. In the cathodic direction, at approximately −2.0 V versus Fc^+^/Fc, reversible reduction of the tpbz ligand is found but at half the current amplitude.^6^ The electrochemistry of [**5**]^2+^ differs from that of its dimetallic analogue [(Ph_2_C_2_S_2_)Pt(tpbz)Pt(S_2_C_2_Ph_2_)] in that it shows an anodically shifted quasireversible oxidation at approximately +0.16 V and two reduction processes, the first of which reveals a distinctive offset between its peak maxima (Figure 7, top, and Table 5). The anodic process arises from dithiolene ligand oxidation, as corroborated by geometry optimization and inspection of its frontier MOs (Figure S49). Its positive shift relative to [(Ph_2_C_2_S_2_)Pt(tpbz)Pt(S_2_C_2_Ph_2_)] arises from its dipositive charge. The LUMO for [**5**]^2+^ is the σ* combination of the central Pt d_*x*^2^–*y*^2^_ orbital with the phosphine lone pairs. Reduction to Pt^+^ induces a change in geometry from square planar to tetrahedral, which is affirmed computationally and accounts for the quasireversible nature of this feature. Subsequent reduction to Pt^0^, a redox level with ample precedent in a tetraphosphine environment,^38−41^ is reversible because the geometry is maintained at that point.

**Table 5:** Cyclic Voltametric Data (volts vs Fc^+^/Fc) for Selected Compounds

	solvent	oxidation	reductions	
[(mnt)Ni(tpbz)Pt(tpbz)Ni(mnt)]^2+^	DMF	–	–1.62 (Pt^2+^ → Pt^0^)	
[(pdt)Pt(tpbz)Pt(tpbz)Pt(pdt)]^2+^	DMF	+0.16	–1.02^a^ (Pt^2+^ → Pt^+^)	–1.34 (Pt^+^ → Pt^0^)
[(mnt)Ni(tpbz)Au(tpbz)Ni(mnt)]^+^	CH_2_Cl_2_	–	–1.40	
[(pdt)Ni(tpbz)Cu(tpbz)Ni(pdt)]^+^	CH_2_Cl_2_	+0.11	–1.46 (irr)	–1.63
[(pdt)Ni(tpbz)Au(tpbz)Ni(pdt)]^+^	CH_2_Cl_2_	+0.16	–1.92 (tpbz)	

a Attended by a change in geometry from square planar to tetrahedral.

In contrast to [**5**]^2+^, cyclic voltammetry of [**1**]^2+^ reveals no reversible oxidation because the highly electron-withdrawing nature of the nitrile groups of the mnt(2−) ligand renders it unable to sustain the [R_2_C_2_S_2_]^2–^ – e^–^ → [R_2_C_2_S^–^S^•^]^−^ oxidation that is supported by most dithiolene ligands. A surprising point of difference between [**5**]^2+^ and [**1**]^2+^ is that the latter shows a reversible reduction at −1.62 V versus Fc^+^/Fc, which is confirmed as a two-electron process by use of 1 equiv of Cp*_2_Fe as an internal standard (Figure S55). A related complex with zerovalent Pt, [Pt(dppeb)_2_] {dppeb = 1,2-bis[(diphenylphosphino)ethynyl]benzene}, is reported to undergo a reversible, two-electron oxidation to the corresponding dication.^42^ It is unclear why [**5**]^2+^ and [**1**]^2+^, which appear to share a similarly composed LUMO and which differ only in the identity of the peripheral metallodithiolene groups, diverge in the nature of the cathodic process that they undergo.

Cation [**10**]^+^ sustains a quasireversible oxidation at +0.16 V and a reversible reduction at −1.92 V versus Fc^+^/Fc (Figure 7, bottom), again with an anodic potential shift compared to its charge-neutral homodimetallic analogue. The current amplitude is the same for these two processes. Inspection of the frontier MOs for [**10**]^+^ following a geometry optimization shows both HOMO and LUMO to be essentially degenerate orbital pairs (Figure S50), therefore suggesting that both the oxidation wave and the reduction wave are two simultaneous one-electron processes involving the two distal dithiolene ligands and the two bridging tpbz ligands, respectively. The rather negative −1.92 V potential seen for the reduction in [**10**]^+^ is consistent with its assignment as a tpbz-based process, as similar potentials attributable to tpbz reduction are seen in the charge-neutral dimetallic compounds (*vide supra*).^6^ In contrast, cation [**6**]^+^, which differs from [**10**]^+^ only in having mnt(2−) as the terminal ligand in place of pdt(2−), shows a single reversible reduction at −1.40 V. The 0.5 V less negative potential for this process compared to that in [**10**]^+^, and its apparent one-electron nature compared to Cp*Fe as the standard, point toward a locus for reduction other than the tpbz ligands. We tentatively attribute this feature to Au^+^ + e^–^ → Au^0^ reduction but note that no reversible reduction is reported for the related [Au(dppb)_2_]^+^ cation.^22^

## Conclusions

The principal conclusions that emerge from this work are as follows.

(1) Trimetallic complexes of the general form [(R_2_C_2_S_2_)M(μ-tpbz)M′(μ-tpbz)M(S_2_C_2_R_2_)] are readily prepared in moderate to good yields from open-ended [(R_2_C_2_S_2_)M(μ-tpbz)] and an appropriate precursor for M′ [M′ = Pt^2+^, Cu^+^, Ag^+^, Au^+^, or ReBr(CO)/{Re(CO)_2_}^+^] in a 2:1 ratio.

(2) Trimetallic assemblies with third-row transition metals at the nexus reveal a greater stability that is possibly the result of stronger M–L bonds compared to those of analogous first- and second-row complexes.

(3) Structural authentication of numerous compounds of the type reveals centrosymmetric “S” and herringbone core topologies arising from boat and chair conformations of the tpbz connectors about Pt^2+^ or Re^+^. The Au^+^-linked compounds are spiro-like with an orthogonal disposition of the [(R_2_C_2_S_2_)M(μ-tpbz)] end groups.

(4) A computational evaluation of the conformational energetics of [**1**]^2+^ and [**6**]^+^ finds that Ph···Ph clashing greatly disfavors idealized point group symmetries and that multiple, near isoenergetic minima of *C*_1_, *C*_2_, and/or *C*_*i*_ symmetries are likely pertinent to the solution phase.

(5) ^31^P NMR identifies the trimetallic assemblies by downfield shifts of ∼35–55.5 ppm of the open-ended phosphine signal upon chelation. These shifts are ∼55 ppm, ∼43 ppm and ∼35 ppm for the compounds with Pt^2+^, Re^1+^ and Au^1+^, respectively, at the center.

(6) A limited electrochemistry survey shows that trimetallic [[(R_2_C_2_S_2_)M(μ-tpbz)]_2_M′]^2+/+^ compounds with R = Ph support dithiolene oxidation, but at anodically shifted potentials owing to their cationic charge. Cathodic scanning reveals processes that appear to be, on the collective bases of potential, current amplitude, and computational assessment, either tpbz-based {e.g., [[(Ph_2_C_2_S_2_)Ni(μ-tpbz)]_2_Au]^+^} or metal-based at the central ion {e.g., [[(Ph_2_C_2_S_2_)Pt(μ-tpbz)]_2_Pt]^2+^}.

(7) The trimetallic assemblies reported here are electrically activated two-qubit prototypes in which the negligible exchange coupling allows for convenient switching between the singlet and triplet states. The spin coherence lifetimes offered by these organic ligand radicals^43^ provide a potential platform for optically addressing the central d^10^ ion and generating an array of spin coupling options in a well-characterized ensemble. Demonstrating this design concept would represent a step-change in multi-qubit design.

In continuing work, we target the synthesis of related multimetal systems in which either the linking metal ion(s), the organic ligands, or both sustain reversible electrochemistry leading to the creation of isolable multispin states in which spins are weakly coupled and selectively addressable.

## Supporting Information Available

Procedures for crystal growth, X-ray diffraction data collection, and structure solution and refinement; description of computational procedures; tables summarizing unit cell and refinement data (Tables S1–S4); thermal ellipsoid plots with complete atom labeling (Figures S1–S48); MO energy level diagrams for [**5**]^2+^ and [**10**]^+^ (Figures S49 and S50, respectively); spectroscopic, electrochemical, and analytical data for compounds reported (Figures S51–S126); and coordinates for geometry-optimized [**1**]^2+^ and [**6**]^+^ (PDF)
