# Supplementary material for: Heterotrimetallic Assemblies with 1,2,4,5-Tetrakis(diphenylphosphino)benzene Bridges: Constructs for Controlling the Separation and Spatial Orientation of Redox-Active Metallodithiolene Groups
Source: Inorg Chem. 2022 Oct 25;61(44):17804–18. doi: 10.1021/acs.inorgchem.2c03112 (PMC9644372; doi:10.1021/acs.inorgchem.2c03112)
Supplement: Supplementary file 1 — ic2c03112_si_001.pdf [file ic2c03112_si_001.pdf]

Supporting Information

Heterotrimetallic Assemblies with 1,2,4,5-Tetrakis(diphenylphosphino)benzene Bridges: Constructs for Controlling the Separation and Spatial Orientation of Redox-Active Metallodithiolene Groups

by

Satyendra Kumar,[†,£,*] Malathy Selvachandran,[†,‡] Che Wu,[†] Robert A. Pascal, Jr.,[†]
Xiaodong Zhang,[†] Tod Grusenmeyer,[†,&] Russell H. Schmehl,[†]
Stephen Sproules,[¥] Joel T. Mague,[†] and James P. Donahue[†,*]

[†]Department of Chemistry, Tulane University, 6400 Freret Street, New Orleans, Louisiana, 70118, U.S.A.
[£]Current Address: Xavier University of Louisiana, 1 Drexel Dr., New Orleans, Louisiana, 70125, U.S.A.
[‡]Current Address: University of Jaffna, Vavuniya Campus, Sri Lanka.
[&]Current Address: Air Force Research Laboratory, Dayton, OH.
[¥]WestCHEM, School of Chemistry, University of Glasgow, Glasgow G12 8QQ, United Kingdom.

Table of Contents

**Procedures for Crystal Growth, Collection and Processing of Diffraction Data, and Solving and Refining of Structures**.		S7-S9
**Computational Procedures**.		S9-S10
**References**.		S10-S11
**Table S1**.	Unit cell and refinement data for all M-Pt-M trimetallic compounds.	S12
**Table S2**.	Unit cell and refinement data for all M-Au-M trimetallic compounds.	S13
**Table S3**.	Unit cell and refinement data for all M-Re bimetallic compounds.	S14
**Table S4**.	Unit cell and refinement data for all M-Re-M trimetallic compounds.	S15
**Figure S1**.	Thermal ellipsoid plot (50%) of [(mnt)Ni(μ_2-tpbz)Pt(μ_2-tpbz)Ni(mnt)]$^{2+}$, [**1**]$^{2+}$, with full atom labeling.	S16
**Figure S2**.	Thermal ellipsoid plot (50%) of [(mnt)Ni(μ_2-tpbz)Pt(μ_2-tpbz)Ni(mnt)]$^{2+}$ with partial atom labeling.	S16
**Figure S3**.	Thermal ellipsoid plot (50%), alternate view, of [(mnt)Ni(μ_2-tpbz)Pt(μ_2-tpbz)Ni(mnt)]$^{2+}$ with full atom labeling.	S17
**Figure S4**.	Thermal ellipsoid plot (50%), alternate view, of [(mnt)Ni(μ_2-tpbz)Pt(μ_2-tpbz)Ni(mnt)]$^{2+}$ with partial atom labeling.	S17
**Figure S5**.	Core topology of [(mnt)Ni(μ_2-tpbz)Pt(μ_2-tpbz)Ni(mnt)]$^{2+}$ with 50% ellipsoids.	S18
**Figure S6**.	Atom labeling for [CF_3SO_3]$^{1-}$ counteranion and for interstitial solvent in [(mnt)Ni(μ_2-tpbz)Pt(μ_2-tpbz)Ni(mnt)][CF_3SO_3]$_2$·3$C_6H_5NO_2$·2Et_2O.	S18
**Figure S7**.	Packing of [**1**]$^{2+}$ with [CF_3SO_3]$^{1-}$ counteranions (40% ellipsoids).	S19
**Figure S8**.	Thermal ellipsoid plot (50%) of [(pdt)Ni(μ_2-tpbz)Pt(μ_2-tpbz)Ni(pdt]$^{2+}$, [**3**]$^{2+}$.	S20
**Figure S9**.	Atom labeling for [CF_3SO_3]$^{1-}$ counteranion and for interstitial solvent in [(pdt)Ni(μ_2-tpbz)Pt(μ_2-tpbz)Ni(pdt)][CF_3SO_3]$_2$·3DMF.	S20
**Figure S10**.	Thermal ellipsoid plot (40%) of [(pdt)Pd(μ_2-tpbz)Pt(μ_2-tpbz)Pd(pdt)]$^{2+}$, [**4**]$^{2+}$, with full atom labeling.	S21
**Figure S11**.	Thermal ellipsoid plot (40%) of [(pdt)Pd(μ_2-tpbz)Pt(μ_2-tpbz)Pd(pdt)]$^{2+}$, [**4**]$^{2+}$, with partial atom labeling.	S21
**Figure S12**.	Thermal ellipsoid plot (40%) of [(pdt)Pd(μ_2-tpbz)Pt(μ_2-tpbz)Pd(pdt)]$^{2+}$, [**4**]$^{2+}$, alternate view, with full atom labeling	S22
**Figure S13**.	Thermal ellipsoid plot (40%) of [(pdt)Pd(μ_2-tpbz)Pt(μ_2-tpbz)Pd(pdt)]$^{2+}$, [**4**]$^{2+}$, alternate view, with partial atom labeling.	S22
**Figure S14**.	Atom labeling for [CF_3SO_3]$^{1-}$ counteranion and for interstitial solvent in [(pdt)Pd(μ_2-tpbz)Pt(μ_2-tpbz)Pd(pdt)][CF_3SO_3]$_2$·3.5DMF·2(H_2O).	S23
**Figure S15**.	Thermal ellipsoid plot (50%) of [(pdt)Pt(μ_2-tpbz)Pt(μ_2-tpbz)Pt(pdt)]$^{2+}$, [**5**]$^{2+}$, with full atom labeling.	S24
**Figure S16**.	Thermal ellipsoid plot (50%) of [(pdt)Pt(μ_2-tpbz)Pt(μ_2-tpbz)Pt(pdt)]$^{2+}$, [**5**]$^{2+}$, with partial atom labeling.	S24
**Figure S17**.	Atom labeling for [CF_3SO_3]$^{1-}$ counteranion and for interstitial solvent in [(pdt)Pt(μ_2-tpbz)Pt(μ_2-tpbz)Pt(pdt)][CF_3SO_3]$_2$·4DMF·2H_2O.	S25
**Figure S18**.	Thermal ellipsoid plot (50%) of [(mnt)Ni(μ_2-tpbz)Au(μ_2-tpbz)Ni(mnt)]$^{1+}$, [**6**]$^{1+}$, cation 1 of 2, with full atom labeling.	S26
**Figure S19**.	Thermal ellipsoid plot (50%) of [(mnt)Ni(μ_2-tpbz)Au(μ_2-tpbz)Ni(mnt)]$^{1+}$, [**6**]$^{1+}$, cation 2 of 2, with full atom labeling.	S26

Table of Contents, Continued

Figure S20. Atom labeling for $[CF_3SO_3]^{1-}$ counteranion and for interstitial solvent in [**6**]$[CF_3SO_3]\cdot½C_6H_5NO_2\cdot C_6H_6\cdot{}^t$BuOMe. S27

Figure S21. Thermal ellipsoid plot (50%) of [(adt)Ni(μ_2-tpbz)Au(μ_2-tpbz)Ni(adt)]$^{1+}$, [**7**]$^{1+}$, with full atom labeling. S28

Figure S22. Atom labeling for $[Cl]^{1-}$ counteranion and for interstitial solvent in [**7**][Cl]·½(DMI). S28

Figure S23. Thermal ellipsoid plot (30%) of [(pdt)Ni(μ_2-tpbz)Au(μ_2-tpbz)Ni(pdt)]$^{1+}$, [**10**]$^{1+}$, with full atom labeling. S29

Figure S24. Atom labeling for $[CF_3SO_3]^{1-}$ counteranion and for interstitial solvent in [**10**]$[CF_3SO_3]$·2(1,3-Me_2-2-imidazolidinone)·½Et_2O. S29

Figure S25. Thermal ellipsoid plot (40%) of [(pdt)Pd(μ_2-tpbz)Au(μ_2-tpbz)Pd(pdt)]$^{1+}$, [**11**]$^{1+}$, with atom labeling including only one positional variant for disordered Ph groups. S30

Figure S26. Thermal ellipsoid plot (40%) of [(pdt)Pd(μ_2-tpbz)Au(μ_2-tpbz)Pd(pdt)]$^{1+}$, [**11**]$^{1+}$, with atom labeling for second orientation of each disordered Ph group. S30

Figure S27. Atom labeling for disordered $[CF_3SO_3]^{1-}$ anion in [(pdt)Pd(μ_2-tpbz)Au(μ_2-tpbz)Pd(pdt)]$[CF_3SO_3]$, [**11**]$[CF_3SO_3]$. Ellipsoids are presented at 30%. S31

Figure S28. Atom labeling for [(mnt)Ni(μ_2-tpbz)ReBr$(CO)_3$], **13**, (pseudopolymorph 1, JPD979) with 40% ellipsoids. S32

Figure S29. Partial atom labeling for **13**·Et_2O (JPD 979) with 40% ellipsoids. S32

Figure S30. Atom labeling for [(mnt)Ni(μ_2-tpbz)ReBr$(CO)_3$], **13**, (pseudopolymorph 2, JPD1046) with 40% ellipsoids. S33

Figure S31. Alternate view of **13** (JPD 1046) with 40% ellipsoids, showing disorder. S33

Figure S32. Atom labeling for [(pdt)Pd(μ_2-tpbz)ReBr$(CO)_3$], **14**, with 50% ellipsoids. S34

Figure S33. Partial atom labeling for **14** with 50% ellipsoids. S34

Figure S34. Atom labeling for [(pdt)Pt(μ_2-tpbz)ReBr$(CO)_3$], **15**, with 40% ellipsoids. S35

Figure S35. Partial atom labeling for **15** with 40% ellipsoids. S35

Figure S36. Side view of [(mnt)Ni(μ_2-tpbz)ReBr(CO)(μ_2-tpbz)Ni(mnt)], **16**, (30% ellipsoids) with partial atom labeling. S36

Figure S37. Side view of [(mnt)Ni(μ_2-tpbz)ReBr(CO)(μ_2-tpbz)Ni(mnt)], **16**, (30% ellipsoids) with complete atom labeling. S36

Figure S38. Alternate side view of [(mnt)Ni(μ_2-tpbz)ReBr(CO)(μ_2-tpbz)Ni(mnt)], **16**, (30% ellipsoids) with complete atom labeling. S37

Figure S39. Side view of [(mnt)Ni(μ_2-tpbz)Re$(CO)_2$(μ_2-tpbz)Ni(mnt)], [**17**]$^{1+}$, (30% ellipsoids) with complete atom labeling. S38

Figure S40. Side view of [(mnt)Ni(μ_2-tpbz)Re$(CO)_2$(μ_2-tpbz)Ni(mnt)], [**17**]$^{1+}$, (30% ellipsoids) with partial atom labeling. S38

Figure S41. Side view of [(mnt)Ni(μ_2-tpbz)Re$(CO)_2$(μ_2-tpbz)Ni(mnt)], [**17**]Br, (30% ellipsoids) with partial atom labeling. S39

Figure S42. Partial atom labeling for [(pdt)Pt(μ_2-tpbz)ReBr(CO)(μ_2-tpbz)Pt(pdt)], **18**, (35% ellipsoids). S40

Figure S43. Full atom labeling for [(pdt)Pt(μ_2-tpbz)ReBr(CO)(μ_2-tpbz)Pt(pdt)], **18**, (35% ellipsoids). S40

Table of Contents, Continued

Figure S44. Atom labeling for interstitial solvent in [(pdt)Pt(μ_2-tpbz)ReBr(CO)(μ_2-tpbz)Pt(pdt)]·4($C_6H_5NO_2$). S41

Figure S45. Atom labeling for [(pdt)Pt(μ_2-tpbz)Re$(CO)_2$(μ_2-tpbz)Pt(pdt)]$^{1+}$, [**19**]$^{1+}$, (30% ellipsoids). S41

Figure S46. Atom labeling for interstitial $PhNO_2$ molecule 1 in [**19**][Br]·3($PhNO_2$)·^{t}BuOMe (30% ellipsoids). S42

Figure S47. Atom labeling for interstitial $PhNO_2$ molecule 2 in [**19**][Br]·3($PhNO_2$)·^{t}BuOMe (30% ellipsoids). S42

Figure S48. Atom labeling for [Br]$^{1-}$ and interstitial ^{t}BuOMe in [**19**][Br]·3($PhNO_2$)·^{t}BuOMe (30% ellipsoids). S42

Figure S49. MO energy level diagram for [($Ph_2C_2S_2$)Pt(tpbz)Pt(tpbz)Pt($S_2C_2Ph_2$)]$^{2+}$. S43

Figure S50. MO energy level diagram for [($Ph_2C_2S_2$)Ni(tpbz)Au(tpbz)Ni($S_2C_2Ph_2$)]$^{1+}$. S44

Figure S51. ^{1}H NMR spectrum (DMSO-d_6) of [(mnt)Ni(tpbz)Pt(tpbz)Ni(mnt)][CF_3SO_3]$_2$. S45

Figure S52. ^{31}P NMR spectrum (DMSO-d_6) of [(mnt)Ni(tpbz)Pt(tpbz)Ni(mnt)][CF_3SO_3]$_2$. S45

Figure S53. UV-vis spectrum (DMF) of [(mnt)Ni(tpbz)Pt(tpbz)Ni(mnt)][CF_3SO_3]$_2$. S46

Figure S54. Cyclic voltammogram of [(mnt)Ni(tpbz)Pt(tpbz)Ni(mnt)][CF_3SO_3]$_2$ in DMF with [^{n}Bu_4N][PF_6], 100 mV/sec. S46

Figure S55. Cyclic voltammogram of [(mnt)Ni(tpbz)Pt(tpbz)Ni(mnt)][CF_3SO_3]$_2$ in DMF with [^{n}Bu_4N][PF_6], 100 mV/sec, with Cp*$_2$Fe as standard. S47

Figure S56. Differential pulse voltammogram of [(mnt)Ni(tpbz)Pt(tpbz)Ni(mnt)]$^{2+}$ in DMF with [^{n}Bu_4N][PF_6], 50 mV pulse amplitude. S47

Figure S57. Mass spectrum (ESI+) of [(mnt)Ni(tpbz)Pt(tpbz)Ni(mnt)][CF_3SO_3]$_2$. S48

Figure S58. ^{1}H NMR spectrum (DMSO-d_6) of [(adt)Ni(tpbz)Pt(tpbz)Ni(adt)][CF_3SO_3]$_2$. S49

Figure S59. ^{31}P NMR spectrum (DMSO-d_6) of [(adt)Ni(tpbz)Pt(tpbz)Ni(adt)][CF_3SO_3]$_2$. S49

Figure S60. Mass spectrum (ESI+) of [(adt)Ni(tpbz)Pt(tpbz)Ni(adt)][CF_3SO_3]$_2$. S50

Figure S61. ^{1}H NMR spectrum (DMSO-d_6) of [(pdt)Ni(tpbz)Pt(tpbz)Ni(pdt)][CF_3SO_3]$_2$. S51

Figure S62. ^{31}P NMR spectrum (DMSO-d_6) of [(pdt)Ni(tpbz)Pt(tpbz)Ni(pdt)][CF_3SO_3]$_2$. S51

Figure S63. Mass spectrum (ESI+) of [(pdt)Ni(tpbz)Pt(tpbz)Ni(pdt)][CF_3SO_3]$_2$. S52

Figure S64. ^{1}H NMR spectrum (DMSO-d_6) of [(pdt)Pd(tpbz)Pt(tpbz)Pd(pdt)][CF_3SO_3]$_2$. S53

Figure S65. ^{31}P NMR spectrum (DMSO-d_6) of [(pdt)Pd(tpbz)Pt(tpbz)Pd(pdt)][CF_3SO_3]$_2$. S53

Figure S66. Mass spectrum (ESI+) of [(pdt)Pd(tpbz)Pt(tpbz)Pd(pdt)][CF_3SO_3]$_2$. S54

Figure S67. ^{1}H NMR spectrum (DMSO-d_6) of [(pdt)Pt(tpbz)Pt(tpbz)Pt(pdt)][CF_3SO_3]$_2$. S55

Figure S68. ^{31}P NMR spectrum (DMSO-d_6) of [(pdt)Pt(tpbz)Pt(tpbz)Pt(pdt)][CF_3SO_3]$_2$. S55

Figure S69. Cyclic voltammogram of [(pdt)Pt(tpbz)Pt(tpbz)Pt(pdt)][CF_3SO_3]$_2$ in DMF with [^{n}Bu_4N][PF_6], 100 mV/sec. S56

Figure S70. Cyclic voltammogram of [(pdt)Pt(tpbz)Pt(tpbz)Pt(pdt)][CF_3SO_3]$_2$ in DMF with [^{n}Bu_4N][PF_6], 100 mV/sec, with cathodic scanning only to first reduction. S56

Figure S71. Differential pulse voltammogram of [(pdt)Pt(tpbz)Pt(tpbz)Pt(pdt)] [CF_3SO_3]$_2$. in DMF with [^{n}Bu_4N][PF_6], 50 mV pulse amplitude. S57

Figure S72. UV-vis spectrum (DMF) of [(pdt)Pt(tpbz)Pt(tpbz)Pt(pdt)][CF_3SO_3]$_2$. S57

Figure S73. Mass spectrum (ESI+) of [(pdt)Pt(tpbz)Pt(tpbz)Pt(pdt)][CF_3SO_3]$_2$. S58

Figure S74. ^{1}H NMR spectrum (CD_2Cl_2) of [(mnt)Ni(tpbz)Au(tpbz)Ni(mnt)][CF_3SO_3]. S59

Figure S75. ^{31}P-{^{1}H} NMR (CD_2Cl_2) of [(mnt)Ni(tpbz)Au(tpbz)Ni(mnt)][CF_3SO_3]. S59

Figure S76. (a) Cyclic voltammogram of [(mnt)Ni(tpbz)Au(tpbz)Ni(mnt)][CF_3SO_3] in DMF with [^{n}Bu_4N][PF_6], 100 mV/sec; (b) CV with Cp*$_2$Fe as standard. S60

Table of Contents, Continued

**Figure S77**.	Mass spectrum (ESI+) of [(mnt)Ni(tpbz)Au(tpbz)Ni(mnt)][CF_3SO_3].	S61
**Figure S78**.	2nd mass spectrum (ESI+) of [(mnt)Ni(tpbz)Au(tpbz)Ni(mnt)][CF_3SO_3].	S62
**Figure S79**.	UV-vis spectrum (CH_2Cl_2) of [(mnt)Ni(tpbz)Au(tpbz)Ni(mnt)][CF_3SO_3].	S62
**Figure S80**.	^{1}H NMR spectrum (CD_2Cl_2) of [(adt)Ni(tpbz)Au(tpbz)Ni(adt)][Cl].	S63
**Figure S81**.	^{31}P-{^{1}H} NMR (CD_2Cl_2) of [(adt)Ni(tpbz)Au(tpbz)Ni(adt)][Cl].	S63
**Figure S82**.	Mass spectrum (ESI+) of [(adt)Ni(tpbz)Au(tpbz)Ni(adt)][Cl].	S64
**Figure S83**.	2nd mass spectrum (ESI+) of [(adt)Ni(tpbz)Au(tpbz)Ni(adt)][Cl].	S65
**Figure S84**.	UV-vis spectrum (CH_2Cl_2) of [(adt)Ni(tpbz)Au(tpbz)Ni(adt)][Cl].	S65
**Figure S85**.	^{31}P-{^{1}H} NMR spectrum (CD_2Cl_2) of [(pdt)Ni(tpbz)Cu(tpbz)Ni(pdt)]$^+$.	S66
**Figure S86**.	UV-vis spectrum (CH_2Cl_2) of [(pdt)Ni(tpbz)Cu(tpbz)Ni(pdt)][$BArF_{24}$].	S66
**Figure S87**.	X-band EPR spectrum of [(pdt)Ni(tpbz)Cu(tpbz)Ni(pdt)]$^{3+}$ in CH_2Cl_2 at 293 K.	S67
**Figure S88**.	Cyclic voltammogram of [($Ph_2C_2S_2$)Ni(tpbz)Cu(tpbz)Ni($S_2C_2Ph_2$)]$^+$ in CH_2Cl_2 with (0.10 *M* [^{n}Bu_4N][PF_6] supporting electrolyte) at 22 °C at a scan rate of 100 mV s^{-1}.	S67
**Figure S89**.	Mass spectrum (ESI+) of [($Ph_2C_2S_2$)Ni(tpbz)Cu(tpbz)Ni($S_2C_2Ph_2$)]$^+$.	S68
**Figure S90**.	Fragment peaks (ESI+) of [($Ph_2C_2S_2$)Ni(tpbz)Cu(tpbz)Ni($Ph_2C_2S_2$)]$^+$.	S69
**Figure S91**.	^{31}P-{1H} NMR spectrum of [($Ph_2C_2S_2$)Ni(tpbz)Ag(tpbz)Ni($Ph_2C_2S_2$)]$^+$.	S70
**Figure S92**.	Mass spectrum (ESI+) of [($Ph_2C_2S_2$)Ni(tpbz)Ag(tpbz)Ni($Ph_2C_2S_2$)]$^+$.	S70
**Figure S93**.	^{1}H NMR spectrum (CD_2Cl_2) of [(pdt)Ni(tpbz)Au(tpbz)Ni(pdt)][CF_3SO_3].	S71
**Figure S94**.	^{31}P-{^{1}H} NMR (CD_2Cl_2) of [(pdt)Ni(tpbz)Au(tpbz)Ni(pdt)][CF_3SO_3].	S71
**Figure S95**.	Cyclic voltammogram of [(pdt)Ni(tpbz)Au(tpbz)Ni(pdt)][CF_3SO_3] in CH_2Cl_2 with [^{n}Bu_4N][PF_6], 100 mV/sec.	S72
**Figure S96**.	Mass spectrum (ESI+) of [(pdt)Ni(tpbz)Au(tpbz)Ni(pdt)][CF_3SO_3], assuming 2+ charge.	S72
**Figure S97**.	Mass spectrum (ESI+) of [(pdt)Ni(tpbz)Au(tpbz)Ni(pdt)][CF_3SO_3].	S73
**Figure S98**.	UV-vis spectrum (CH_2Cl_2) of [(pdt)Ni(tpbz)Au(tpbz)Ni(pdt)][CF_3SO_3].	S74
**Figure S99**.	Overlay UV-vis spectra of [(pdt)Ni(tpbz)Au(tpbz)Ni(pdt)][CF_3SO_3], [(pdt)Pd(tpbz)Au(tpbz)Pd(pdt)][CF_3SO_3] and [(pdt)Pt(tpbz)Au(tpbz)Pt(pdt)][CF_3SO_3] in CH_2Cl_2.	S74
**Figure S100**.	^{1}H NMR spectrum (CD_2Cl_2) of [(pdt)Pd(tpbz)Au(tpbz)Pd(pdt)][CF_3SO_3].	S75
**Figure S101**.	^{31}P NMR spectrum (CD_2Cl_2) of [(pdt)Pd(tpbz)Au(tpbz)Pd(pdt)][CF_3SO_3].	S75
**Figure S102**.	Mass spectrum (ESI+) of [(pdt)Pd(tpbz)Au(tpbz)Pd(pdt)][CF_3SO_3].	S76
**Figure S103**.	UV-vis spectrum (CH_2Cl_2) of [(pdt)Pd(tpbz)Au(tpbz)Pd(pdt)][CF_3SO_3].	S76
**Figure S104**.	^{1}H NMR spectrum (CD_2Cl_2) of [(pdt)Pt(tpbz)Au(tpbz)Pt(pdt)][CF_3SO_3].	S77
**Figure S105**.	^{31}P NMR spectrum (CD_2Cl_2) of [(pdt)Pt(tpbz)Au(tpbz)Pt(pdt)][CF_3SO_3].	S77
**Figure S106**.	Mass spectrum (ESI+) of [(pdt)Pt(tpbz)Au(tpbz)Pt(pdt)][CF_3SO_3].	S78
**Figure S107**.	UV-vis spectrum (CH_2Cl_2) of [(pdt)Pt(tpbz)Au(tpbz)Pt(pdt)][CF_3SO_3].	S78
**Figure S108**.	X-band EPR spectrum of [(pdt)Pt(tpbz)Au(tpbz)Pt(pdt)]$^{3+}$ in CH_2Cl_2 at 293 K.	S79
**Figure S109**.	X-band EPR spectrum of [(pdt)Pt(tpbz)Au(tpbz)Pt(pdt)]$^{3+}$ in CH_2Cl_2 at 130 K.	S79
**Figure S110**.	Comparison of the X-band EPR spectra of [(pdt)Ni(tpbz)Cu(tpbz)Ni(pdt)]$^{3+}$ and [(pdt)Pt(tpbz)Au(tpbz)Pt(pdt)]$^{3+}$ recorded in CH_2Cl_2 solution at 293 K.	S80
**Figure S111**.	^{31}P-{^{1}H} NMR spectrum ($CDCl_3$) of [(mnt)Ni(tpbz)ReBr$(CO)_3$], **13**.	S81

Table of Contents, Continued

**Figure S112**.	Mass spectrum (ESI+) of [(mnt)Ni(tpbz)ReBr(CO)$_3$], **13**.	S81
**Figure S113**.	UV-vis spectrum (CH_2Cl_2) of [(mnt)Ni(tpbz)ReBr(CO)$_3$], **13**.	S82
**Figure S114**.	^{31}P-{^{1}H} NMR spectrum ($CDCl_3$) of [(pdt)Pd(tpbz)ReBr(CO)$_3$], **14**.	S83
**Figure S115**.	Mass spectrum (ESI+) of [(pdt)Pd(tpbz)ReBr(CO)$_3$], **14**.	S83
**Figure S116**.	^{31}P-{^{1}H} NMR spectrum ($CDCl_3$) of [(pdt)Pt(tpbz)ReBr(CO)$_3$], **15**.	S84
**Figure S117**.	Mass spectrum (ESI+) of [(pdt)Pt(tpbz)ReBr(CO)$_3$], **15**.	S84
**Figure S118**.	Element analysis request form for [(pdt)Pt(tpbz)ReBr(CO)$_3$], **15**.	S85
**Figure S119**.	Element analysis results for [(pdt)Pt(tpbz)ReBr(CO)$_3$], **15**.	S86
**Figure S120.**	^{31}P-{^{1}H} NMR (CD_2Cl_2) of [(mnt)Ni(tpbz)ReBr(CO)(tpbz)Ni(mnt)].	S87
**Figure S121**.	Mass spectrum (ESI+) of [(mnt)Ni(tpbz)ReBr(CO)(tpbz)Ni(mnt)].	S87
**Figure S122**.	^{31}P-{^{1}H} NMR (CD_2Cl_2) of [(mnt)Ni(tpbz)Re(CO)$_2$(tpbz)Ni(mnt)]Br.	S88
**Figure S123**.	Mass spectrum (ESI+) of [(mnt)Ni(tpbz)Re(CO)$_2$(tpbz)Ni(mnt)]Br.	S88
**Figure S124**.	^{31}P-{^{1}H} NMR spectrum ($CDCl_3$) of [(pdt)Pt(tpbz)Re(CO)Br(tpbz)Pt(pdt)].	S89
**Figure S125**.	Mass spectrum (ESI+) of [(pdt)Pt(tpbz)Re(CO)Br(tpbz)Pt(pdt)].	S89
**Figure S126**.	UV-vis spectrum (CH_2Cl_2) of [(pdt)Pt(tpbz)Re(CO)Br(tpbz)Pt(pdt)].	S90
**Figure S127**.	^{31}P-{^{1}H} NMR spectrum ($CDCl_3$) of [(pdt)Pt(tpbz)Re(CO)$_2$(tpbz)Pt(pdt)]Br.	S91
**Figure S128**.	Mass spectrum (ESI+) of [(pdt)Pt(tpbz)Re(CO)$_2$(tpbz)Pt(pdt)]Br.	S91
**Table S5**.	Atomic coord. for D_{2h} complex [**1**]$^{2+}$, B3PW91/LANL2DZ-optimized.	S92-S95
**Table S6**.	Atomic coord. for D_2 complex [**1**]$^{2+}$, B3PW91/LANL2DZ-optimized.	S96-S99
**Table S7**.	Atomic coord. for C_i complex [**1**]$^{2+}$, B3PW91/LANL2DZ-optimized.	S100-S103
**Table S8**.	Atomic coord. for C_1 complex [**1**]$^{2+}$, B3PW91/LANL2DZ-optimized.	S104-S107
**Table S9**.	Atomic coord. for C_2 complex [**1**]$^{2+}$, B3PW91/LANL2DZ-optimized.	S108-S111
**Table S10**.	Atomic coord. for D_2 complex tetracyano-[**6**]$^{1+}$, B3PW91/LANL2DZ-optimized.	S112-S115
**Table S11**.	Atomic coord. for C_1 complex tetracyano-[**6**]$^{1+}$, B3PW91/LANL2DZ-optimized.	S116-S119
**Table S12**.	Atomic coord. for C_2 complex tetracyano-[**6**]$^{1+}$, B3PW91/LANL2DZ-optimized.	S120-S123
**Table S13**.	Atomic coord. for D_{2h} complex [**1**]$^{2+}$, B3PW91/Def2SVP-optimized.	S124-S127
**Table S14**.	Atomic coord. for D_2 complex [**1**]$^{2+}$, B3PW91/Def2SVP-optimized.	S128-S131
**Table S15**.	Atomic coord. for C_i complex [**1**]$^{2+}$, B3PW91/Def2SVP-optimized.	S132-S135
**Table S16**.	Atomic coord. for C_1 complex [**1**]$^{2+}$, B3PW91/Def2SVP-optimized.	S136-S139
**Table S17**.	Atomic coord. for C_2 complex [**1**]$^{2+}$, B3PW91/Def2SVP-optimized.	S140-S143

Procedures for Crystal Growth, Collection and Processing of Diffraction Data, and Solving and Refining of Structures.

Ligand abbreviations used in the procedural description below are mnt(2–) = maleonitriledithiolate(2–) = $[(NC)_2C_2S_2]^{2-}$; tpbz = 1,2,4,5- tetrakis(diphenylphosphino)benzene; pdt(2–) = phenyldithiolene(2–) = $[Ph_2C_2S_2]^{2-}$; adt(2–) = anisyldithiolene(2–) = $[(H_3CO\text{-}p\text{-}C_6H_4)_2C_2S_2]^{2-}$. DMI represents 1,3-dimethyl-2-imidazolidinone.

All crystals from which X-ray diffraction data were collected were prepared by the small scale vial-in-a-vial vapor diffusion technique, where the solvent/diffusing vapor pair were as follows: $[(mnt)Ni(tpbz)Pt(tpbz)Ni(mnt)][CF_3SO_3]_2\cdot 3(C_6H_5NO_2)\cdot 2Et_2O$ (orange plates): $C_6H_5NO_2/Et_2O$; $[(pdt)Ni(tpbz)Pt(tpbz)Ni(pdt)][CF_3SO_3]_2\cdot 3(DMF)$ (orange plates): DMF/Et_2O; $[(pdt)Pd(tpbz)Pt(tpbz)Pd(pdt)][CF_3SO_3]_2\cdot 3.5(DMF)\cdot 2H_2O$ (red plates): DMF/Et_2O; $[(pdt)Pt(tpbz)Pt(tpbz)Pt(pdt)][CF_3SO_3]_2\cdot 4(DMF)\cdot 2H_2O$ (red columns): DMF/Et_2O; $[(mnt)Ni(tpbz)Au(tpbz)Ni(mnt)][CF_3SO_3]\cdot ½PhNO_2\cdot C_6H_6\cdot {}^tBuOMe$ (orange plates): $PhNO_2/C_6H_6,{}^tBuOMe$; $[(adt)Ni(tpbz)Au(tpbz)Ni(adt)][Cl]\cdot ½(1,3\text{-}Me_2\text{-}2\text{-imidazolidinone})$ (dark orange blocks): $1,3\text{-}Me_2\text{-}2\text{-imidazolidinone}/Et_2O$; $[(pdt)Ni(tpbz)Au(tpbz)Ni(pdt)][CF_3SO_3]\cdot 2(1,3\text{-}Me_2\text{-}2\text{-imidazolidinone})\cdot ½Et_2O$ (green-yellow plates): $1,3\text{-}Me_2\text{-}2\text{-imidazolidinone}/Et_2O$; $[(pdt)Pd(tpbz)Au(tpbz)Pd(pdt)][CF_3SO_3]$ (red-orange blocks): $1,3\text{-}Me_2\text{-}2\text{-imidazolidinone}/Et_2O$; $[(mnt)Ni(tpbz)ReBr(CO)_3]\cdot Et_2O$ (orange blocks): CH_2Cl_2/Et_2O; $[(pdt)Pd(tpbz)ReBr(CO)_3]$ (pale brown needles): CH_2Cl_2/Et_2O; $[(pdt)Pt(tpbz)ReBr(CO)_3]$ (orange columns): CH_2Cl_2/Et_2O; $[(mnt)Ni(tpbz)ReBr(CO)(tpbz)Ni(mnt)]\cdot 2(1,3\text{-}Me_2\text{-}2\text{-imidazolidinone})\cdot 2Et_2O$ (orange plates): $1,3\text{-}Me_2\text{-}2\text{-imidazolidinone}/Et_2O$; $[(mnt)Ni(tpbz)Re(CO)_2(tpbz)Ni(mnt)][Br]$ (orange plates): $CH_2Cl_2/{}^tBuOMe$; $[(pdt)Pt(tpbz)ReBr(CO)(tpbz)Pt(pdt)]\cdot 4(PhNO_2)$ (orange plates): $PhNO_2/Et_2O$; $[(pdt)Pt(tpbz)Re(CO)_2(tpbz)Pt(pdt)][Br]\cdot 3PhNO_2\cdot {}^tBuOMe$ (red-orange columns): $PhNO_2/{}^tBuOMe$.

All crystals were coated with paratone oil and mounted on the end of a nylon loop attached to the end of the goniometer. Data were collected at 100, 150, or 200 K under a dry N_2 stream supplied under the control of an Oxford Cryostream 800 attachment. The data collection instrument was either a Bruker Smart APEX II CCD diffractometer equipped with a Mo fine-focus sealed tube providing radiation at λ = 0.71073 nm or a Bruker D8 Quest Photon 3 diffractometer that similarly operated with the Mo Kα 0.71073 nm light source.

The data sets for $[(mnt)Ni(tpbz)Pt(tpbz)Ni(mnt)][OTf]_2 \cdot 3C_6H_5NO_2 \cdot 2Et_2O$, $[(pdt)Ni(tpbz)Pt(pdt)Ni(pdt)][OTf]_2 \cdot 3DMF$, $[(pdt)Pd(tpbz)Pt(tpbz)Pd(pdt)][OTf]_2 \cdot 3.5DMF \cdot 2H_2O$, $[(pdt)Pt(tpbz)Pt(tpbz)Pt(pdt)][OTf]_2 \cdot 4DMF \cdot 2H_2O$, $[(pdt)Pd(tpbz)ReBr(CO)_3]$, $[(mnt)Ni(tpbz)ReBr(CO)(tpbz)Ni(mnt)] \cdot 2DMI \cdot 2Et_2O$, $[(mnt)Ni(tpbz)Re(CO)_2(tpbz)Ni(mnt)][Br]$, and $[pdt)Pt(tpbz)Re(CO)_2(tpbz)Pt(mnt)][Br] \cdot 3PhNO_2 \cdot {}^tBuOMe$ were collected with a programmed routine of three sets of 363 frames in ω (0.5º/scan) with φ held constant at 0°, 120°, and then 240º. The frame times were 120, 90, 90, 180, 120, 60, 120, and 120 seconds/frame, respectively. The data for $[(mnt)Ni(tpbz)Au(tpbz)Ni(mnt)][OTf] \cdot ½PhNO_2 \cdot C_6H_6 \cdot {}^tBuOMe$ (7 sets of 336 frames and 1 set of 383 frames), $[(pdt)Ni(tpbz)Au(tpbz)Ni(pdt)][OTf] \cdot 2DMI \cdot ½Et_2O$ (7 sets of 377 frames and 1 set of 719 frames), [(pdt)Pd(tpbz)Au(tpbz)Pd(pdt)][OTf] (7 x 395 frames and 1 x 720 frames), [(adt)Ni(tpbz)Au(tpbz)Ni(adt)][Cl]·½DMI (7 x 395 frames and 2 x 719 frames), and $[(pdt)Pt(tpbz)ReBr(CO)(tpbz)Pt(pdt)] \cdot 4C_6H_5NO_2$ (5 sets of 336 frames, and 1 set of 720 frames) were collected with 0.5° width in ω or ϕ with scan parameters determined by the strategy routine within *APEX3*.[1] Raw data were reduced to F^2 values using *SAINT*,[2] and a global refinement of unit cell parameters was performed using ~5500–9700 selected reflections from the full data sets. For $[(Ph_2C_2S_2)Pt(tpbz)ReBr(CO)_3]$, analysis with *CELL_NOW*[3] of 1008 selected reflections having $I/\sigma(I) > 15$ from the full data set showed the crystal to belong to the triclinic system and to consist of two components separated by a 7° rotation about the real axis –0.921 –0.336 1.000. The raw data were processed using the multi-component version of *SAINT* under control of the two-component orientation file generated by *CELL_NOW*,[3] and an absorption correction was applied using the *TWINABS* routine.[4] All other data sets were corrected for absorption on the basis of multiple measurements of symmetry equivalent reflections or by numerical methods with the use of *SADABS*,[5] as described by Krause *et al*.[6] All structure solutions were obtained by direct methods using *SHELXT*,[7] while refinements were accomplished by full-matrix least-squares procedures using *SHELXL*.[8] The *SHELXL* program is incorporated into both the *SHELXTL*[9] and *APEX3*[1] software suites.

For all the structures, hydrogen atoms attached to carbon were placed in calculated positions (C---H = 0.95 Å) and were included as riding contributions with isotropic displacement parameters 1.2 times those of the attached atoms. In many instances, individual phenyl rings of the tpbz ligand

or the $[Ph_2C_2S_2]^{2-}$ or $[(MeO\text{-}p\text{-}C_6H_4)_2C_2S_2]^{2-}$ ligands were disordered over two orientations and were treated as rigid hexagons using a split-atom model with a site-occupancy distribution determined as a best-fit by the refinement software. In a few cases, disorder included a PPh_2 fragment of the tpbz ligand or complete dithiolene ligand but was similarly modeled. The $CF_3SO_3^{1-}$ counteranions were typically disordered, which demanded implementation of heavy interatomic distance restraints in order to achieve stable refinement. Interstitial solvent molecules that were similarly afflicted with disorder were generally handled with what minimal interatomic distance restraints were necessary to accomplish good refinement behavior. For both [(mnt)Ni(tpbz)ReBr(CO)(tpbz)Ni(mnt)]·2(1,3-Me_2-2-imidazolidinone)·2Et_2O and [(pdt)Pt(tpbz)ReBr(CO)(tpbz)Pt(pdt)]·4$C_6H_5NO_2$, a crystallographic inversion center that was coincident with the position of Re imposed disorder between the bromide and carbonyl ligands. For the latter compound, the carbonyl group was constrained to be linear and to have Re–C and C≡O distances fixed at values similar to those in related compounds.[10] Refinement of atoms that were subject to disorder was often, but not always, limited to isotropic rather than full anisotropic treatment. In 9 of the 16 structures reported here, small amounts of density remote from the main coordination complex and attributable to partially occupied/disordered solvent sites were removed with the *SQUEEZE* routine in *PLATON*.[11] All structures were checked for overlooked symmetry and other errors by the checkCIF service provided by the International Union of Crystallography.[12]

Computational Procedures and Details

Theoretical calculations were carried out at the Supercomputing Facility at Tulane University New Orleans. The GAUSSIAN-09 package[13] was used to perform all calculations. All geometry optimizations, as well as the single point calculations, were conducted using the Becke, 3-Parameter, Lee-Yang-Parr (B3LYP) level of theory[14] with typical basis sets. Geometry optimizations of trimetallic structures were performed with no symmetry restrictions, and crystallographic data were used for starting x, y, z coordinates. Frequency calculations were done to confirm the validity of optimized structures. For the transition metals (nickel, palladium, platinum, rhenium and gold), a double-ζ (DZ) basis set with an effective electron core potential (LANL2DZ ECP) was implemented.[15] The 6-31G (d,p) basis set was chosen for the light main group elements (C and N), whereas the triple-ζ (TZVP) was used for the heavier elements (S and P), and a Gaussian split valence (SV) basis set[16] was used for the hydrogen atoms. The molecular

orbital (MOs) images were rendered using the Chemcraft[17] program package (http://www.chemcraftprog.com) at the 0.03 contour level. Computational assessments of the conformational energetics for [**1**]$^{2+}$ were conducted at the B3PW91/LANL2DZ and B3PW91/Def2SVP levels of theory, while for [**6**]$^{1+}$, the calculations were limited to the B3PW91/LANL2DZ level.[14(a),18-20]

References

(**1**) (**a**) *APEX3*, Bruker-AXS, Inc., Madison, Wisconsin, USA, 2016. (**b**) *APEX3*, Bruker-AXS, Inc., Madison, Wisconsin, USA, 2020.

(**2**) (**a**) *SAINT*, Bruker AXS, Inc., Madison, Wisconsin, 2016. (**b**) *SAINT*, Bruker AXS, Inc., Madison, Wisconsin, 2020.

(**3**) Sheldrick, G, M. *CELL_NOW*, University of Göttingen, Göttingen, Germany, 2008.

(**4**) Sheldrick, G, M. *TWINABS*, University of Göttingen, Göttingen, Germany, 2009.

(**5**) *SADABS*, Bruker AXS, Inc., Madison, Wisconsin, 2016.

(**6**) Krause, L.; Herbst-Irmer, R.; Sheldrick, G.M.; Stalke; D. Comparison of Silver and Molybdenum Microfocus X-ray Sources for Single-Crystal Structure Determination. *J. Appl. Cryst.* **2015**, *48*, 3-10.

(**7**) Sheldrick, G. M. *SHELXT* – Integrated Space-Group and Crystal-Structure Determination. *Acta Crystallogr., Sect. A* **2015**, *71*, 3-8.

(**8**) (**a**) Sheldrick, G. M. A Short History of *SHELX*. *Acta Crystallogr., Sect. A* **2008**, *64*, 112-122. (**b**) Sheldrick, G. M. *SHELXL-2014/7*. University of Göttingen, Göttingen, Germany, 2015. (**c**) Sheldrick, G. M. *SHELXL-2018/1*. University of Göttingen, Göttingen, Germany, 2018.

(**9**) (**a**) *SHELXTL*, Bruker-AXS, Madison, WI, 2016. (**b**) *SHELXTL*, Bruker-AXS, Madison, WI, 2020.

(**10**) Fernández-García, F.; Bolaño, S.; Carballo, R.; García-Fontán, S.; Bravo, J. Reactions of [1,2-bis(diphenylphosphinite)ethane]bromotricarbonylrhenium(I) with Phosphites, Phosphonites, and Phosphinites. The Crystal Structure of [ReBr(CO)$_2${$Ph_2PO(CH_2)_2OPPh_2$}**L**'] [**L**' = $P(OCH_3)_3$, $P(OC_2H_5)_3$ and $PPh(OC_2H_5)_2$]. *Polyhedron* **2001**, *20*, 2675-2681.

(**11**) (**a**) Spek, A. L. *PLATON, A Multipurpose Crystallographic Tool*, Utrecht University, Utrecht, The Netherlands, 2015. (**b**) Spek, A. L. *PLATON SQUEEZE*: A Tool for the Calculation of the Disordered Solvent Contribution to the Calculated Structure Factors. *Acta Crystallogr., Sect. C* **2015**, *71*, 9-18.

(**12**) See http://checkcif.iucr.org/

(**13**) Gaussian 09, Revision A. 02, Frisch, M. J.; Trucks, G. W.; Schlegel, H. B.; Scuseria, G. E.; Robb, M. A.; Cheeseman, J. R.; Scalmani, G.; Barone, V.; Mennucci, B.; Petersson, G. A.; Nakatsuji, H.; Caricato, M.; Li, X.; Hratchian, H. P.; Izmaylov, A. F.; Bloino, J.; Zheng, G.; Sonnenberg, J. L.; Hada, M.; Ehara, M.; Toyota, K.; Fukuda, R.; Hasegawa, J.; Ishida, M.; Nakajima, T.; Honda, Y.; Kitao, O.; Nakai, H.; Vreven, T.; Montgomery, J. A., Jr.; Peralta, J. E.; Ogliaro, F.; Bearpark, M.; Heyd, J. J.; Brothers, E.; Kudin, K. N.; Staroverov, V. N.; Kobayashi,

R.; Normand, J.; Raghavachari, K.; Rendell, A.; Burant, J. C.; Iyengar, S. S.; Tomasi, J.; Cossi, M.; Rega, N.; Millam, J. M.; Klene, M.; Knox, J. E.; Cross, J. B.; Bakken, V.; Adamo, C.; Jaramillo, J.; Gomperts, R.; Stratmann, R. E.; Yazyev, O.; Austin, A. J.; Cammi, R.; Pomelli, C.; Ochterski, J. W.; Martin, R. L.; Morokuma, K.; Zakrzewski, V. G.; Voth, G. A.; Salvador, P.; Dannenberg, J. J.; Dapprich, S.; Daniels, A. D.; Farkas, Ö.; Foresman, J. B.; Ortiz, J. V.; Cioslowski, J.; Fox, D. J. Gaussian, Inc., Wallingford CT, 2009.

(**14**) (**a**) Becke, A. D. Density-Functional Thermochemistry. III. The Role of Exact Exchange. *J. Chem. Phys.* **1993**, *98*, 5648-5652. (**b**) Lee, C. T.; Yang, W. T.; Parr, R. G. Development of the Colle-Salvetti Correlation-Energy Formula into a Functional of the Electron Density *Phys. Rev. B* **1988**, *37*, 785-789.

(**15**) https://bse.pnl.gov/bse/portal. (Accessed April 24, 2019).

(**16**) Schäfer, A.; Horn, H.; Ahlrichs, R. Fully Optimized Contracted Gaussian Basis Sets for Atoms Li to Kr. *J. Chem. Phys.* **1992**, *97*, 2571-2577.

(**17**) Chemcraft, Version 1.8 (build 445); http://chemcraftprog.com (Accessed May 14, 2021).

(**18**) Perdew, J. P.; Wang, Y. Accurate and Simple Representation of the Electron-Gas Correlation Energy. *Phys. Rev. B* **1992**, *45*, 13244-13249.

(**19**) (**a**) Dunning, T. H., Jr.; Hay, P. J. Gaussian Basis Sets for Molecular Calculations in *Modern Theoretical Chemistry*; Schaefer, H. F., III, Ed.; Plenum: New York, 1977; Vol. 3, pp 1-28. (**b**) Hay, P. J.; Wadt, W. R. *Ab Initio* Effective Core Potentials for Molecular Calculations. Potentials for the Transition Metal Atoms Sc to Hg. *J. Chem. Phys.* **1985**, *82*, 270-283. (**c**) Wadt, W. R.; Hay, P. *Ab Initio* Effective Core Potentials for Molecular Calculations. Potentials for the Main Group Elements Na to Bi. *J. Chem. Phys.* **1985**, *82*, 284-298. (**d**) Hay, P. J.; Wadt, W. R. *Ab Initio* Effective Core Potentials for Molecular Calculations. Potentials for K to Au Including the Outermost Core Orbitals. *J. Chem. Phys.* **1985**, *82*, 299-310.

(**20**) Weigend, F. Accurate Coulomb-Fitting Basis Sets for H to Rn. *Phys. Chem. Chem. Phys.* **2006**, *8*, 1057-1065.

Table S1. Unit Cell and Refinement Data for M–Pt–M Trimetallic Compounds.

compound	[[(mnt)Ni(tpbz)]$_2$Pt][OTf]$_2$	[[(pdt)Ni(tpbz)]$_2$Pt][OTf]$_2$	[[(pdt)Pd(tpbz)]$_2$Pt][OTf]$_2$	[[(pdt)Pt(tpbz)]$_2$Pt][OTf]$_2$
compound #	**[1]**$^{2+}$	**[3]**$^{2+}$	**[4]**$^{2+}$	[**5**]$^{2+}$
structure #	JPD870	JPD1088	JPD1090	JPD973
solvent/cocrystallite	$3C_6H_5NO_2 \cdot 2Et_2O$	3DMF	$3.5DMF \cdot 2H_2O$	$4DMF \cdot 2H_2O$
formula	$C_{144}H_{119}N_7O_{14}F_6P_8S_6Ni_2Pt$	$C_{147}H_{129}N_3O_9F_6P_8S_6Ni_2Pt$	$C_{148.5}H_{128.5}N_{3.5}O_{11.5}F_6P_8S_6Pd_2Pt$	$C_{150}H_{136}N_4O_{12}F_6P_8S_6Pt_3$
fw, g/mol	3038.08	2944.12	3108.05	3326.01
temperature, K	100	150	150	150
wavelength, Å	0.71073	0.71073	0.71073	0.71073
2θ range, deg.	3.382 – 54.244	2.902 – 46.654	2.722 – 48.334	2.84 – 44.16
crystal system	triclinic	monoclinic	monoclinic	monoclinic
space group	*P*-1	$P2_1/n$	$P2_1/n$	$P2_1/n$
a, Å	13.592(3)	18.0711(13)	18.1595(15)	18.176(3)
b, Å	14.064(3)	16.1273(12)	15.9710(13)	15.956(3)
c, Å	21.188(5)	24.8605(18)	24.785(2)	24.813(4)
α, deg.	73.458(3)	90	90	90
β, deg.	89.326(3)	94.710(2)	92.570(2)	92.568(2)
γ, deg.	63.186(3)	90	90	90
volume, Å^3	3432.9(13)	7220.8(9)	7181.0(10)	7189(2)
Z	1	2	2	2
density, g/cm^3	1.470	1.354	1.437	1.537
μ, mm^{-1}	1.545	1.464	1.463	3.157
crystal size, mm	0.047 x 0.070 x 0.121	0.020 x 0.210 x 0.385	0.070 x 0.117 x 0.232	0.013 x 0.034 x 0.044
color, habit	orange plate	orange plate	red plate	red column
limiting indices, *h*	$-17 < h < 17$	$-20 < h < 20$	$-20 < h < 20$	$-19 \leq h \leq 19$
limiting indices, *k*	$-18 < k < 18$	$-17 < k < 17$	$-18 < k < 18$	$-16 \leq k \leq 16$
limiting indices, *l*	$-27 < l < 27$	$-27 < l < 27$	$-28 < l < 28$	$-26 \leq l \leq 26$
reflections collected	30509	47946	54113	34574
independent data	14975	10390	11475	8843
restraints	12	30	229	516
parameters refined	891	734	856	857
GooF^a	1.015	1.028	1.028	1.054
R1,^{b,c} wR2^{d,c}	0.0677, 0.1614	0.0761, 0.2093	0.0820, 0.2350	0.0790, 0.1671
R1,^{b,e} wR2^{d,e}	0.1041, 0.1771	0.1327, 0.2575	0.1208, 0.2798	0.1365, 0.1992
largest diff. peak, e·Å^{-3}	4.953	2.214	4.965	1.769
largest diff. hole, e·Å^{-3}	–2.316	–1.091	–2.973	–1.260
abs structure parameter	-	-	-	-

^{a}GooF = $\{\Sigma[w(F_o^2 - F_c^2)^2]/(n-p)\}^{1/2}$, where n = number of reflections and p is the total number of parameters refined; ^{b}R1 = $\Sigma||F_o| - |F_c||/\Sigma|F_o|$; ^{c}R indices for data cut off at I > 2σ(I); ^{d}wR2 = $\{\Sigma[w(F_o^2 - F_c^2)^2]/\Sigma[w(F_o^2)^2]\}^{1/2}$; $w = 1/[\sigma^2(F_o^2) + (xP)^2 + yP]$, where $P = [2F_c^2 + \mathrm{Max}(F_o^2,0)]/3$; ^{e}R indices for all data.

Table S2. Unit Cell and Refinement Data for M–Au–M Trimetallic Compounds.

compound	[[(mnt)Ni(tpbz)]$_2$Au][OTf]	[[(adt)Ni(tpbz)]$_2$Au][Cl]	[[(pdt)Ni(tpbz)]$_2$Au][OTf]	[[(pdt)Pd(tpbz)]$_2$Au][OTf]
compound #	[**6**]$^{1+}$	[**7**]$^{1+}$	[**10**]$^{1+}$	[**11**]$^{1+}$
structure #	JPD1084	JPD1100	JPD1086	JPD1114
solvent/cocrystallite	$C_6H_5NO_2 \cdot C_6H_6 \cdot {}^tBuOMe$	½DMI	2DMI·½Et_2O	-
formula	$C_{134}H_{107.5}N_{4.5}O_5F_3P_8S_5Ni_2Au$	$C_{142.5}H_{117}NO_{4.5}P_8S_4ClNi_2Au$	$C_{149}H_{129}N_4O_{5.5}F_3P_8S_5Ni_2Au$	$C_{137}H_{104}O_3F_3P_8S_5Pd_2Au$
fw, g/mol	2640.19	2641.20	2843.00	2673.02
temperature, K	150	150	150	150
wavelength, Å	0.71073	0.71073	0.71073	0.71073
2θ range, deg.	2.302 – 58.436	1.624 – 48.132	2.528 – 57.006	1.550 – 49.458
crystal system	monoclinic	trigonal	monoclinic	tetragonal
space group	*C*2	$P3_1c$	$P2_1/n$	$I4_1/a$
a, Å	36.6060(12)	28.9658(4)	16.9691(7)	40.930(3)
b, Å	20.5956(7)	28.9658(4)	26.4603(10)	40.930(3)
c, Å	21.1856(7)	35.2990(15)	32.2338(12)	34.252(4)
α, deg.	90	90	90	90
β, deg.	123.408(1)	90	92.246(1)	90
γ, deg.	90	120	90	90
volume, Å^3	13333.2(8)	25648.6(18)	14462.1(10)	57382(11)
Z	4	6	4	16
density, g/cm^3	1.315	1.026	1.306	1.238
μ, mm^{-1}	1.608	1.254	1.488	1.479
crystal size, mm	0.030 x 0.149 x 0.233	0.204 x 0.251 x 0.406	0.036 x 0.233 x 0.286	0.198 x 0.226 x 0.230
color, habit	orange plate	dark orange block	green-yellow plate	red-orange block
limiting indices, *h*	$-50 < h < 50$	$-32 < h < 32$	$-22 < h < 22$	$-48 < h < 48$
limiting indices, *k*	$-28 < k < 28$	$-32 < k < 32$	$-35 < k < 35$	$-48 < k < 48$
limiting indices, *l*	$-29 < l < 29$	$-40 < l < 40$	$-43 < l < 42$	$-40 < l < 40$
reflections collected	270886	812689	527320	849008
independent data	36107	26413	36255	24484
restraints	8	1	1029	1371
parameters refined	1357	1460	1505	1297
GooF[a]	1.066	1.050	1.159	1.100
R1,[b,c] wR2[d,c]	0.0463, 0.1036	0.0400, 0.1076	0.1198, 0.2876	0.0762, 0.1944
R1,[b,e] wR2[d,e]	0.0614, 0.1105	0.0451, 0.1122	0.1504, 0.3049	0.1210, 0.2537
largest diff. peak, e·Å^{-3}	1.165	1.224	2.840	3.535
largest diff. hole, e·Å^{-3}	–1.103	–0.391	–1.603	-1.091
abs structure parameter	0.467(5)	0.0115(18)	-	-

[a]GooF = $\{\Sigma[w(F_o^2 - F_c^2)^2]/(n - p)\}^{1/2}$, where *n* = number of reflections and *p* is the total number of parameters refined; [b]R1 = $\Sigma||F_o| - |F_c||/\Sigma|F_o|$; [c]R indices for data cut off at $I > 2\sigma(I)$; [d]wR2 = $\{\Sigma[w(F_o^2 - F_c^2)^2]/\Sigma[w(F_o^2)^2]\}^{1/2}$; $w = 1/[\sigma^2(F_o^2) + (xP)^2 + yP]$, where $P = [2F_c^2 + \mathrm{Max}(F_o^2,0)]/3$; [e]R indices for all data.

Table S3. Unit Cell and Refinement Data for M–ReBr(CO)$_3$ Bimetallic Compounds.

compound	[(mnt)Ni(tpbz)ReBr(CO)$_3$]	[(mnt)Ni(tpbz)ReBr(CO)$_3$]	[(pdt)Pd(tpbz)ReBr(CO)$_3$]	[(pdt)Pt(tpbz)ReBr(CO)$_3$]
compound #	**13**	**13**	**14**	**15**
structure #	JPD979	JPD1046	JPD1130	JPD1133
solvent/cocrystallite	Et_2O	-	-	-
formula	$C_{65}H_{52}N_2O_4P_4S_2NiBrRe$	$C_{61}H_{42}BrN_2NiO_3P_4ReS_2$	$C_{71}H_{52}O_3P_4S_2BrPdRe$	$C_{71}H_{52}O_3P_4S_2BrRePt$
fw, g/mol	1437.90	1363.78	1513.63	1602.32
temperature, K	150	150	150	150
wavelength, Å	0.71073	0.71073	0.71073	0.71073
2θ range, deg.	3.374 – 54.026	3.480 – 59.052	4.510 – 48.304	4.124 – 49.682
crystal system	triclinic	triclinic	triclinic	triclinic
space group	*P*-1	*P*-1	*P*-1	*P*-1
a, Å	12.485(1)	12.2669(7)	12.4836(8)	12.5072(16)
b, Å	14.3945(11)	14.3287(8)	13.6154(9)	13.6271(18)
c, Å	18.1824(14)	19.9983(11)	22.4715(15)	22.613(3)
α, deg.	80.245(1)	87.473(1)	93.405(1)	93.476(4)
β, deg.	75.687(1)	87.403(1)	103.368(1)	103.521(4)
γ, deg.	83.856(1)	79.214(1)	114.643(1)	114.768(4)
volume, $Å^3$	3113.2(4)	3447.2(3)	3324.7(4)	3347.3(8)
Z	2	2	2	2
density, g/cm^3	1.534	1.314	1.512	1.590
μ, mm^{-1}	3.104	2.799	2.894	4.693
crystal size. mm	0.144 x 0.195 x 0.370	0.211 x 0.266 x 0.285	0.023 x 0.074 x 0.198	0.045 x 0.085 x 0.217
color, habit	orange block	orange block	pale brown needle	orange column
limiting indices, *h*	-15 < *h* < 15	-16 < *h* < 16	-14 < *h* < 14	-14 < *h* < 14
limiting indices, *k*	-18 < *k* < 18	-19 < *k* < 19	-15 < *k* < 15	-16 < *k* < 16
limiting indices, *l*	-23 < *l* < 23	-27 < *l* < 27	-25 < *l* < 25	0 < *l* < 26
reflections collected	52460	67814	24695	24705
independent data	13368	18544	10584	24705
restraints	0	112	0	101
parameters refined	656	652	748	746
GooF[a]	1.080	1.055	1.009	1.016
R1,[b,c] wR2[d,c]	0.0453, 0.1337	0.0458, 0.1301	0.0482, 0.1013	0.0833, 0.2131
R1,[b,e] wR2[d,e]	0.0538, 0.1396	0.0602, 0.1371	0.0795, 0.1113	0.1191, 0.2449
largest diff. peak, e·$Å^{-3}$	2.463	1.915	1.841	2.876
largest diff. hole, e·$Å^{-3}$	–1.465	–1.936	–1.198	–3.225
abs structure parameter	-	-	-	-

[a]GooF = $\{\Sigma[w(F_o^2 - F_c^2)^2]/(n - p)\}^{1/2}$, where *n* = number of reflections and *p* is the total number of parameters refined; [b]R1 = $\Sigma||F_o| - |F_c||/\Sigma|F_o|$; [c]R indices for data cut off at I > 2σ(I); [d]wR2 = $\{\Sigma[w(F_o^2 - F_c^2)^2]/\Sigma[w(F_o^2)^2]\}^{1/2}$; $w = 1/[\sigma^2(F_o^2) + (xP)^2 + yP]$, where $P = [2F_c^2 + \mathrm{Max}(F_o^2,0)]/3$; [e]R indices for all data.

Table S4. Unit Cell and Refinement Data for M–Re–M Trimetallic Compounds.

compound	[[(mnt)Ni(tpbz)]$_2$ReBr(CO)]	[[(mnt)Ni(tpbz)]$_2$Re(CO)$_2$][Br]	[[(pdt)Pt(tpbz)]$_2$ReBr(CO)]	[[(pdt)Pt(tpbz)]$_2$Re(CO)$_2$][Br]
compound #	**16**	[**17**]$^{1+}$	**18**	[**19**]$^{1+}$
structure #	JPD1079	JPD1028	JPD1107	JPD1077
solvent/cocrystallite	2DMI·2Et_2O[a]	-	4$PhNO_2$	3$PhNO_2$·^{t}BuOMe
formula	$C_{117}H_{84}N_4OP_8S_4Ni_2BrRe$	$C_{118}H_{84}N_4O_2P_8S_4Ni_2BrRe$	$C_{161}H_{124}N_4O_9P_8S_4BrRePt_2$	$C_{161}H_{114}N_3O_9P_8S_4BrRePt_2$
fw, g/mol	2321.41	2349.42	3290.92	3266.83
temperature, K	200	150	150	200
wavelength, Å	0.71073	0.71073	0.71073	0.71073
2θ range, deg.	2.692 – 44.070	2.740 – 28.538	4.010 – 53.102	2.584 – 56.732
crystal system	monoclinic	monoclinic	monoclinic	monoclinic
space group	$P2_1/n$	$P2_1/n$	$P2_1/c$	$P2_1/c$
a, Å	14.0537(10)	13.924(3)	14.2385(7)	14.0974(14)
b, Å	24.4013(17)	23.264(5)	21.6579(11)	21.564(2)
c, Å	20.3377(14)	19.354(4)	23.3488(12)	23.225(2)
α, deg.	90	90	90	90
β, deg.	108.650(2)	92.803(2)	96.601(2)	96.407
γ, deg.	90	90	90	90
volume, Å^3	6608.1(8)	6262(2)	7152.5(6)	7016.4(12)
Z	2	2	2	2
density, g/cm^3	1.167	1.246	1.528	1.546
μ, mm^{-1}	1.700	1.795	3.283	3.346
crystal size, mm	0.118 x 0.159 x 0.255	0.047 x 0.089 x 0.341	0.043 x 0.197 x 0.240	0.045 x 0.059 x 0.212
color, habit	orange plate	orange-brown column	orange plate	red-orange column
limiting indices, *h*	$-14 < h < 14$	$-9 < h < 9$	$-16 < h < 17$	$-18 < h < 18$
limiting indices, *k*	$-25 < k < 25$	$-16 < k < 16$	$-27 < k < 27$	$-28 < k < 28$
limiting indices, *l*	$-21 < l < 21$	$-13 < l < 13$	$-29 < l < 29$	$-30 < l < 31$
reflections collected	41069	11192	228634	69920
independent data	8112	2180	14769	17463
restraints	0	3	618	39
parameters refined	365	217	853	715
GooF[b]	1.011	1.072	1.118	1.009
R1,[c,d] wR2[e,d]	0.0716, 0.2030	0.1056, 0.2776	0.0701, 0.1558	0.0789, 0.1596
R1,[c,f] wR2[e,f]	0.1068, 0.2363	0.1265, 0.3023	0.1028, 0.1786	0.2013, 0.2086
largest diff. peak, e·Å^{-3}	1.635	1.639	4.520	2.625
largest diff. hole, e·Å^{-3}	–1.236	–2.642	–1.864	–1.710
abs structure parameter	-	-	-	-

[a]Solvent removed using SQUEEZE and not factored into formula, fw, or density; [b]GooF = $\{\Sigma[w(F_o^2 - F_c^2)^2]/(n - p)\}^{1/2}$, where *n* = number of reflections and *p* is the total number of parameters refined; [c]R1 = $\Sigma||F_o| - |F_c||/\Sigma|F_o|$; [d]R indices for data cut off at I > 2σ(I); [e]wR2 = $\{\Sigma[w(F_o^2 - F_c^2)^2]/\Sigma[w(F_o^2)^2]\}^{1/2}$; $w = 1/[\sigma^2(F_o^2) + (xP)^2 + yP]$, where $P = [2F_c^2 + \mathrm{Max}(F_o^2,0)]/3$; [f]R indices for all data.

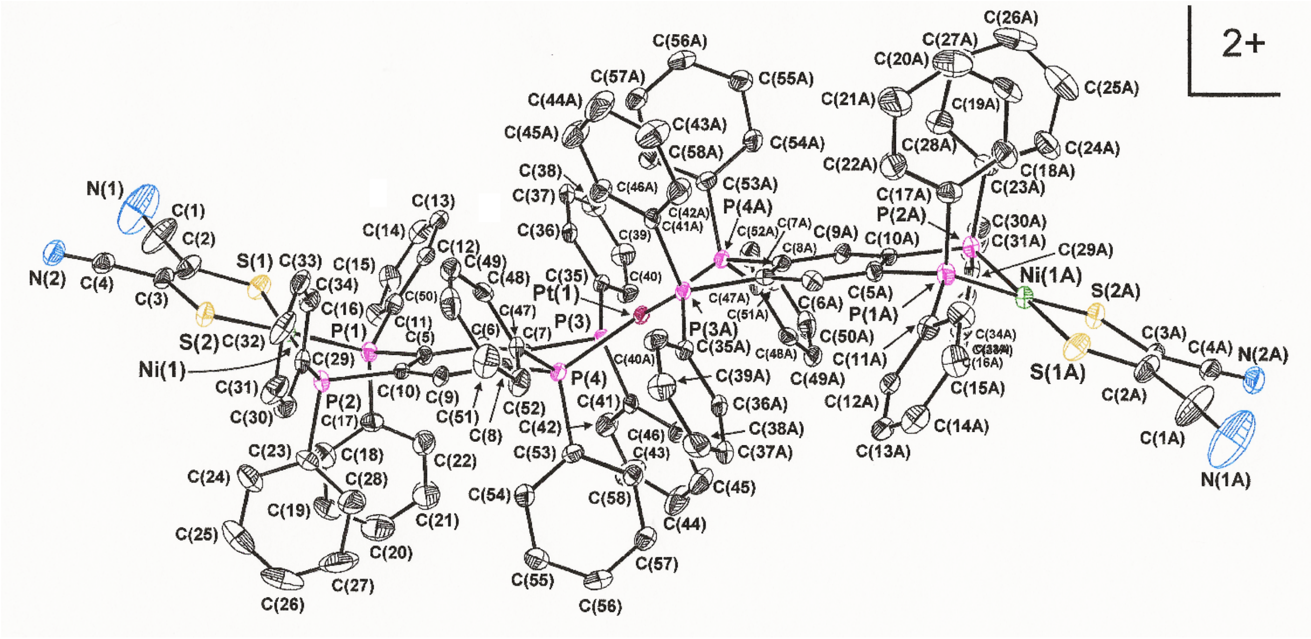

Figure S1. Thermal ellipsoid plot (50% level) of [(mnt)Ni(μ_2-tpbz)Pt(μ_2-tpbz)Ni(mnt)]$^{2+}$ cation, [**1**]$^{2+}$, with complete atom labeling. All hydrogen atoms are omitted for clarity. The cation resides on an inversion center that is coincident with Pt(1).

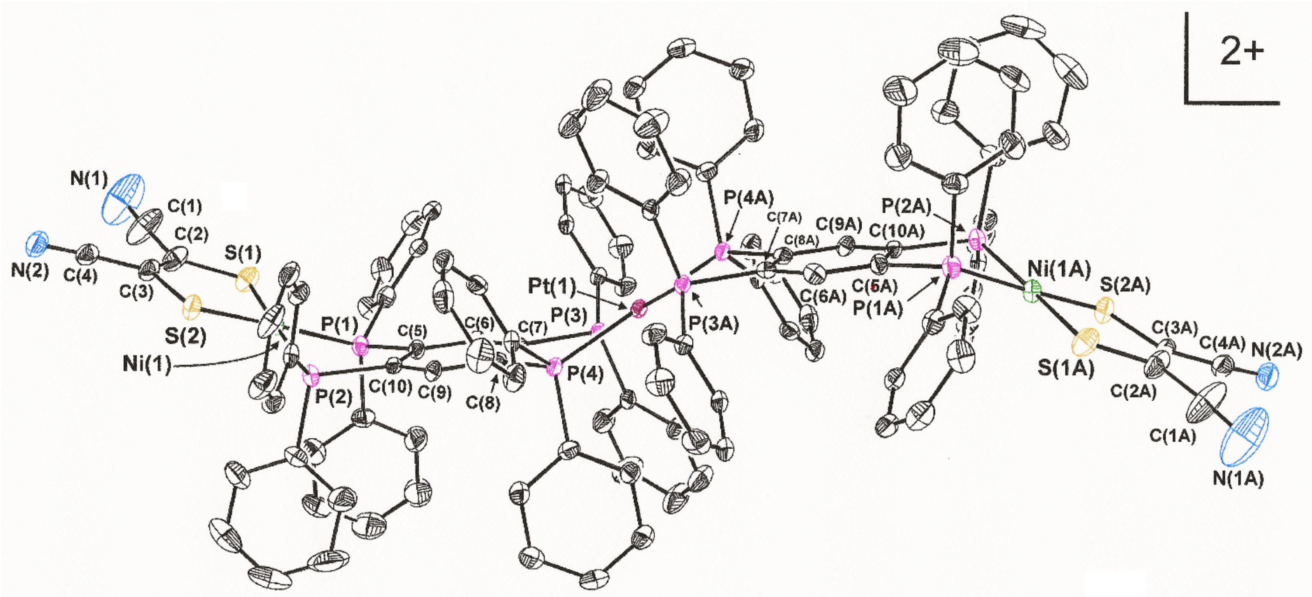

Figure S2. Thermal ellipsoid plot (50% level) of [(mnt)Ni(μ_2-tpbz)Pt(μ_2-tpbz)Ni(mnt)]$^{2+}$ cation, [**1**]$^{2+}$, with partial atom labeling. All hydrogen atoms are omitted for clarity. The cation resides on an inversion center that is coincident with Pt(1).

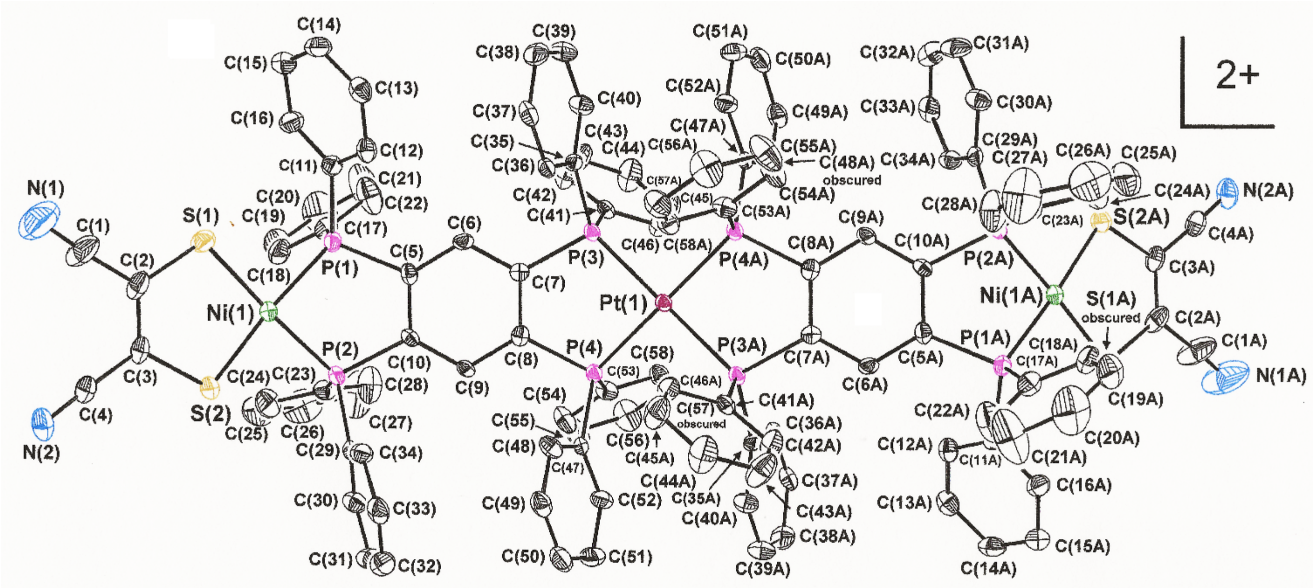

Figure S3. Thermal ellipsoid plot (50% level) of [(mnt)Ni(μ_2-tpbz)Pt(μ_2-tpbz)Ni(mnt)]$^{2+}$ cation, [**1**]$^{2+}$, with complete atom labeling. In this view, the cation is rotated forward 90° relative to the view in **Figure S1**. All hydrogen atoms are omitted for clarity. The cation resides on an inversion center that is coincident with Pt(1).

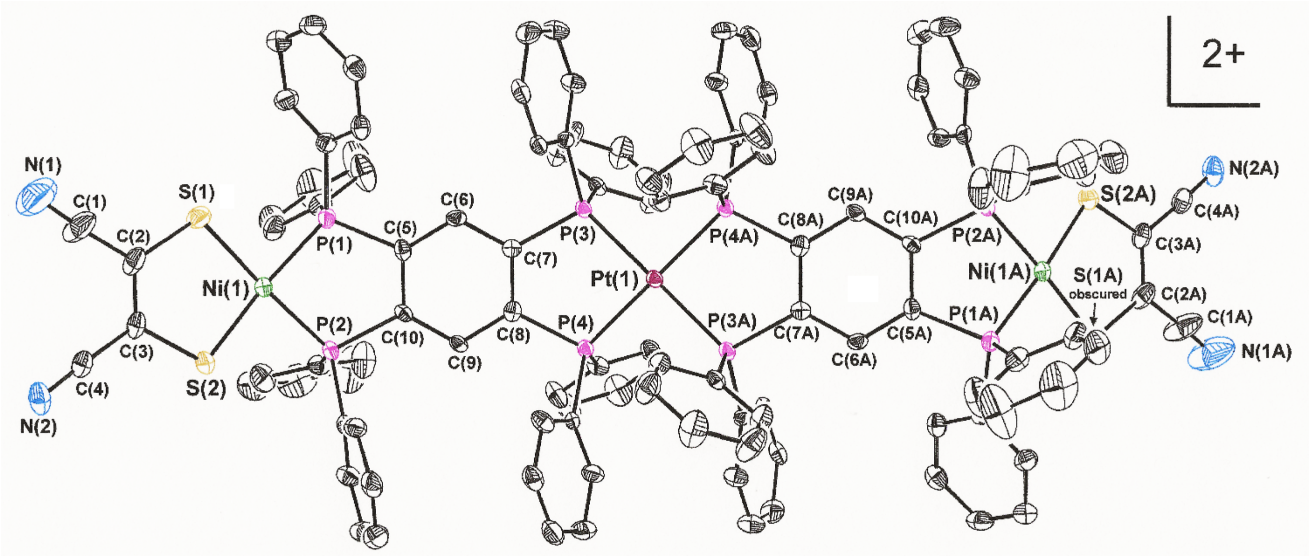

Figure S4. Thermal ellipsoid plot (50% level) of [(mnt)Ni(μ_2-tpbz)Pt(μ_2-tpbz)Ni(mnt)]$^{2+}$ cation, [**1**]$^{2+}$, with partial atom labeling. In this view, the cation is rotated forward 90° relative to the view in **Figure S2**. All hydrogen atoms are omitted for clarity. The cation resides on an inversion center that is coincident with Pt(1).

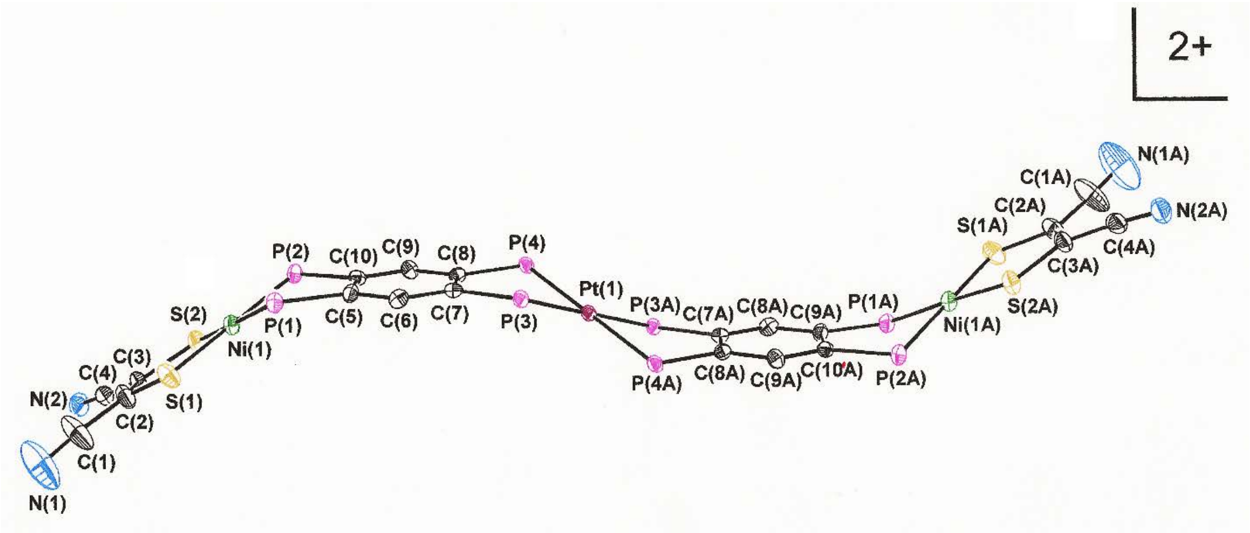

Figure S5. Core topology of the [(mnt)Ni(μ_2-tpbz)Pt(μ_2-tpbz)Ni(mnt)]$^{2+}$ cation, [**1**]$^{2+}$, with atom labeling and with ellipsoids drawn at the 50% level. Hydrogen atoms and phenyl groups are omitted for clarity.

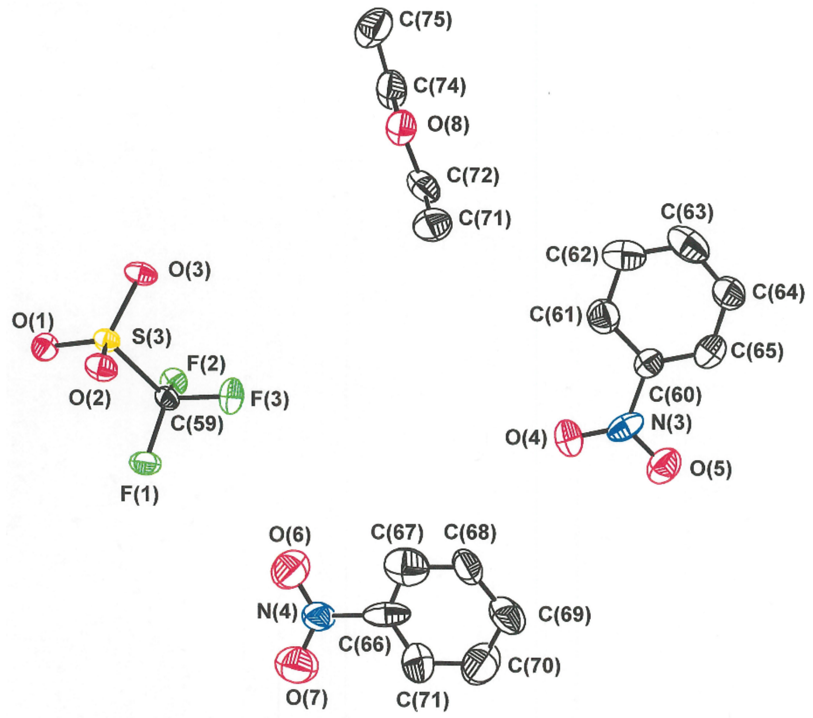

Figure S6. Atom labeling for $[CF_3SO_3]^{1-}$ counteranion and for interstitial solvent in [(mnt)Ni(μ_2-tpbz)Pt(μ_2-tpbz)Ni(mnt)]$[CF_3SO_3]_2$·$3C_6H_5NO_2$·$2Et_2O$ The thermal ellipsoids are presented at the 50% level. Hydrogen atoms are omitted for clarity.

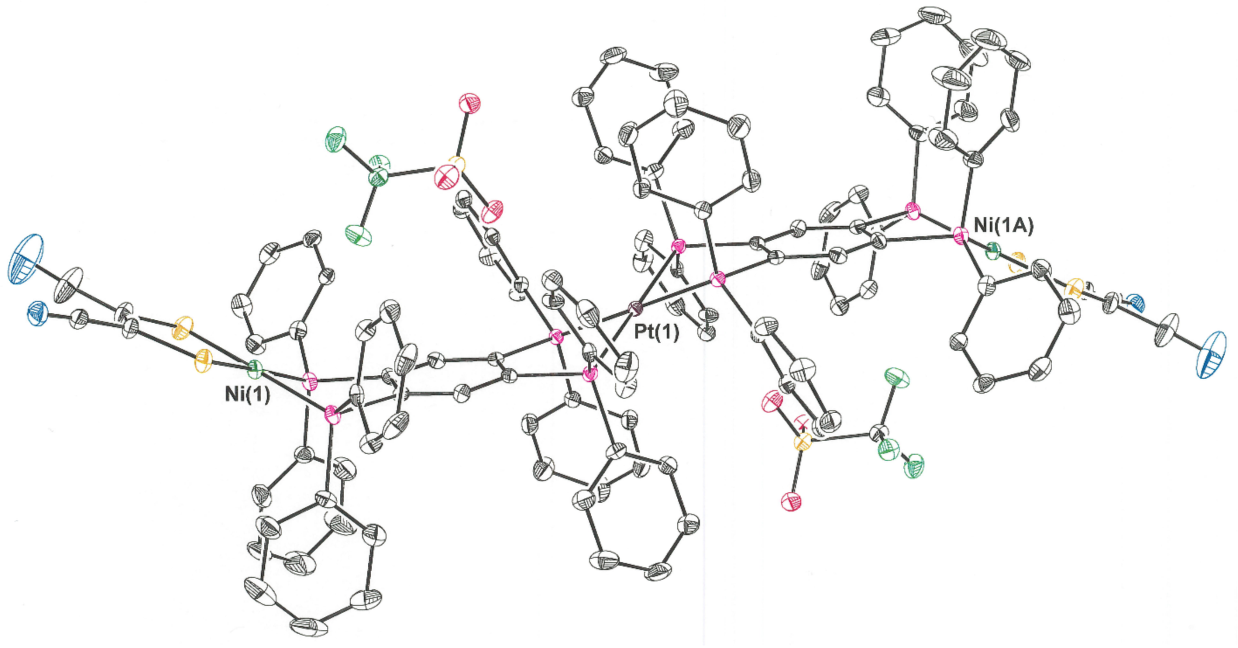

Figure S7. Illustration of the packing of [(mnt)Ni(μ_2-tpbz)Pt(μ_2-tpbz)Ni(mnt)]$^{2+}$ cation, [**1**]$^{2+}$ with its [CF_3SO_3]$^{1-}$ counteranions in the cavities at top left and bottom right. Ellipsoids are drawn at the 40% level. Hydrogen atoms and phenyl groups are omitted for clarity.

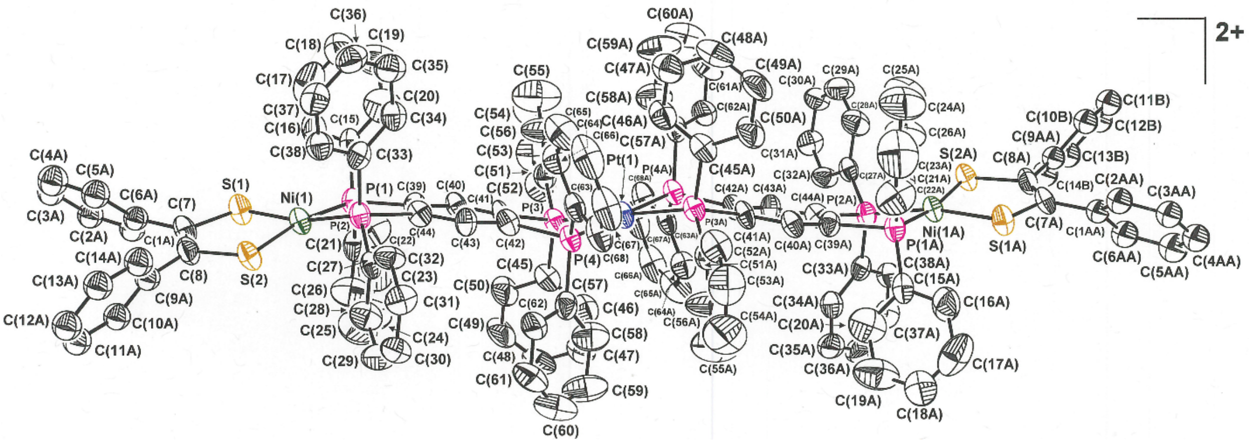

Figure S8. Thermal ellipsoid plot of [(pdt)Ni(μ_2-tpbz)Pt(μ_2-tpbz)Ni(pdt)]$^{2+}$, [**3**]$^{2+}$, drawn at the 50% probability level with all H atoms omitted for clarity. The center of the cation, occupied by the Pt(2+) cation, is coincident with an inversion center such that only half of it is structurally unique. The dithiolene phenyl rings are each disordered over two positions, only one of which is shown. The alternate positions bear the atom labels C(1B) → C(6B) and C(9B) → C(14B).

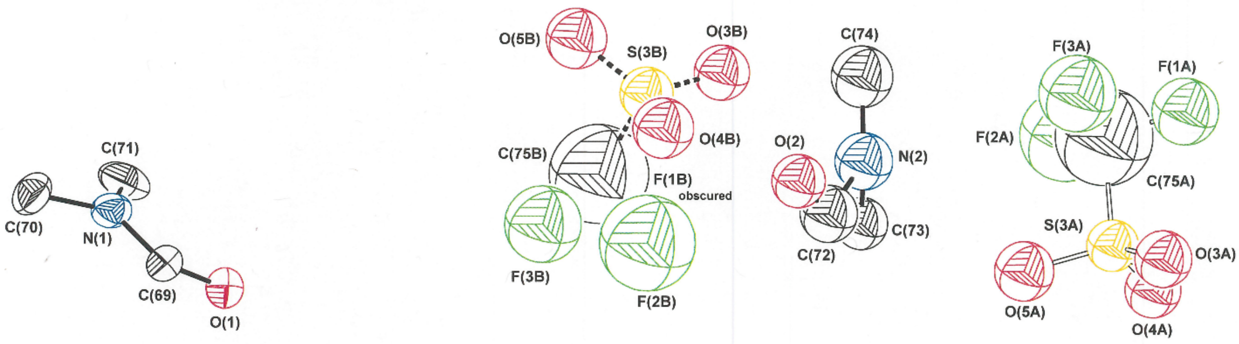

Figure S9. Triflate counteranions and interstitial solvent in [**3**][CF_3SO_3]$_2$·3DMF. The thermal ellipsoid plot is drawn at the 30% probability level, and all H atoms are omitted for clarity. The atoms of the highly disordered triflate anion were restrained to chemically reasonable bonding and nonbonding distances.

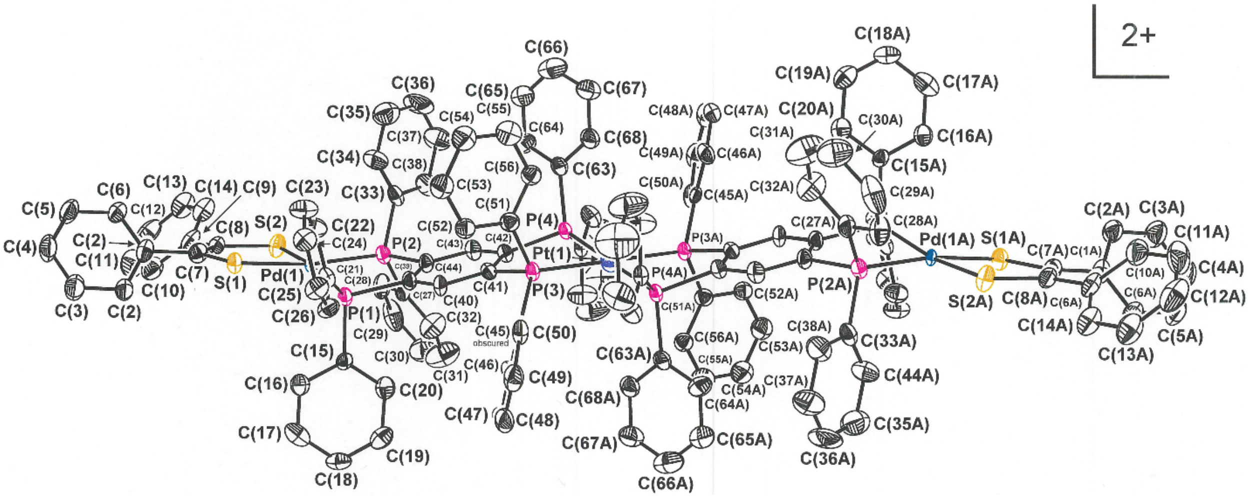

Figure S10. Thermal ellipsoid plot of [(pdt)Pd(μ_2-tpbz)Pt(μ_2-tpbz)Pd(pdt)]$^{2+}$, [**4**]$^{2+}$, drawn at the 40% probability level. All H atoms are omitted for clarity. The Pt^{2+} ion resides on an inversion center. The Ph ring defined by C(51)-C(56) is disordered over two positions, only one of which is shown.

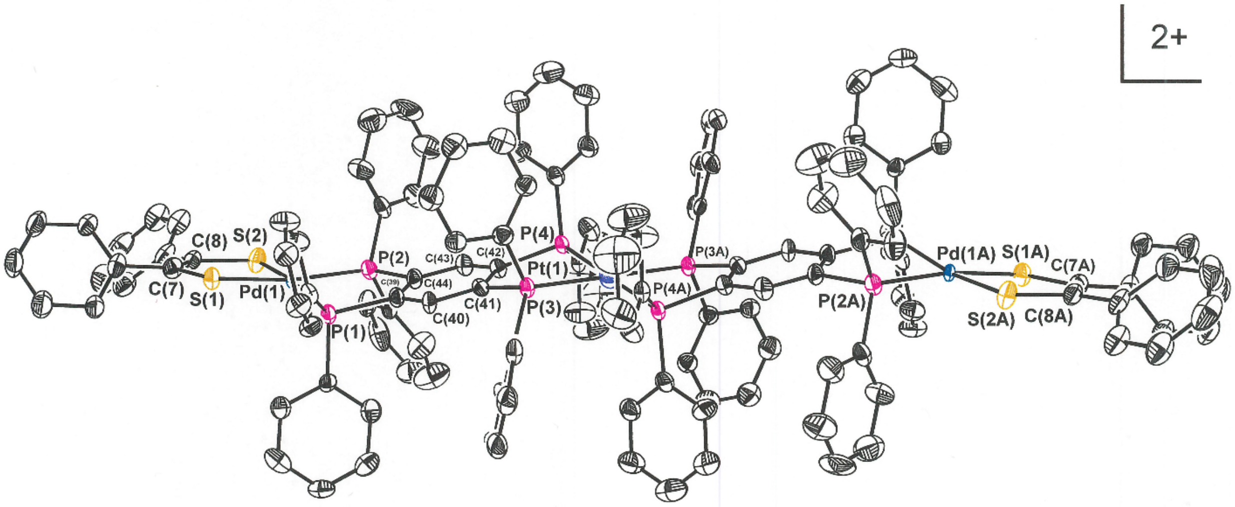

Figure S11. Partial atom labeling for [**4**]$^{2+}$. The thermal ellipsoid plot is drawn at the 40% probability level with all H atoms omitted for clarity. The Pt^{2+} ion resides on an inversion center.

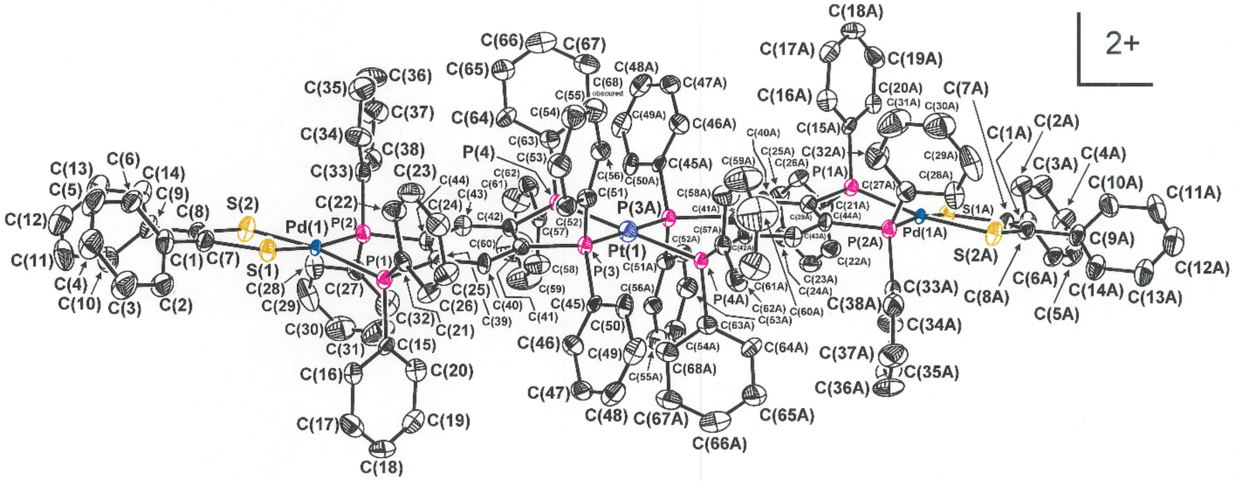

Figure S12. Alternate view of [**4**]$^{2+}$ with complete atom labeling. The thermal ellipsoid plot is drawn at the 40% probability level with all H atoms omitted for clarity. The Pt^{2+} ion resides on an inversion center.

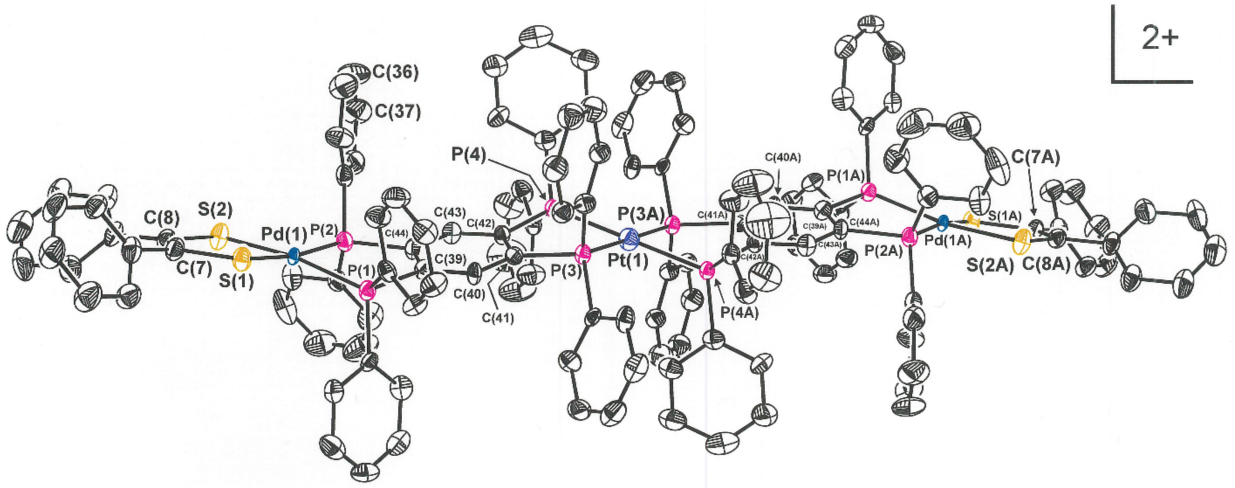

Figure S13. Alternate view of [**4**]$^{2+}$ with partial atom labeling. The thermal ellipsoid plot is drawn at the 40% probability level with all H atoms omitted for clarity. The Pt^{2+} ion resides on an inversion center.

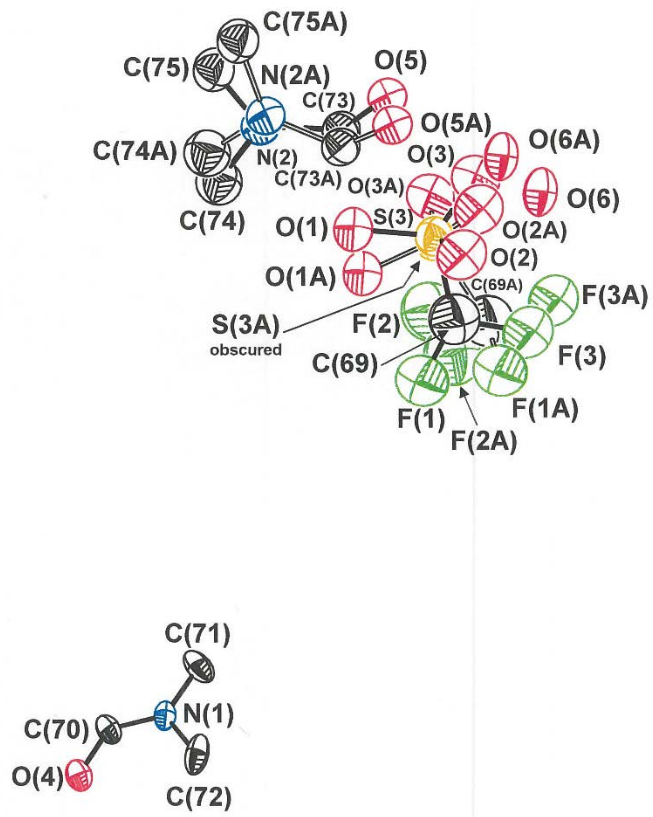

Figure S14. Atom labeling for OTf^{1-} counteranion and interstitial solvent in [(pdt)Pd(μ_2-tpbz)Pt(μ_2-tpbz)Pt(pdt)][OTf]$_2$·3.5(DMF)·2(H_2O). The thermal ellipsoid plot is drawn at the 30% probability level. All H atoms are omitted for clarity.

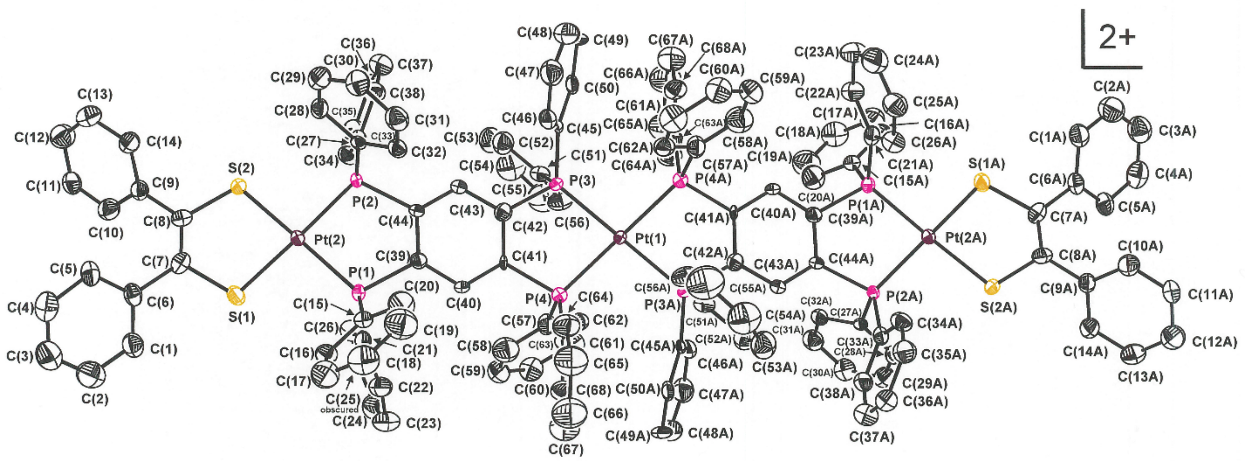

Figure S15. Thermal ellipsoid plot (50% level) of the [(pdt)Pt(μ_2-tpbz)Pt(μ_2-tpbz)Pt(pdt)]$^{2+}$ cation, [**5**]$^{2+}$, with complete atom labeling. All hydrogen atoms are omitted for clarity. The cation resides on an inversion center that is coincident with Pt(1).

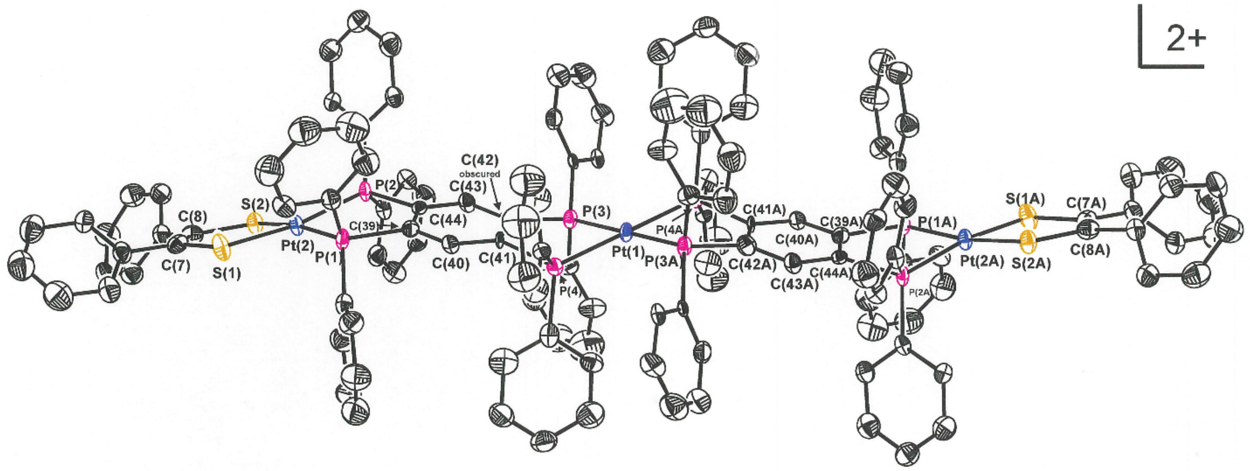

Figure S16. Thermal ellipsoid plot (50% level) of [(pdt)Pt(μ_2-tpbz)Pt(μ_2-tpbz)Pt(pdt)]$^{2+}$ cation, [**5**]$^{2+}$, with partial atom labeling. All hydrogen atoms are omitted for clarity. The cation resides on an inversion center that is coincident with Pt(1).

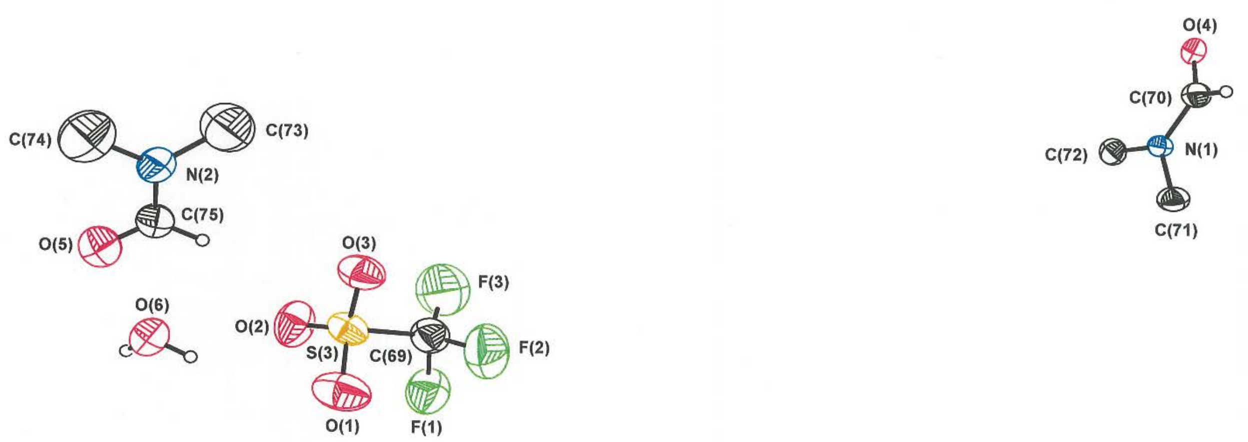

Figure S17. Atom labeling for $[CF_3SO_3]^{1-}$ counteranion and for interstitial solvent in [(pdt)Pt(μ_2-tpbz)Pt(μ_2-tpbz)Pt(pdt)]$[CF_3SO_3]_2 \cdot 4DMF \cdot 2H_2O$. The thermal ellipsoids are presented at the 50% level. Hydrogen atoms are omitted for clarity.

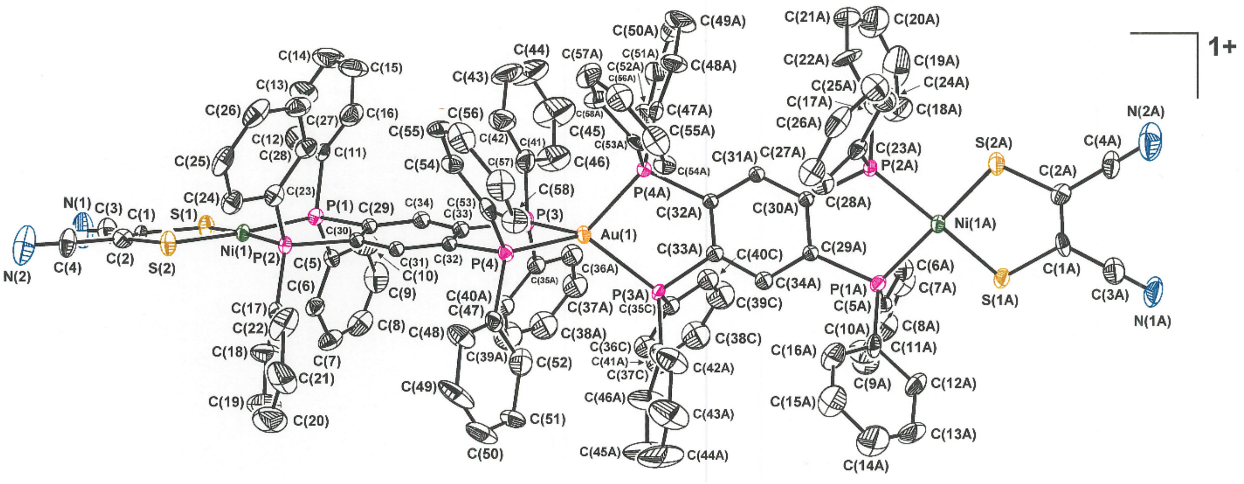

Figure S18. Atom labeling for cation 1 of [(mnt)Ni(μ_2-tpbz)Au(μ_2-tpbz)(mnt)]$^{1+}$, [**6**]$^{1+}$. The thermal ellipsoid plot is drawn at the 50% probability level, and all H atoms are omitted for clarity. The center of the cation, occupied by the Au(1+) cation, is coincident with a C_2 axis such that only half of it is structurally unique. One phenyl ring is disordered over two positions, only one of which is shown. The alternate position bears the atom labels C(35B) → C(40B).

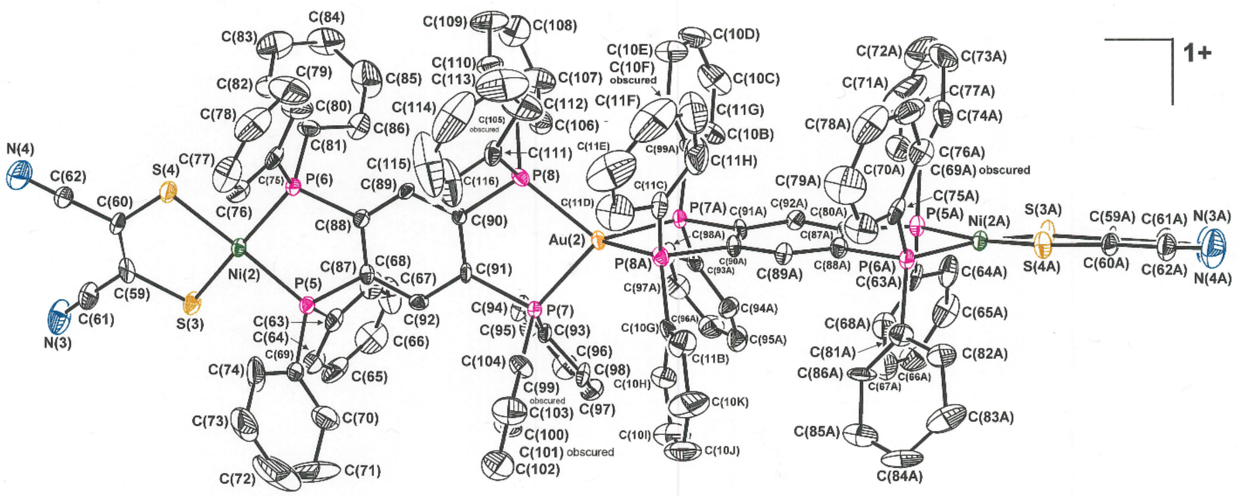

Figure S19. Atom labeling for cation 2 of [(mnt)Ni(μ_2-tpbz)Au(μ_2-tpbz)(mnt)]$^{1+}$, [**6**]$^{1+}$. The thermal ellipsoid plot is drawn at the 50% probability level, and all H atoms are omitted for clarity. The center of the cation, occupied by the Au(1+) cation, is coincident with a C_2 axis such that only half of it is structurally unique.

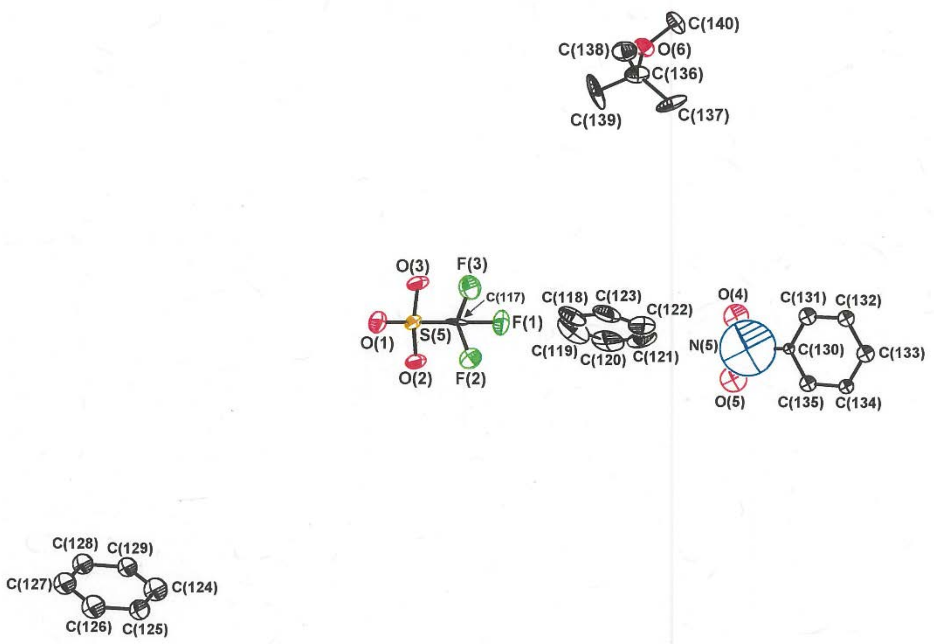

Figure S20. Atom labeling for $[CF_3SO_3]^{1-}$ counteranion and for interstitial solvent in [(mnt)Ni(μ_2-tpbz)Au(μ_2-tpbz)Ni(mnt)][CF_3SO_3]·½$C_6H_5NO_2$·C_6H_6·^{t}BuOMe. The thermal ellipsoids are presented at the 30% level. Hydrogen atoms are omitted for clarity.

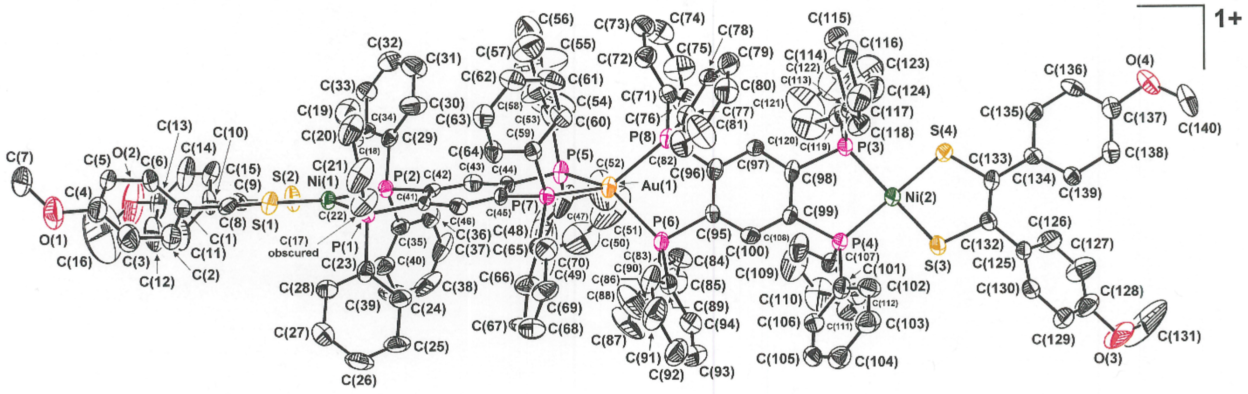

Figure S21. Atom labeling for [(adt)Ni(μ_2-tpbz)Au(μ_2-tpbz)Ni(adt)]$^{1+}$, [**7**]$^{1+}$. The thermal ellipsoid plot is drawn at the 50% probability level, and all H atoms are omitted for clarity.

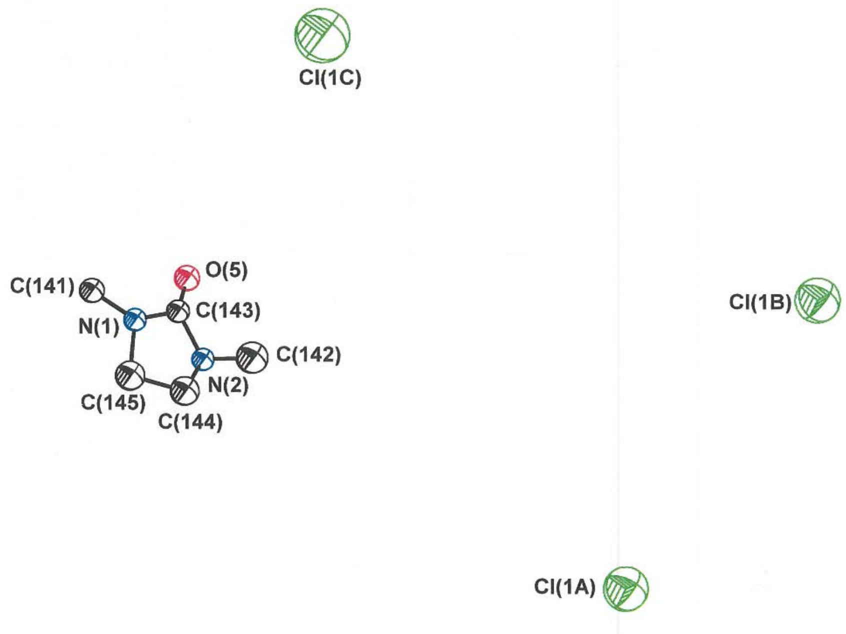

Figure S22. Atom labeling for [Cl]$^{1-}$ counteranion and for interstitial solvent in [(adt)Ni(μ_2-tpbz)Au(μ_2-tpbz)Ni(adt)][Cl]·½(DMI). The thermal ellipsoids are presented at the 30% level. Hydrogen atoms are omitted for clarity.

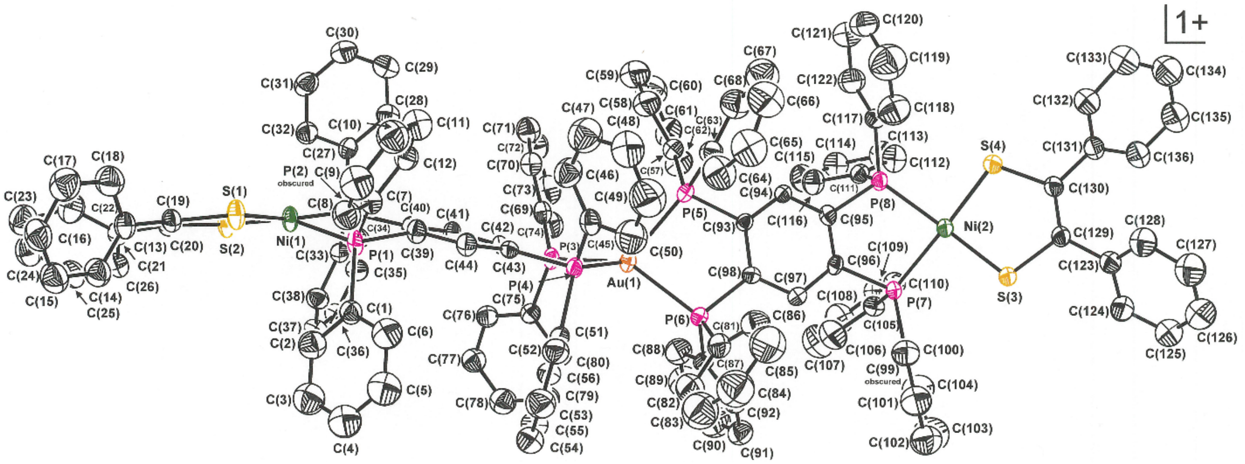

Figure S23. Thermal ellipsoid plot (30% level) of [(pdt)Ni(μ_2-tpbz)Au(μ_2-tpbz)Ni(pdt)]$^{1+}$ cation, [**10**]$^{1+}$, with complete atom labeling. All hydrogen atoms are omitted for clarity.

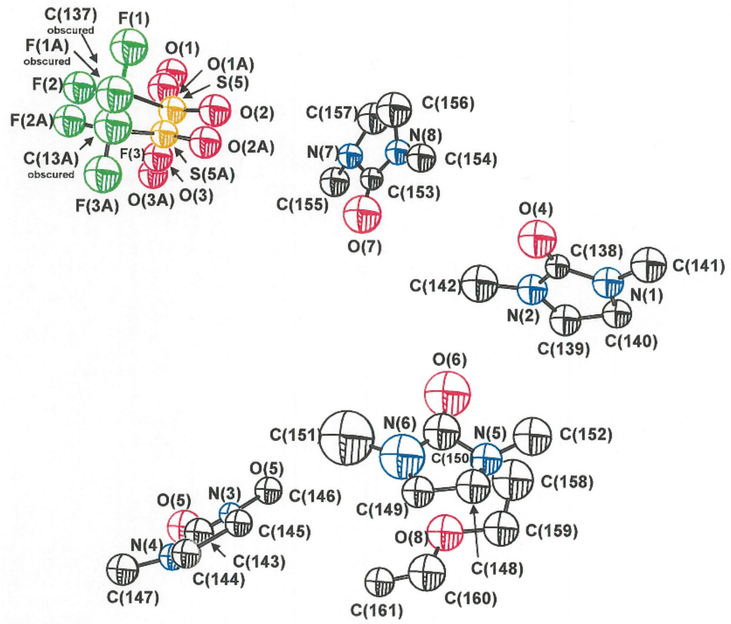

Figure S24. Atom labeling for [CF_3SO_3]$^{1-}$ counteranion and for interstitial solvent in [(pdt)Ni(μ_2-tpbz)Au(μ_2-tpbz)Ni(pdt)][CF_3SO_3]·2(1,3-Me_2-2-imidazolidinone)·½Et_2O. The isotropic thermal ellipsoids are presented at the 30% level. Hydrogen atoms are omitted for clarity.

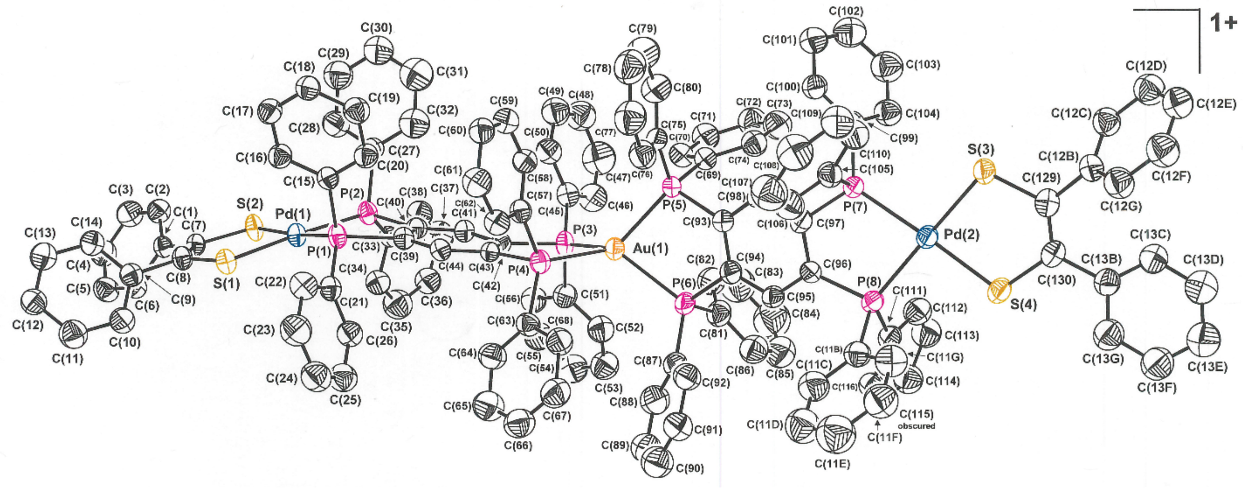

Figure S25. Atom labeling for [(pdt)Pd(μ_2-tpbz)Au(μ_2-tpbz)Pd(pdt)]$^{1+}$, [**11**]$^{1+}$. The thermal ellipsoid plot is drawn at the 40% probability level, and all H atoms are omitted for clarity. Most of the phenyl rings are disordered over two positions, only one of which is shown.

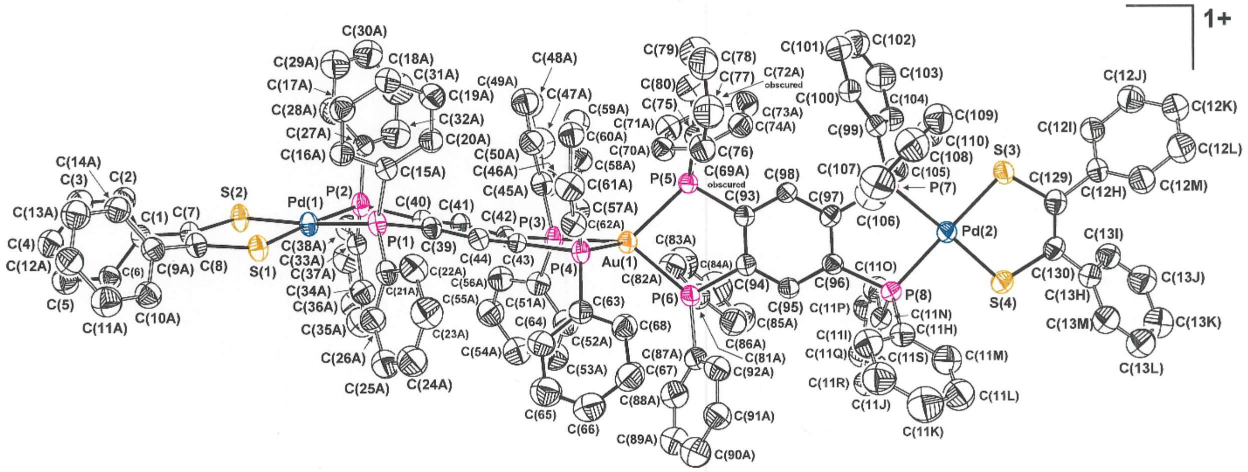

Figure S26. Atom labeling for [(pdt)Pd(μ_2-tpbz)Au(μ_2-tpbz)Pd(pdt)]$^{1+}$, [**11**]$^{1+}$. The thermal ellipsoid plot is drawn at the 40% probability level, and all H atoms are omitted for clarity. Those phenyl groups that are disordered are shown in their second positions, relative to what is presented in **Figure S25**, with complete atom labeling.

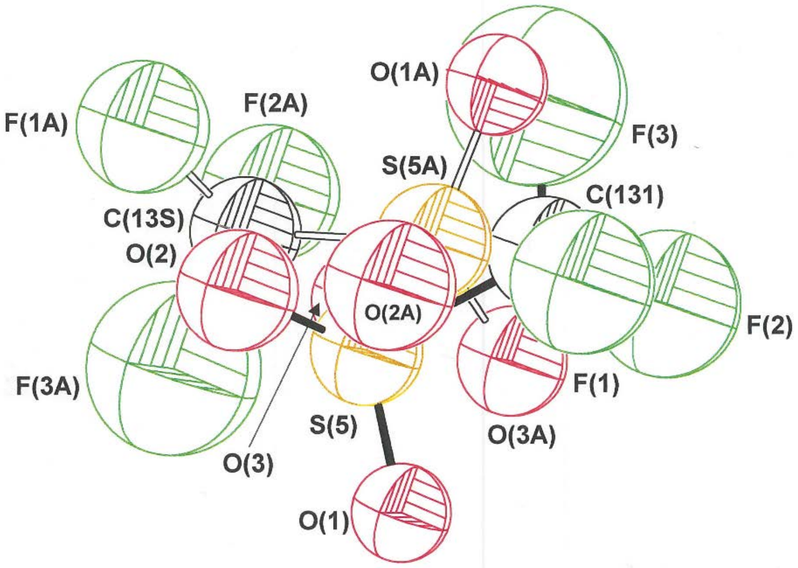

Figure S27. Atom labeling for disordered OTf^{1-} anion in [($Ph_2C_2S_2$)Pd(tpbz)Au(tpbz)Pd($S_2C_2Ph_2$)][OTf], [**11**][CF_3SO_3]. The thermal ellipsoid plot is presented at the 30% level.

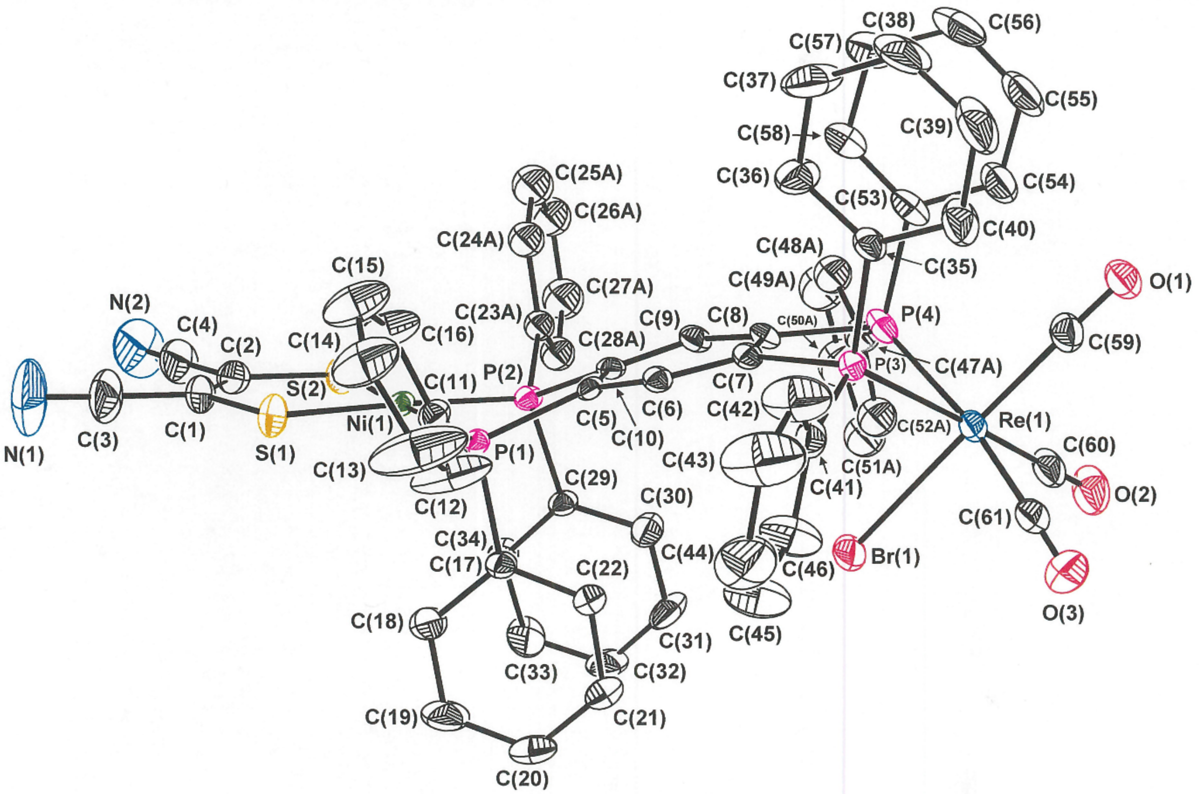

Figure S28. Complete atom labeling for [(mnt)Ni(μ_2-tpbz)ReBr(CO)$_3$], **13**, pseudopolymorph 1 (JPD979). The thermal ellipsoid plot is drawn at the 40% level, and all H atoms are omitted for clarity. The Ph rings defined by C23 → C28 and C47 → C52 are disordered over two positions, only one of which is shown.

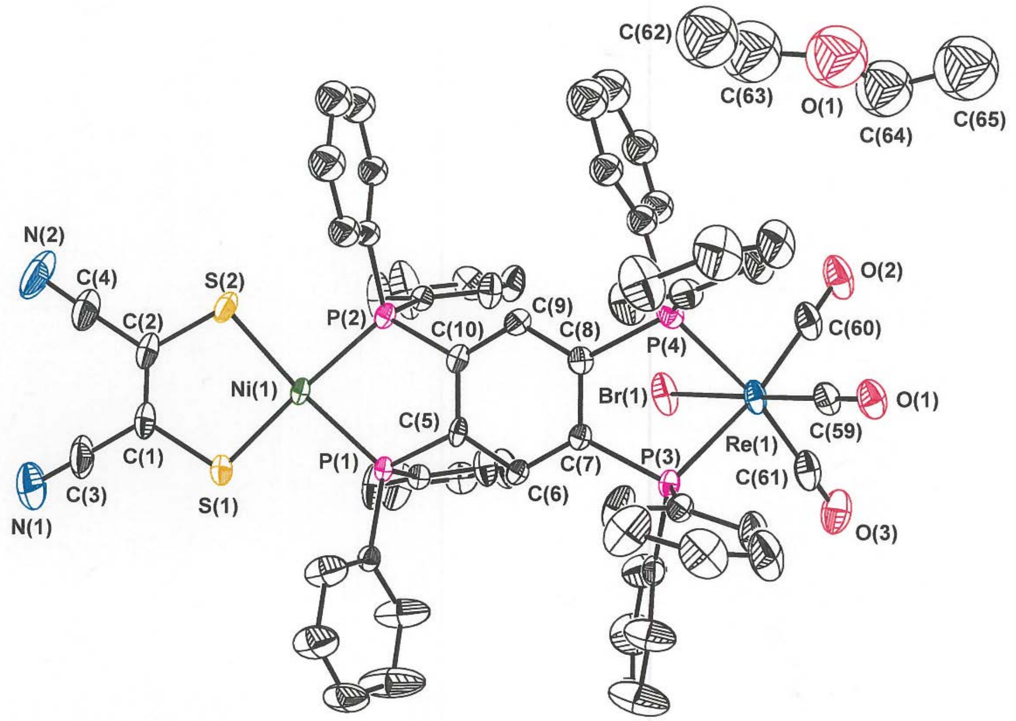

Figure S29. Partial atom labeling for [(mnt)Ni(μ_2-tpbz)ReBr(CO)$_3$], **13**, pseudopolymorph 1 (JPD979) and for interstitial Et_2O solvent molecule. The thermal ellipsoid plot is drawn at the 40% level, and all H atoms are omitted for clarity.

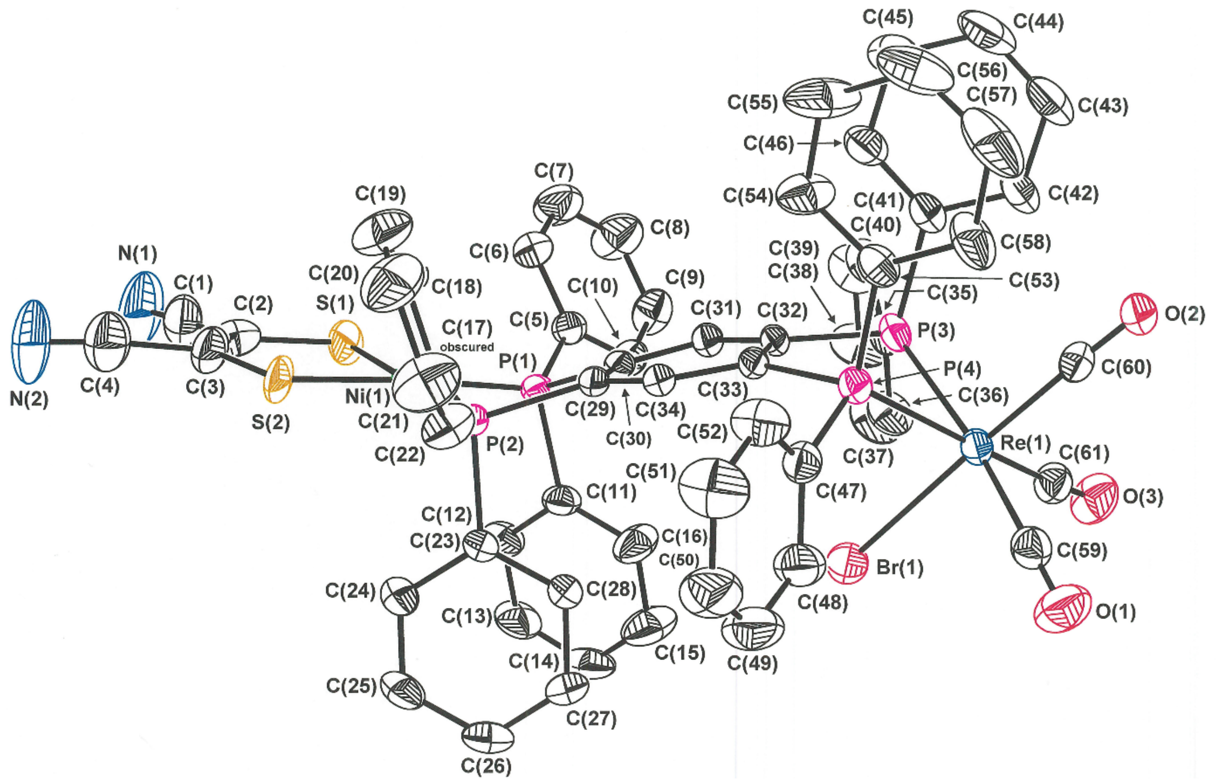

Figure S30. Complete atom labeling for [(mnt)Ni(μ_2-tpbz)ReBr(CO)$_3$], **13**, pseudopolymorph 2 (JPD1046). The thermal ellipsoid plot is drawn at the 40% level, and all H atoms are omitted for clarity.

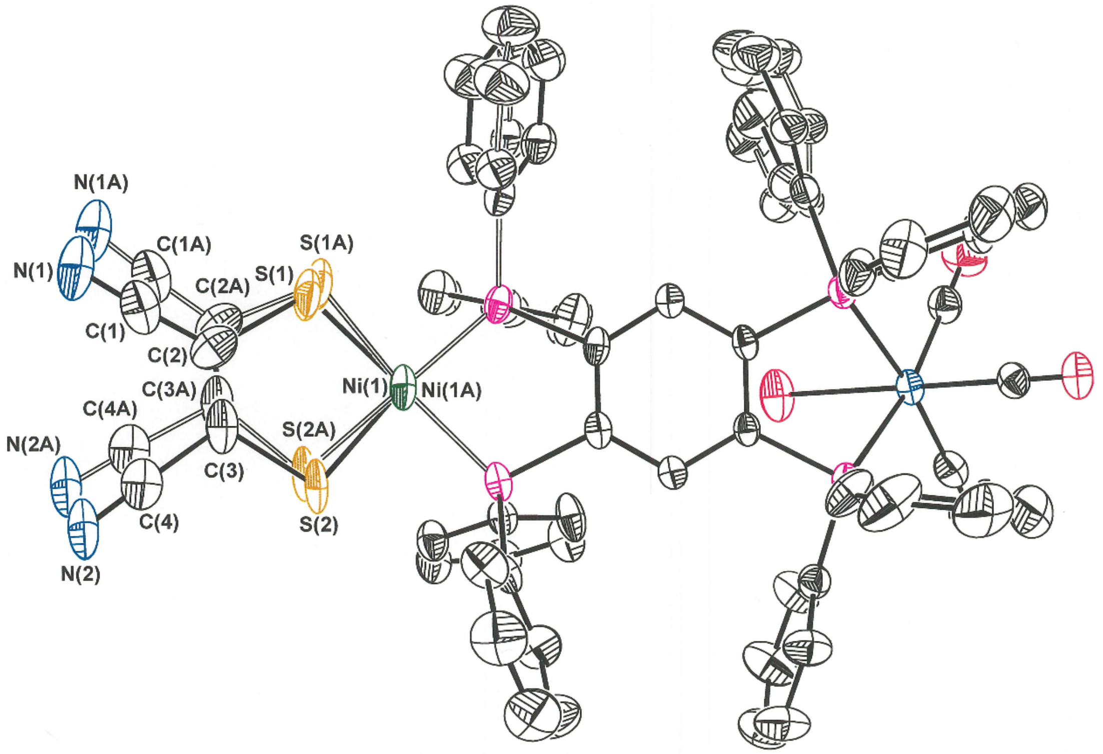

Figure S31. View of the disordered fragments in [(mnt)Ni(tpbz)ReBr(CO)$_3$] (pseudopolymorph 2, JPD1046) with 40% ellipsoids. The phenyl rings at top, encompassing carbon atoms C(5)→C(10) and C(35)→C(40) occur as two positional variants.

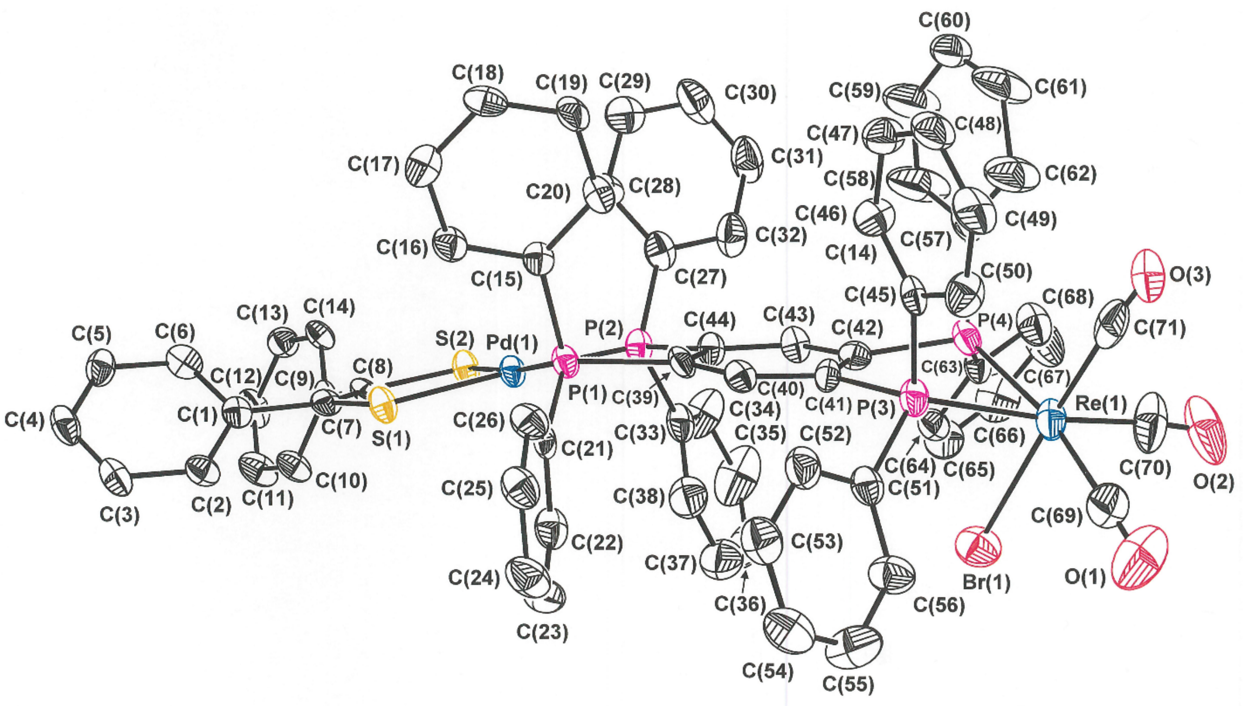

Figure S32. Complete atom labeling for [(pdt)Pd(μ_2-tpbz)ReBr(CO)$_3$], **14**. The thermal ellipsoid plot is drawn at the 50% level, and all H atoms are omitted for clarity.

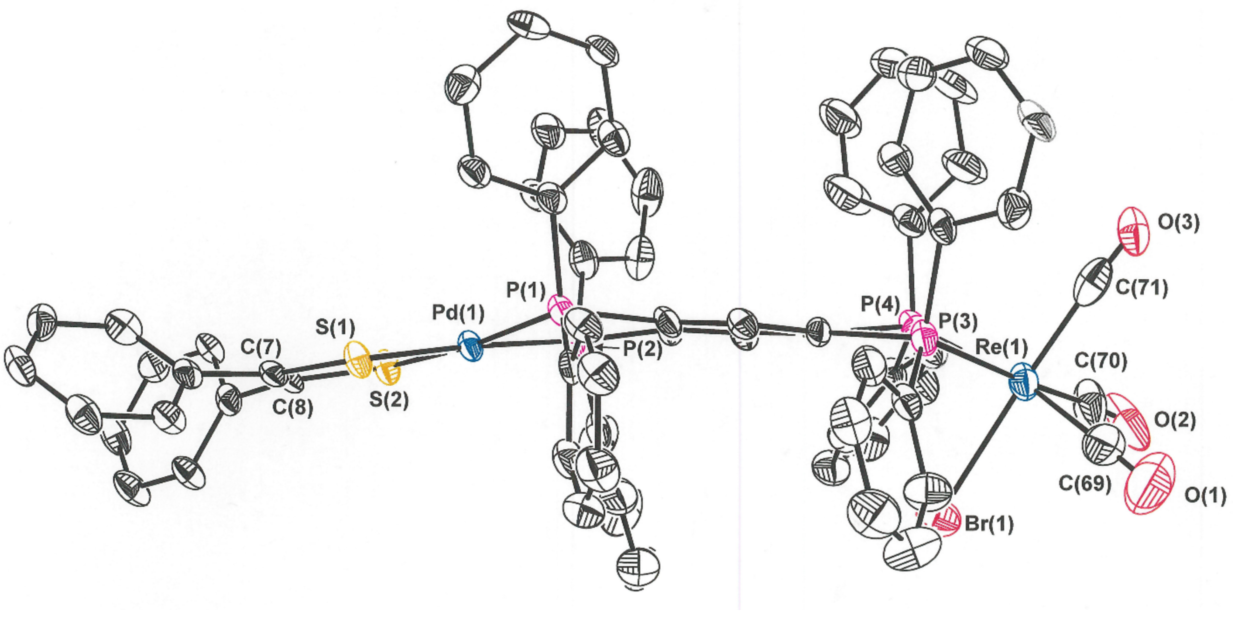

Figure S33. Alternate view for [(pdt)Pd(μ_2-tpbz)ReBr(CO)$_3$], **14**, with partial atom labeling. The thermal ellipsoid plot is drawn at the 50% level, and all H atoms are omitted for clarity.

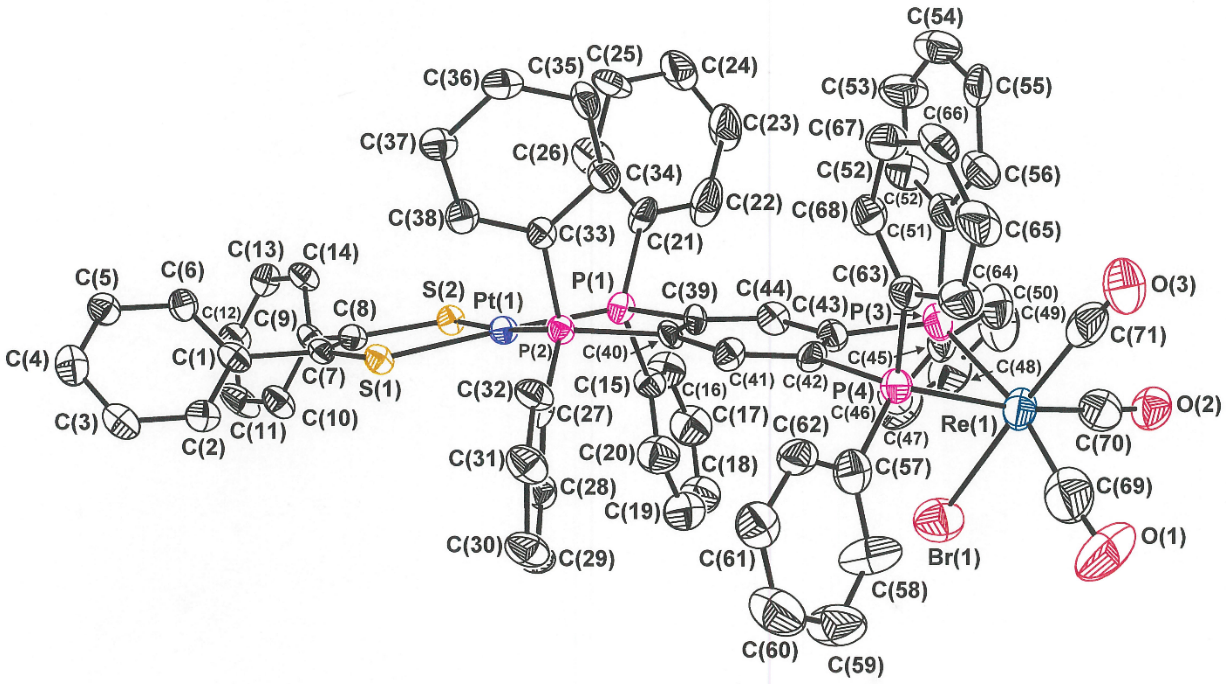

Figure S34. Complete atom labeling for [(pdt)Pt(μ_2-tpbz)ReBr(CO)$_3$], **15**. The thermal ellipsoid plot is drawn at the 40% level, and all H atoms are omitted for clarity. The phenyl rings defined by C(15)-C(20 and C(51-C(56) are each disordered over two positions, only one of which is shown. Similarly, the carbonyl ligand defined by C(70)-O(2) is disordered, and only one of its positional variants is presented.

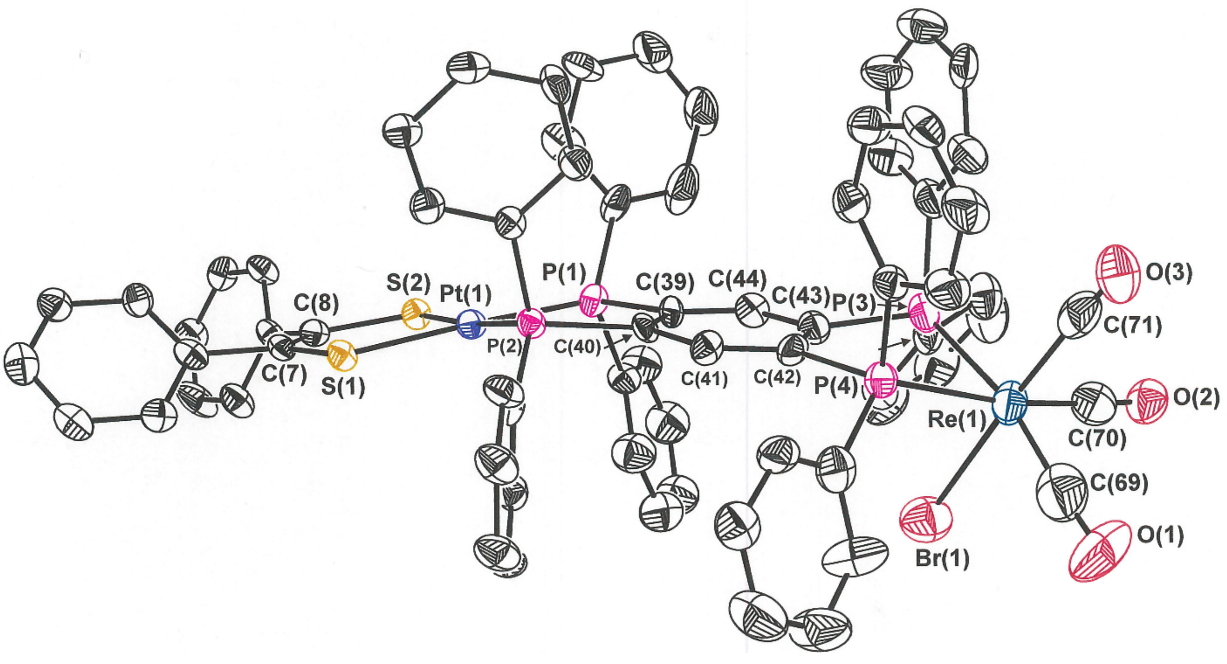

Figure S35. View of [(pdt)Pt(μ_2-tpbz)ReBr(CO)$_3$], **15**, with partial atom labeling. The thermal ellipsoid plot is drawn at the 40% level, and all H atoms are omitted for clarity. The carbonyl ligand defined by C(70)-O(2) is disordered, and only one of its positional variants is presented.

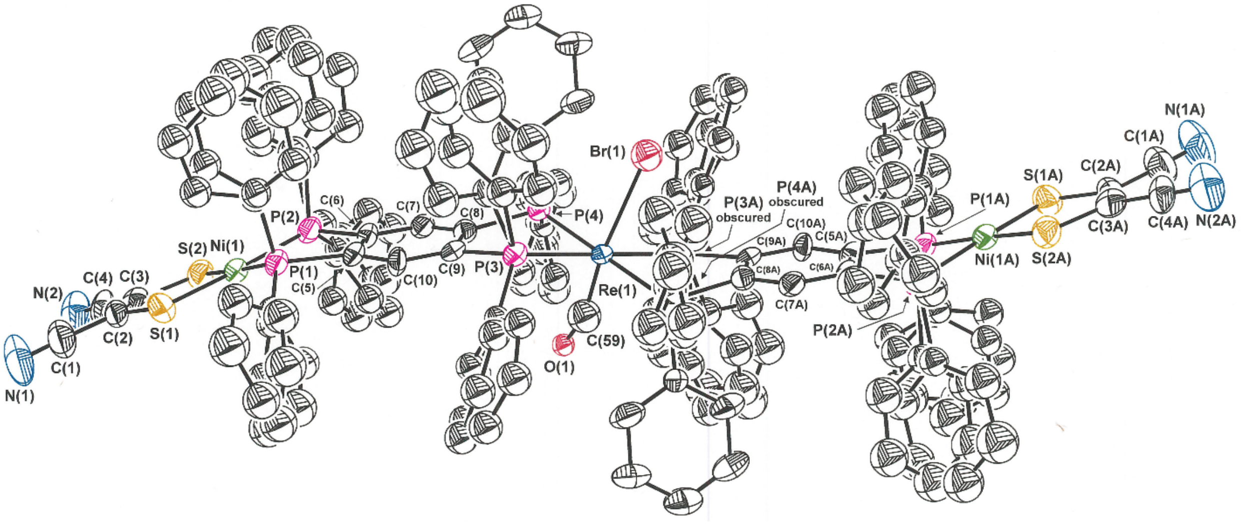

Figure S36. Side view with partial atom labeling for [(mnt)Ni(μ_2-tpbz)ReBr(CO)(μ_2-tpbz)Ni(mnt)], **16**. The thermal ellipsoid plot is drawn at the 30% probability level, and all H atoms are omitted for clarity. The center of the molecule, occupied by the Re(1+) cation, is coincident with an inversion center such that only half of it is structurally unique. All of the tpbz phenyl rings but one are disordered over two positions. The Br(1-) and CO ligands are disordered across the inversion center. The atoms of the CO ligand had to be restrained to chemically reasonable positions.

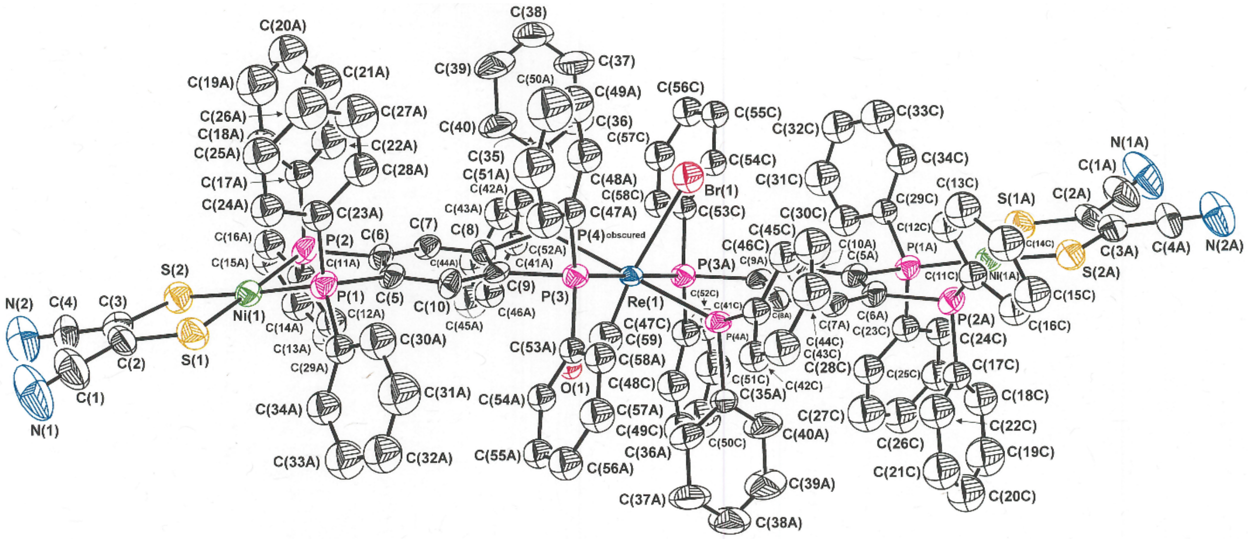

Figure S37. Side view with complete atom labeling for [(mnt)Ni(μ_2-tpbz)ReBr(CO)(μ_2-tpbz)Ni(mnt)], **16**. Thermal ellipsoid plot is drawn at the 30% probability level, and all H atoms are omitted for clarity. The center of the molecule, occupied by the Re(1+) cation, is coincident with an inversion center such that only half of it is structurally unique. All of the tpbz phenyl rings but one are disordered over two positions. Only one of the two orientational variants for each disordered phenyl ring is shown.

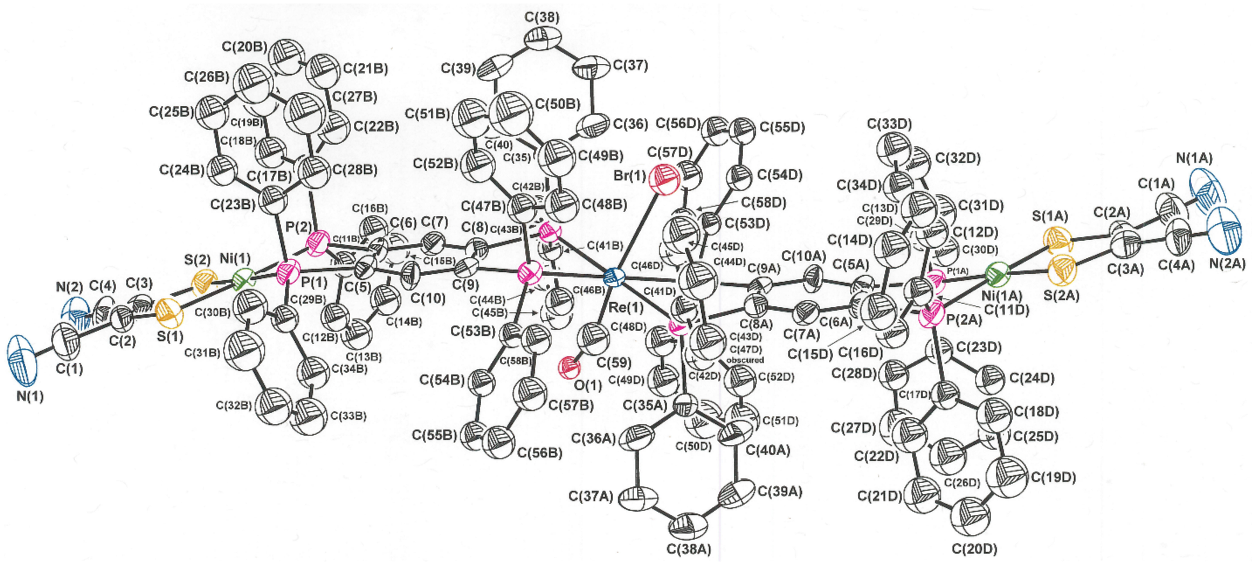

Figure S38. Alternate side view with complete atom labeling for [(mnt)Ni(μ_2-tpbz)ReBr(CO)(μ_2-tpbz)Ni(mnt)], **16**. The thermal ellipsoid plot is drawn at the 30% probability level, and all H atoms are omitted for clarity. The center of the molecule, occupied by the Re(1+) cation, is coincident with an inversion center such that only half of it is structurally unique. All of the tpbz phenyl rings but one are disordered over two positions. Only one of the two orientational variants for each disordered phenyl ring is shown.

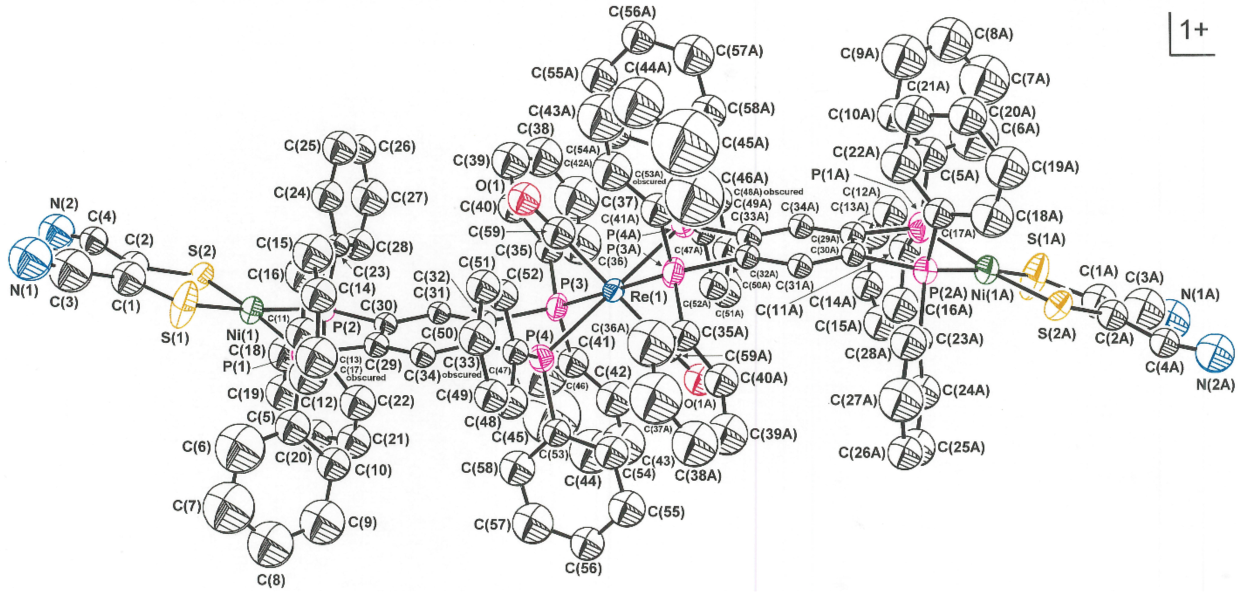

Figure S39. Side view with complete atom labeling for [(mnt)Ni(μ_2-tpbz)Re(CO)$_2$(μ_2-tpbz)Ni(mnt)]$^+$, [**17**]$^{1+}$. The thermal ellipsoid plot is drawn at the 30% probability level, and all H atoms are omitted for clarity. The center of the cation, occupied by the Re(1+) cation, is coincident with an inversion center such that only half of it is structurally unique. Owing to the limited quality of the data, only Re, Ni, Br, S and P could be treated anisotropically.

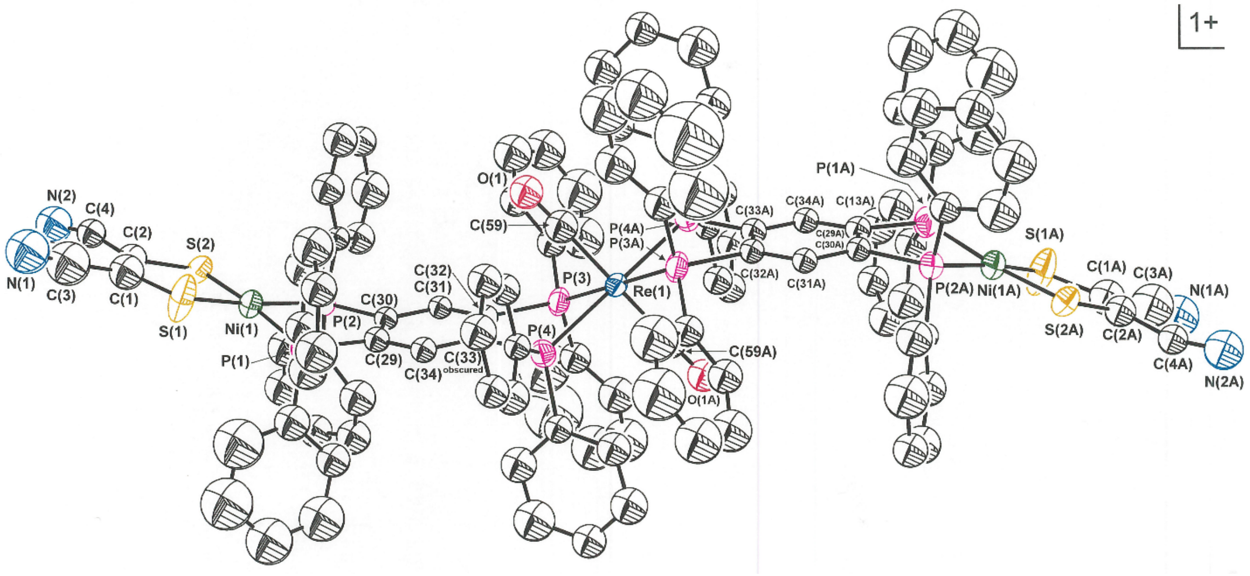

Figure S40. Side view with partial atom labeling for [(mnt)Ni(μ_2-tpbz)Re(CO)$_2$(μ_2-tpbz)Ni(mnt)]$^+$, [**17**]$^{1+}$. The thermal ellipsoid plot is drawn at the 30% probability level, and all H atoms are omitted for clarity. The center of the cation, occupied by the Re(1+) cation, is coincident with an inversion center such that only half of it is structurally unique. Owing to the limited quality of the data, only Re, Ni, Br, S and P could be treated anisotropically.

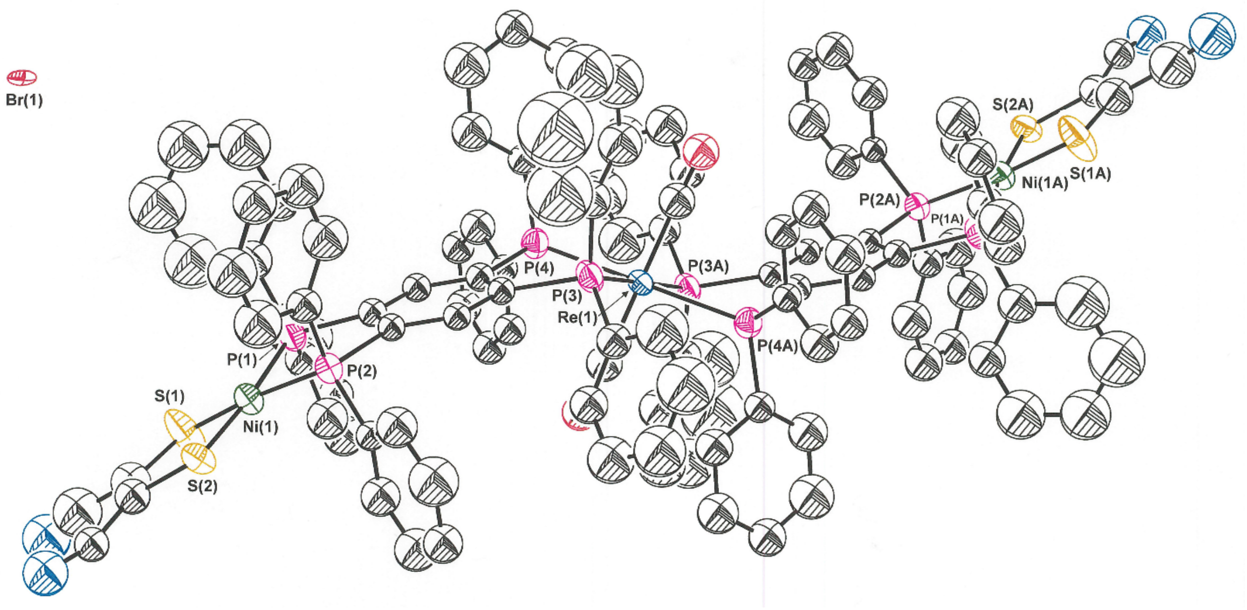

Figure S41. Side view with partial atom labeling for [(mnt)Ni(μ_2-tpbz)Re(CO)$_2$(μ_2-tpbz)Ni(mnt)][Br], [**17**][Br]. The thermal ellipsoid plot is drawn at the 30% probability level, and all H atoms are omitted for clarity. The center of the cation, occupied by the Re(1+) cation, is coincident with an inversion center such that only half of it is structurally unique. Owing to the limited quality of the data, only Re, Ni, Br, S and P could be treated anisotropically.

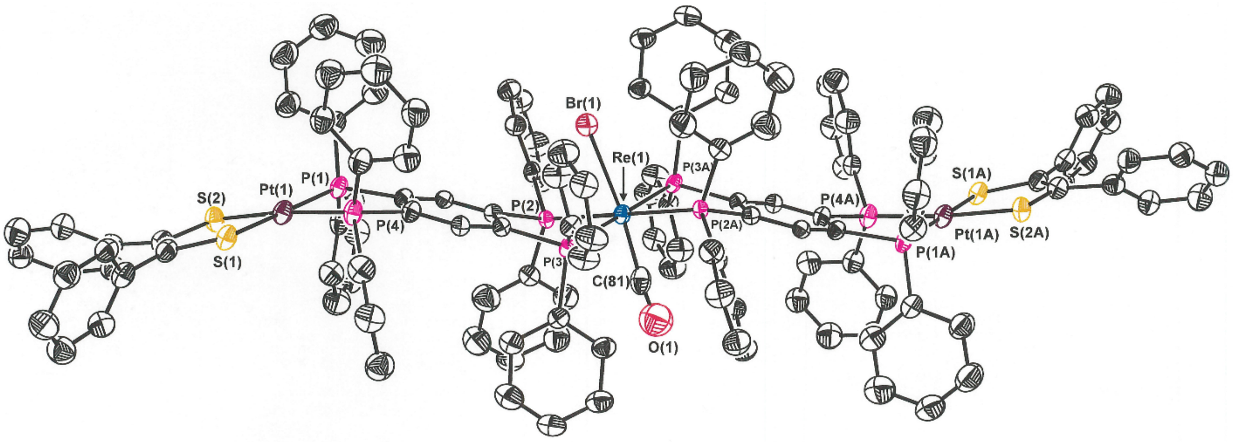

Figure S42. Thermal ellipsoid plot (35% level) of [(pdt)Pt(μ_2-tpbz)ReBr(CO)(μ_2-tpbz)Pt(pdt)], **18**. with partial atom labeling. All hydrogen atoms are omitted for clarity. The compound resides on an inversion center that is coincident with Re(1); consequently, the Br and CO ligands are disordered.

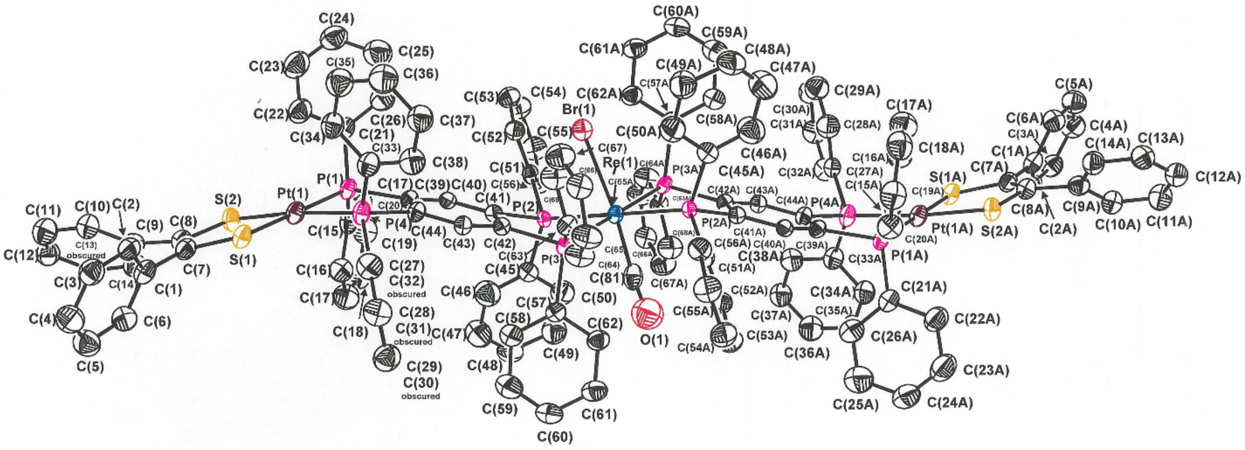

Figure S43. Thermal ellipsoid plot (35% level) of [(pdt)Pt(μ_2-tpbz)ReBr(CO)(μ_2-tpbz)Pt(pdt)], **18**, with complete atom labeling. All hydrogen atoms are omitted for clarity. The compound resides on an inversion center that is coincident with Re(1); consequently, the Br and CO ligands are disordered. The phenyl groups attached to C(8) and P(4) are disordered between two orientations. For clarity, only one positional variant is shown.

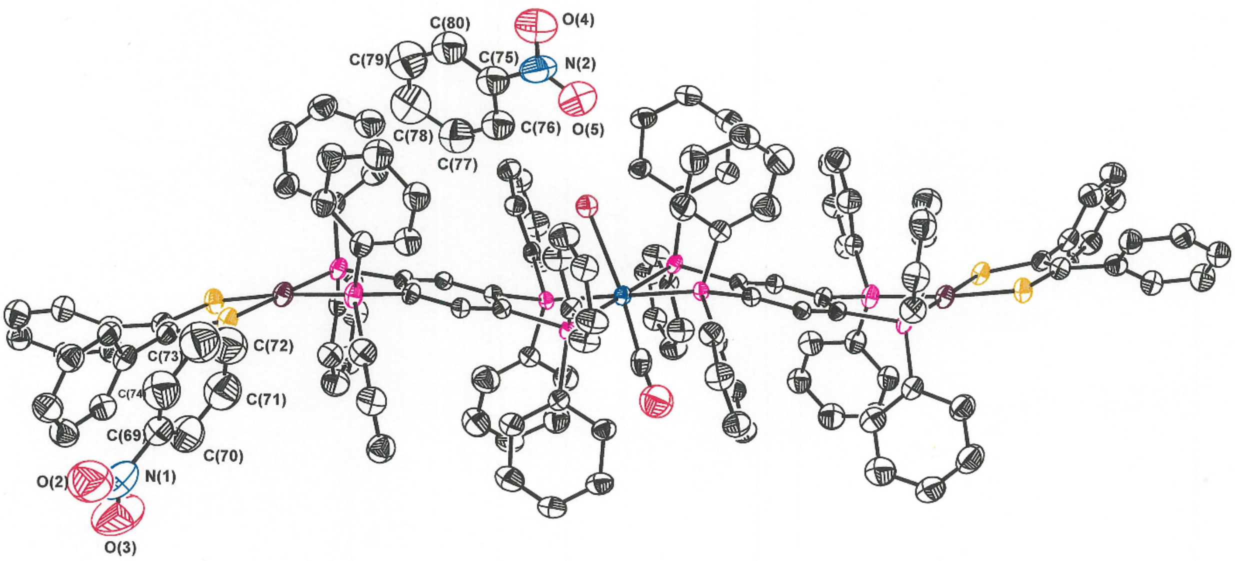

Figure S44. Atom labeling for interstitial solvent in [(pdt)Pt(μ_2-tpbz)ReBr(CO)(μ_2-tpbz)Pt(pdt)]·4($C_6H_5NO_2$). All hydrogen atoms are omitted for clarity. The thermal ellipsoids plot is presented at the 35% level.

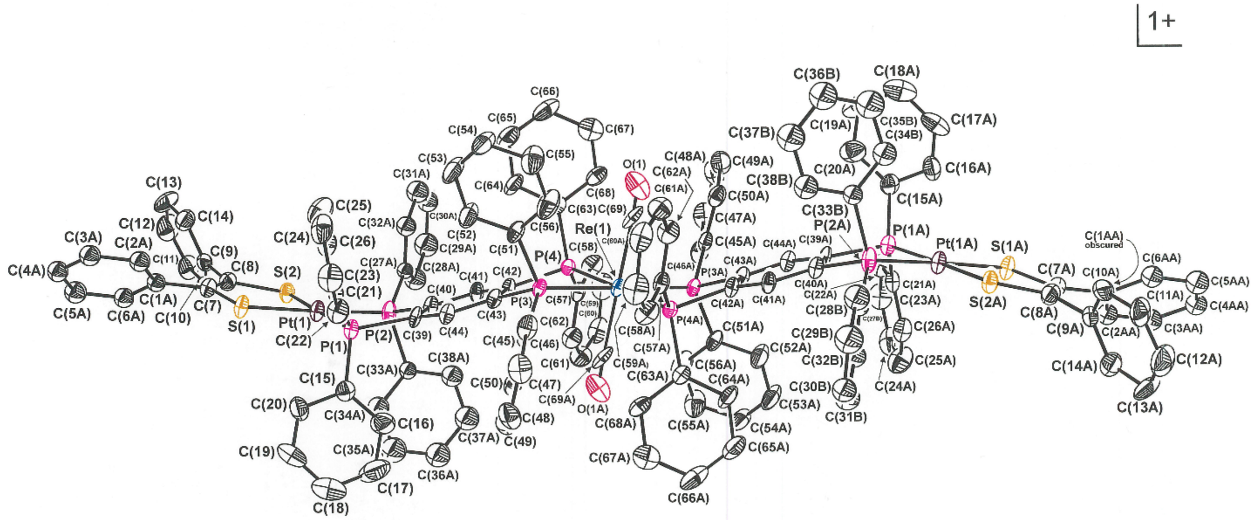

Figure S45. Thermal ellipsoid plot at the 30% level with complete atom labeling for [(pdt)Pt(μ_2-tpbz)Re(CO)$_2$(μ_2-tpbz)Pt(pdt)]$^{1+}$, [**19**]$^{1+}$, All hydrogen atoms are omitted for clarity. The phenyl rings defined by C(1A)-C(6A), C(27A)-C(32A), and C(33A)-C(38A) were each disordered over two positions, only one of which is shown. The cation crystallizes on an inversion center that is coincident with the position of Re(1).

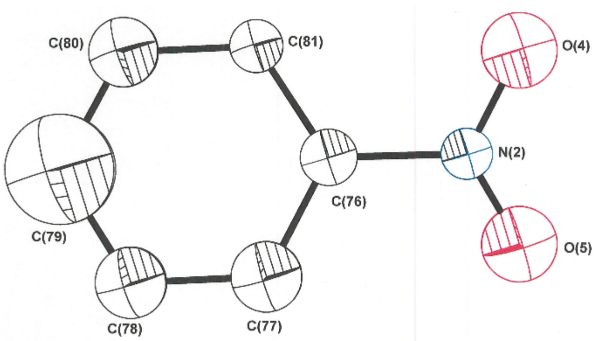

Figure S46. Atom labeling for interstitial solvent in [(pdt)Pt(μ_2-tpbz)Re(CO)$_2$(μ_2-tpbz)Pt(pdt)][Br]·3($PhNO_2$)·^{t}BuOMe. The thermal ellipsoid plot is presented at the 30% level. All hydrogen atoms are omitted for clarity. This $PhNO_2$ molecule is disordered across an inversion center; consequently, the positions shown are at 50% site occupancy.

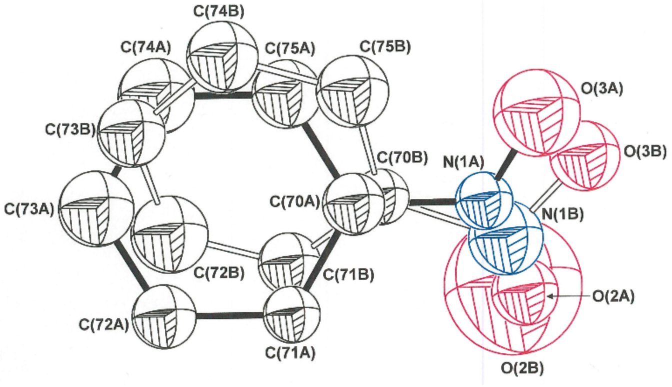

Figure S47. Atom labeling for interstitial solvent in [(pdt)Pt(μ_2-tpbz)Re(CO)$_2$(μ_2-tpbz)Pt(pdt)][Br]·3($PhNO_2$)·^{t}BuOMe. The thermal ellipsoid plot is presented at the 30% level. All hydrogen atoms are omitted for clarity. This $PhNO_2$ molecule is disordered over two positions and consequently is refined isotropically with appropriate distance restraints.

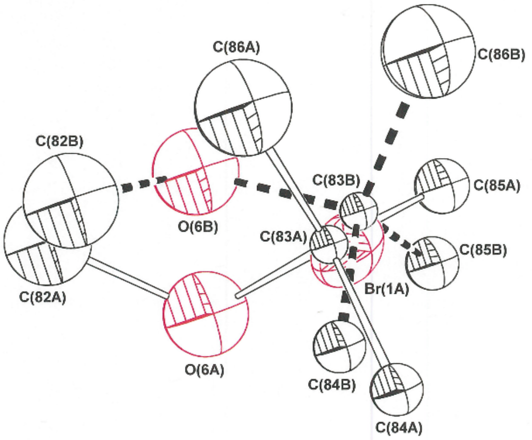

Figure S48. Atom labeling for interstitial ^{t}BuOMe in [(pdt)Pt(μ_2-tpbz)Re(CO)$_2$(μ_2-tpbz)Pt(pdt)][Br]·3($PhNO_2$)·^{t}BuOMe. The thermal ellipsoid plot is presented at the 30% level. All hydrogen atoms are omitted for clarity. The Br^{1-} anion and the ^{t}BuOMe molecule are disordered across an inversion center. The two positions shown for the ^{t}BuOMe molecule collectively amount to half a molecule such that the inversion center accounts for the other half. The interatomic distances are heavily restrained, and refinement is isotropic, except for the Br^{1-} anion.

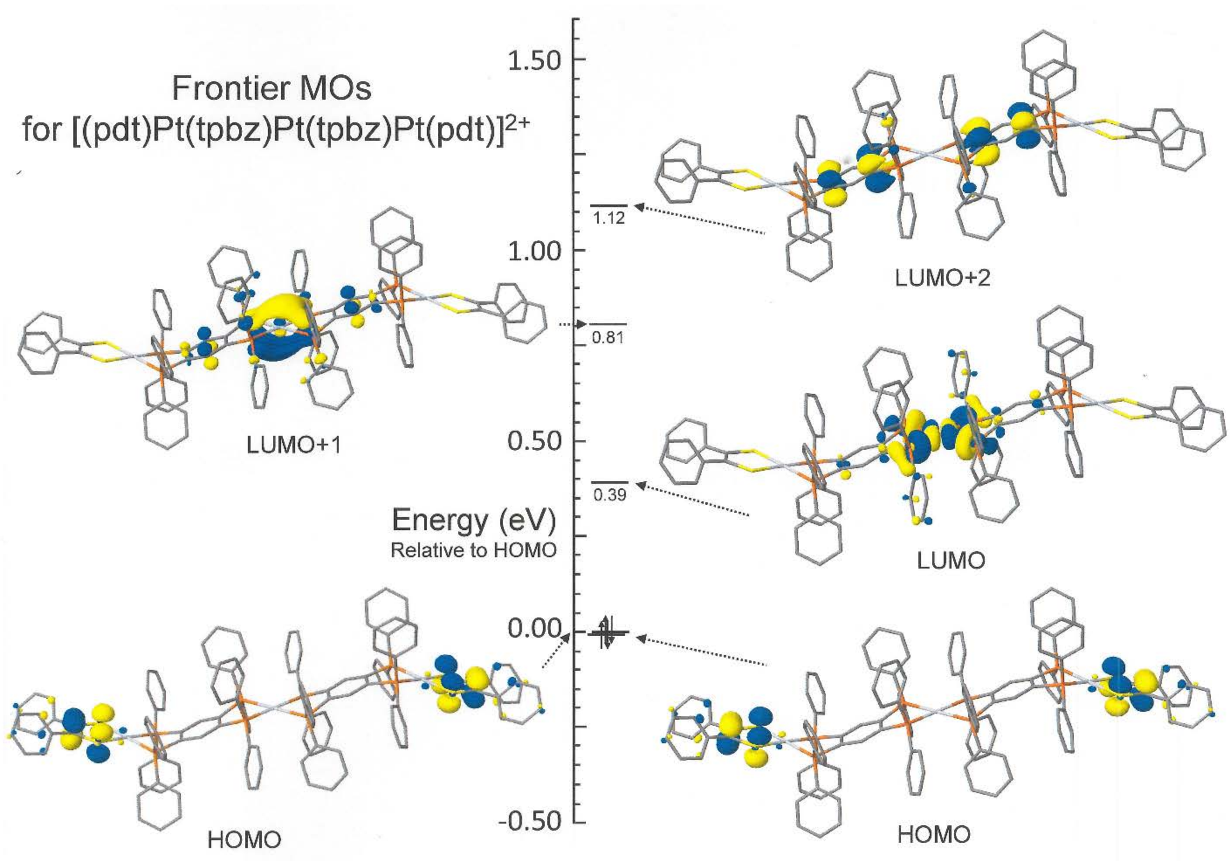

Figure S49. Frontier MOs for $[(Ph_2C_2S_2)Pt(tpbz)Pt(tpbz)Pt(S_2C_2Ph_2)]^{2+}$, as assessed from geometry optimization using Gaussian. Orbital images are presented at the 0.03 contour level.

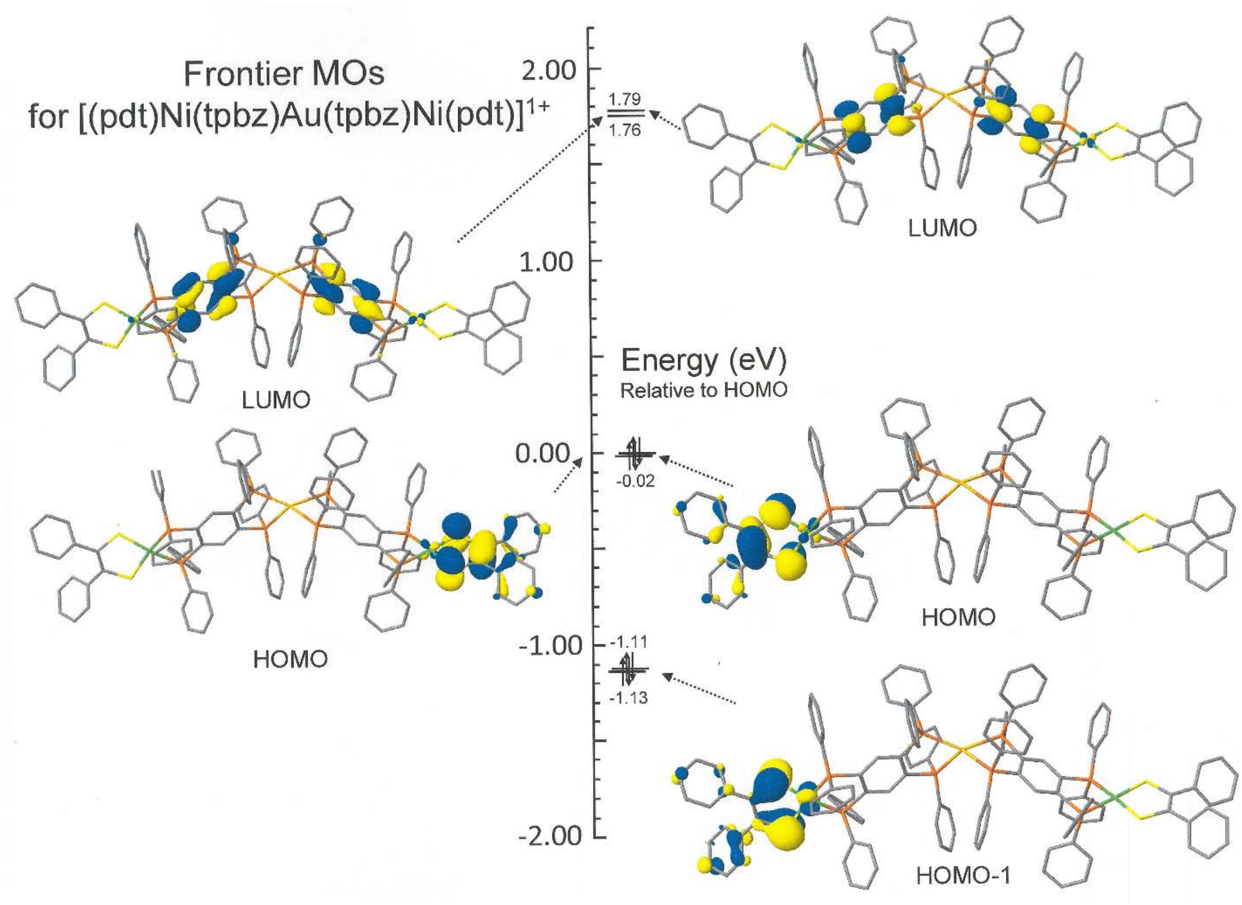

Figure S50. Frontier MOs for $[(Ph_2C_2S_2)Ni(tpbz)Au(tpbz)Ni(S_2C_2Ph_2)]^{2+}$, as assessed from geometry optimization using Gaussian. Orbital images are presented at the 0.03 contour level.

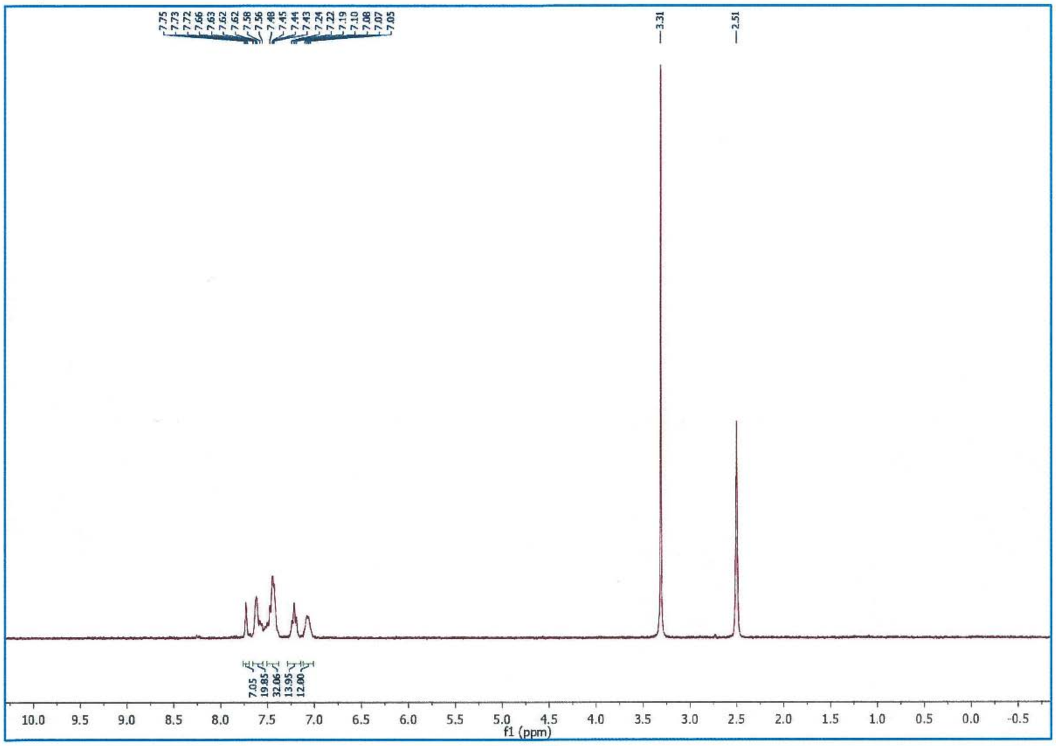

Figure S51. ^{1}H NMR spectrum (DMSO-d_6) of $[((NC)_2C_2S_2)Ni(tpbz)Pt(tpbz)Ni(S_2C_2(CN)_2)][CF_3SO_3]_2$, [**1**]$[CF_3SO_3]_2$.

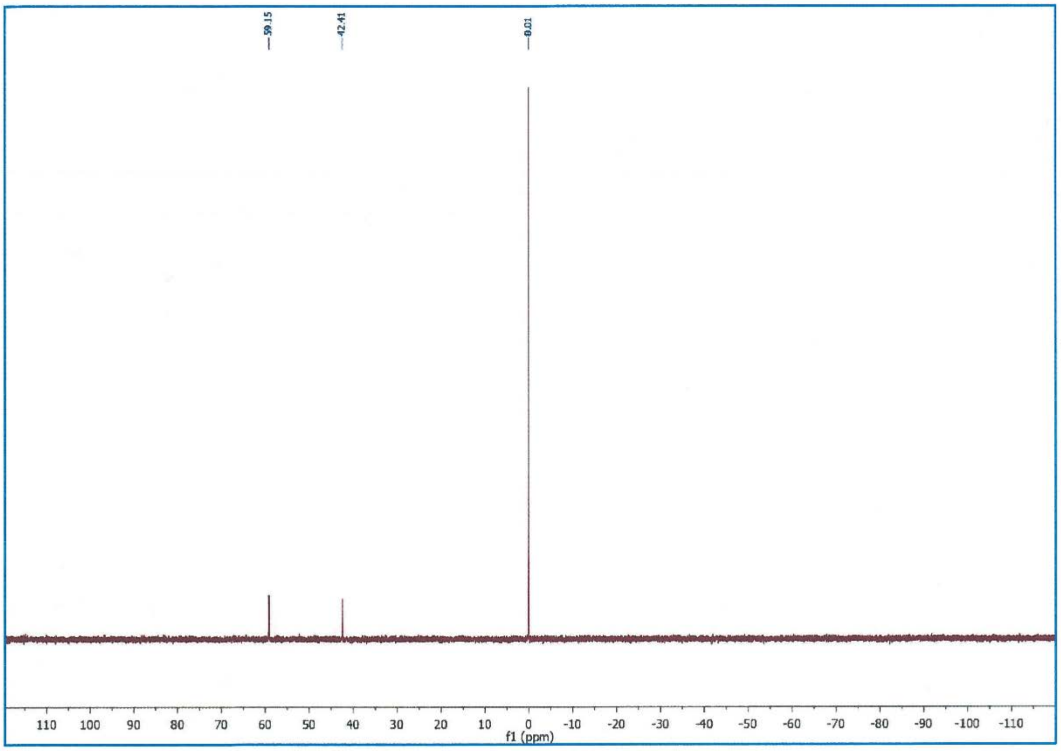

Figure S52. ^{31}P-{^{1}H} NMR spectrum (DMSO-d_6) of $[((NC)_2C_2S_2)Ni(tpbz)Pt(tpbz)Ni(S_2C_2(CN)_2)][CF_3SO_3]_2$, [**1**]$[CF_3SO_3]_2$.

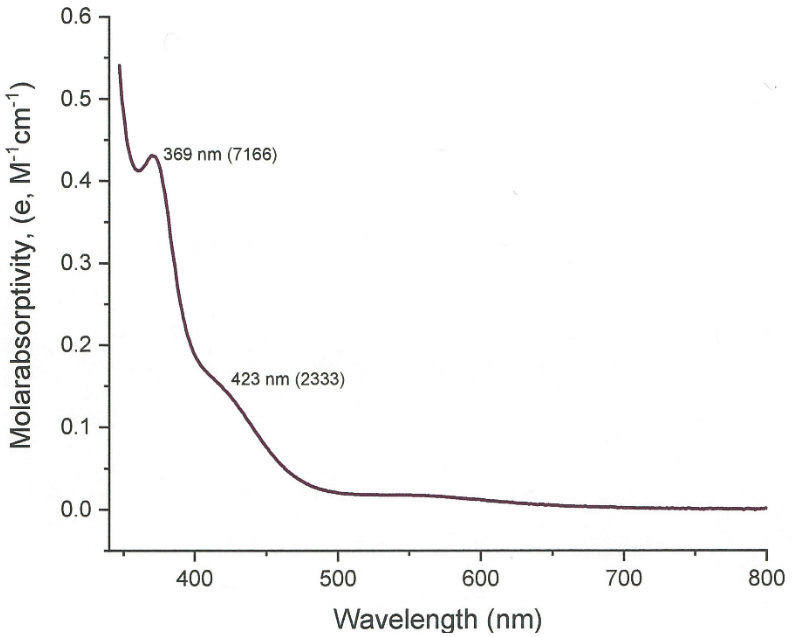

Figure S53. UV-vis spectrum of $[((NC)_2C_2S_2)Ni(tpbz)Pt(tpbz)Ni(S_2C_2(CN)_2)][CF_3SO_3]_2$, [**1**]$[CF_3SO_3]_2$, in *N*,*N*-dimethylformamide.

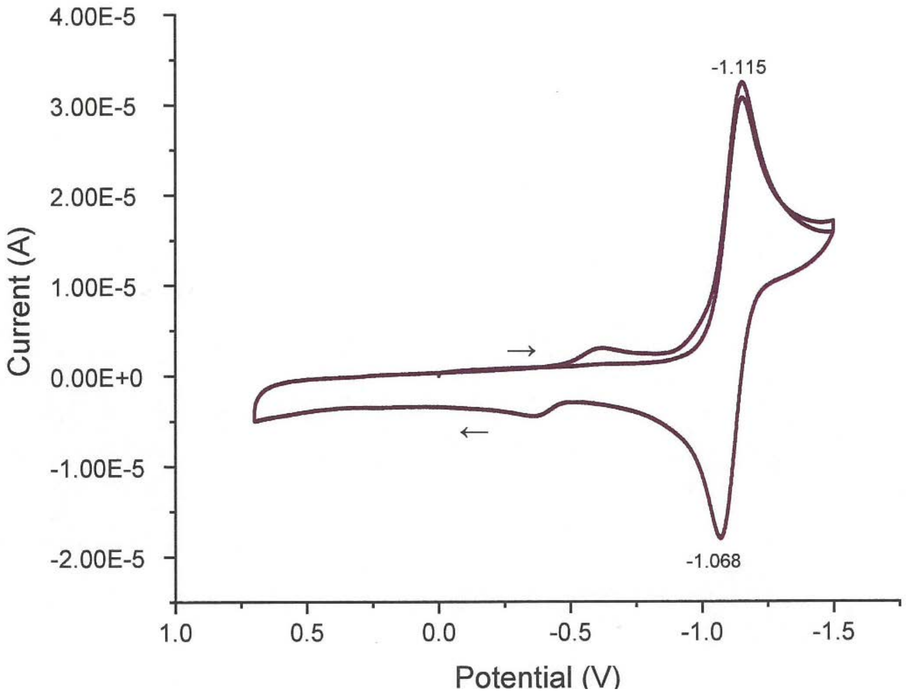

Figure S54. Cyclic voltammogram of $[((NC)_2C_2S_2)Ni(tpbz)Pt(tpbz)Ni(S_2C_2(CN)_2)][CF_3SO_3]_2$, [**1**]$[CF_3SO_3]_2$, in *N*,*N*-dimethylformamide at 25 °C using $[^nBu_4N][PF_6]$ as supporting electrolyte. The reference electrode is Ag/AgCl.

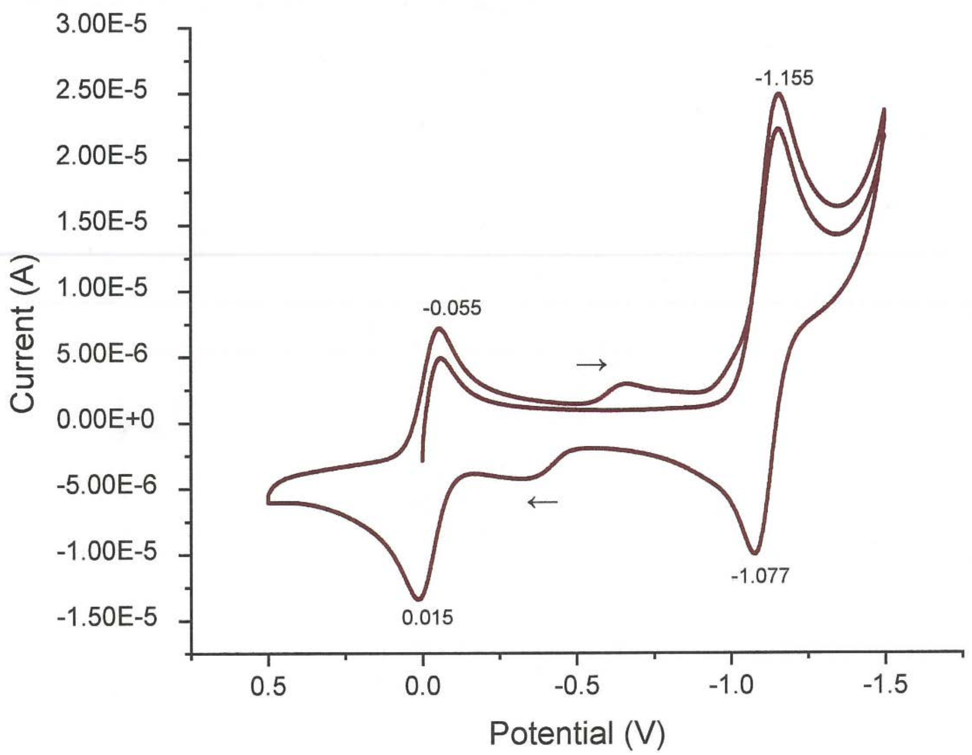

Figure S55. Cyclic voltammogram of $[((NC)_2C_2S_2)Ni(tpbz)Pt(tpbz)Ni(S_2C_2(CN)_2)][CF_3SO_3]_2$, [**1**]$[CF_3SO_3]_2$, in *N*,*N*-dimethylformamide at 25 °C using $[^nBu_4N][PF_6]$ as supporting electrolyte. The reference electrode is Ag/AgCl, and $[(Me_5C_5)_2Fe]$ has been added (1 eq.) as an internal standard.

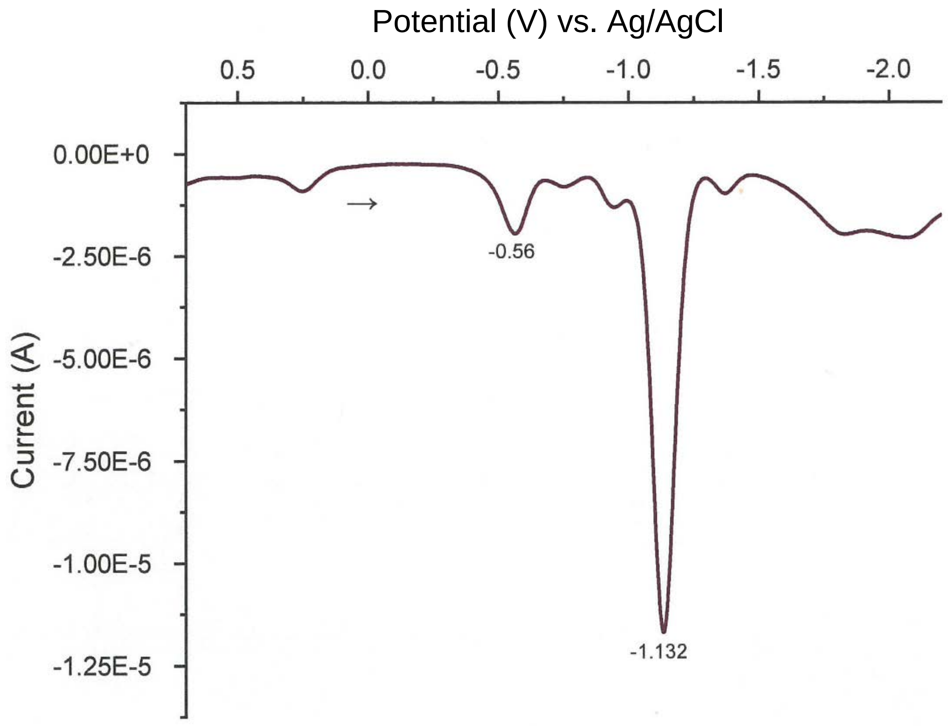

Figure S56. Differential pulse voltammogram of $[((NC)_2C_2S_2)Ni(tpbz)Pt(tpbz)Ni(S_2C_2(CN)_2)][CF_3SO_3]_2$, [**1**]$[CF_3SO_3]_2$, in *N*,*N*-dimethylformamide at 25 °C using $[^nBu_4N][PF_6]$ as supporting electrolyte. The reference electrode is Ag/AgCl, and the pulse amplitude is 50 mV.

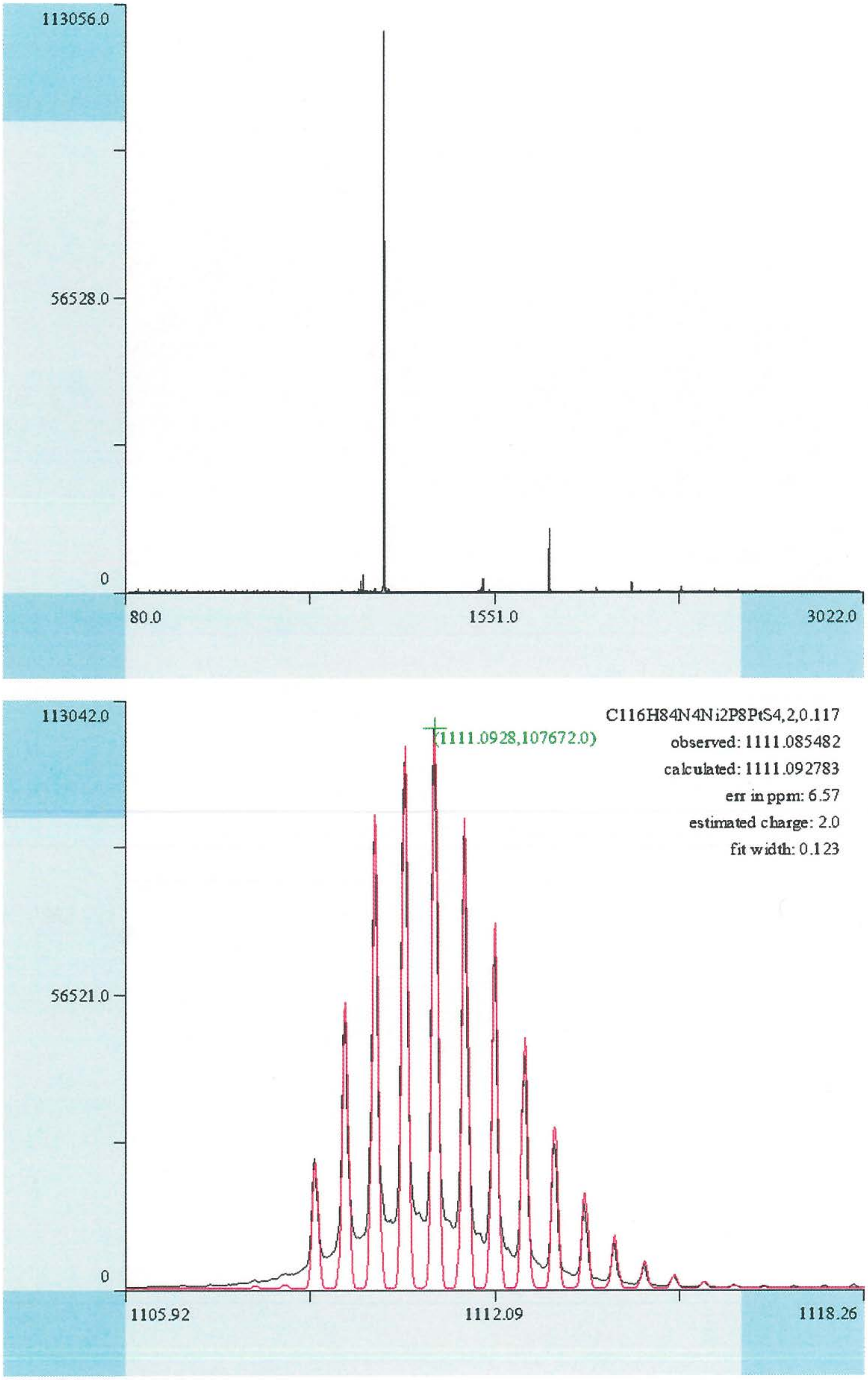

Figure S57. Mass spectrum (ESI+) of $[((NC)_2C_2S_2)Ni(tpbz)Pt(tpbz)Ni(S_2C_2(CN)_2)][CF_3SO_3]_2$, [**1**]$[CF_3SO_3]_2$.

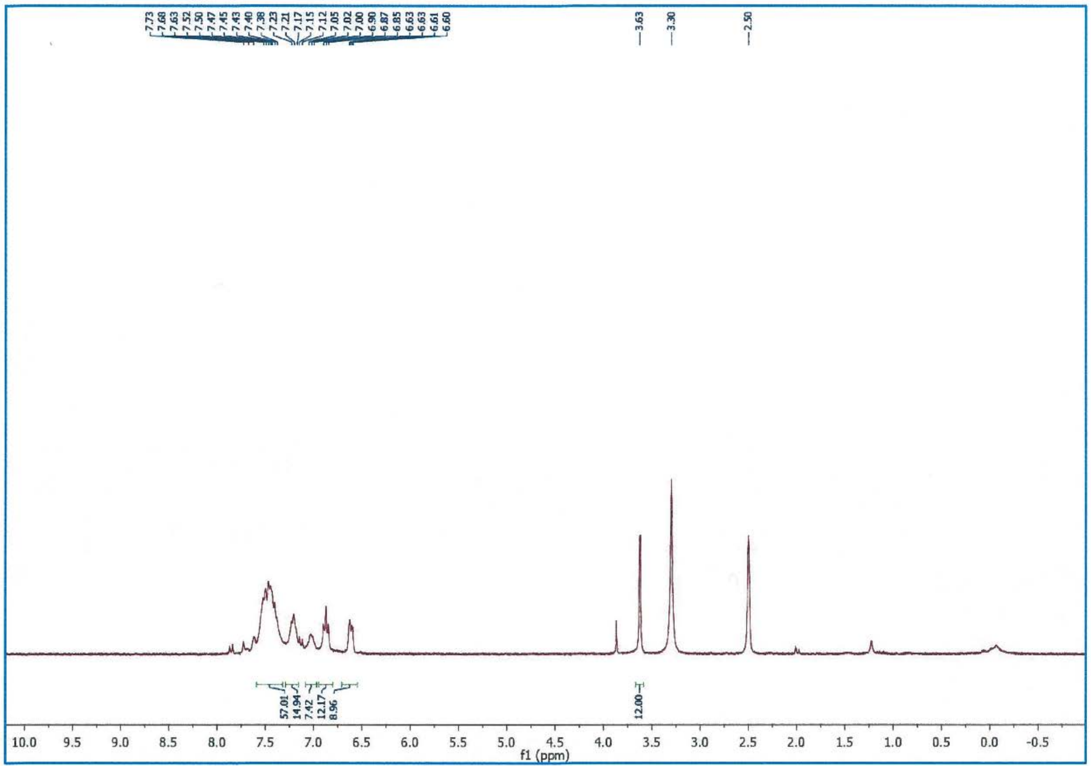

Figure S58. ^{1}H NMR spectrum (DMSO-d_6) of [(adt)Ni(tpbz)Pt(tpbz)Ni(adt)][CF_3SO_3]$_2$, [**2**][CF_3SO_3]$_2$.

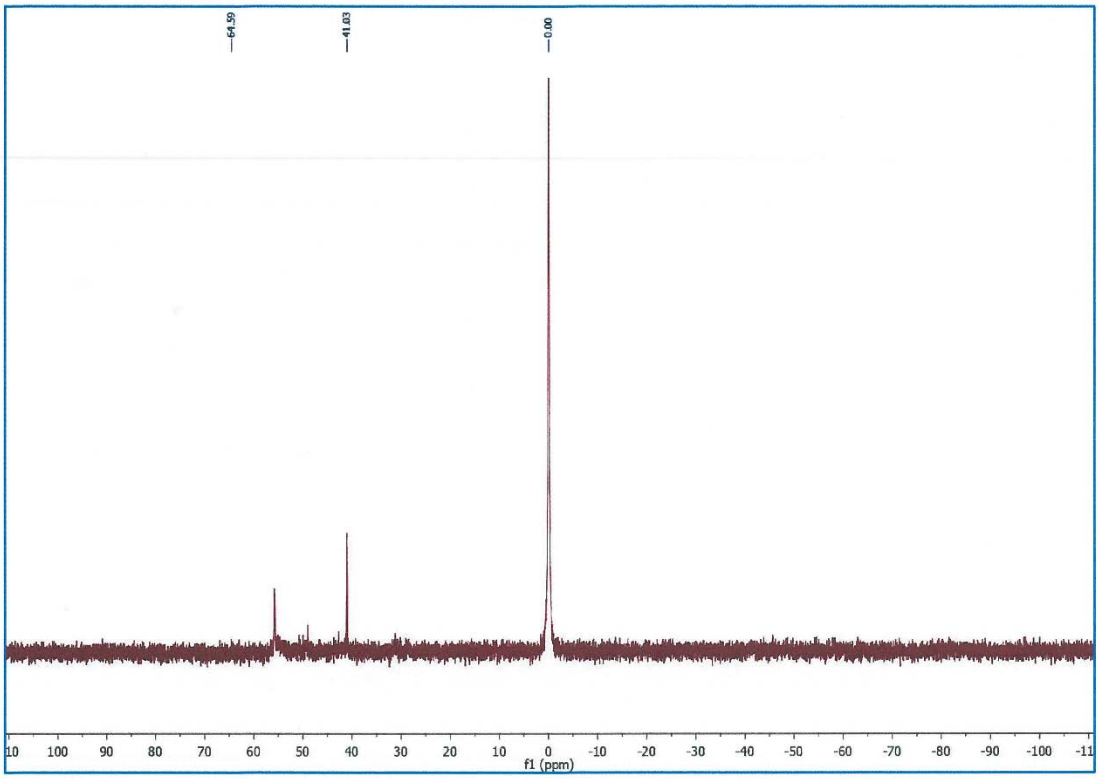

Figure S59. ^{31}P-{^{1}H} NMR spectrum (DMSO-d_6) of [(adt)Ni(tpbz)Pt(tpbz)Ni(adt)][CF_3SO_3]$_2$, [**2**][CF_3SO_3]$_2$.

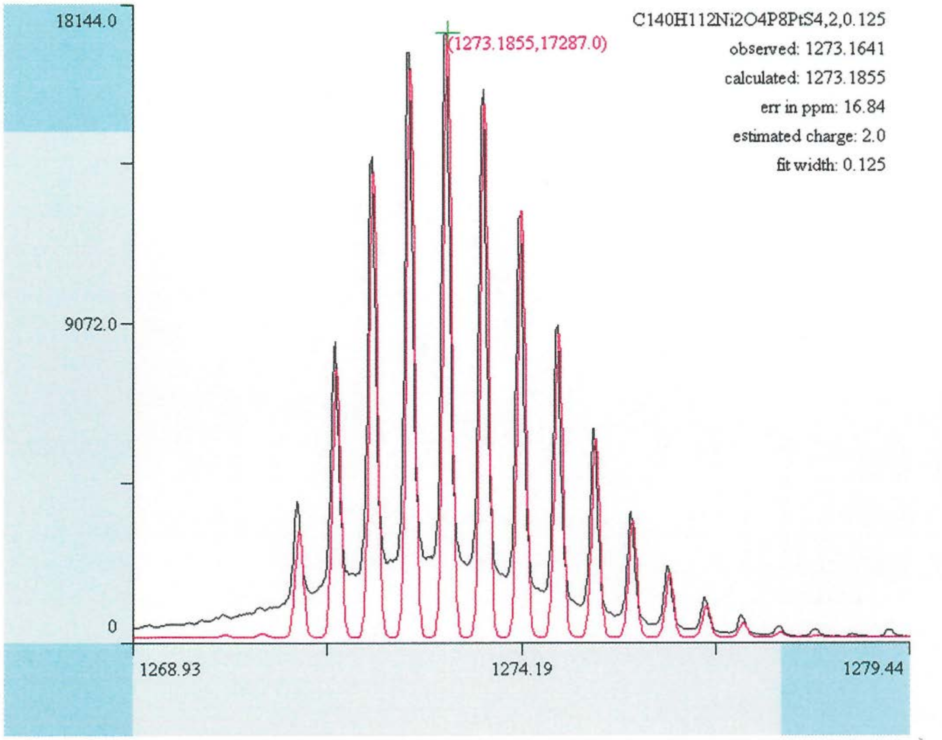

Figure S60. Mass spectrum (ESI+) of [(adt)Ni(tpbz)Pt(tpbz)Ni(adt)][CF_3SO_3]$_2$, [**2**][CF_3SO_3]$_2$.

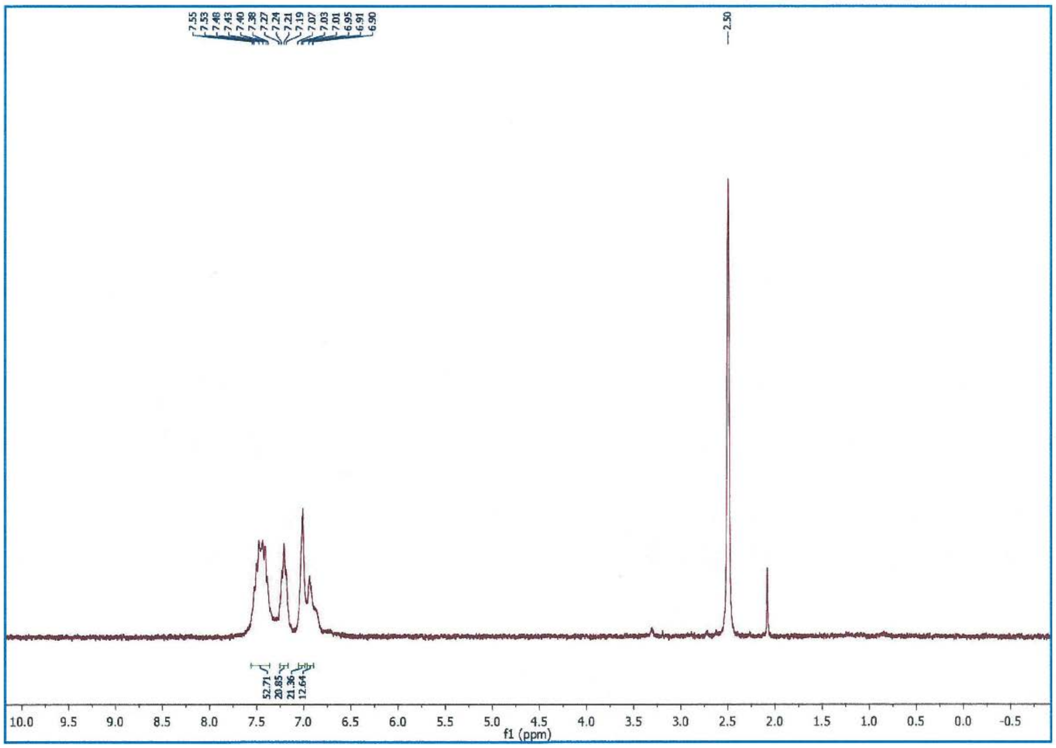

Figure S61. ^{1}H NMR spectrum (DMSO-d_6) of [(pdt)Ni(tpbz)Pt(tpbz)Ni(pdt)][CF_3SO_3]$_2$, [**3**][CF_3SO_3]$_2$.

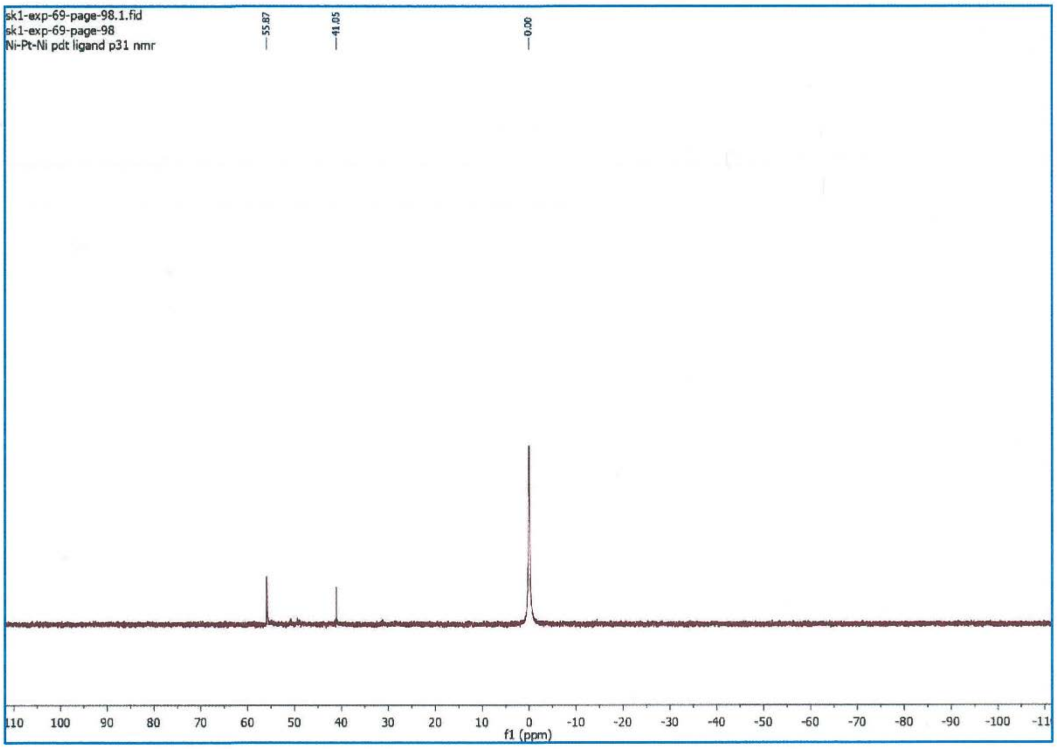

Figure S62. ^{31}P-{^{1}H} NMR spectrum (DMSO-d_6) of [(pdt)Ni(tpbz)Pt(tpbz)Ni(pdt)][CF_3SO_3]$_2$, [**3**][CF_3SO_3]$_2$.

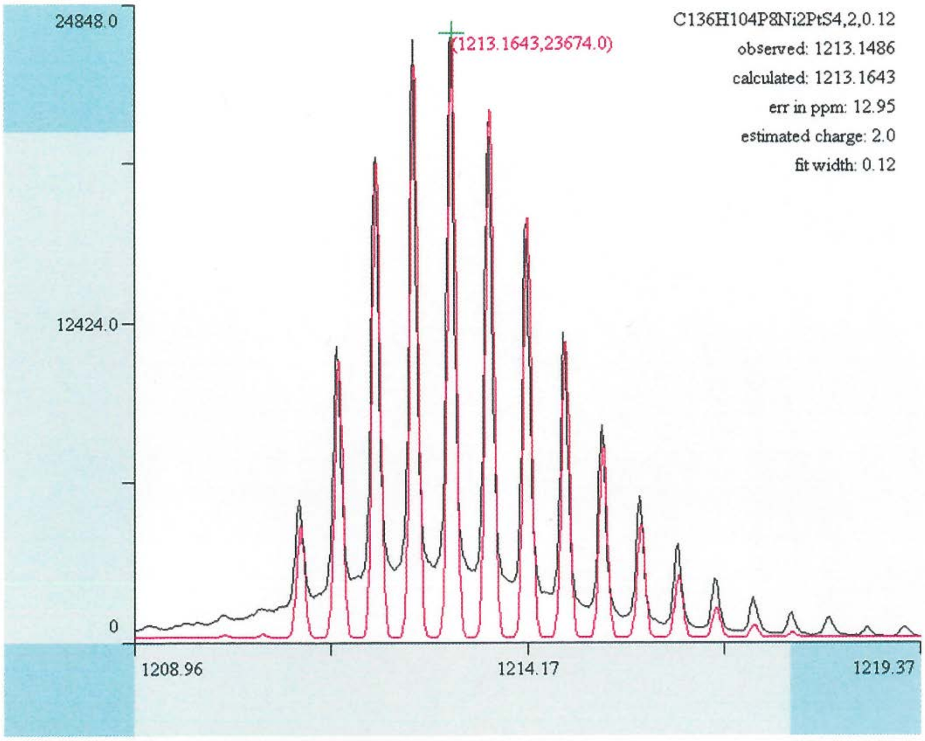

Figure S63. Mass spectrum (ESI+) of [(pdt)Ni(tpbz)Pt(tpbz)Ni(pdt)][CF_3SO_3]$_2$, [**3**][CF_3SO_3]$_2$.

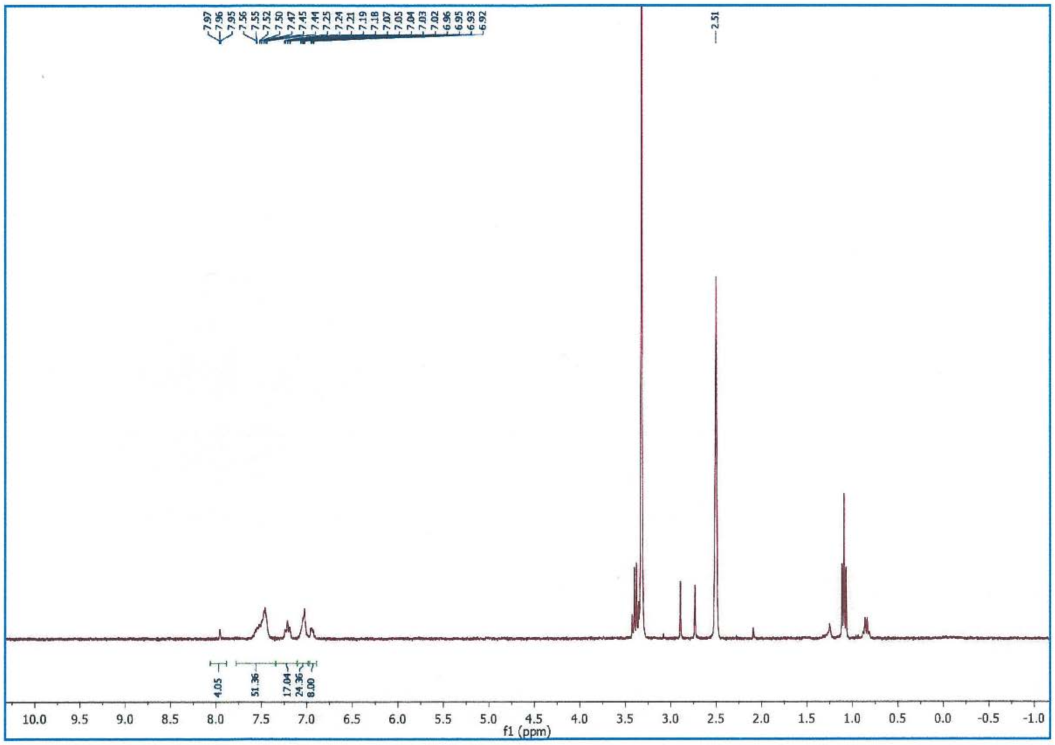

Figure S64. ^{1}H NMR spectrum (DMSO-d_6) of [(pdt)Pd(tpbz)Pt(tpbz)Pd(pdt)][CF_3SO_3]$_2$, [**4**][CF_3SO_3]$_2$.

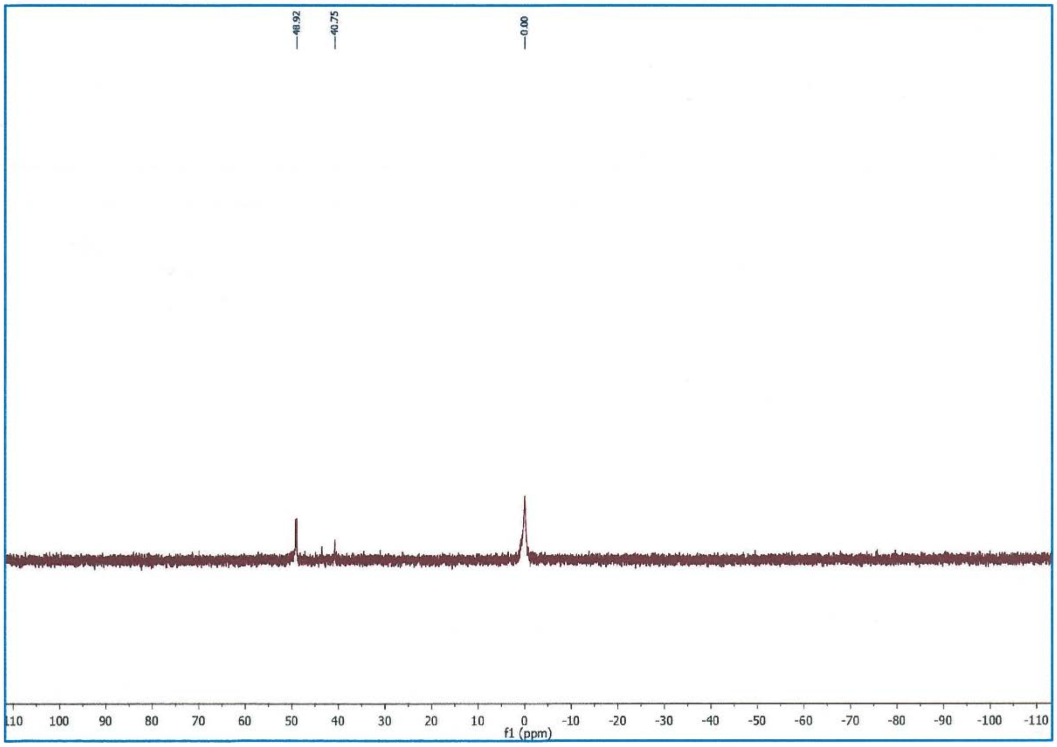

Figure S65. ^{31}P-{^{1}H} NMR spectrum (DMSO-d_6) of [(pdt)Pd(tpbz)Pt(tpbz)Pd(pdt)][CF_3SO_3]$_2$, [**4**][CF_3SO_3]$_2$.

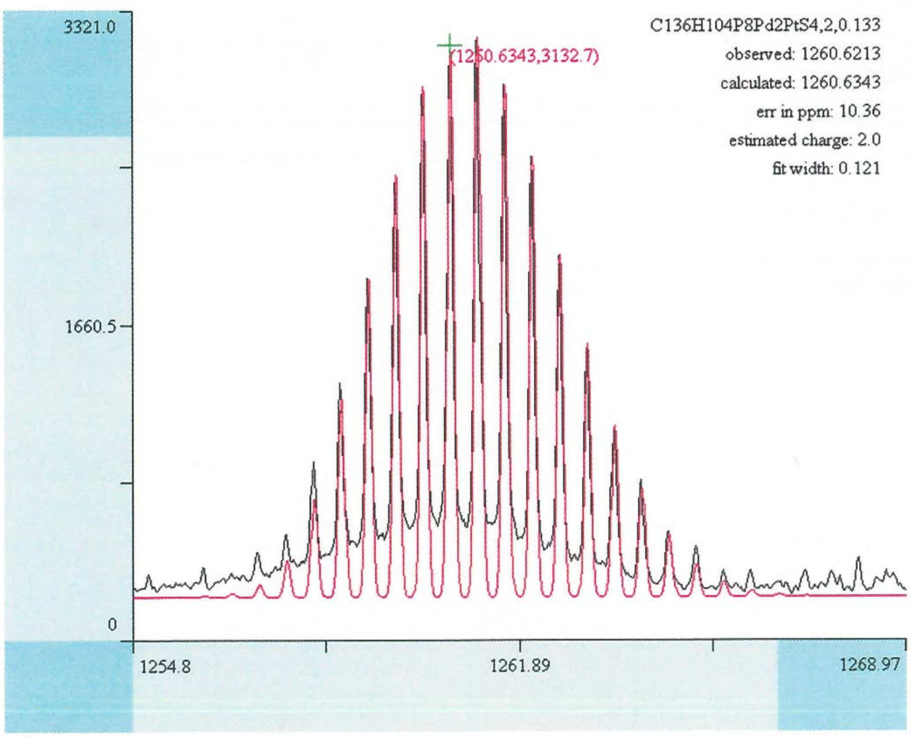

Figure S66. Mass spectrum (ESI+) of [(pdt)Pd(tpbz)Pt(tpbz)Pd(pdt)][CF_3SO_3]$_2$, [**4**][CF_3SO_3]$_2$.

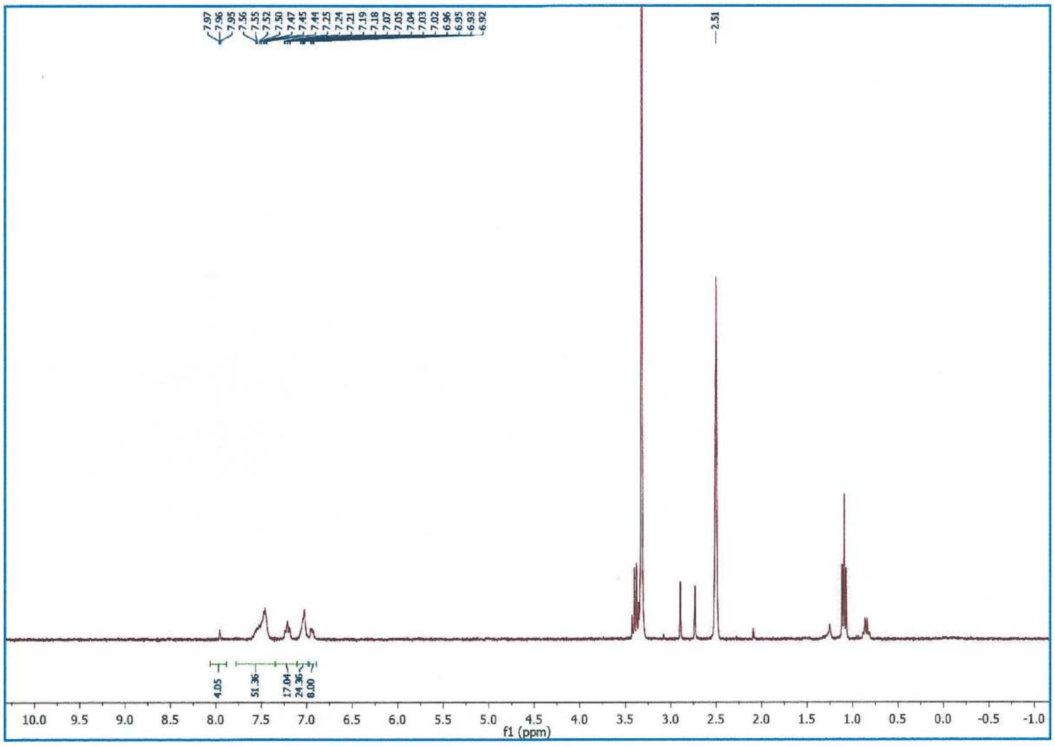

Figure S67. ^{1}H NMR spectrum (DMSO-d_6) of [(pdt)Pt(tpbz)Pt(tpbz)Pt(pdt)][CF_3SO_3]$_2$, [**5**][CF_3SO_3]$_2$.

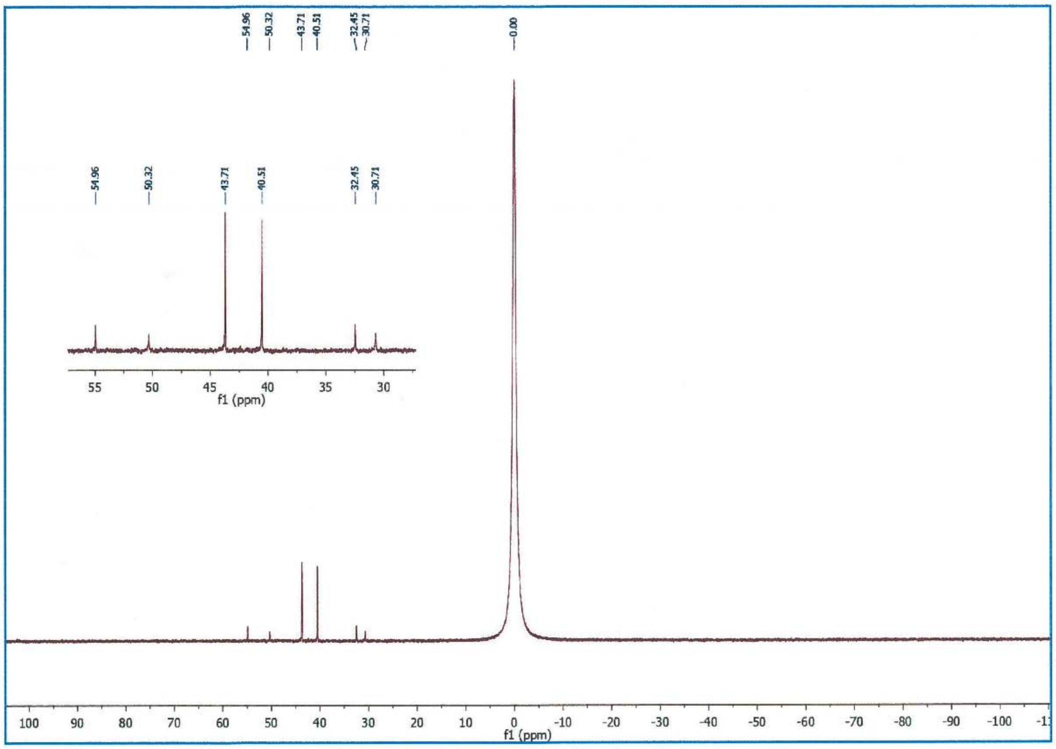

Figure S68. ^{31}P-{^{1}H} NMR spectrum (DMSO-d_6) of [(pdt)Pt(tpbz)Pt(tpbz)Pt(pdt)][CF_3SO_3]$_2$, [**5**][CF_3SO_3]$_2$.

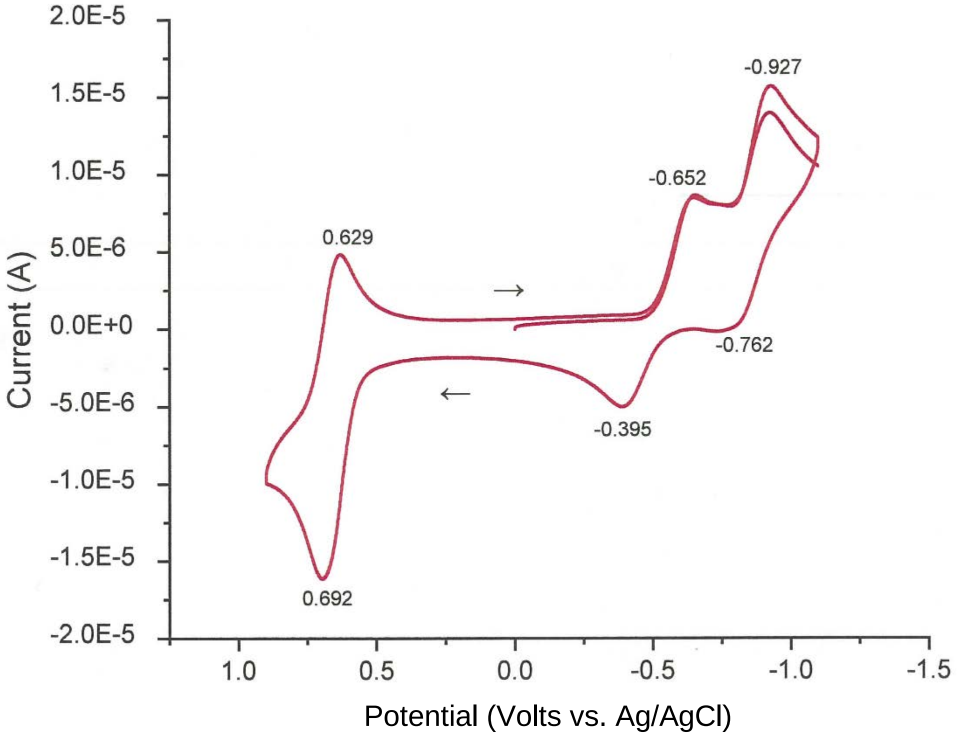

Figure S69. Cyclic voltammogram of [($Ph_2C_2S_2$)Pt(tpbz)Pt(tpbz)Pt($S_2C_2Ph_2$)][CF_3SO_3]$_2$, [**5**][CF_3SO_3]$_2$, in *N*,*N*-dimethylformamide at 25 °C using [^{n}Bu$_4$N][PF_6] as supporting electrolyte. The reference electrode is Ag/AgCl, and the scan rate is 100 mV/sec.

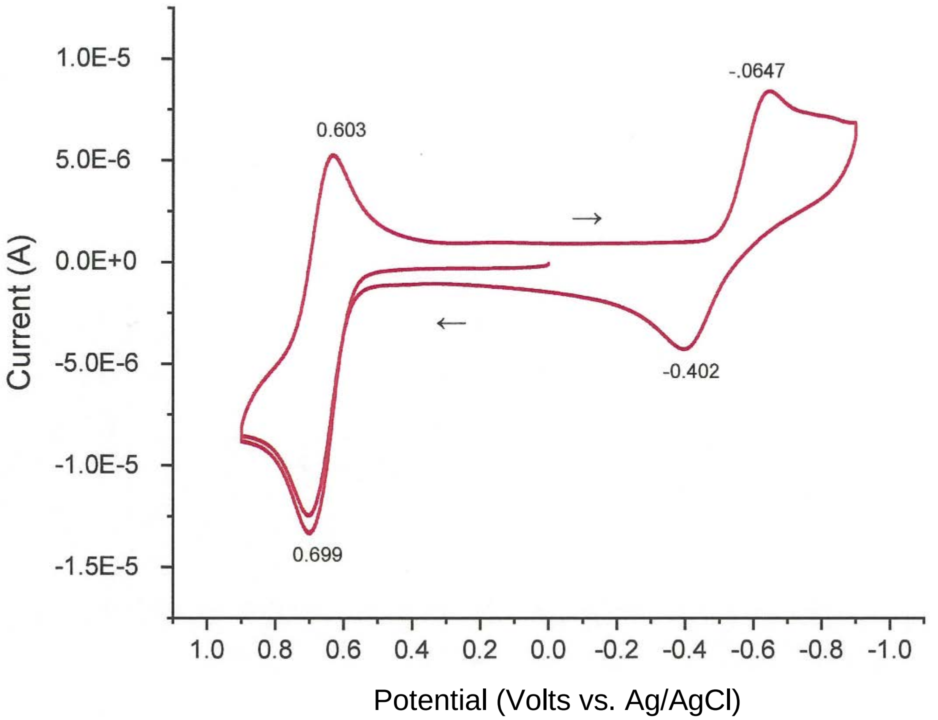

Figure S70. Cyclic voltammogram of [($Ph_2C_2S_2$)Pt(tpbz)Pt(tpbz)Pt($S_2C_2Ph_2$)][CF_3SO_3]$_2$, [**5**][CF_3SO_3]$_2$, in *N*,*N*-dimethylformamide at 25 °C using [^{n}Bu$_4$N][PF_6] as supporting electrolyte. The reference electrode is Ag/AgCl, and the scan rate is 100 mV/sec. Cathodic scanning is stopped at –0.90 V before the onset of the second reduction process.

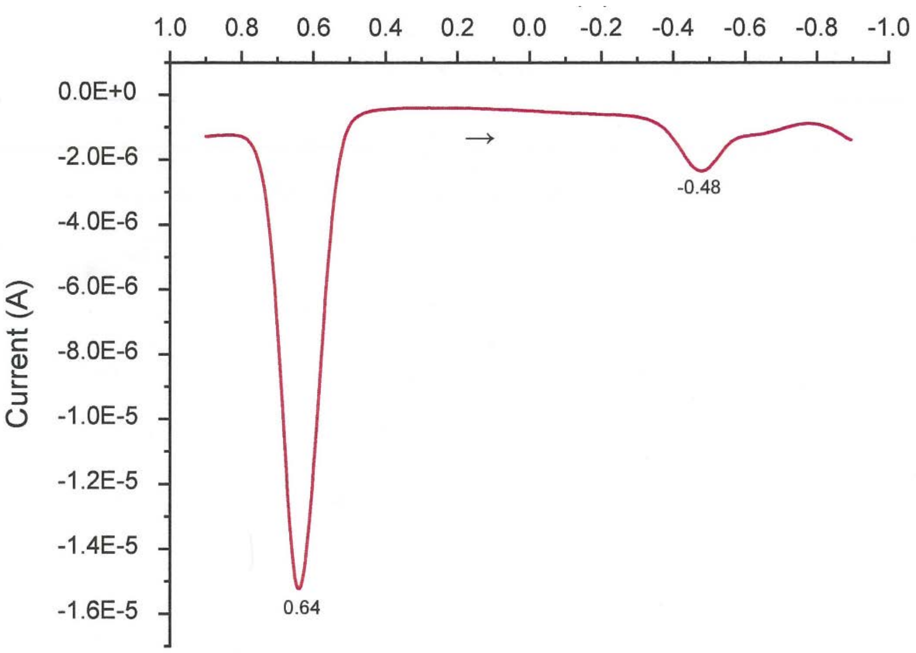

Figure S71. Differential pulse voltammogram of $[(Ph_2C_2S_2)Pt(tpbz)Pt(tpbz)Pt(S_2C_2Ph_2)][CF_3SO_3]_2$, [**5**]$[CF_3SO_3]_2$, in *N*,*N*-dimethylformamide at 25 °C using $[^nBu_4N][PF_6]$ as supporting electrolyte. The reference electrode is Ag/AgCl, and the pulse amplitude is 50 mV.

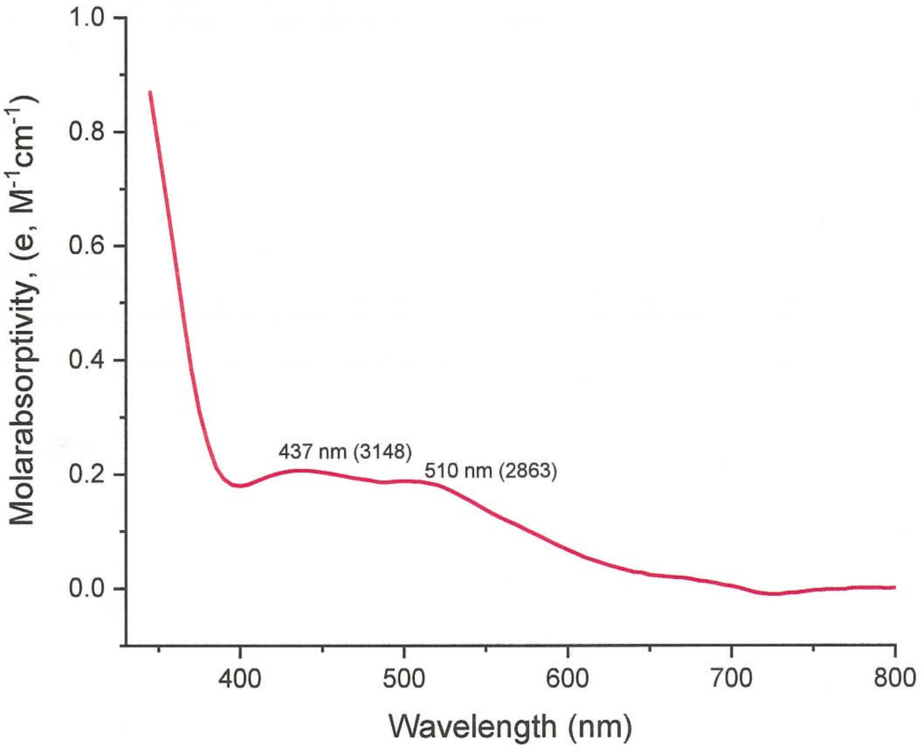

Figure S72. UV-vis spectrum of $[(Ph_2C_2S_2)Pt(tpbz)Pt(tpbz)Pt(S_2C_2Ph_2)][CF_3SO_3]_2$, [**5**]$[CF_3SO_3]_2$, in *N*,*N*-dimethylformamide.

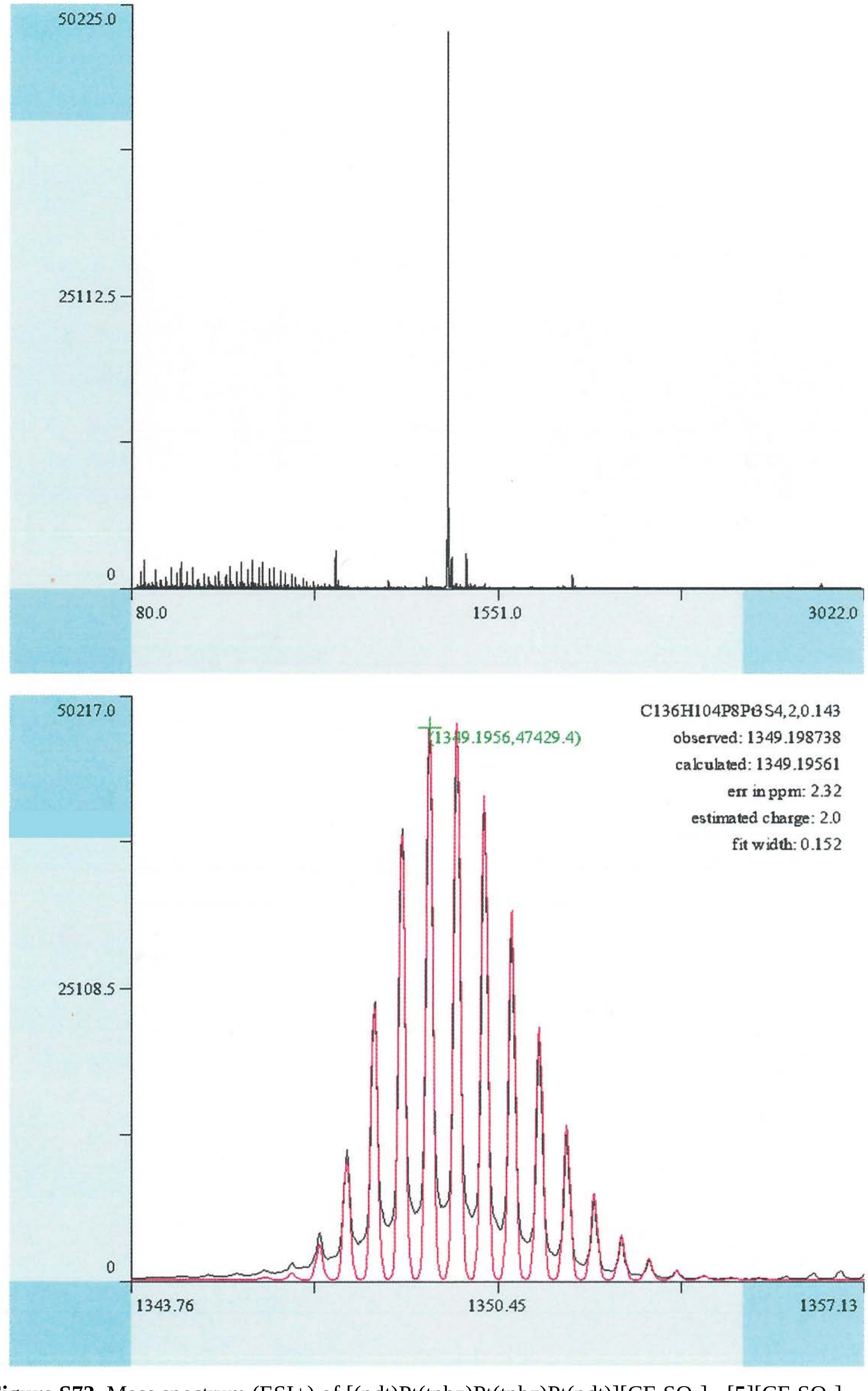

Figure S73. Mass spectrum (ESI+) of [(pdt)Pt(tpbz)Pt(tpbz)Pt(pdt)][CF_3SO_3]$_2$, [**5**][CF_3SO_3]$_2$.

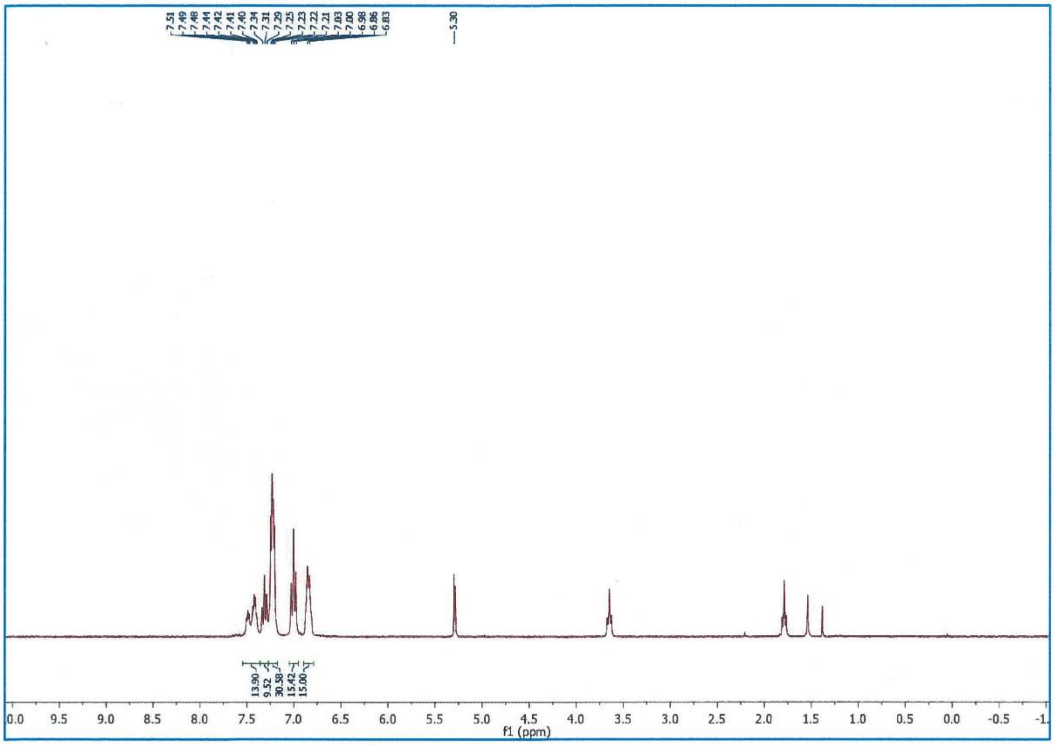

Figure S74. ^{1}H NMR spectrum (CD_2Cl_2) of [(mnt)Ni(tpbz)Au(tpbz)Ni(mnt)][CF_3SO_3], [**6**][CF_3SO_3].

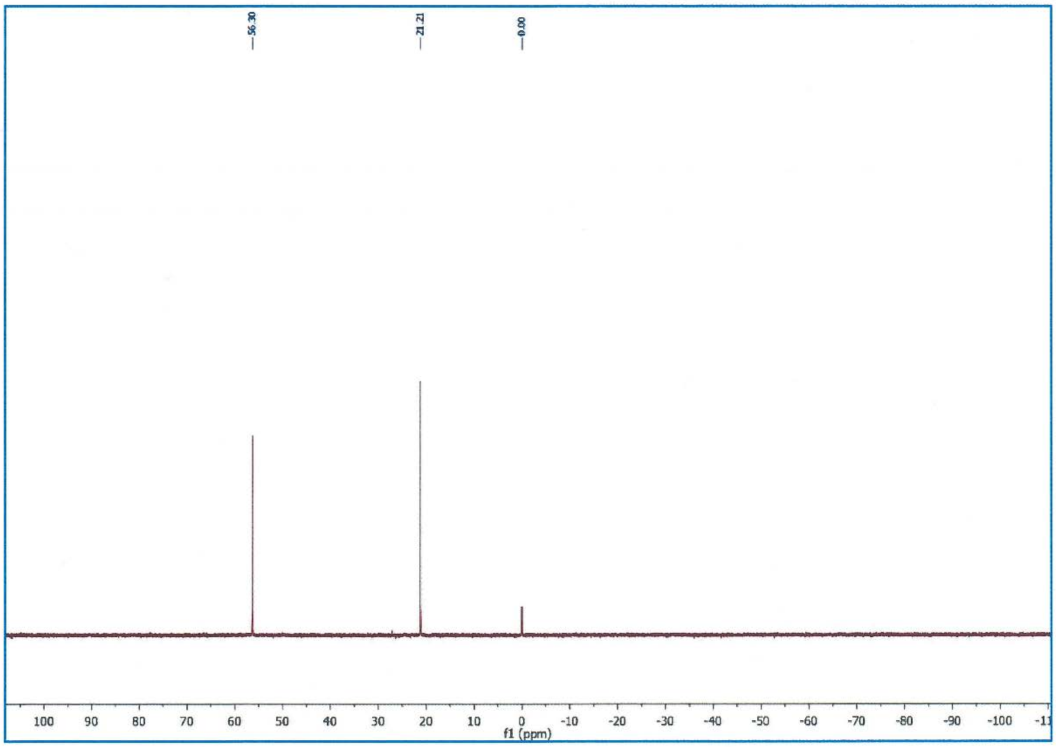

Figure S75. ^{31}P-{^{1}H} NMR spectrum (CD_2Cl_2) of [(mnt)Ni(tpbz)Au(tpbz)Ni(mnt)][CF_3SO_3], [**6**][CF_3SO_3].

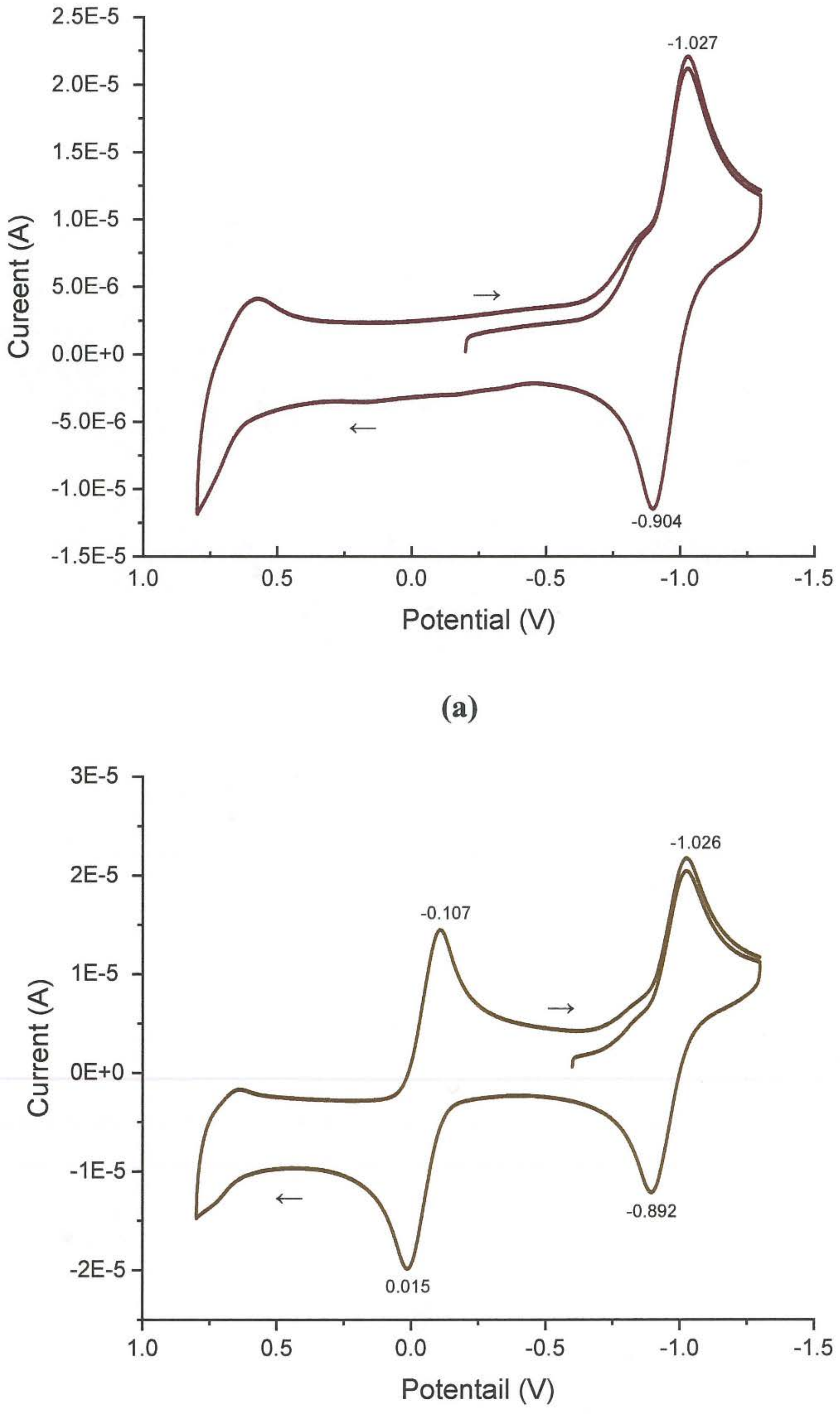

(a)

(b)

Figure S76. (**a**) Cyclic voltammogram of [(mnt)Ni(tpbz)Au(tpbz)Ni(mnt)][CF_3SO_3], [**6**][CF_3SO_3], in CH_2Cl_2 at 25 °C using [^{n}Bu_4N][PF_6] as supporting electrolyte. The reference electrode is Ag/AgCl, and the scan rate is 100 mV/sec. (**b**) Cyclic voltammogram of, [**6**][CF_3SO_3] under the same conditions as in (**a**) but with 1 eq of Cp^*_2Fe added as standard.

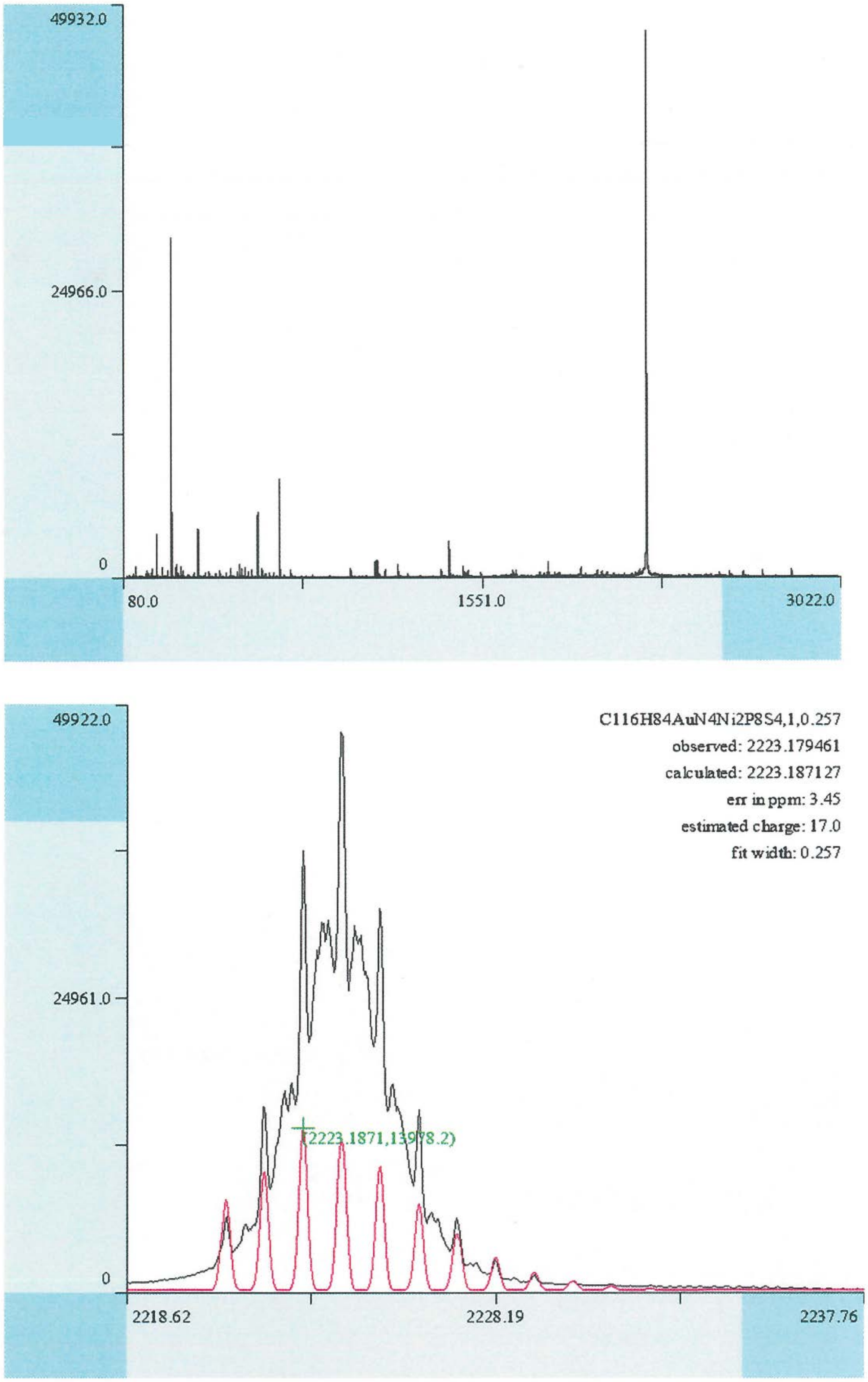

Figure S77. Mass spectrum (ESI+) of [(mnt)Ni(tpbz)Au(tpbz)Ni(mnt)][CF_3SO_3], [**6**][CF_3SO_3].

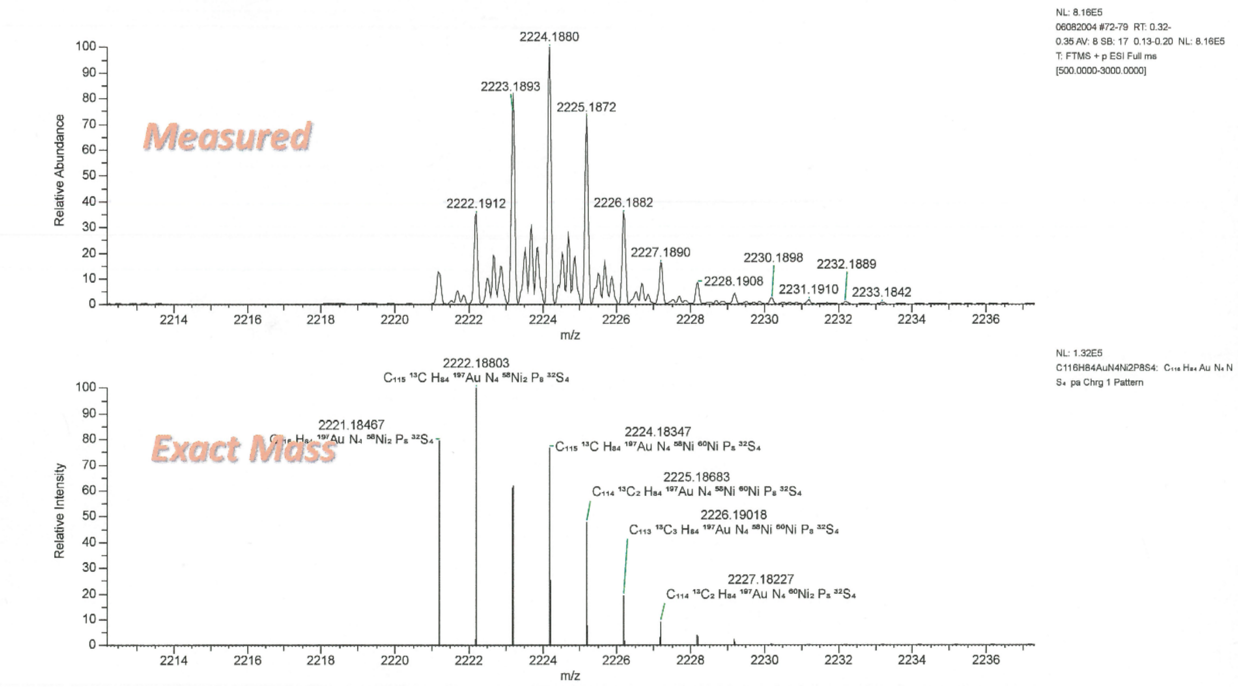

Figure S78. Mass spectrum (ESI+) of [(mnt)Ni(tpbz)Au(tpbz)Ni(mnt)][CF_3SO_3], [**6**][CF_3SO_3].

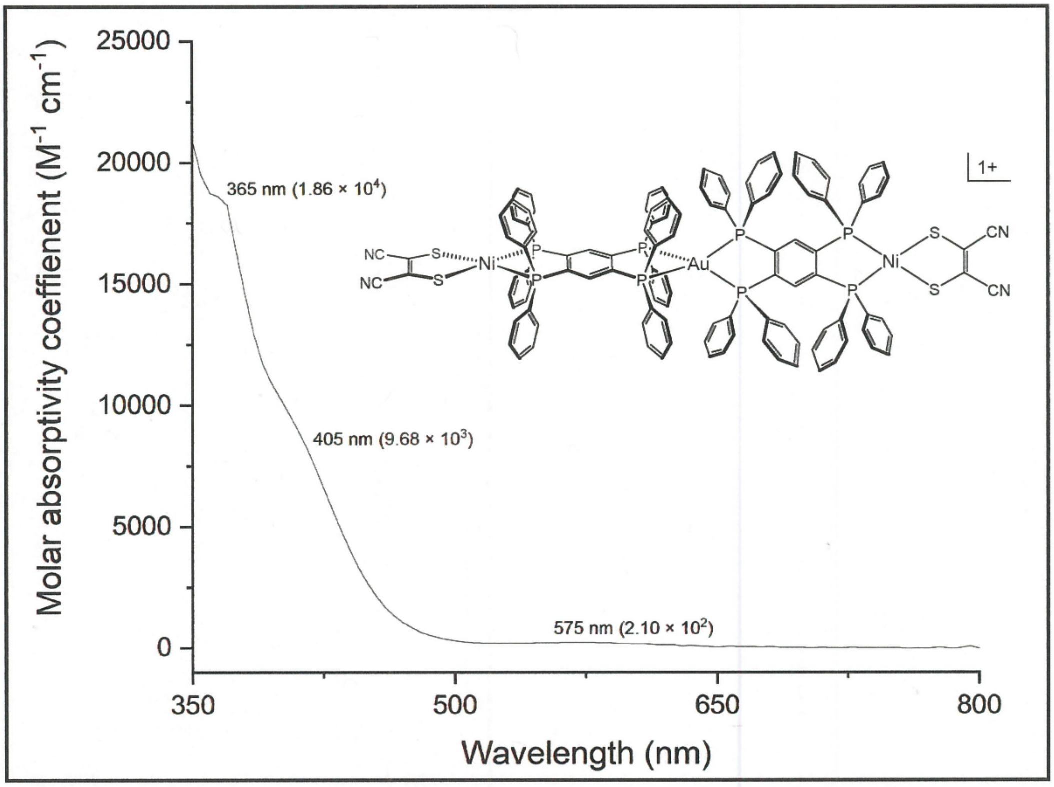

Figure S79. UV-vis spectrum of [(mnt)Ni(tpbz)Au(tpbz)Ni(mnt)][CF_3SO_3], [**6**][CF_3SO_3] in CH_2Cl_2.

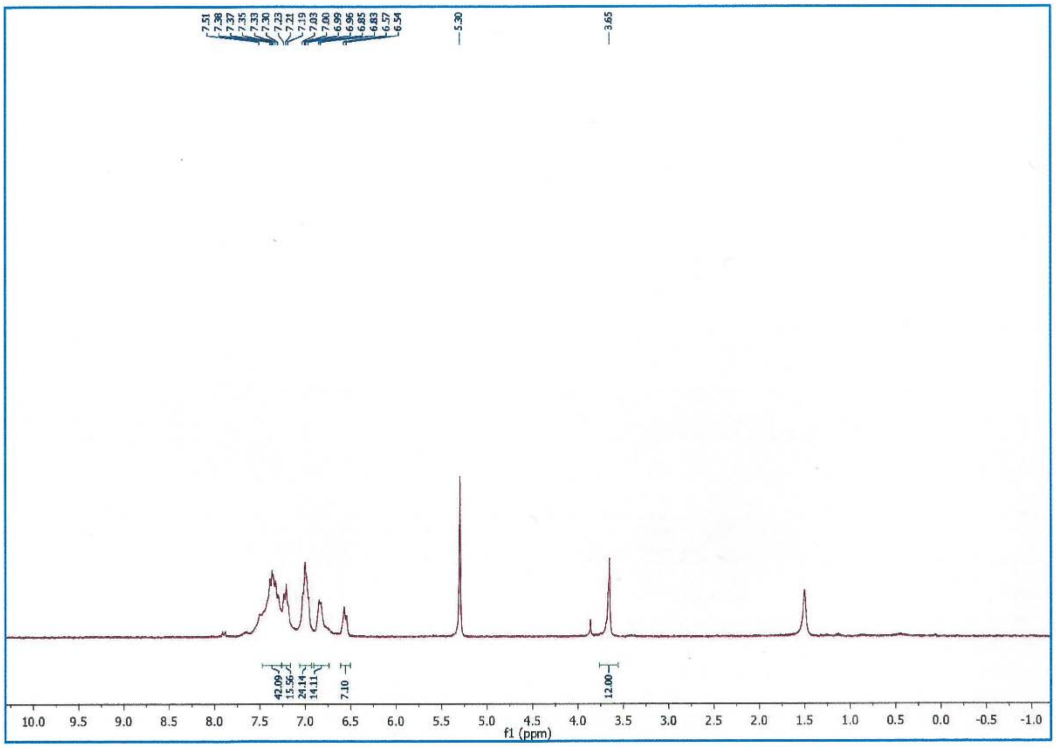

Figure S80. ^{1}H NMR spectrum (CD_2Cl_2) of [(adt)Ni(tpbz)Au(tpbz)Ni(adt)][Cl], [**7**][Cl].

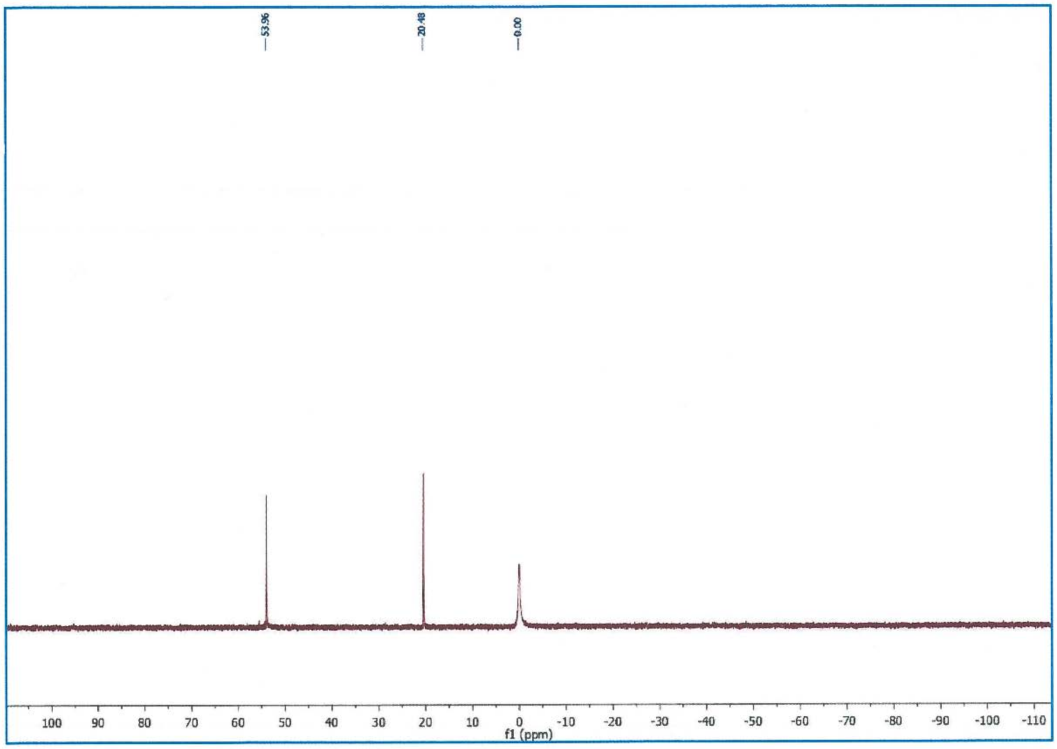

Figure S81. ^{31}P-{^{1}H} NMR spectrum (CD_2Cl_2) of [(adt)Ni(tpbz)Au(tpbz)Ni(adt)][Cl], [**7**][Cl].

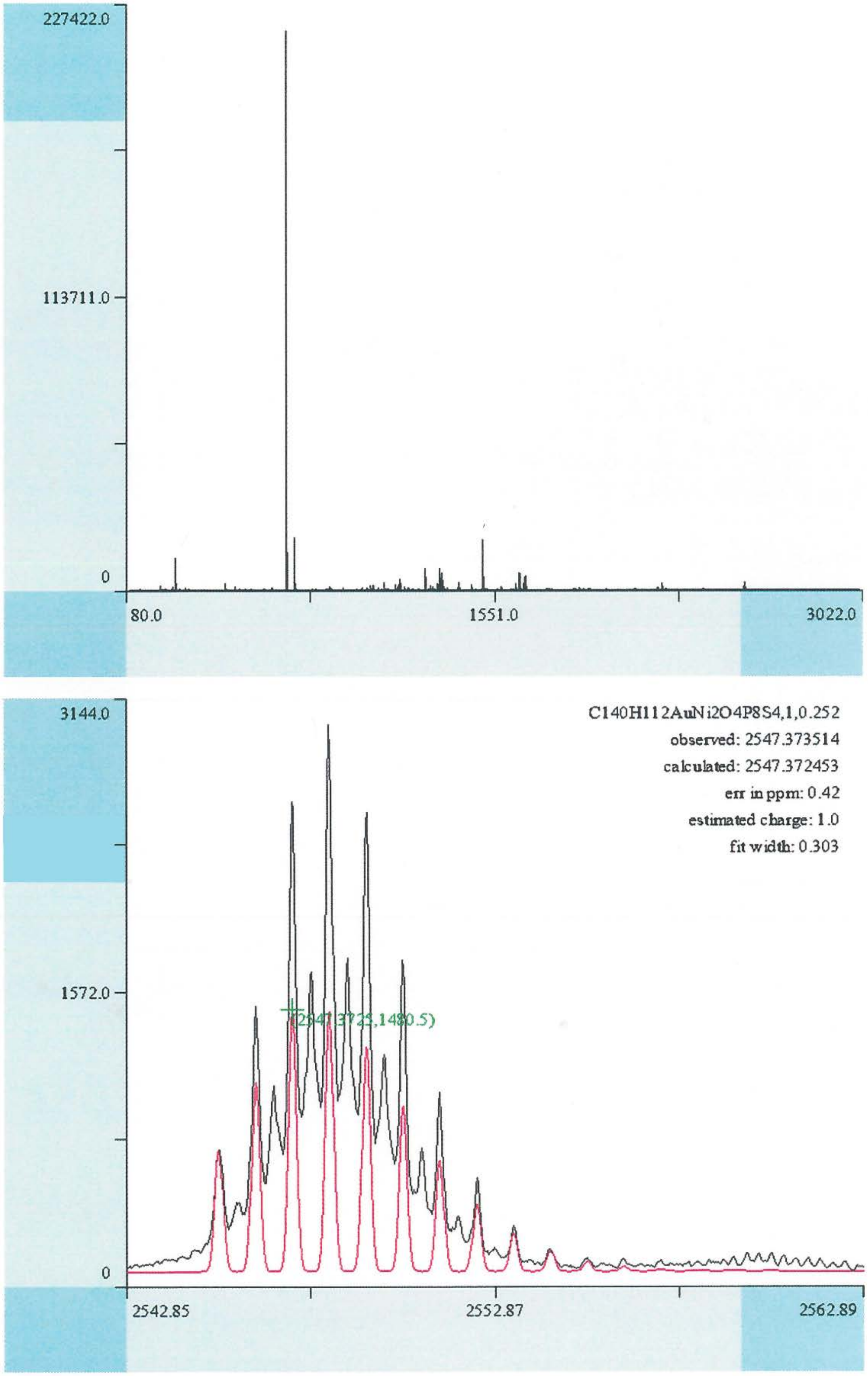

Figure S82. Mass spectrum (ESI+) of [(adt)Ni(tpbz)Au(tpbz)Ni(adt)][Cl], [**7**][Cl].

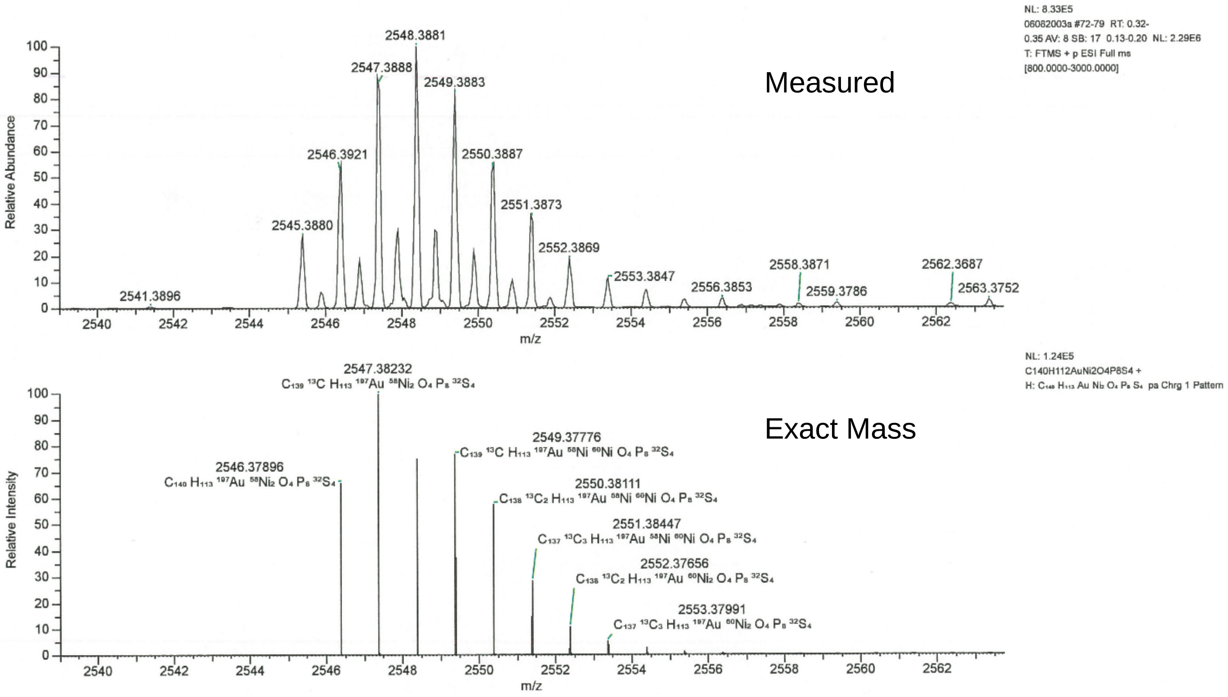

Figure S83. Mass spectrum (ESI+) of [(adt)Ni(tpbz)Au(tpbz)Ni(adt)][Cl], [**7**][Cl].

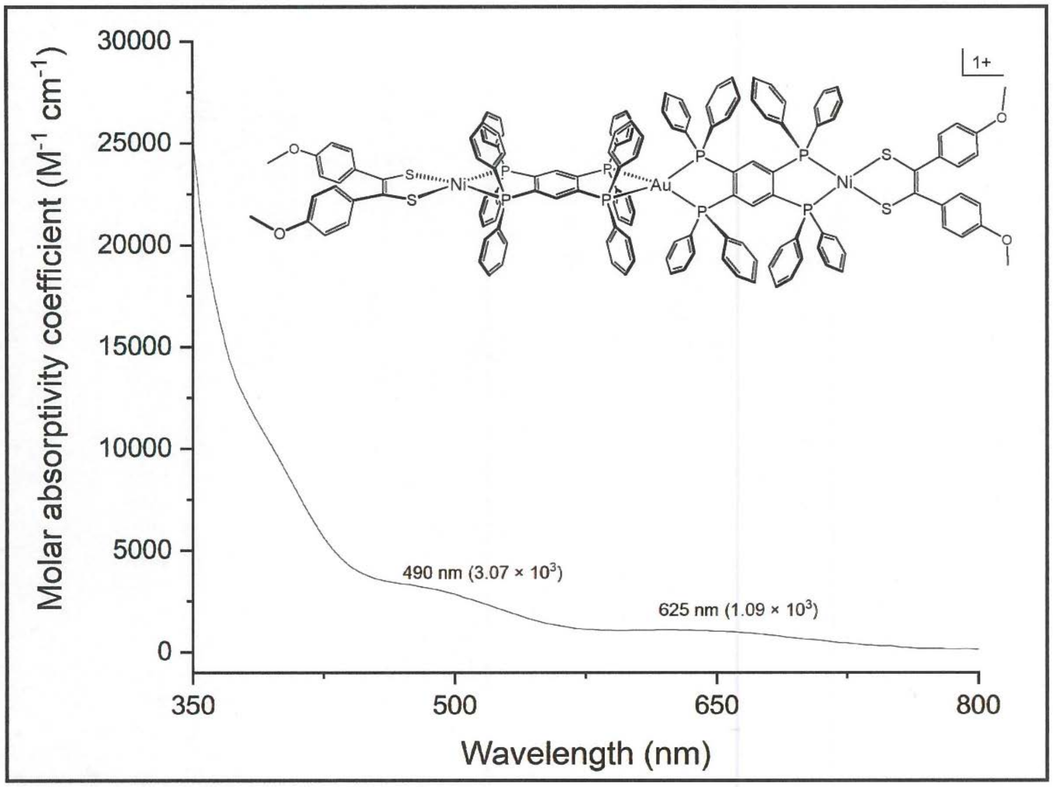

Figure S84. UV-vis spectrum (CH_2Cl_2) of [(adt)Ni(tpbz)Au(tpbz)Ni(adt)][Cl], [**7**][Cl].

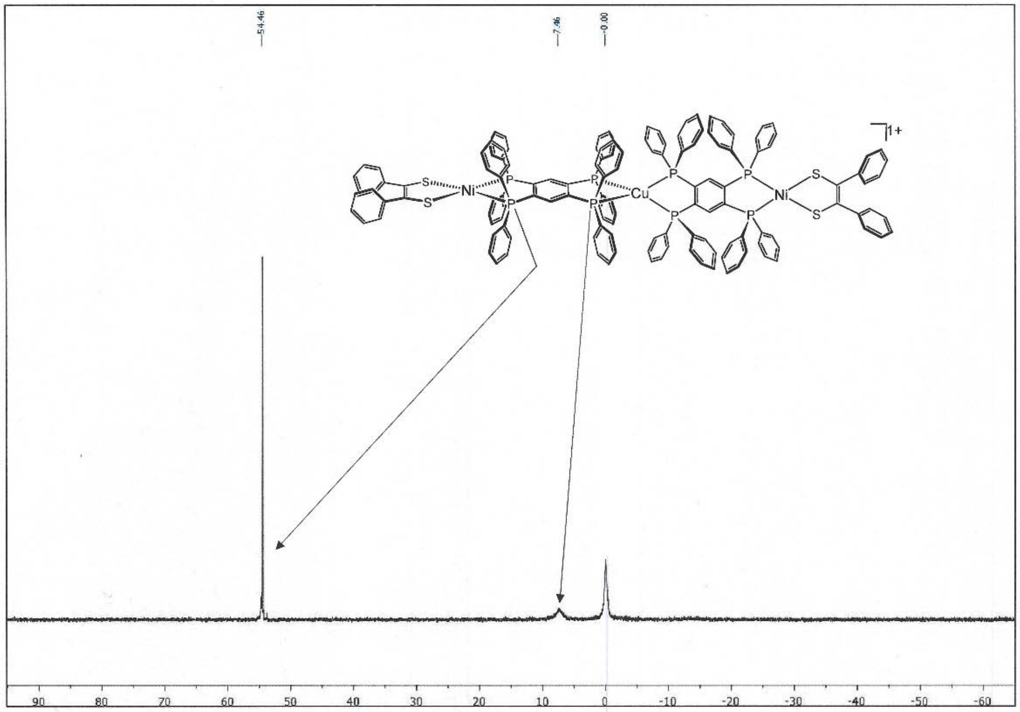

Figure S85. ^{31}P-{^{1}H} NMR spectrum (CD_2Cl_2) of crude [(pdt)Ni(tpbz)Cu(tpbz)Ni(pdt)]$^{1+}$.

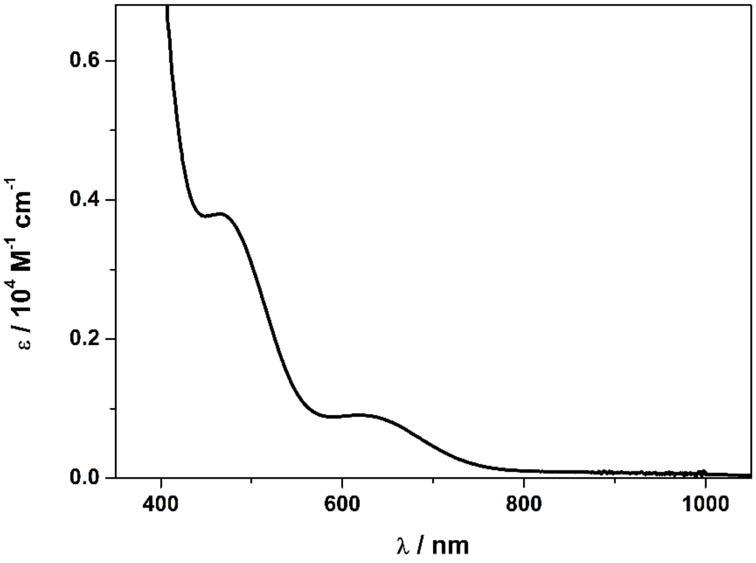

Figure S86. Electronic spectrum of [($Ph_2C_2S_2$)Ni(tpbz)Cu(tpbz)Ni($S_2C_2Ph_2$)]$^{+}$ recorded in CH_2Cl_2 solution at ambient temperature. Absorption maxima are as follows: λ_{max} / nm (ε / M^{-1} cm^{-1}): 620 (950), 464 (3840).

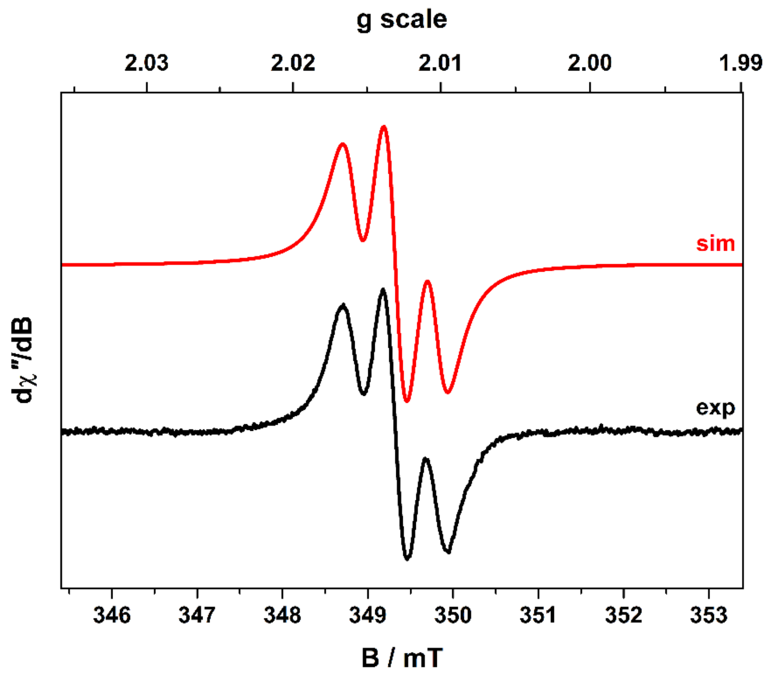

Figure S87. X-band EPR spectrum of **[Ni$_2$Cu]**$^{3+}$ recorded in CH_2Cl_2 solution at 293 K. Experimental data are shown by the black line, and simulations are depicted by the dashed red trace (conditions: frequency, 9.436 GHz; power, 0.63 mW; modulation, 0.1 mT).

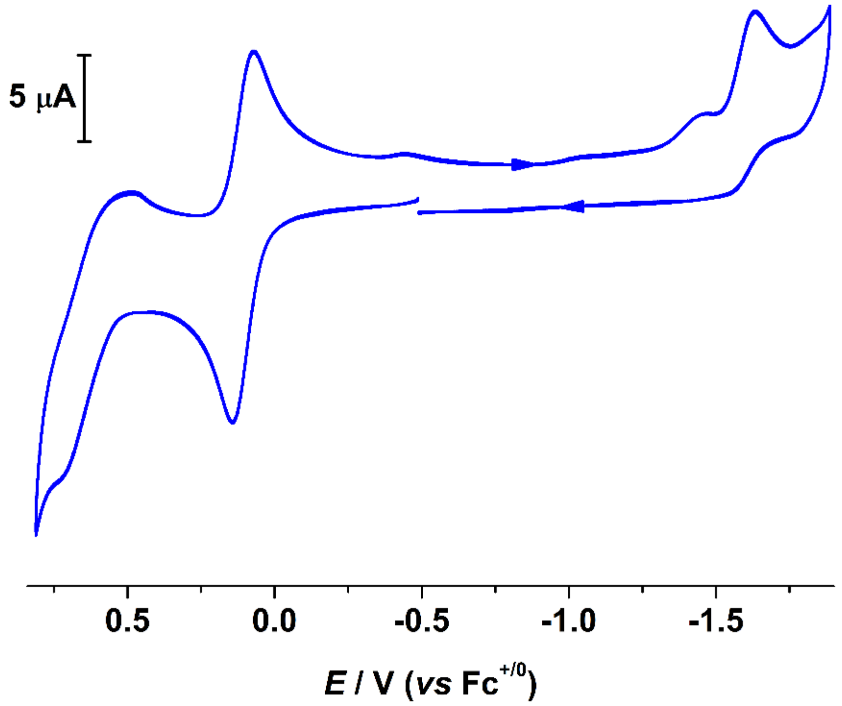

Figure S88. Cyclic voltammogram of $[(Ph_2C_2S_2)Ni(tpbz)Cu(tpbz)Ni(S_2C_2Ph_2)]^+$ in CH_2Cl_2 solution (0.10 *M* $[^nBu_4N][PF_6]$ supporting electrolyte) at 22 °C at a scan rate of 100 mV s^{-1}. Potentials are referenced versus the $Fc^{+/0}$ couple: $E_{1/2}$(**[Ni$_2$Cu]**$^{3+/1+}$) = +0.106 V (E_{pp} = 72 mV), $E_{1/2}$(**[Ni$_2$Cu]**$^{5+/3+}$) = +0.652 V irr., E_{pc}(**[Ni$_2$Cu]**$^{1+/0}$) = –1.46 V, E_{pc}(**[Ni$_2$Cu]**$^{0/1-}$) = –1.63 V

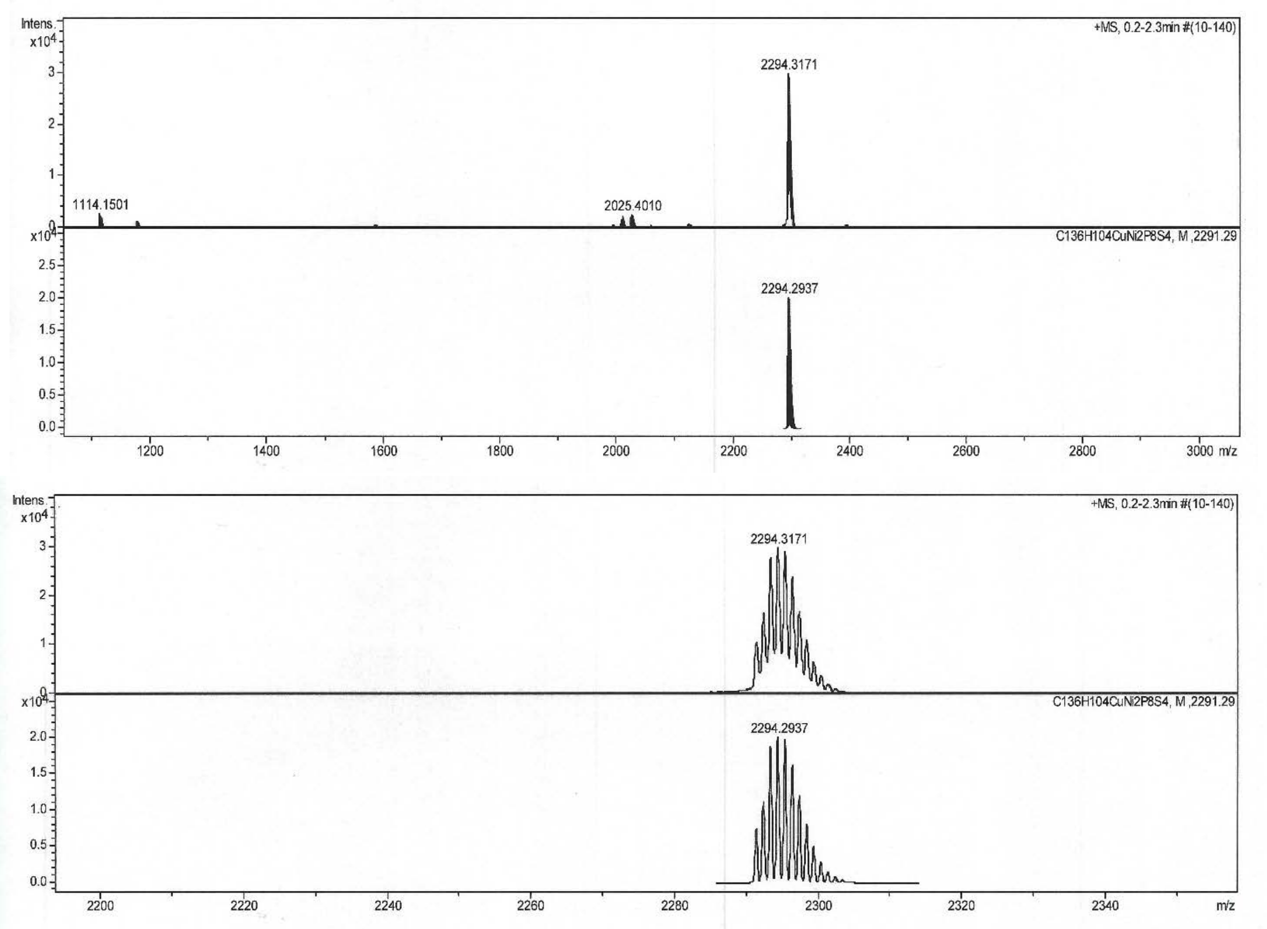

$$\frac{|2294.3171\ - 2294.2937|}{2294.2937}\ x\ 1E6$$

10.2 ppm difference

Figure S89. Mass spectrum (ESI+) of $[(Ph_2C_2S_2)Ni(tpbz)Cu(tpbz)Ni(S_2C_2Ph_2)]^+$, $C_{136}H_{104}P_8S_4Ni_2Cu$.

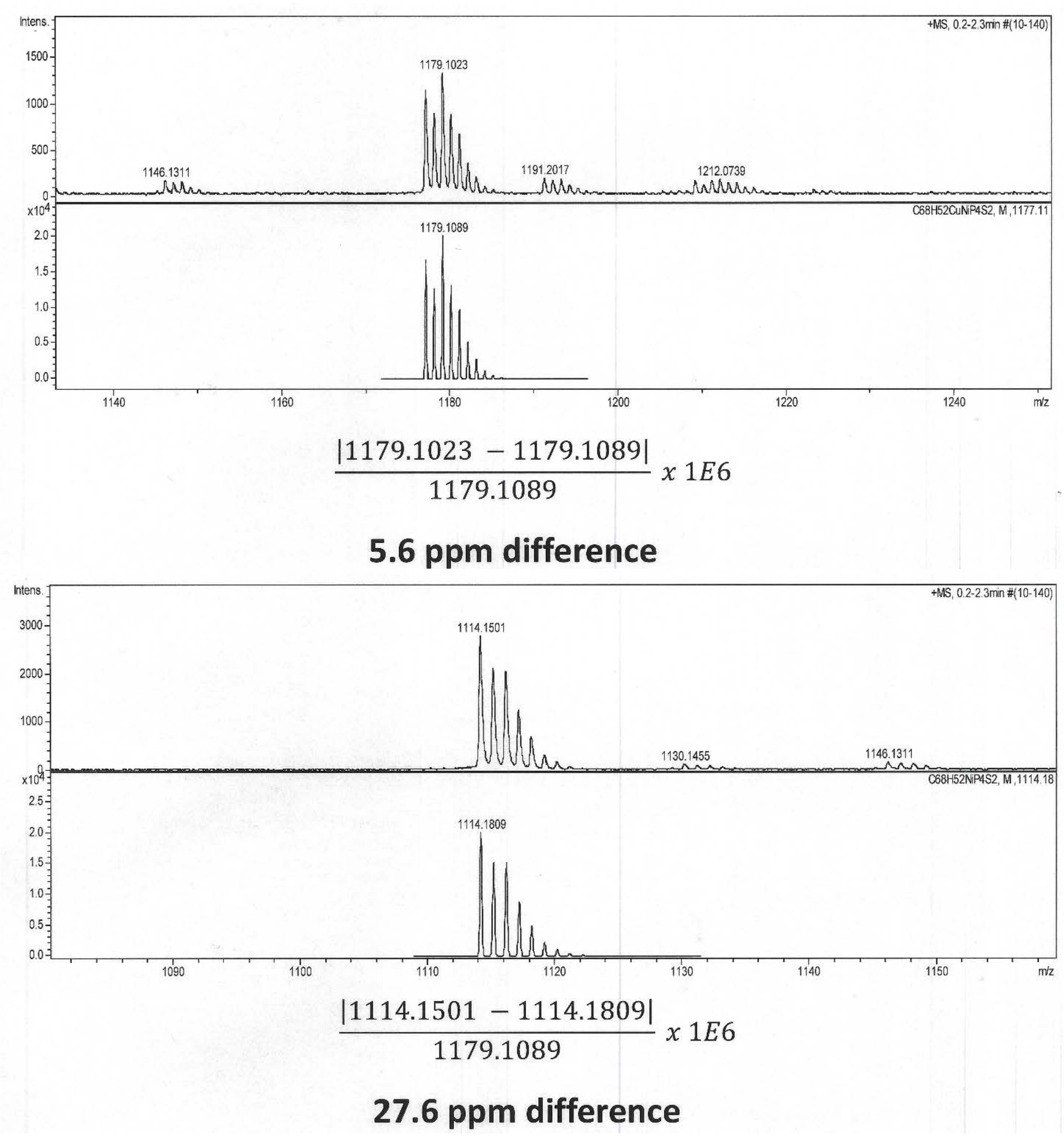

Figure S90. Mass spectrum (ESI+) of $[(Ph_2C_2S_2)Ni(tpbz)Cu(tpbz)Ni(S_2C_2Ph_2)]^+$ showing fragment peaks corresponding to $[(Ph_2C_2S_2)Ni(tpbz)Cu]^+$ (top) and $[(Ph_2C_2S_2)Ni(tpbz)]^+$ (bottom).

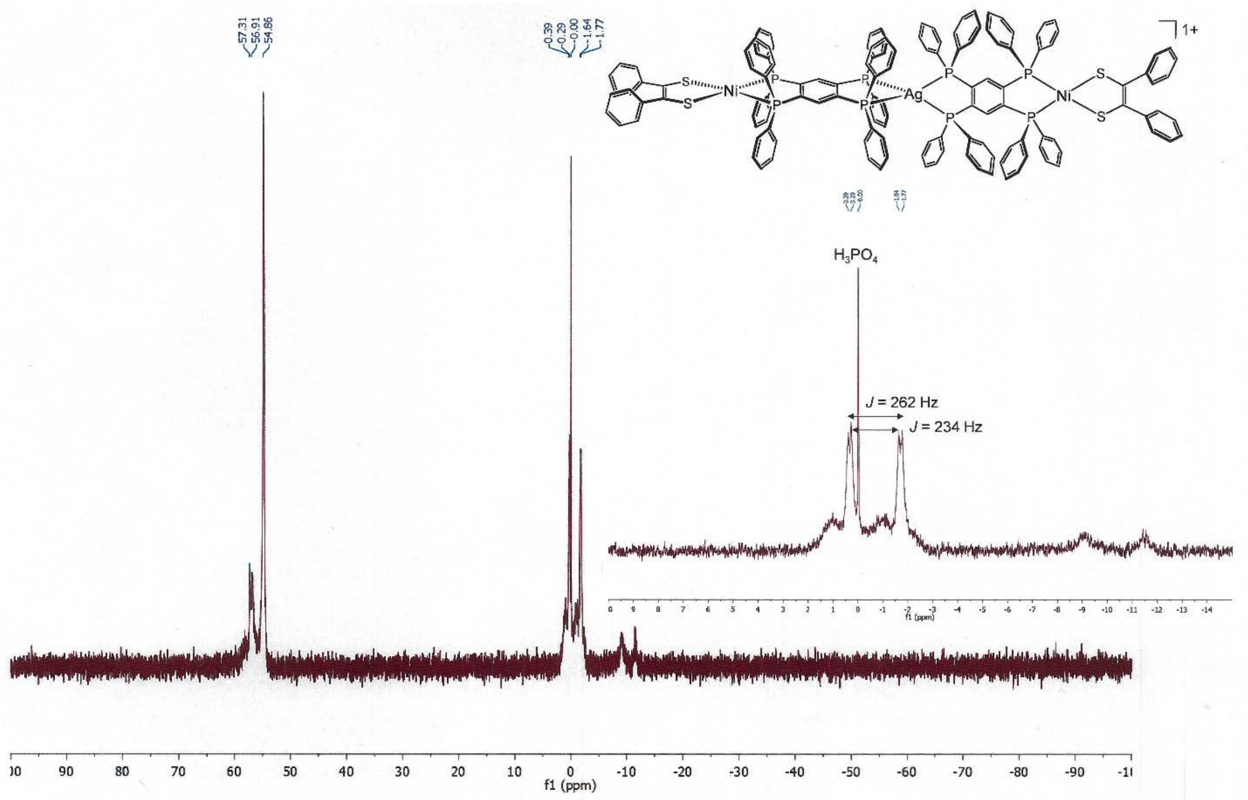

Figure S91. ^{1}H NMR spectrum ($CDCl_3$) of [(pdt)Ni(tpbz)Ag(tpbz)Ni(pdt)]$^+$.

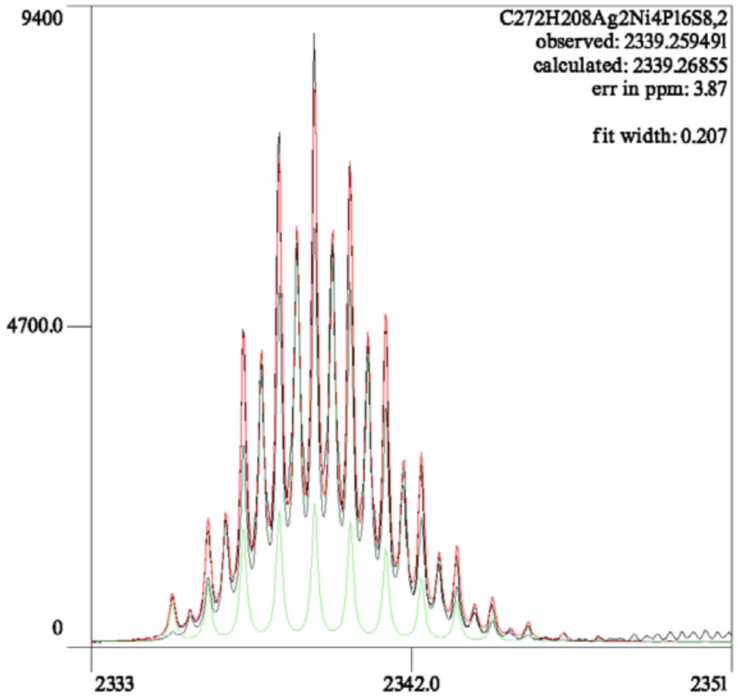

Figure S92. Mass spectrum (ESI+) of [(pdt)Ni(tpbz)Ag(tpbz)Ni(pdt)]$^+$. The ESI-MS fit is described as a mixture of the monocation and a dimer dication.

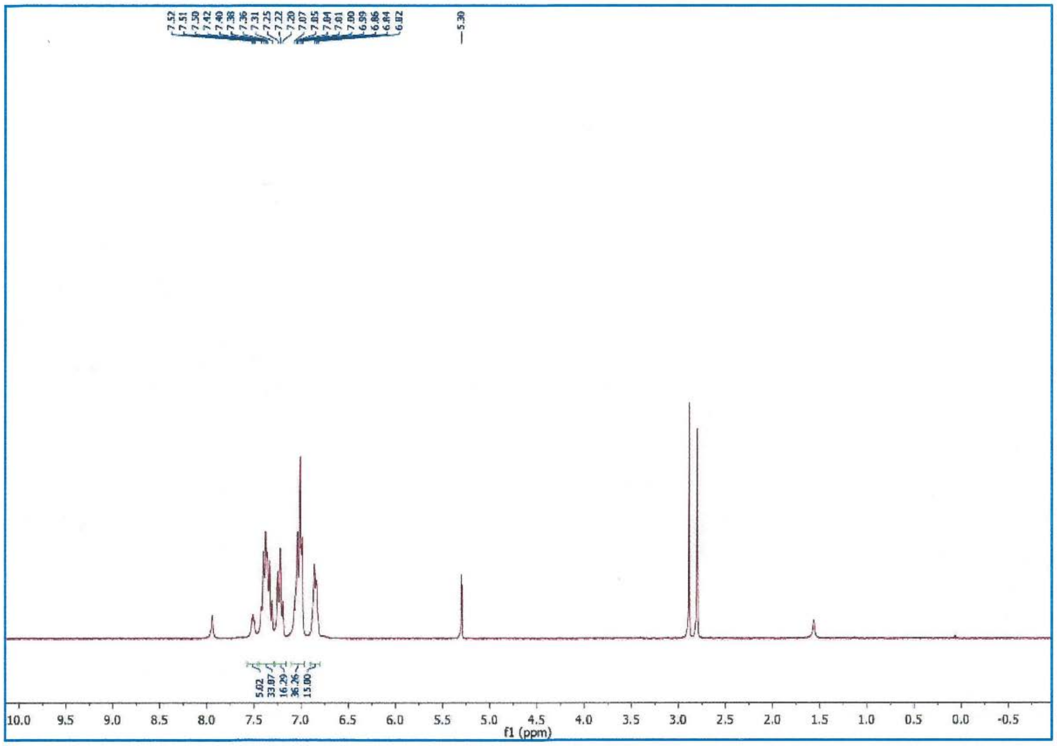

Figure S93. ^{1}H NMR spectrum (CD_2Cl_2) of [(pdt)Ni(tpbz)Au(tpbz)Ni(pdt)][CF_3SO_3], [**10**][CF_3SO_3].

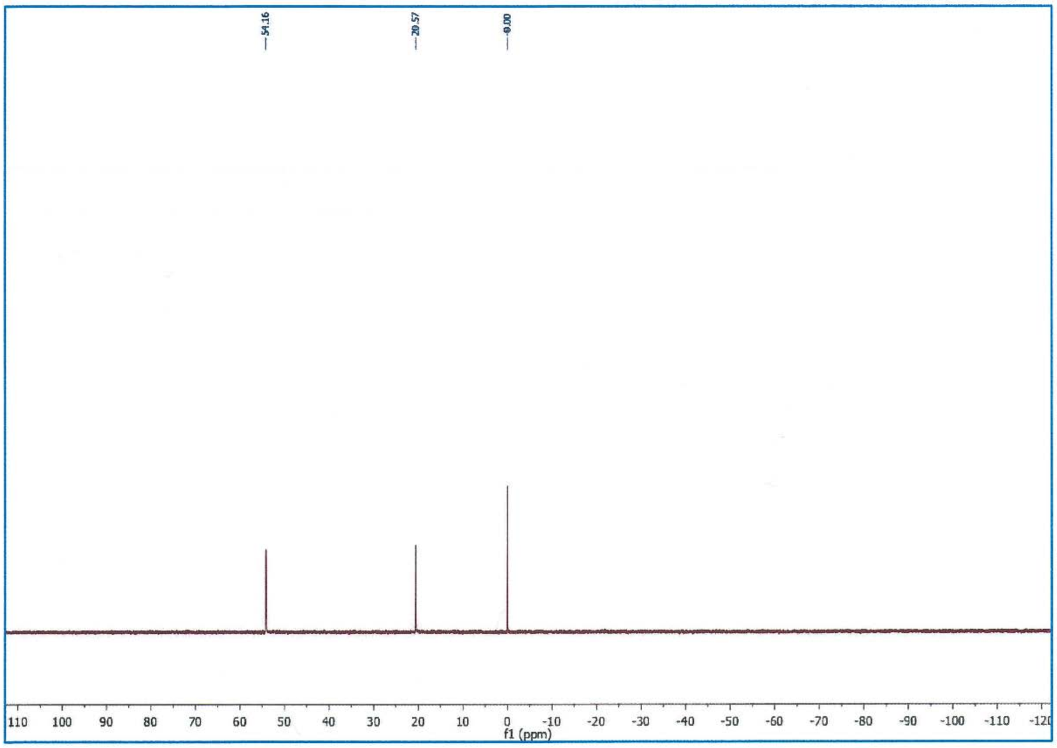

Figure S94. ^{31}P-{^{1}H} NMR spectrum (CD_2Cl_2) of [(pdt)Ni(tpbz)Au(tpbz)Ni(pdt)][CF_3SO_3], [**10**][CF_3SO_3].

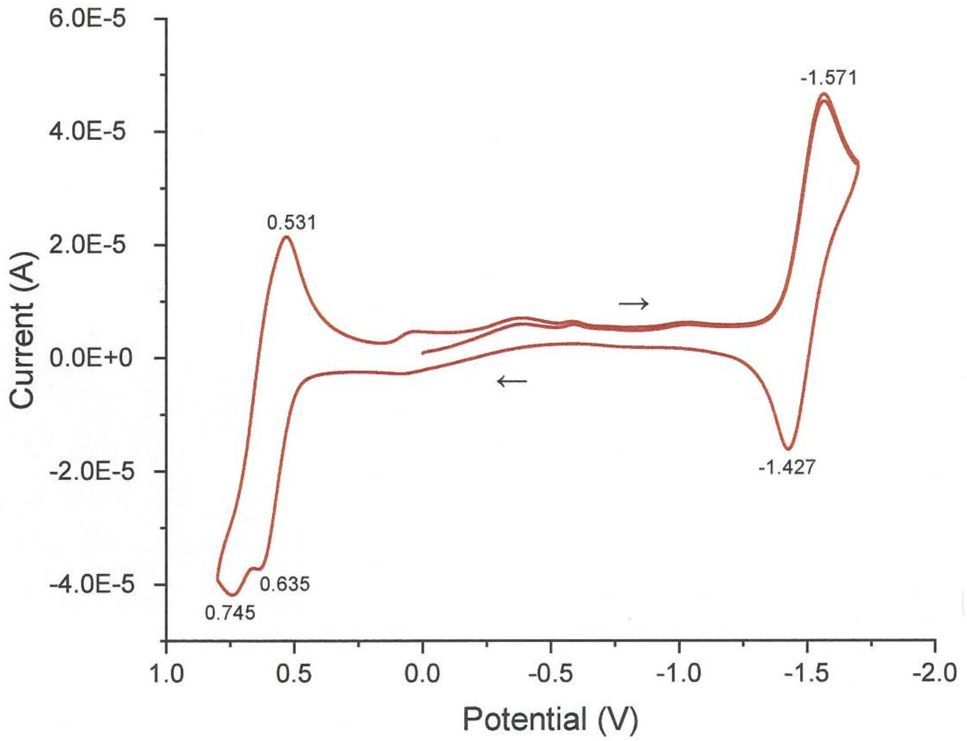

Figure S95. Cyclic voltammogram of [(pdt)Ni(tpbz)Au(tpbz)Ni(pdt)][CF_3SO_3], [**10**][CF_3SO_3], in CH_2Cl_2 at 25 °C using [^{n}Bu$_4$N][PF_6] as supporting electrolyte. The reference electrode is Ag/AgCl, and the scan rate is 100 mV/sec.

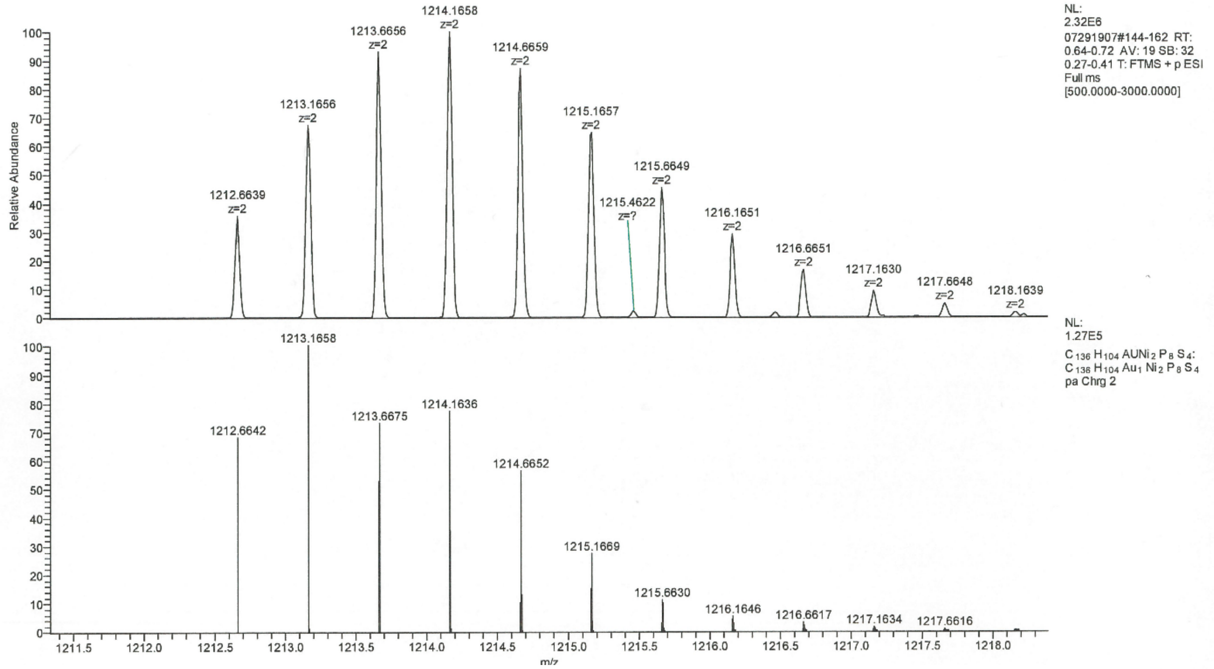

Figure S96. Mass spectrum (ESI+) of [(pdt)Ni(tpbz)Au(tpbz)Ni(pdt)][CF_3SO_3], [**10**][CF_3SO_3], with 2+ charge.

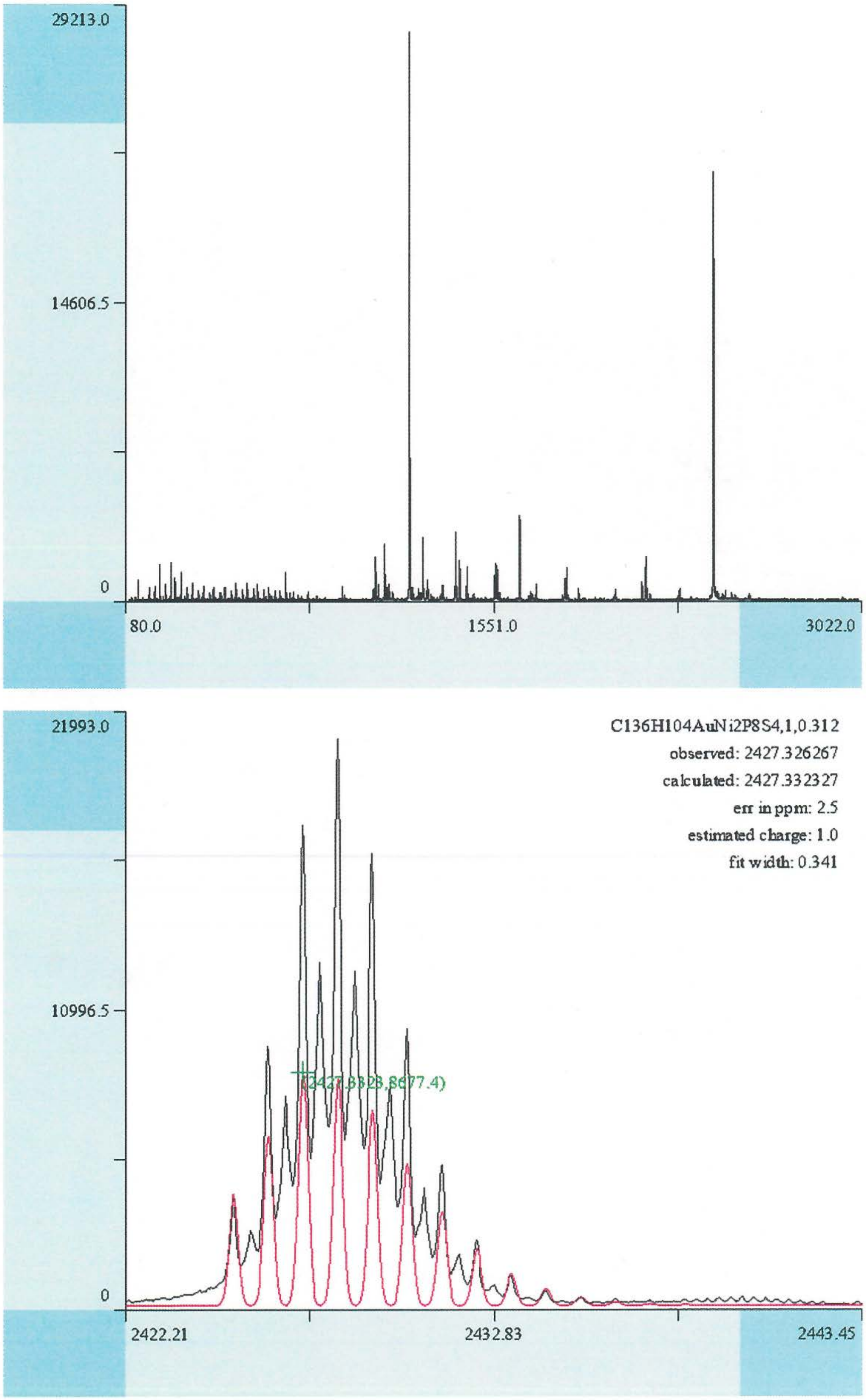

Figure S97. Mass spectrum (ESI+) of [(pdt)Ni(tpbz)Au(tpbz)Ni(pdt)][CF_3SO_3], [**10**][CF_3SO_3].

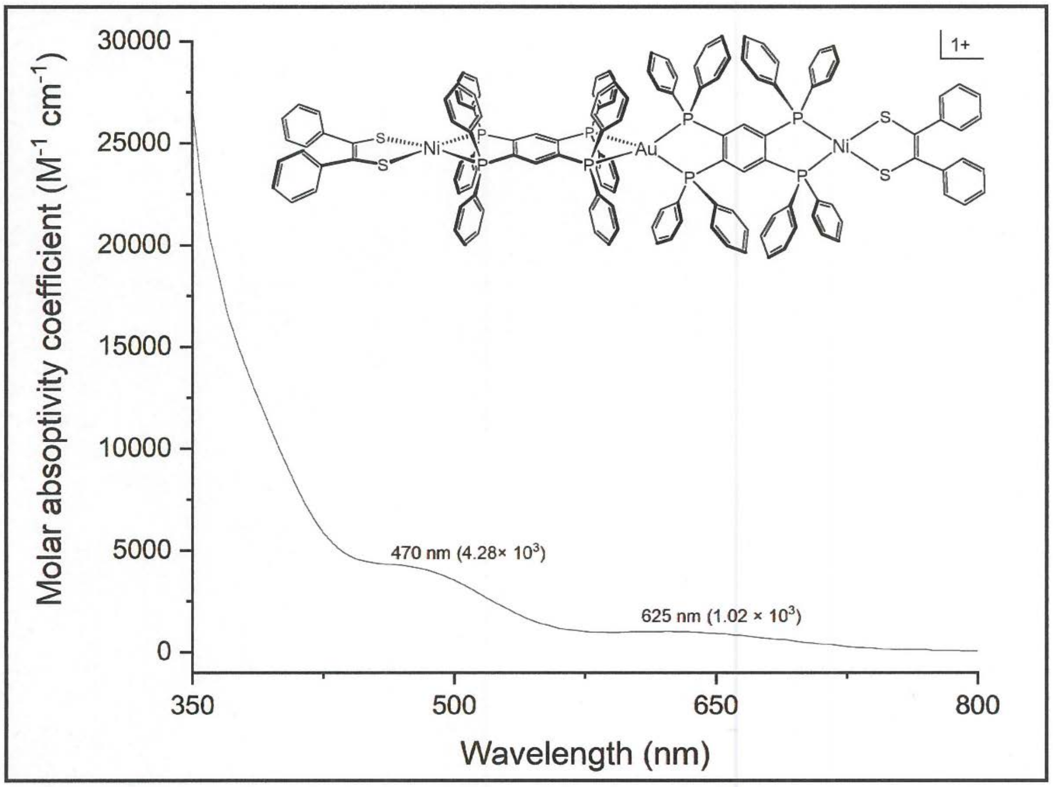

Figure S98. UV-vis spectrum (CH_2Cl_2) of [(pdt)Ni(tpbz)Au(tpbz)Ni(pdt)][CF_3SO_3], [**10**][CF_3SO_3].

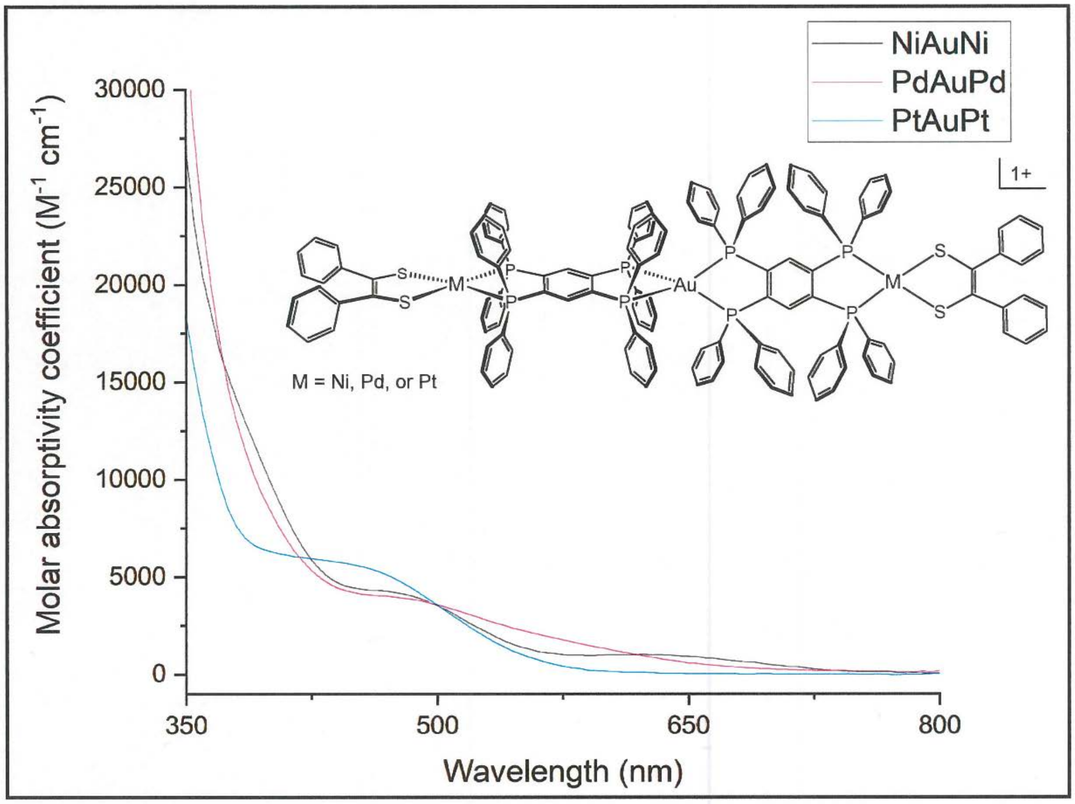

Figure S99. Overlay of the UV-vis spectra (CH_2Cl_2) of [(pdt)Ni(tpbz)Au(tpbz)Ni(pdt)][CF_3SO_3], [(pdt)Pd(tpbz)Au(tpbz)Pd(pdt)][CF_3SO_3] and [(pdt)Pt(tpbz)Au(tpbz)Pt(pdt)][CF_3SO_3].

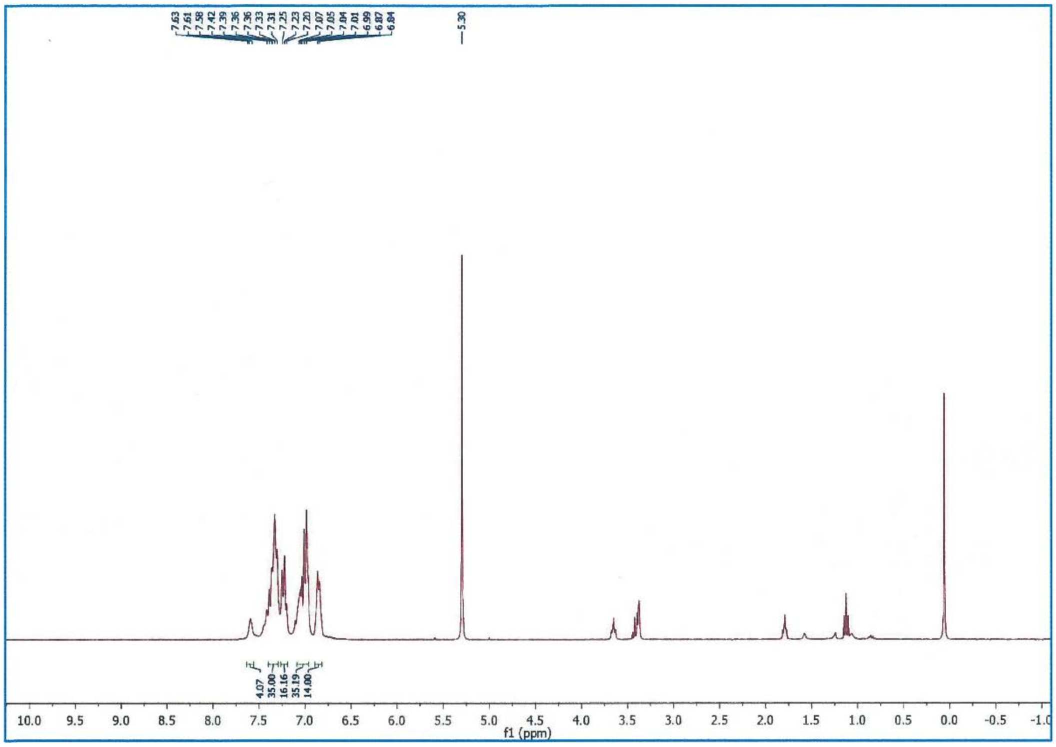

Figure S100. ^{1}H NMR spectrum (CD_2Cl_2) of [(pdt)Pd(tpbz)Au(tpbz)Pd(pdt)][CF_3SO_3], [**11**][CF_3SO_3].

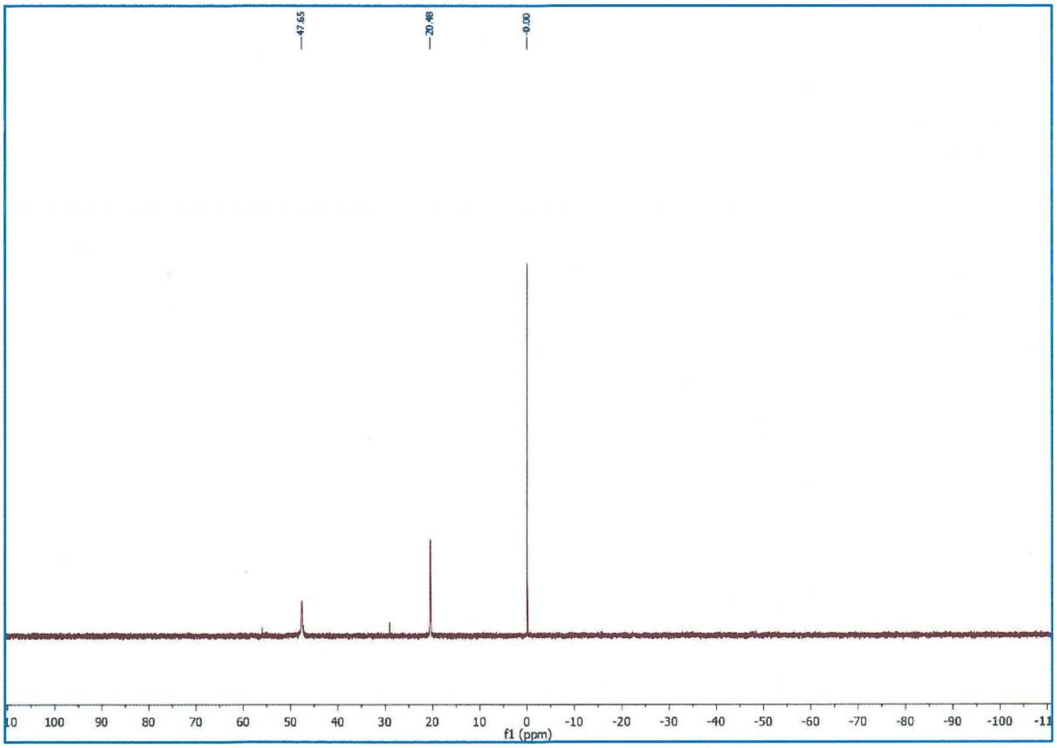

Figure S101. ^{31}P-{^{1}H} NMR spectrum (CD_2Cl_2) of [(pdt)Pd(tpbz)Au(tpbz)Pd(pdt)][CF_3SO_3], [**11**][CF_3SO_3].

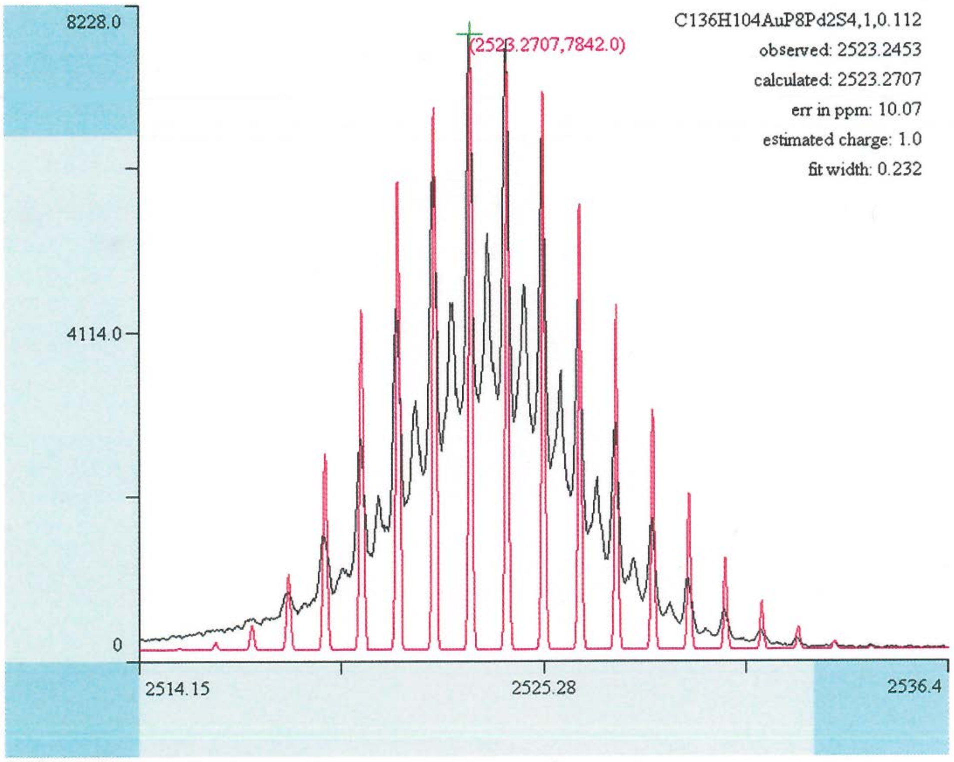

Figure S102. Mass spectrum (ESI+) of [(pdt)Pd(tpbz)Au(tpbz)Pd(pdt)][CF_3SO_3], [**11**][CF_3SO_3].

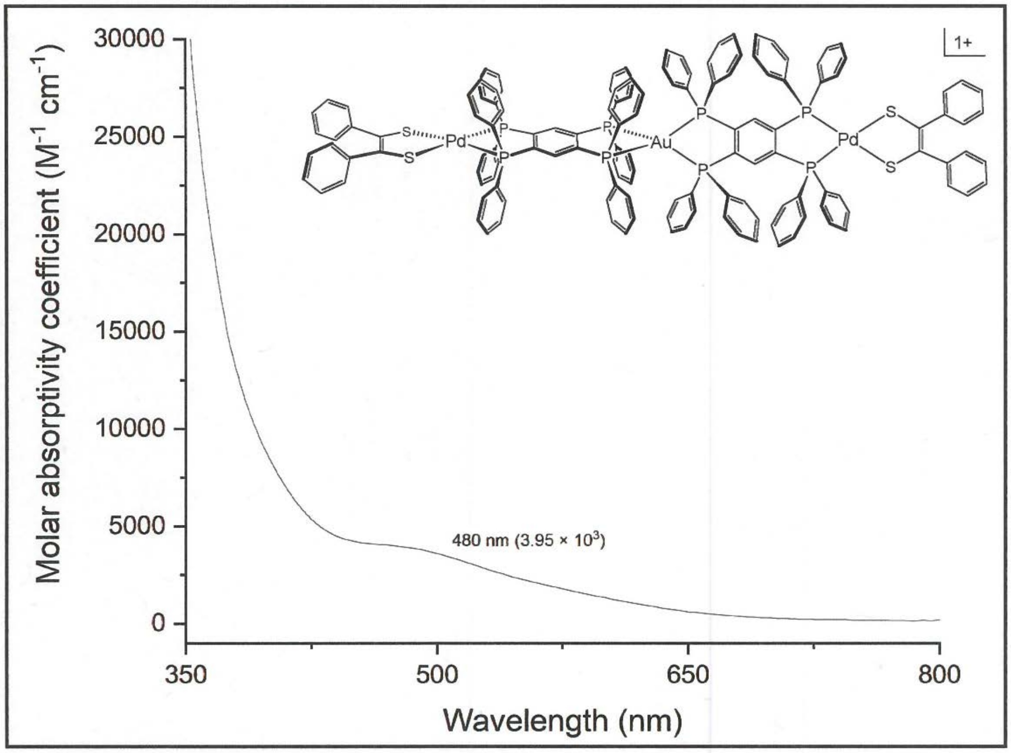

Figure S103. UV-vis spectrum (CH_2Cl_2) of [(pdt)Pd(tpbz)Au(tpbz)Pd(pdt)][CF_3SO_3], [**11**][CF_3SO_3].

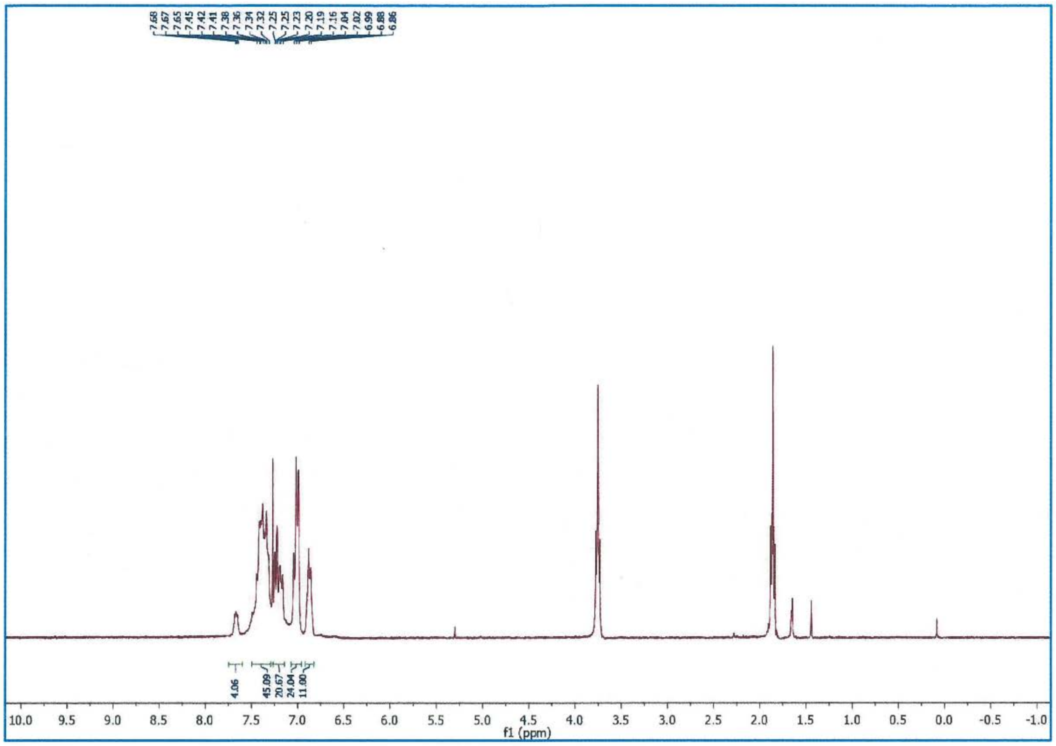

Figure S104. ^{1}H NMR spectrum (CD_2Cl_2) of [(pdt)Pt(tpbz)Au(tpbz)Pt(pdt)][CF_3SO_3], [**12**][CF_3SO_3].

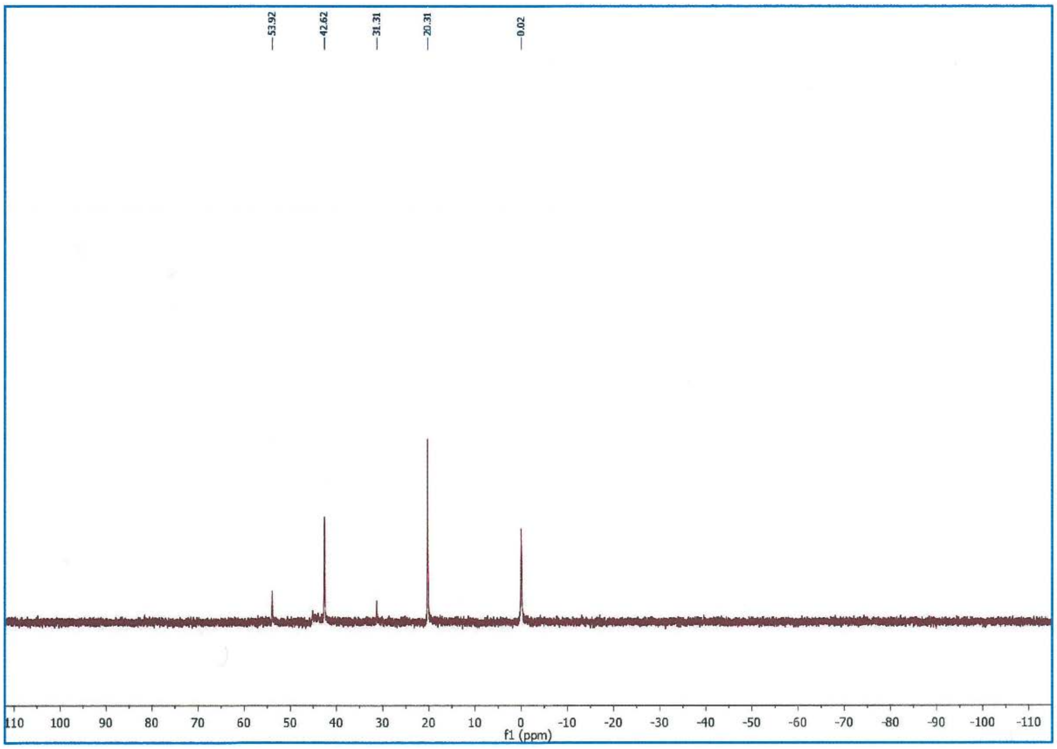

Figure S105. ^{31}P-{^{1}H} NMR spectrum (CD_2Cl_2) of [(pdt)Pt(tpbz)Au(tpbz)Pt(pdt)][CF_3SO_3], [**12**][CF_3SO_3].

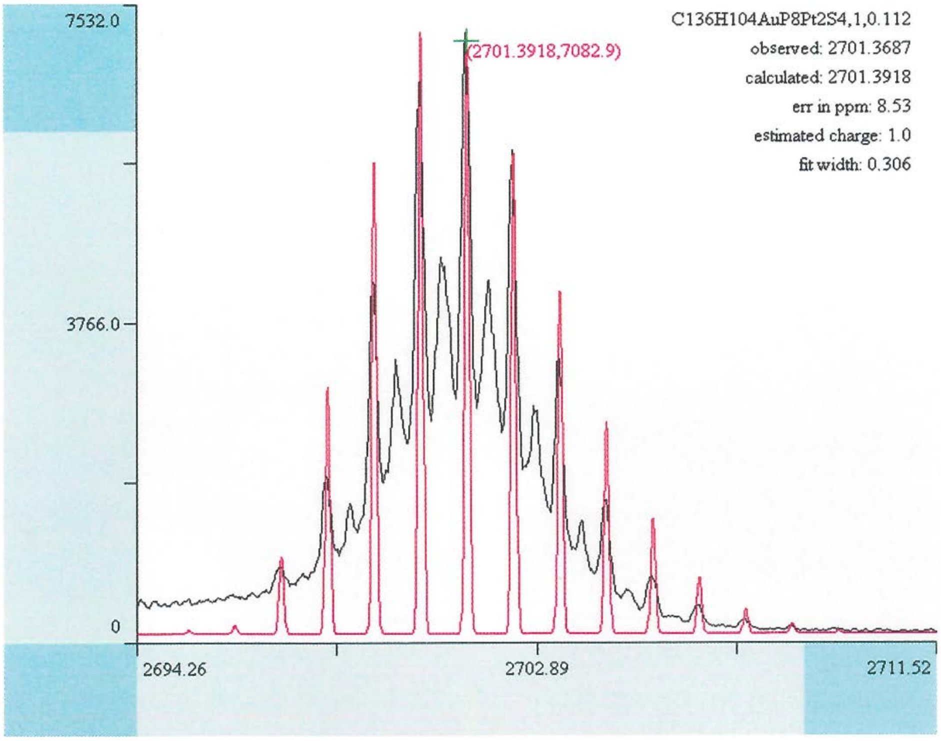

Figure S106. Mass spectrum (ESI+) of [(pdt)Pt(tpbz)Au(tpbz)Pt(pdt)][CF_3SO_3], [**12**][CF_3SO_3].

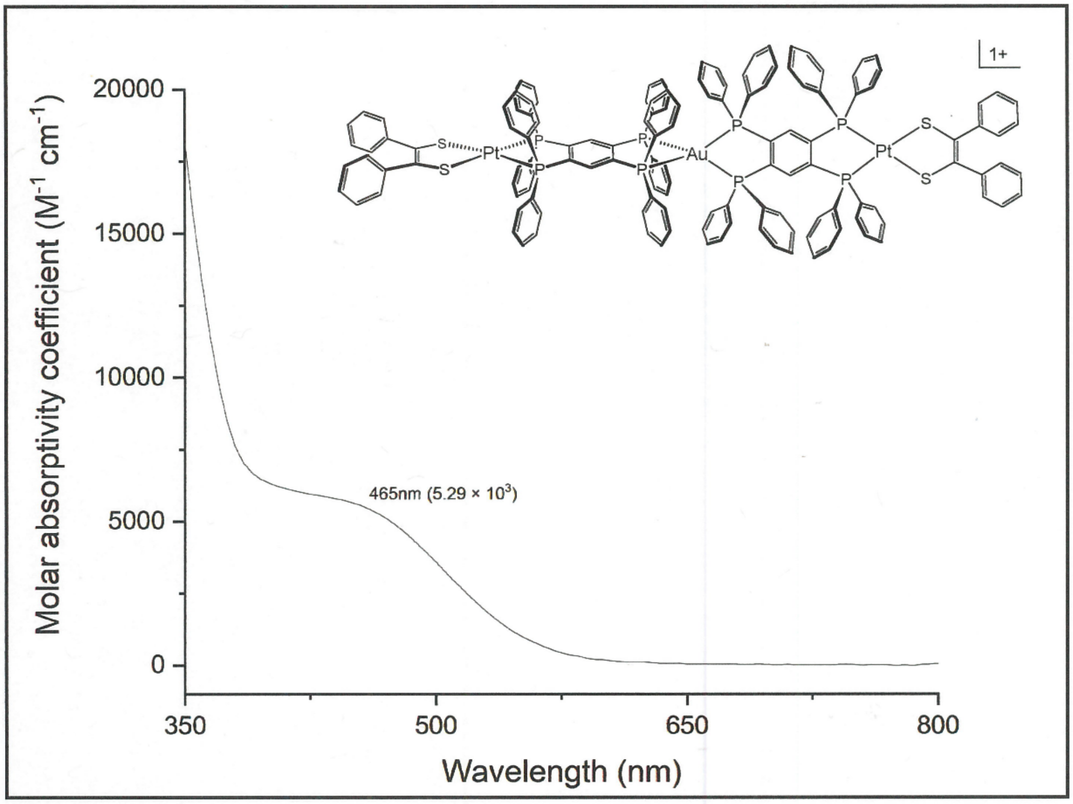

Figure S107. UV-vis spectrum (CH_2Cl_2) of [(pdt)Pt(tpbz)Au(tpbz)Pt(pdt)][CF_3SO_3], [**12**][CF_3SO_3].

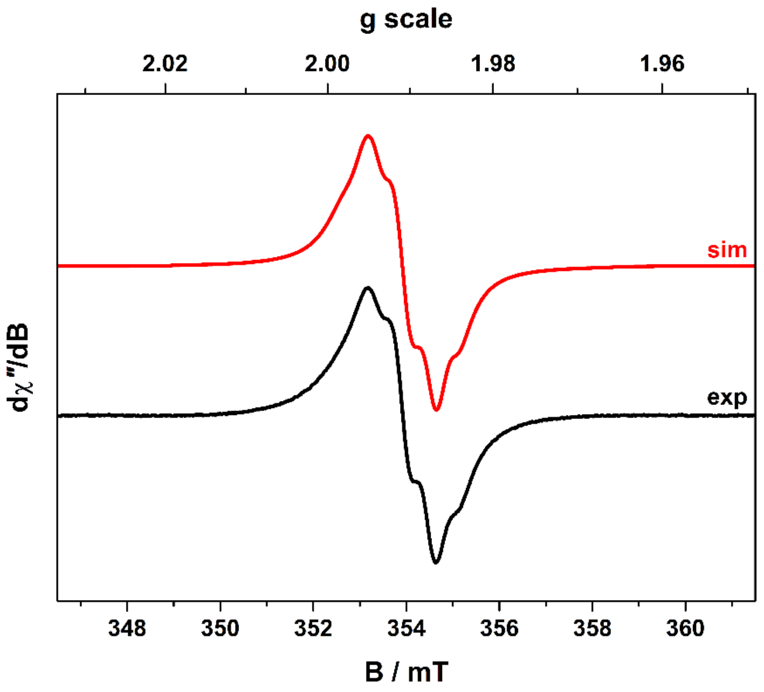

Figure S108. X-band EPR spectrum of **[Pt$_2$Au]**$^{3+}$ recorded in CH_2Cl_2 solution at 293 K. Experimental data are shown by the black line, and simulations are depicted by the dashed red trace (conditions: frequency, 9.436 GHz; power, 0.63 mW; modulation, 0.1 mT). Simulation: g_{iso} = 1.9911; $A\{^{195}Pt\}$ = 12.0 × 10^{-4} cm^{-1}; $A\{^{31}P\}$ = 4.4 × 10^{-4} cm^{-1}.

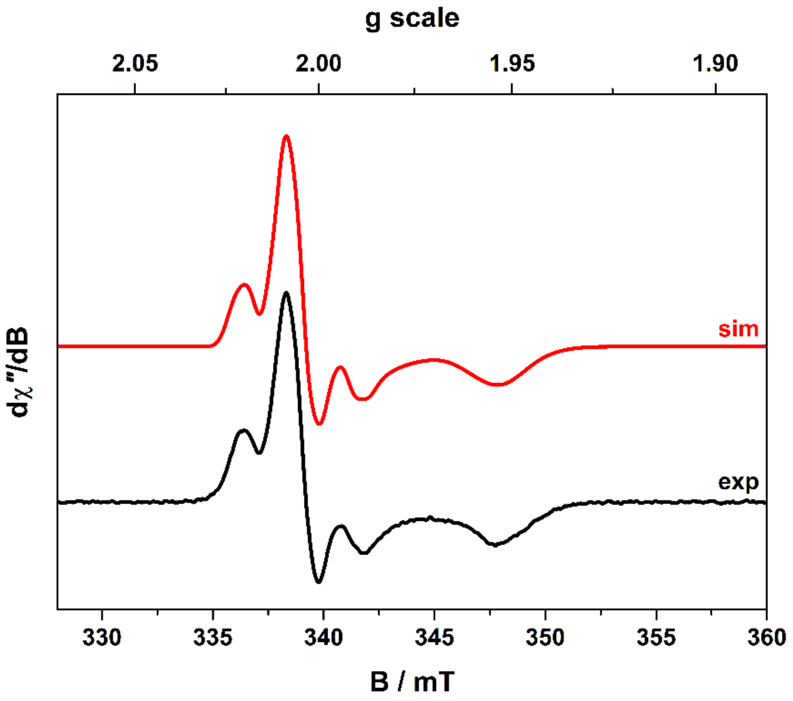

Figure S109. X-band EPR spectrum of **[Pt$_2$Au]**$^{3+}$ recorded in CH_2Cl_2 solution at 130 K. Experimental data are shown by the black line, and simulations are depicted by the dashed red trace (conditions: frequency, 9.436 GHz; power, 0.63 mW; modulation, 0.1 mT). Simulation: g = (2.0085, 2.0042, 1.9530); $\langle g \rangle$ = 1.9886, $A\{^{195}Pt\}$ = (34, 39, –25) × 10^{-4} cm^{-1}; $\langle A \rangle_{Pt}$ = 16 × 10^{-4} cm^{-1}, $A\{^{31}P\}$ = (6, 6, 5) × 10^{-4} cm^{-1}; $\langle A \rangle_{P}$ = 5.7 × 10^{-4} cm^{-1}.

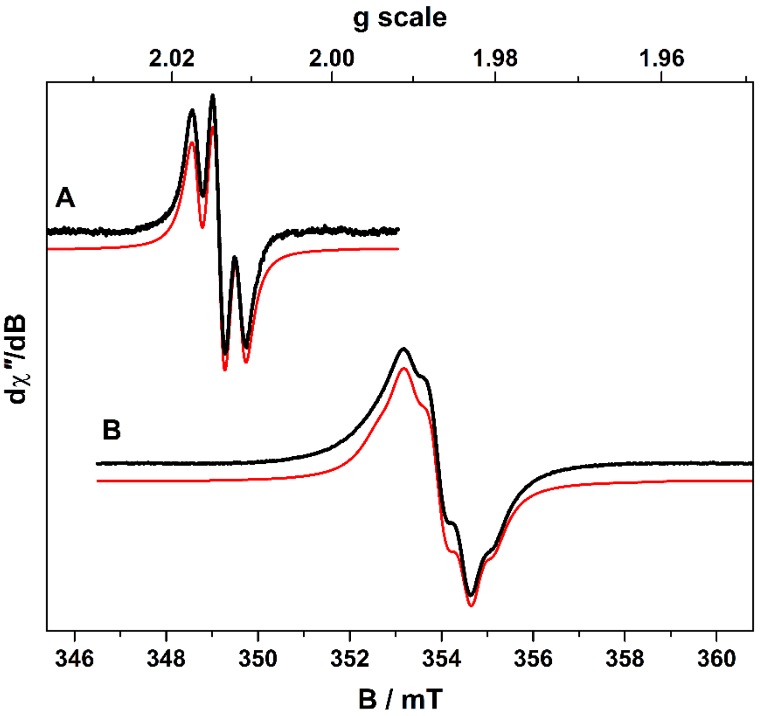

Figure S110. Comparison of the X-band EPR spectra of (a) **[Ni$_2$Cu]**$^{3+}$ and (b) **[Pt$_2$Au]**$^{3+}$ recorded in CH_2Cl_2 solution at 293 K. Experimental data are shown by the black line, and simulations are depicted by the dashed red trace (conditions: frequency, 9.436 GHz; power, 0.63 mW; modulation, 0.1 mT). Spin Hamiltonian parameters for **[Ni$_2$Cu]**$^{3+}$: g_{iso} = 2.0130; $A\{^{31}P\}$ = 4.5 × 10^{-4} cm^{-1}; Spin Hamiltonian parameters for **[Pt$_2$Au]**$^{3+}$: g_{iso} = 1.9911; $A\{^{195}Pt\}$ = 12.0 × 10^{-4} cm^{-1}; $A\{^{31}P\}$ = 4.4 × 10^{-4} cm^{-1}.

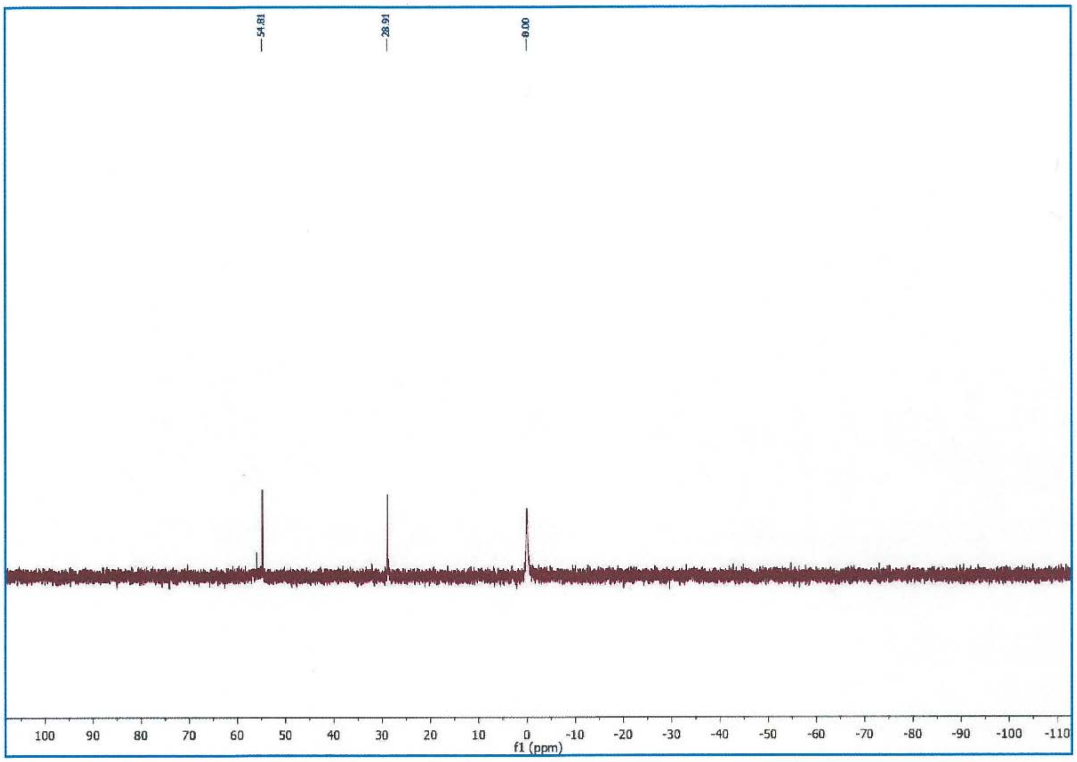

Figure S111. $^{31}P-\{^{1}H\}$ NMR spectrum ($CDCl_3$) of [(mnt)Ni(tpbz)ReBr(CO)$_3$], **13**.

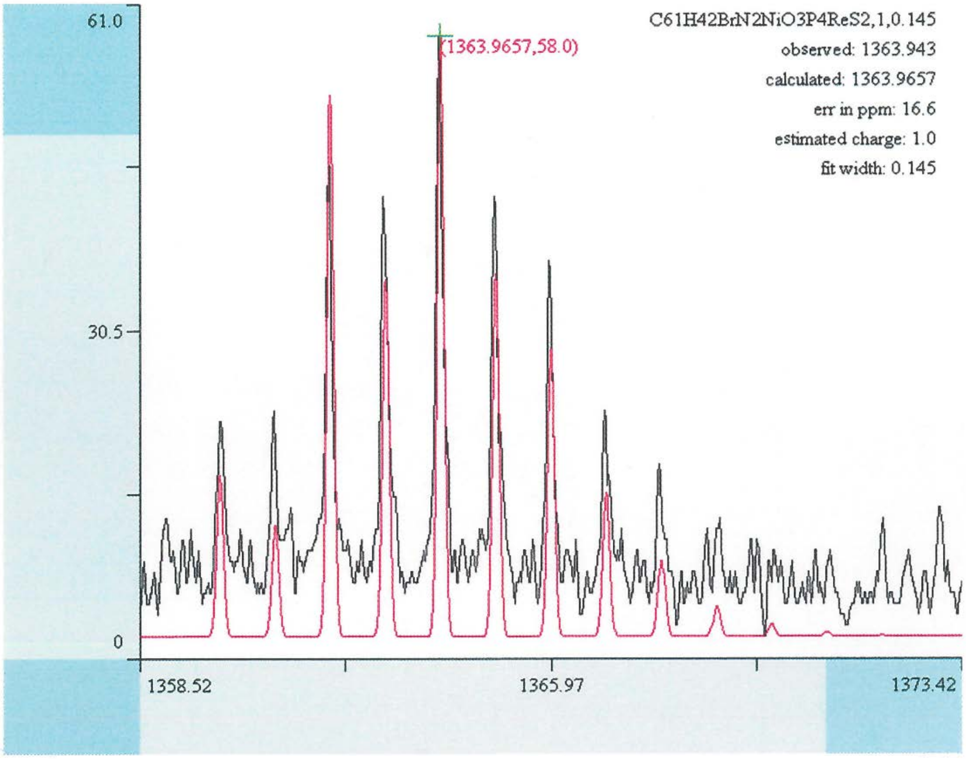

Figure S112. Mass spectrum (ESI+) of [(mnt)Ni(tpbz)ReBr(CO)$_3$], **13**.

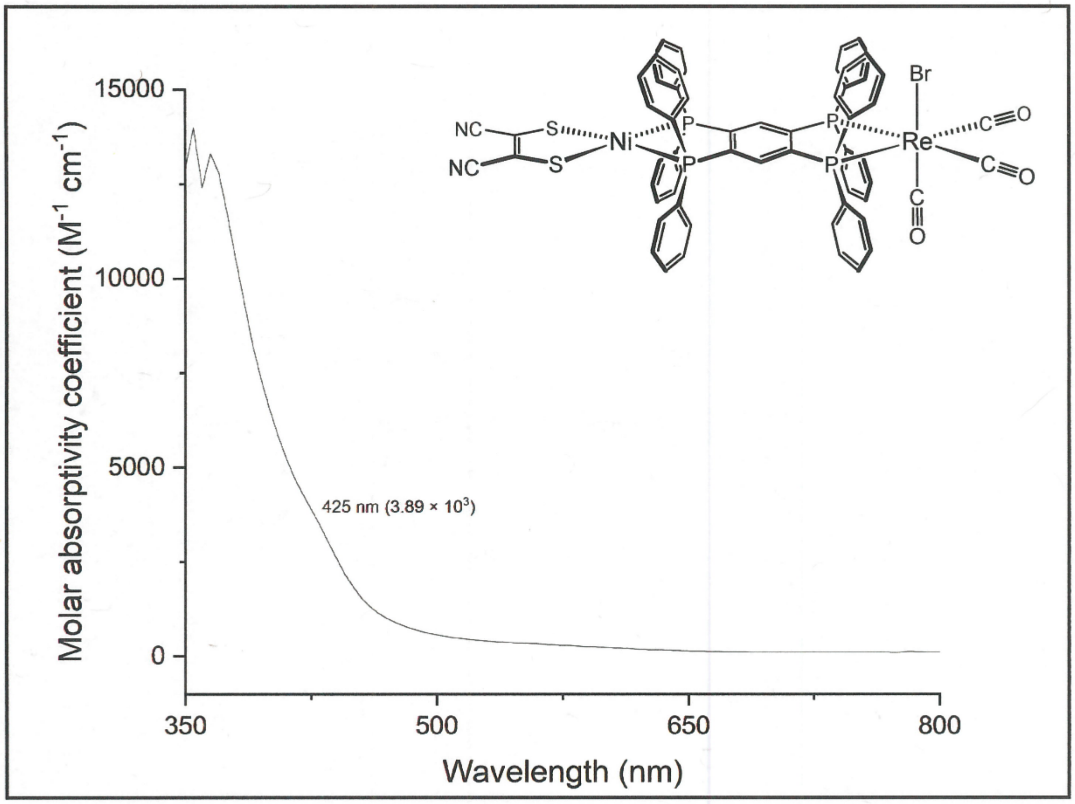

Figure S113. UV-vis spectrum (CH_2Cl_2) of [(mnt)Ni(tpbz)ReBr(CO)$_3$], **13**.

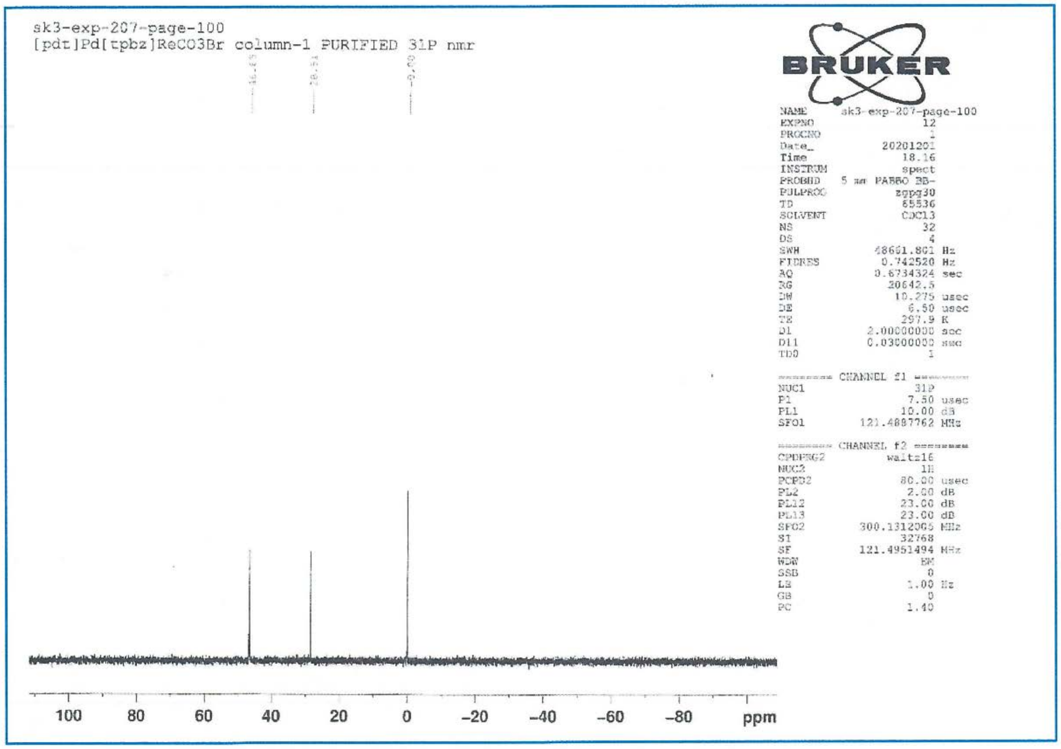

Figure S114. ^{31}P-{^{1}H} NMR spectrum ($CDCl_3$) of [(pdt)Pd(tpbz)ReBr(CO)$_3$], **14**.

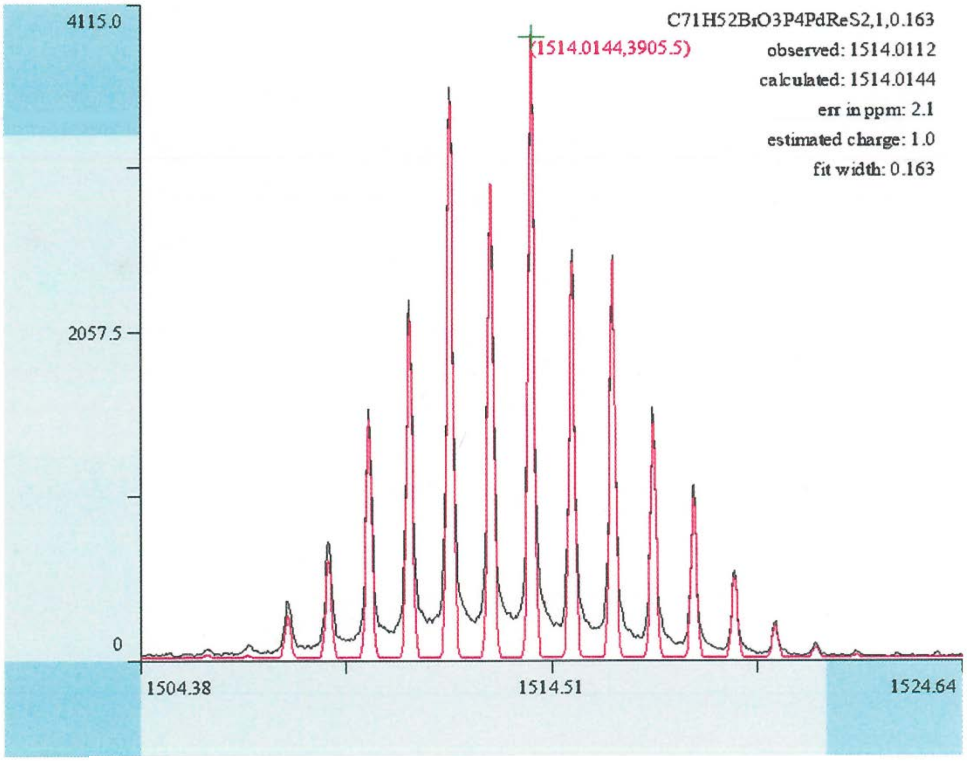

Figure S115. Mass spectrum (ESI+) of [(pdt)Pd(tpbz)ReBr(CO)$_3$], **14**.

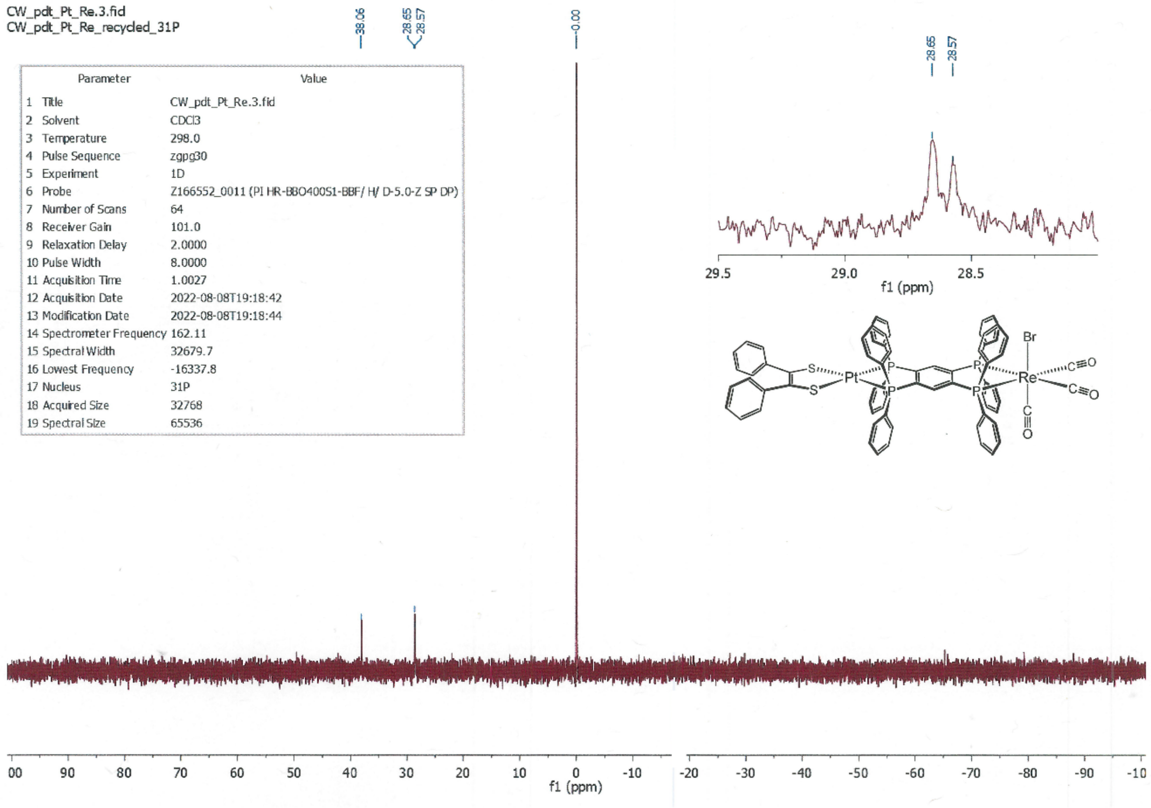

Figure S116. $^{31}P-\{^{1}H\}$ NMR spectrum ($CDCl_3$) of [(pdt)Pt(tpbz)ReBr(CO)$_3$], **15**.

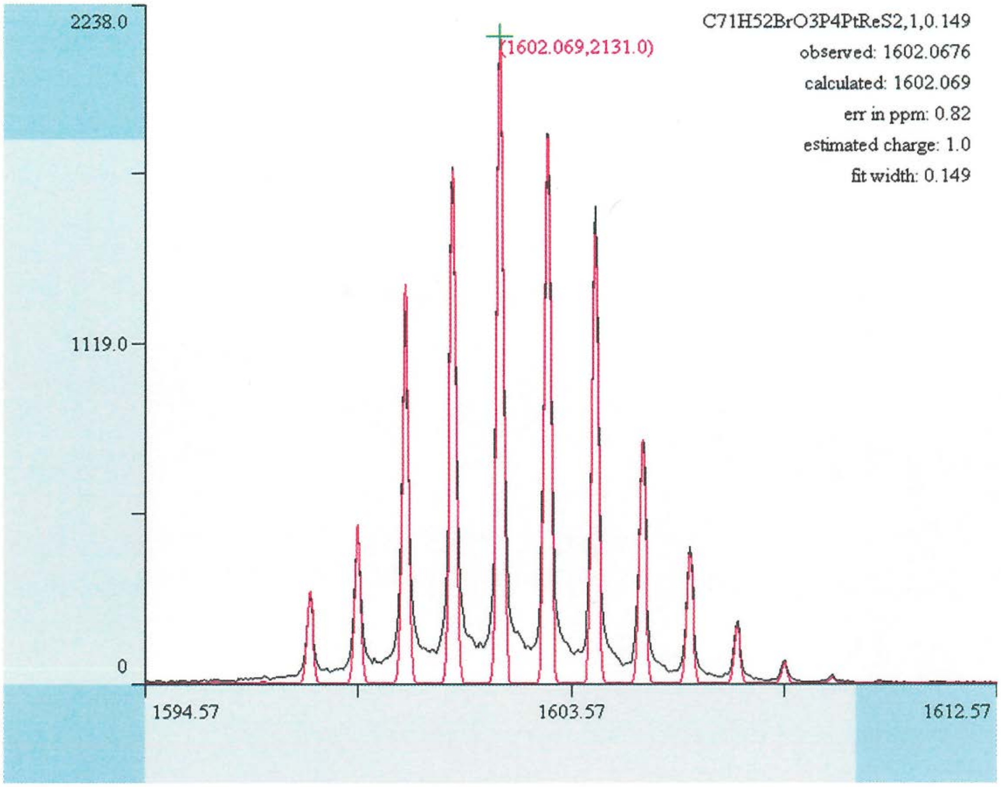

Figure S117. Mass spectrum (ESI+) of [(pdt)Pt(tpbz)ReBr(CO)$_3$], **15**.

Mikroanalytisches Laboratorium Kolbe (Nachf.)

Adress Höhenweg 17
45470 Mülheim an der Ruhr
www.mikro-lab.de

Tel.Nr. +49 - (0)208 - 32502
Fax.Nr. +49 - (0)208 - 382314
E-Mail info@mikro-lab.de

Analysis Request

Report to :

Name: James P. Donahue

Address: Dept. of Chemistry, Tulane University
6400 Freret St., New Orleans, LA 70118-5698, U.S.A.

E-Mail-Address: donahue@tulane.edu

Sample-Name.: JPD202

Elements to be determined : C, H

Other elements present : O, P, S, Br, Pd, Re

The sample is located under Argon [ ] Nitrogen [ ] Air [X] Vacuum [ ]

	Yes	No		Yes	No
**Handle under Argon**	[ ]	[X]	**Explosive**	[ ]	[X]
**CHN combustion charge** *	[X]	[ ]	**Sublimated**	[ ]	[X]
**Sensitive to moisture**	[ ]	[X]	**Volatile**	[ ]	[X]
**Hygroscopic**	[ ]	[X]	**Sample return requested**	[ ]	[X]

Drying instruction ______ **mbar** ______ °C

Molecular formula : $C_{71}H_{52}O_3P_4S_2BrRePt$

Expected results % :

C :	53.22%
H :	3.27%
O :	3.00%
P :	7.73%
S :	4.00%
Br :	4.99%
Re :	11.62%
Pt :	12.17%

Molecular structure

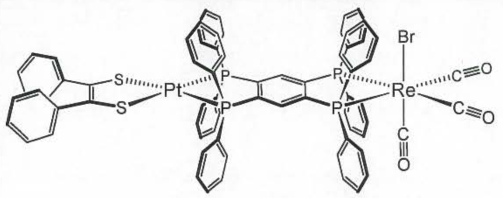

$C_{71}H_{52}O_3P_4S_2BrRePt$
1602.41 g/mol

Single : [ ] **Duplicate :** [ ] **Express :** [ ]

Duplicate determination only if the results differ from the expectation : [X]

Duplicate determination only if the results do not differ from the expectation : [ ]

Absolute deviation for a duplicate determination (standard 1%) [ ] %

Date : 12 August 2022 **Signet :** James P. Donahue

*) Combustion charge to the CHN analysis is required if the sample contains metals, silicon, fluorine or nitrogen-containing rings

Version 01/2016

Figure S118. Elemental analysis request form for [(pdt)Pt(tpbz)ReBr(CO)$_3$], **15**.

MIKROLAB
Mikroanalytisches Laboratorium Kolbe

Professor James P. Donahue
Department of Chemistry
Tulane University
6400 Freret St.
New Orleans, Louisiana 70118-5698, USA

Address : Osterfelder Str. 3
D-46047 Oberhausen
Phone : +49 - (0)208 - 32502
Fax : +49 - (0)208 - 382314
Email : info@mikro-lab.de
Website : www.mikro-lab.de

Date : 31.08.2022

Sample Name	% C	% H										V2O
JPD202	53,02	3,29										x
												x
												x

Kind regards

Patrick Springer

Figure S119. Elemental analysis results for [(pdt)Pt(tpbz)ReBr$(CO)_3$], **15**, from the Kolbe Microanalytical Laboratory in Oberhausen, Germany.

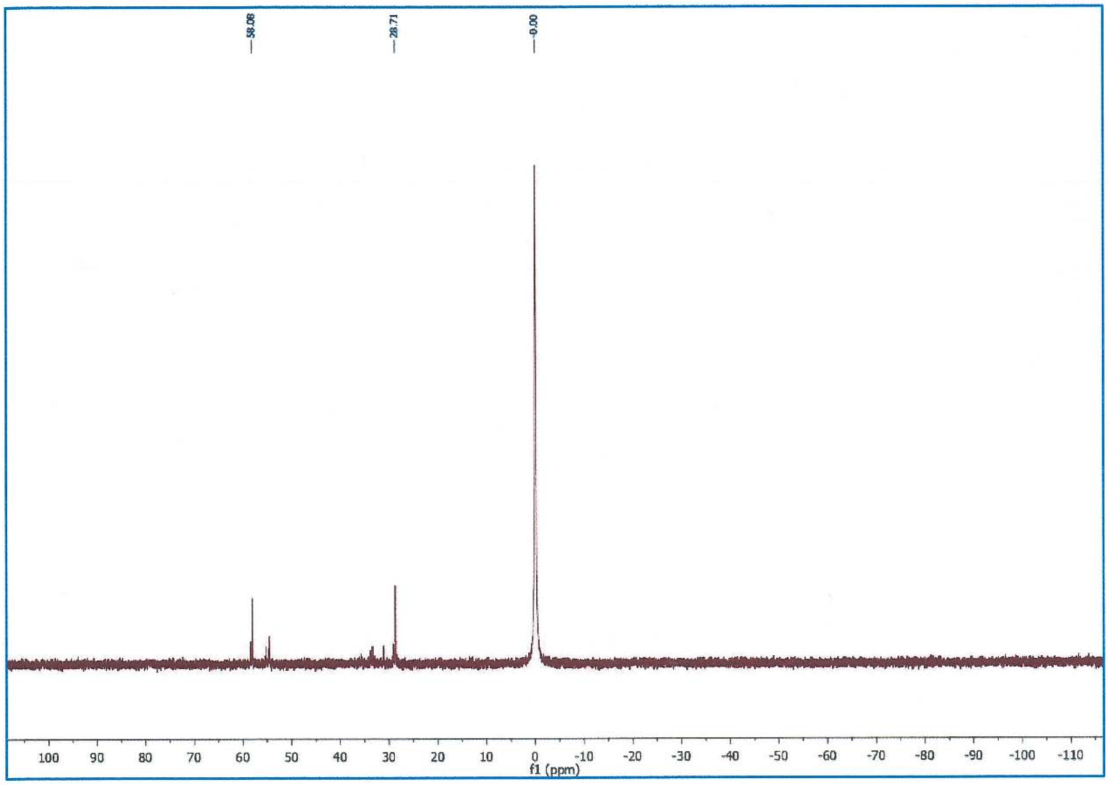

Figure S120. ${}^{31}P$-{${}^{1}H$} NMR spectrum (CD_2Cl_2) of [(mnt)Ni(tpbz)ReBr(CO)(tpbz)Ni(mnt)], **16**.

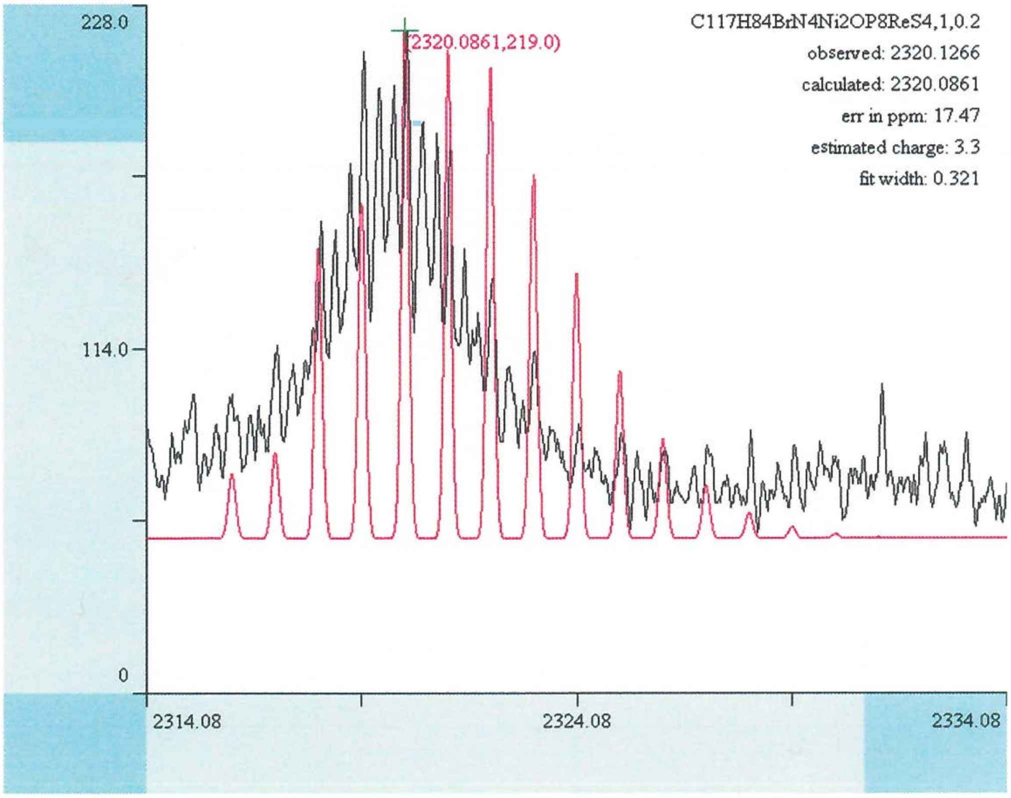

Figure S121. Mass spectrum (ESI+) of [(mnt)Ni(tpbz)ReBr(CO)(tpbz)Ni(mnt)], **16**.

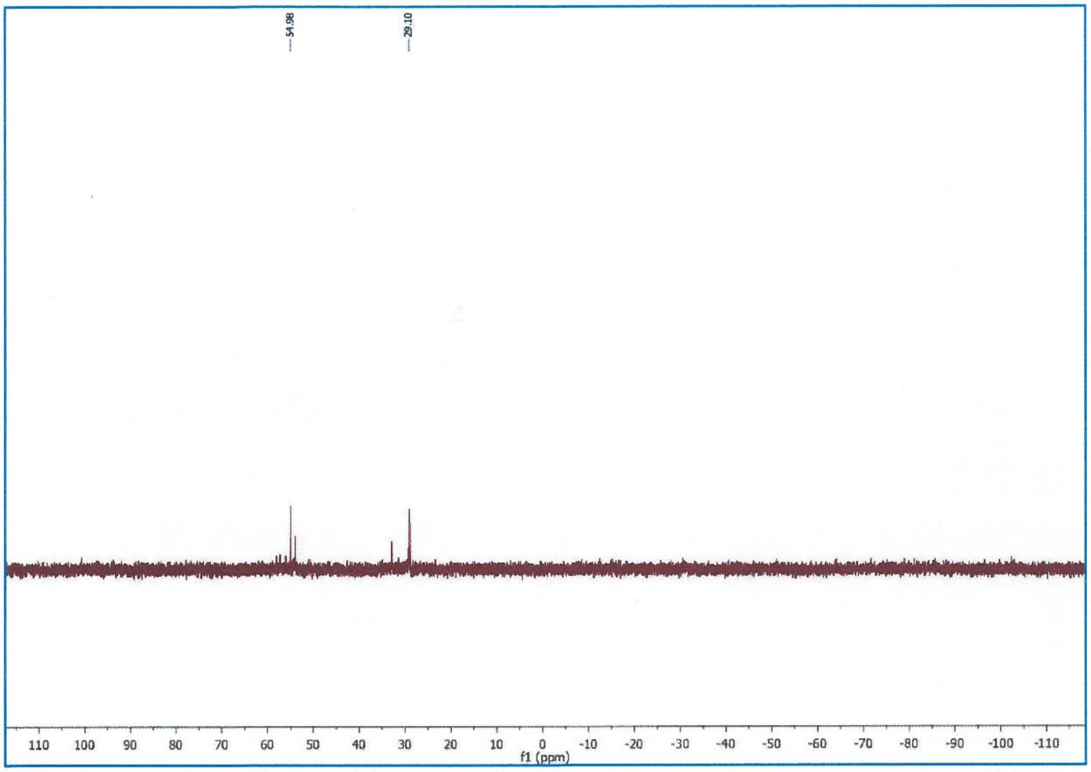

Figure S122. $^{31}P-\{^{1}H\}$ NMR spectrum (CD_2Cl_2) of [(mnt)Ni(tpbz)Re(CO)$_2$(tpbz)Ni(mnt)]Br, [**17**]Br.

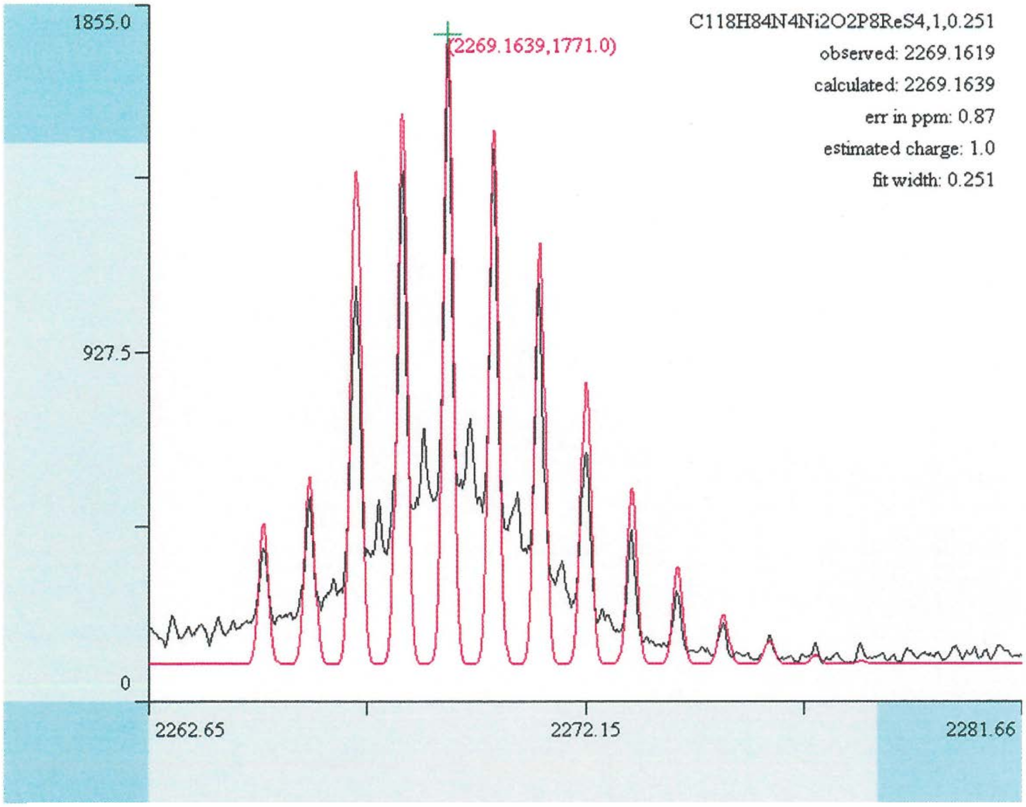

Figure S123. Mass spectrum (ESI+) of [(mnt)Ni(tpbz)Re(CO)$_2$(tpbz)Ni(mnt)]Br, [**17**]Br.

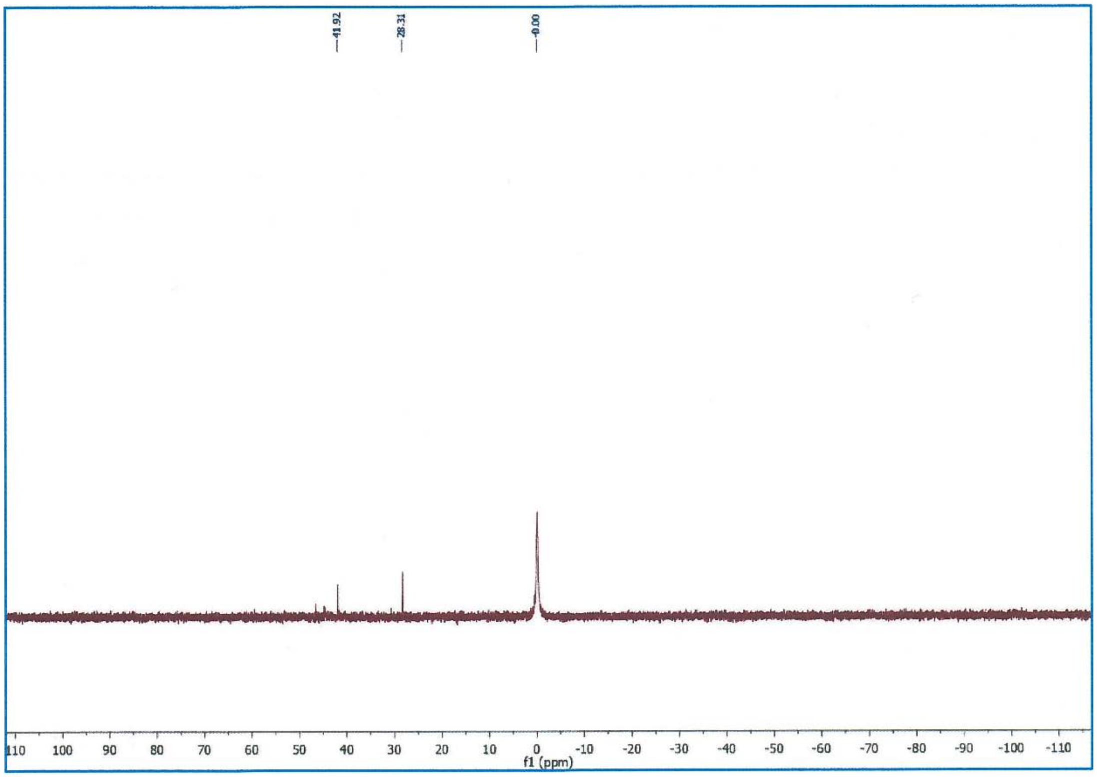

Figure S124. ^{31}P-{^{1}H} NMR spectrum ($CDCl_3$) of [(pdt)Pt(tpbz)Re(CO)Br(tpbz)Pt(pdt)], **18**.

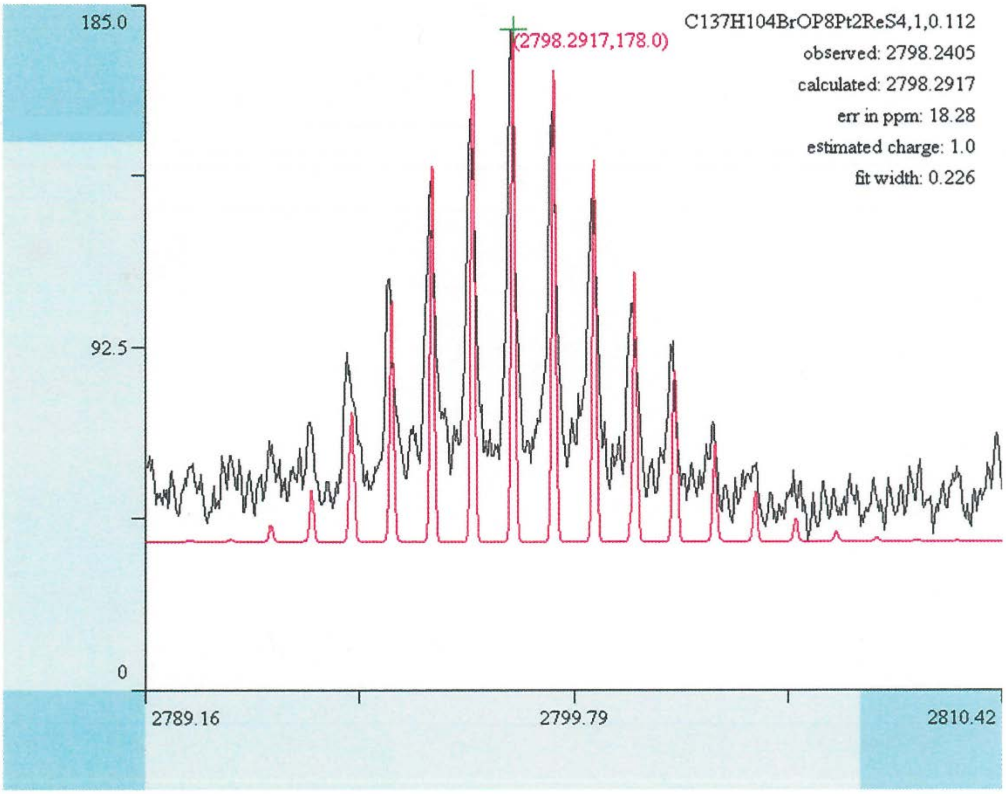

Figure S125. Mass spectrum (ESI+) of [(pdt)Pt(tpbz)Re(CO)Br(tpbz)Pt(pdt)], **18**.

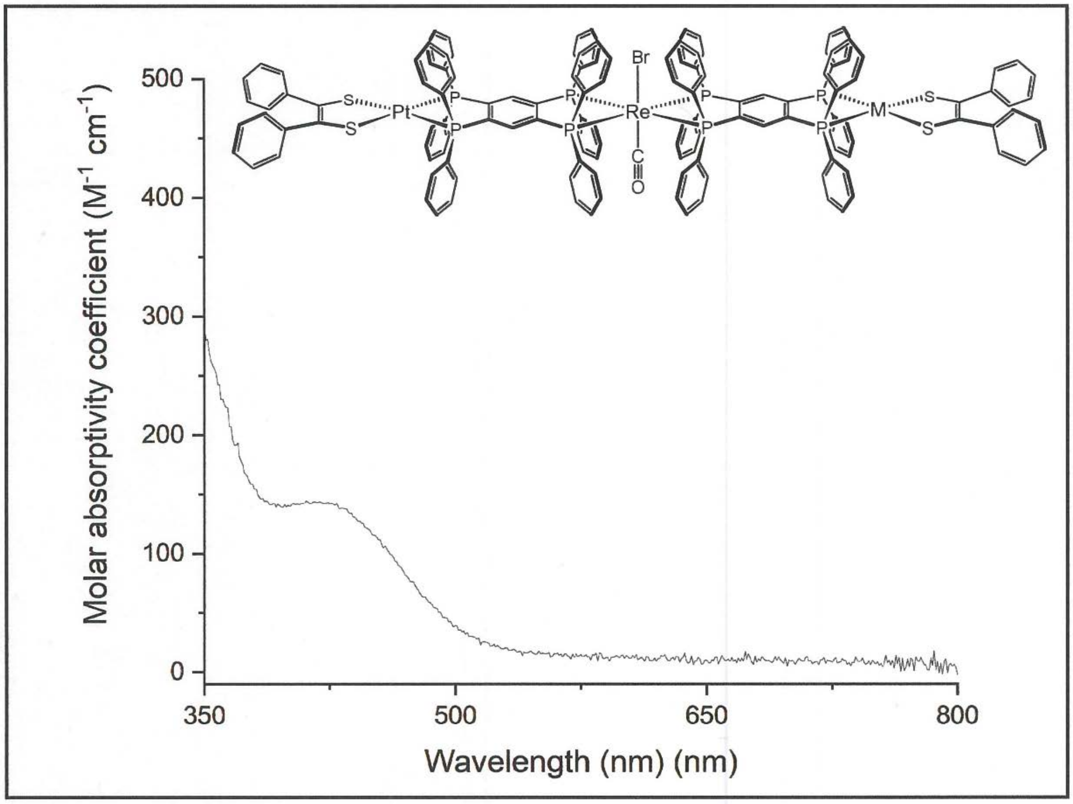

Figure S126. UV-vis spectrum (CH_2Cl_2) of [(pdt)Pt(tpbz)Re(CO)Br(tpbz)Pt(pdt)], **18**.

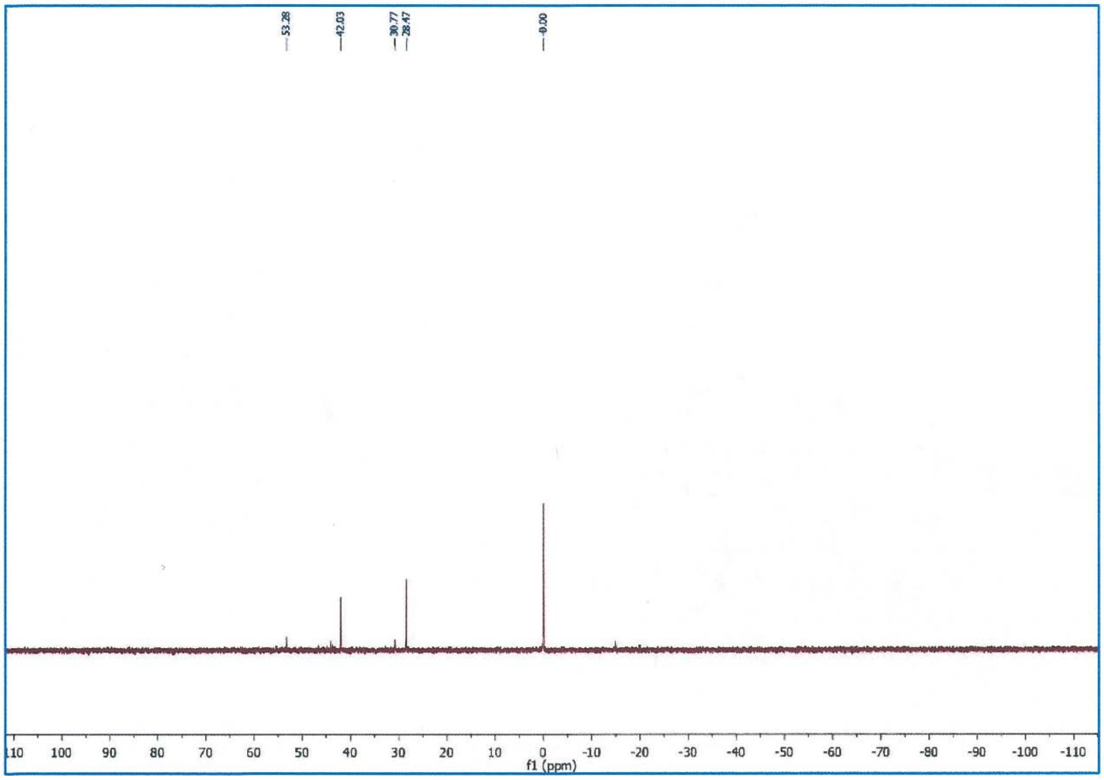

Figure S127. $^{31}P-\{^{1}H\}$ NMR spectrum ($CDCl_3$) of [(pdt)Pt(tpbz)Re$(CO)_2$(tpbz)Pt(pdt)]Br, [**19**]Br.

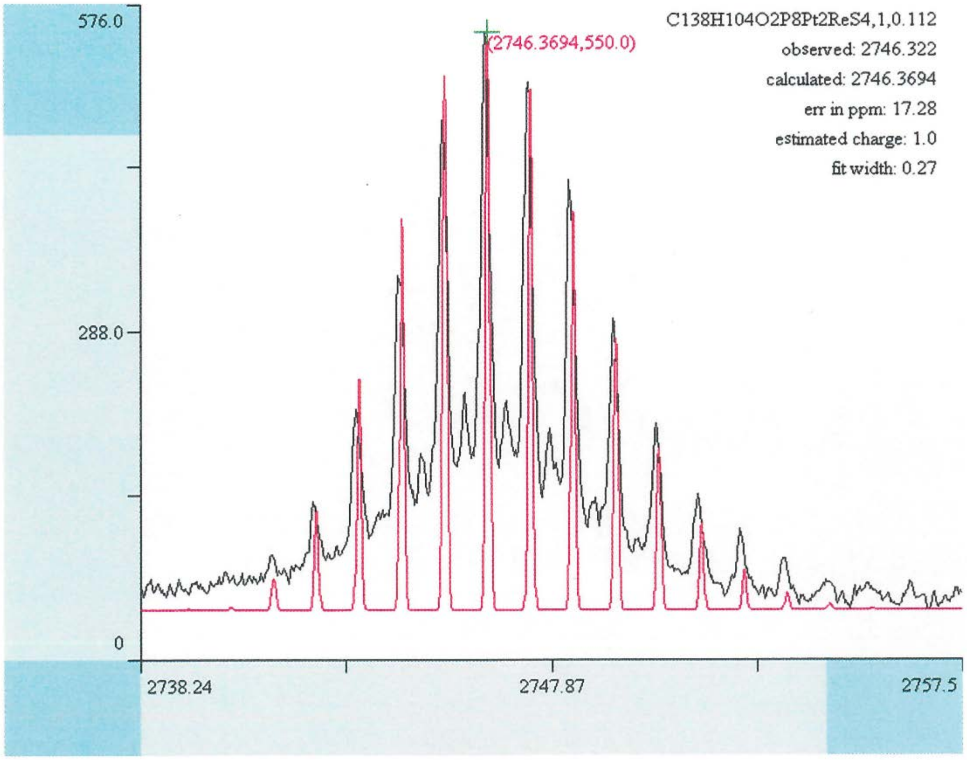

Figure S128. Mass spectrum (ESI+) of [(pdt)Pt(tpbz)Re$(CO)_2$(tpbz)Pt(pdt)]Br, [**19**]Br.

Table S5. D_{2h} complex [**1**]$^{2+}$
B3PW91/LANL2DZ-optimized
E = -5237.507053 au

Center Number	Atomic Number	Atomic Type	Coordinates (Angstroms) X	Y	Z
1	78	0	0.000000	0.000000	0.000000
2	28	0	9.140571	-0.000000	-0.000000
3	28	0	-9.140571	0.000000	-0.000000
4	16	0	-10.689056	1.594591	0.000000
5	16	0	10.689056	-1.594591	0.000000
6	16	0	-10.689056	-1.594591	-0.000000
7	16	0	10.689056	1.594591	0.000000
8	15	0	1.789457	-1.665104	0.000000
9	15	0	1.789457	1.665104	0.000000
10	15	0	-1.789457	-1.665104	-0.000000
11	15	0	-1.789457	1.665104	0.000000
12	15	0	-7.519393	-1.598331	0.000000
13	15	0	-7.519393	1.598331	0.000000
14	15	0	7.519393	-1.598331	0.000000
15	15	0	7.519393	1.598331	0.000000
16	7	0	14.397813	-2.165493	0.000000
17	7	0	14.397813	2.165493	0.000000
18	7	0	-14.397813	-2.165493	-0.000000
19	7	0	-14.397813	2.165493	0.000000
20	6	0	3.396871	-0.699375	0.000000
21	6	0	5.840245	-0.706989	0.000000
22	6	0	5.840245	0.706989	0.000000
23	6	0	3.396871	0.699375	0.000000
24	6	0	-3.396871	-0.699375	-0.000000
25	6	0	-5.840245	0.706989	0.000000
26	6	0	-3.396871	0.699375	0.000000
27	6	0	-5.840245	-0.706989	-0.000000
28	6	0	4.617947	-1.396865	0.000000
29	6	0	4.617947	1.396865	0.000000
30	6	0	-13.434805	-1.478347	0.000000
31	6	0	13.434805	1.478347	0.000000
32	6	0	13.434805	-1.478347	0.000000
33	6	0	-13.434805	1.478347	0.000000
34	6	0	12.253902	-0.683424	0.000000
35	6	0	12.253902	0.683424	0.000000
36	6	0	-12.253902	-0.683424	-0.000000
37	6	0	-12.253902	0.683424	0.000000
38	6	0	-4.617947	-1.396865	-0.000000
39	6	0	-4.617947	1.396865	0.000000
40	6	0	1.981029	2.692726	1.568566
41	6	0	-1.981029	2.692726	-1.568566
42	6	0	-1.981029	2.692726	1.568566
43	6	0	1.981029	2.692726	-1.568566
44	6	0	1.981029	-2.692726	-1.568566
45	6	0	1.981029	-2.692726	1.568566
46	6	0	-1.981029	-2.692726	-1.568566
47	6	0	-1.981029	-2.692726	1.568566
48	6	0	-7.480606	-2.653495	-1.552699
49	6	0	-7.480606	-2.653495	1.552699

50	6	0	-7.480606	2.653495	1.552699
51	6	0	-7.480606	2.653495	-1.552699
52	6	0	7.480606	2.653495	-1.552699
53	6	0	7.480606	2.653495	1.552699
54	6	0	7.480606	-2.653495	1.552699
55	6	0	7.480606	-2.653495	-1.552699
56	6	0	2.404831	4.031475	4.026102
57	6	0	-2.404831	-4.031475	4.026102
58	6	0	-2.404831	4.031475	4.026102
59	6	0	2.404831	-4.031475	4.026102
60	6	0	2.404831	4.031475	-4.026102
61	6	0	-2.404831	4.031475	-4.026102
62	6	0	-2.404831	-4.031475	-4.026102
63	6	0	2.404831	-4.031475	-4.026102
64	6	0	1.903821	4.724778	2.916110
65	6	0	-1.903821	-4.724778	2.916110
66	6	0	-1.903821	4.724778	2.916110
67	6	0	1.903821	-4.724778	2.916110
68	6	0	1.903821	4.724778	-2.916110
69	6	0	-1.903821	4.724778	-2.916110
70	6	0	-1.903821	-4.724778	-2.916110
71	6	0	1.903821	-4.724778	-2.916110
72	6	0	1.682459	4.061125	1.698325
73	6	0	-1.682459	-4.061125	1.698325
74	6	0	-1.682459	4.061125	1.698325
75	6	0	1.682459	-4.061125	1.698325
76	6	0	1.682459	4.061125	-1.698325
77	6	0	-1.682459	-4.061125	-1.698325
78	6	0	1.682459	-4.061125	-1.698325
79	6	0	-1.682459	4.061125	-1.698325
80	6	0	2.689805	2.661624	3.909653
81	6	0	-2.689805	-2.661624	3.909653
82	6	0	-2.689805	2.661624	3.909653
83	6	0	2.689805	-2.661624	3.909653
84	6	0	2.689805	2.661624	-3.909653
85	6	0	-2.689805	2.661624	-3.909653
86	6	0	-2.689805	-2.661624	-3.909653
87	6	0	2.689805	-2.661624	-3.909653
88	6	0	2.481976	2.002329	2.692383
89	6	0	-2.481976	-2.002329	2.692383
90	6	0	-2.481976	2.002329	2.692383
91	6	0	2.481976	-2.002329	2.692383
92	6	0	2.481976	2.002329	-2.692383
93	6	0	-2.481976	2.002329	-2.692383
94	6	0	-2.481976	-2.002329	-2.692383
95	6	0	2.481976	-2.002329	-2.692383
96	6	0	7.595347	4.112883	-3.964212
97	6	0	7.595347	-4.112883	-3.964212
98	6	0	7.595347	4.112883	3.964212
99	6	0	7.595347	-4.112883	3.964212
100	6	0	-7.595347	4.112883	-3.964212
101	6	0	-7.595347	4.112883	3.964212
102	6	0	-7.595347	-4.112883	-3.964212
103	6	0	-7.595347	-4.112883	3.964212
104	6	0	6.632730	3.760966	-1.754627
105	6	0	-6.632730	3.760966	-1.754627
106	6	0	6.632730	-3.760966	1.754627

107	6	0	-6.632730	3.760966	1.754627
108	6	0	-6.632730	-3.760966	-1.754627
109	6	0	6.632730	-3.760966	-1.754627
110	6	0	-6.632730	-3.760966	1.754627
111	6	0	6.632730	3.760966	1.754627
112	6	0	8.383845	2.282590	-2.569108
113	6	0	8.383845	-2.282590	-2.569108
114	6	0	-8.383845	2.282590	-2.569108
115	6	0	8.383845	2.282590	2.569108
116	6	0	-8.383845	-2.282590	2.569108
117	6	0	-8.383845	2.282590	2.569108
118	6	0	-8.383845	-2.282590	-2.569108
119	6	0	8.383845	-2.282590	2.569108
120	6	0	8.437913	3.008113	-3.769651
121	6	0	8.437913	-3.008113	-3.769651
122	6	0	8.437913	3.008113	3.769651
123	6	0	8.437913	-3.008113	3.769651
124	6	0	-8.437913	3.008113	-3.769651
125	6	0	-8.437913	3.008113	3.769651
126	6	0	-8.437913	-3.008113	-3.769651
127	6	0	-8.437913	-3.008113	3.769651
128	6	0	6.693628	4.486748	-2.953558
129	6	0	6.693628	4.486748	2.953558
130	6	0	6.693628	-4.486748	-2.953558
131	6	0	6.693628	-4.486748	2.953558
132	6	0	-6.693628	4.486748	-2.953558
133	6	0	-6.693628	4.486748	2.953558
134	6	0	-6.693628	-4.486748	-2.953558
135	6	0	-6.693628	-4.486748	2.953558
136	1	0	4.603223	2.481346	0.000000
137	1	0	-4.603223	-2.481346	0.000000
138	1	0	-4.603223	2.481346	-0.000000
139	1	0	4.603223	-2.481346	-0.000000
140	1	0	1.290124	4.641913	0.877190
141	1	0	-1.290124	-4.641913	0.877190
142	1	0	-1.290124	4.641913	0.877190
143	1	0	1.290124	-4.641913	0.877190
144	1	0	1.290124	4.641913	-0.877190
145	1	0	-1.290124	4.641913	-0.877190
146	1	0	-1.290124	-4.641913	-0.877190
147	1	0	1.290124	-4.641913	-0.877190
148	1	0	3.087973	2.112580	4.757452
149	1	0	-3.087973	-2.112580	4.757452
150	1	0	-3.087973	2.112580	4.757452
151	1	0	3.087973	-2.112580	4.757452
152	1	0	3.087973	2.112580	-4.757452
153	1	0	-3.087973	2.112580	-4.757452
154	1	0	-3.087973	-2.112580	-4.757452
155	1	0	3.087973	-2.112580	-4.757452
156	1	0	-1.684732	-5.786004	-2.990392
157	1	0	-1.684732	5.786004	-2.990392
158	1	0	1.684732	5.786004	2.990392
159	1	0	-1.684732	-5.786004	2.990392
160	1	0	-1.684732	5.786004	2.990392
161	1	0	1.684732	5.786004	-2.990392
162	1	0	1.684732	-5.786004	2.990392
163	1	0	1.684732	-5.786004	-2.990392

164	1	0	2.576413	-4.549288	4.964804
165	1	0	-2.576413	4.549288	-4.964804
166	1	0	2.576413	4.549288	4.964804
167	1	0	-2.576413	-4.549288	4.964804
168	1	0	-2.576413	4.549288	4.964804
169	1	0	2.576413	4.549288	-4.964804
170	1	0	2.576413	-4.549288	-4.964804
171	1	0	-2.576413	-4.549288	-4.964804
172	1	0	2.742035	0.953780	2.617152
173	1	0	-2.742035	-0.953780	2.617152
174	1	0	-2.742035	0.953780	2.617152
175	1	0	2.742035	-0.953780	2.617152
176	1	0	2.742035	0.953780	-2.617152
177	1	0	-2.742035	0.953780	-2.617152
178	1	0	-2.742035	-0.953780	-2.617152
179	1	0	2.742035	-0.953780	-2.617152
180	1	0	9.073655	1.459629	-2.405686
181	1	0	9.073655	1.459629	2.405686
182	1	0	-9.073655	1.459629	-2.405686
183	1	0	-9.073655	1.459629	2.405686
184	1	0	-9.073655	-1.459629	-2.405686
185	1	0	-9.073655	-1.459629	2.405686
186	1	0	9.073655	-1.459629	2.405686
187	1	0	9.073655	-1.459629	-2.405686
188	1	0	5.938133	4.092658	-0.990680
189	1	0	5.938133	4.092658	0.990680
190	1	0	5.938133	-4.092658	-0.990680
191	1	0	5.938133	-4.092658	0.990680
192	1	0	-5.938133	4.092658	0.990680
193	1	0	-5.938133	4.092658	-0.990680
194	1	0	-5.938133	-4.092658	-0.990680
195	1	0	-5.938133	-4.092658	0.990680
196	1	0	6.051533	5.352580	3.089835
197	1	0	6.051533	5.352580	-3.089835
198	1	0	6.051533	-5.352580	-3.089835
199	1	0	6.051533	-5.352580	3.089835
200	1	0	-6.051533	5.352580	-3.089835
201	1	0	-6.051533	5.352580	3.089835
202	1	0	-6.051533	-5.352580	-3.089835
203	1	0	-6.051533	-5.352580	3.089835
204	1	0	7.652801	4.687228	4.884499
205	1	0	7.652801	4.687228	-4.884499
206	1	0	7.652801	-4.687228	-4.884499
207	1	0	7.652801	-4.687228	4.884499
208	1	0	-7.652801	4.687228	-4.884499
209	1	0	-7.652801	4.687228	4.884499
210	1	0	-7.652801	-4.687228	-4.884499
211	1	0	-7.652801	-4.687228	4.884499
212	1	0	9.153051	2.723182	-4.535442
213	1	0	9.153051	2.723182	4.535442
214	1	0	9.153051	-2.723182	4.535442
215	1	0	9.153051	-2.723182	-4.535442
216	1	0	-9.153051	2.723182	-4.535442
217	1	0	-9.153051	2.723182	4.535442
218	1	0	-9.153051	-2.723182	-4.535442
219	1	0	-9.153051	-2.723182	4.535442

Table S6. D_2 complex [**1**]$^{2+}$
B3PW91/LANL2DZ-optimized
E = -5237.595433 au

Center Number	Atomic Number	Atomic Type	Coordinates (Angstroms) X	Y	Z
1	1	0	0.430728	-2.451353	4.639565
2	6	0	0.238256	-1.382553	4.631174
3	6	0	-0.238256	1.382553	4.631174
4	6	0	0.087394	-0.695773	3.414692
5	6	0	0.154201	-0.687708	5.848226
6	6	0	-0.154201	0.687708	5.848226
7	6	0	-0.087394	0.695773	3.414692
8	15	0	-0.056396	-1.628462	1.794617
9	15	0	0.452371	-1.517329	7.521197
10	15	0	-0.452371	1.517329	7.521197
11	15	0	0.056396	1.628462	1.794617
12	1	0	-0.430728	2.451353	4.639565
13	16	0	-0.075159	1.603816	-10.653708
14	6	0	-0.009538	0.684347	-12.212072
15	6	0	-1.253937	2.943601	1.912951
16	6	0	-0.000957	-1.474704	-13.396467
17	15	0	0.056396	-1.628462	-1.794617
18	6	0	0.009538	-0.684347	-12.212072
19	6	0	1.253937	2.943601	-1.912951
20	6	0	0.000957	1.474704	-13.396467
21	16	0	-0.075159	-1.603816	10.653708
22	6	0	-0.087394	-0.695773	-3.414692
23	6	0	0.154201	0.687708	-5.848226
24	6	0	-0.238256	-1.382553	-4.631174
25	6	0	0.087394	0.695773	-3.414692
26	6	0	0.238256	1.382553	-4.631174
27	6	0	-0.154201	-0.687708	-5.848226
28	1	0	-0.430728	-2.451353	-4.639565
29	15	0	-0.056396	1.628462	-1.794617
30	1	0	0.430728	2.451353	-4.639565
31	15	0	-0.452371	-1.517329	-7.521197
32	15	0	0.452371	1.517329	-7.521197
33	6	0	-1.727244	2.447022	-1.942038
34	78	0	0.000000	0.000000	0.000000
35	6	0	1.727244	2.447022	1.942038
36	16	0	0.075159	-1.603816	-10.653708
37	28	0	0.000000	-0.000000	9.121362
38	6	0	1.253937	-2.943601	1.912951
39	16	0	0.075159	1.603816	10.653708
40	6	0	-0.000957	1.474704	13.396467
41	6	0	-0.009538	-0.684347	12.212072
42	6	0	0.009538	0.684347	12.212072
43	6	0	0.000957	-1.474704	13.396467
44	28	0	-0.000000	-0.000000	-9.121362
45	6	0	-1.727244	-2.447022	1.942038
46	6	0	1.727244	-2.447022	-1.942038
47	6	0	-1.253937	-2.943601	-1.912951
48	6	0	0.445100	-3.148599	-7.415039
49	6	0	-2.278097	-1.922062	-7.486998

50	6	0	-0.445100	3.148599	-7.415039
51	6	0	2.278097	1.922062	-7.486998
52	6	0	0.445100	3.148599	7.415039
53	6	0	-2.278097	1.922062	7.486998
54	6	0	-0.445100	-3.148599	7.415039
55	6	0	2.278097	-1.922062	7.486998
56	7	0	0.012001	-2.160614	14.360332
57	7	0	-0.012001	2.160614	14.360332
58	7	0	-0.012001	-2.160614	-14.360332
59	7	0	0.012001	2.160614	-14.360332
60	6	0	-3.274633	4.884234	2.118053
61	6	0	-0.988534	4.274191	1.541952
62	6	0	-2.532190	2.582380	2.383877
63	6	0	-3.537169	3.553918	2.488657
64	6	0	-2.000781	5.241297	1.647110
65	1	0	0.000260	4.566073	1.201846
66	1	0	-2.743020	1.559736	2.687758
67	1	0	-4.515899	3.278202	2.869567
68	1	0	-1.791766	6.271445	1.373628
69	1	0	-4.052338	5.636910	2.206947
70	6	0	-3.274633	-4.884234	-2.118053
71	6	0	-2.532190	-2.582380	-2.383877
72	6	0	-0.988534	-4.274191	-1.541952
73	6	0	-2.000781	-5.241297	-1.647110
74	6	0	-3.537169	-3.553918	-2.488657
75	1	0	-2.743020	-1.559736	-2.687758
76	1	0	0.000260	-4.566073	-1.201846
77	1	0	-1.791766	-6.271445	-1.373628
78	1	0	-4.515899	-3.278202	-2.869567
79	1	0	-4.052338	-5.636910	-2.206947
80	6	0	-4.309809	3.502091	-2.280910
81	6	0	-1.952097	3.467402	-2.888462
82	6	0	-2.795258	1.957786	-1.169187
83	6	0	-4.084612	2.485079	-1.339562
84	6	0	-3.241741	3.992772	-3.051763
85	1	0	-1.144623	3.857038	-3.501383
86	1	0	-2.621488	1.174962	-0.434884
87	1	0	-4.907063	2.108177	-0.738703
88	1	0	-3.410795	4.775566	-3.784739
89	1	0	-5.307586	3.909657	-2.414314
90	6	0	-4.309809	-3.502091	2.280910
91	6	0	-2.795258	-1.957786	1.169187
92	6	0	-1.952097	-3.467402	2.888462
93	6	0	-3.241741	-3.992772	3.051763
94	6	0	-4.084612	-2.485079	1.339562
95	1	0	-2.621488	-1.174962	0.434884
96	1	0	-1.144623	-3.857038	3.501383
97	1	0	-3.410795	-4.775566	3.784739
98	1	0	-4.907063	-2.108177	0.738703
99	1	0	-5.307586	-3.909657	2.414314
100	6	0	4.309809	3.502091	2.280910
101	6	0	1.952097	3.467402	2.888462
102	6	0	2.795258	1.957786	1.169187
103	6	0	4.084612	2.485079	1.339562
104	6	0	3.241741	3.992772	3.051763
105	1	0	1.144623	3.857038	3.501383
106	1	0	2.621488	1.174962	0.434884

107	1	0	4.907063	2.108177	0.738703
108	1	0	3.410795	4.775566	3.784739
109	1	0	5.307586	3.909657	2.414314
110	6	0	3.274633	4.884234	-2.118053
111	6	0	0.988534	4.274191	-1.541952
112	6	0	2.532190	2.582380	-2.383877
113	6	0	3.537169	3.553918	-2.488657
114	6	0	2.000781	5.241297	-1.647110
115	1	0	-0.000260	4.566073	-1.201846
116	1	0	2.743020	1.559736	-2.687758
117	1	0	4.515899	3.278202	-2.869567
118	1	0	1.791766	6.271445	-1.373628
119	1	0	4.052338	5.636910	-2.206947
120	6	0	4.309809	-3.502091	-2.280910
121	6	0	2.795258	-1.957786	-1.169187
122	6	0	1.952097	-3.467402	-2.888462
123	6	0	3.241741	-3.992772	-3.051763
124	6	0	4.084612	-2.485079	-1.339562
125	1	0	2.621488	-1.174962	-0.434884
126	1	0	1.144623	-3.857038	-3.501383
127	1	0	3.410795	-4.775566	-3.784739
128	1	0	4.907063	-2.108177	-0.738703
129	1	0	5.307586	-3.909657	-2.414314
130	6	0	3.274633	-4.884234	2.118053
131	6	0	2.532190	-2.582380	2.383877
132	6	0	0.988534	-4.274191	1.541952
133	6	0	2.000781	-5.241297	1.647110
134	6	0	3.537169	-3.553918	2.488657
135	1	0	2.743020	-1.559736	2.687758
136	1	0	-0.000260	-4.566073	1.201846
137	1	0	1.791766	-6.271445	1.373628
138	1	0	4.515899	-3.278202	2.869567
139	1	0	4.052338	-5.636910	2.206947
140	6	0	1.866079	5.569836	7.322041
141	6	0	-0.146399	4.288015	6.834720
142	6	0	1.741947	3.223315	7.958419
143	6	0	2.451042	4.434045	7.906629
144	6	0	0.566766	5.496489	6.788938
145	1	0	-1.167956	4.249948	6.463464
146	1	0	2.176974	2.359965	8.454832
147	1	0	3.440712	4.495966	8.349297
148	1	0	0.100419	6.382950	6.367598
149	1	0	2.405635	6.512889	7.309932
150	6	0	-5.038864	2.441009	7.651670
151	6	0	-3.165717	1.307943	6.584411
152	6	0	-2.776362	2.785238	8.484213
153	6	0	-4.151843	3.048235	8.557490
154	6	0	-4.543743	1.568451	6.669471
155	1	0	-2.800939	0.614566	5.830917
156	1	0	-2.106140	3.226265	9.217356
157	1	0	-4.529277	3.708115	9.332796
158	1	0	-5.229034	1.078037	5.982903
159	1	0	-6.105408	2.634038	7.723617
160	6	0	5.038864	-2.441009	7.651670
161	6	0	2.776362	-2.785238	8.484213
162	6	0	3.165717	-1.307943	6.584411
163	6	0	4.543743	-1.568451	6.669471

164	6	0	4.151843	-3.048235	8.557490
165	1	0	2.106140	-3.226265	9.217356
166	1	0	2.800939	-0.614566	5.830917
167	1	0	5.229034	-1.078037	5.982903
168	1	0	4.529277	-3.708115	9.332796
169	1	0	6.105408	-2.634038	7.723617
170	6	0	-1.866079	-5.569836	7.322041
171	6	0	-1.741947	-3.223315	7.958419
172	6	0	0.146399	-4.288015	6.834720
173	6	0	-0.566766	-5.496489	6.788938
174	6	0	-2.451042	-4.434045	7.906629
175	1	0	-2.176974	-2.359965	8.454832
176	1	0	1.167956	-4.249948	6.463464
177	1	0	-0.100419	-6.382950	6.367598
178	1	0	-3.440712	-4.495966	8.349297
179	1	0	-2.405635	-6.512889	7.309932
180	6	0	5.038864	2.441009	-7.651670
181	6	0	3.165717	1.307943	-6.584411
182	6	0	2.776362	2.785238	-8.484213
183	6	0	4.151843	3.048235	-8.557490
184	6	0	4.543743	1.568451	-6.669471
185	1	0	2.800939	0.614566	-5.830917
186	1	0	2.106140	3.226265	-9.217356
187	1	0	4.529277	3.708115	-9.332796
188	1	0	5.229034	1.078037	-5.982903
189	1	0	6.105408	2.634038	-7.723617
190	6	0	-1.866079	5.569836	-7.322041
191	6	0	0.146399	4.288015	-6.834720
192	6	0	-1.741947	3.223315	-7.958419
193	6	0	-2.451042	4.434045	-7.906629
194	6	0	-0.566766	5.496489	-6.788938
195	1	0	1.167956	4.249948	-6.463464
196	1	0	-2.176974	2.359965	-8.454832
197	1	0	-3.440712	4.495966	-8.349297
198	1	0	-0.100419	6.382950	-6.367598
199	1	0	-2.405635	6.512889	-7.309932
200	6	0	1.866079	-5.569836	-7.322041
201	6	0	1.741947	-3.223315	-7.958419
202	6	0	-0.146399	-4.288015	-6.834720
203	6	0	0.566766	-5.496489	-6.788938
204	6	0	2.451042	-4.434045	-7.906629
205	1	0	2.176974	-2.359965	-8.454832
206	1	0	-1.167956	-4.249948	-6.463464
207	1	0	0.100419	-6.382950	-6.367598
208	1	0	3.440712	-4.495966	-8.349297
209	1	0	2.405635	-6.512889	-7.309932
210	6	0	-5.038864	-2.441009	-7.651670
211	6	0	-2.776362	-2.785238	-8.484213
212	6	0	-3.165717	-1.307943	-6.584411
213	6	0	-4.543743	-1.568451	-6.669471
214	6	0	-4.151843	-3.048235	-8.557490
215	1	0	-2.106140	-3.226265	-9.217356
216	1	0	-2.800939	-0.614566	-5.830917
217	1	0	-5.229034	-1.078037	-5.982903
218	1	0	-4.529277	-3.708115	-9.332796
219	1	0	-6.105408	-2.634038	-7.723617

Table S7. C_i complex [**1**]$^{2+}$
B3PW91/LANL2DZ-optimized
E = -5237.600267 au

Center Number	Atomic Number	Atomic Type	Coordinates (Angstroms) X	Y	Z
1	78	0	0.000000	0.000000	0.000000
2	28	0	6.278318	6.318381	-0.862862
3	28	0	-6.278318	-6.318381	0.862862
4	16	0	6.126510	8.481551	-0.405400
5	16	0	-6.126510	-8.481551	0.405400
6	16	0	8.407780	6.243436	-0.252946
7	16	0	-8.407780	-6.243436	0.252946
8	15	0	4.122073	6.296679	-1.584897
9	15	0	-4.122073	-6.296679	1.584897
10	15	0	6.337788	4.116851	-1.426829
11	15	0	-6.337788	-4.116851	1.426829
12	15	0	-0.059089	-2.345158	0.615056
13	15	0	0.059089	2.345158	-0.615056
14	15	0	2.389173	0.065258	-0.410525
15	15	0	-2.389173	-0.065258	0.410525
16	7	0	8.151066	11.429762	0.726767
17	7	0	-8.151066	-11.429762	-0.726767
18	7	0	11.237262	8.401335	0.934854
19	7	0	-11.237262	-8.401335	-0.934854
20	6	0	-8.013695	-10.287818	-0.449778
21	6	0	-7.807614	-8.920737	-0.109670
22	6	0	-8.782543	-7.964069	-0.175137
23	6	0	-10.119483	-8.221414	-0.591610
24	6	0	-3.544539	-4.517037	1.320124
25	6	0	-2.204816	-4.163596	1.116634
26	1	0	-1.446446	-4.941391	1.125620
27	6	0	-1.857226	-2.825562	0.844349
28	6	0	-2.854616	-1.846788	0.765591
29	6	0	-4.203970	-2.200026	0.963685
30	1	0	-4.991641	-1.459815	0.855676
31	6	0	-4.551883	-3.526896	1.245270
32	6	0	-2.802814	-7.343194	0.782015
33	6	0	-2.898496	-7.598413	-0.600261
34	1	0	-3.734831	-7.205229	-1.171845
35	6	0	-1.947379	-8.418690	-1.225893
36	1	0	-2.041708	-8.639729	-2.285139
37	6	0	-0.907680	-8.993419	-0.475101
38	1	0	-0.190573	-9.653035	-0.955833
39	6	0	-0.819227	-8.746965	0.905261
40	1	0	-0.035918	-9.217174	1.493262
41	6	0	-1.766002	-7.923862	1.537011
42	1	0	-1.717712	-7.772205	2.612116
43	6	0	-3.963058	-6.598478	3.420904
44	6	0	-4.703699	-7.672589	3.954045
45	1	0	-5.351920	-8.263648	3.311027
46	6	0	-4.603665	-7.976790	5.320428
47	1	0	-5.172616	-8.807419	5.727082
48	6	0	-3.780119	-7.205874	6.158117
49	1	0	-3.711226	-7.440124	7.216612

50	6	0	-3.052498	-6.127847	5.626788
51	1	0	-2.422817	-5.524588	6.275157
52	6	0	-3.139243	-5.823967	4.258469
53	1	0	-2.576358	-4.982605	3.861965
54	6	0	-6.767831	-3.832141	3.221369
55	6	0	-7.853183	-4.567562	3.738554
56	1	0	-8.379371	-5.282457	3.109979
57	6	0	-8.252938	-4.375110	5.070030
58	1	0	-9.092093	-4.940327	5.464183
59	6	0	-7.566273	-3.463766	5.889816
60	1	0	-7.874566	-3.322871	6.921871
61	6	0	-6.476765	-2.740800	5.375600
62	1	0	-5.939048	-2.042332	6.011059
63	6	0	-6.077495	-2.920312	4.041311
64	1	0	-5.228733	-2.359340	3.658012
65	6	0	-7.342420	-2.878193	0.459007
66	6	0	-7.987642	-1.799450	1.093267
67	1	0	-7.913682	-1.670770	2.169900
68	6	0	-8.774741	-0.914991	0.337436
69	1	0	-9.295119	-0.099433	0.831914
70	6	0	-8.921102	-1.107206	-1.046630
71	1	0	-9.552861	-0.437839	-1.623912
72	6	0	-8.282717	-2.189054	-1.676712
73	1	0	-8.426648	-2.363552	-2.739048
74	6	0	-7.498167	-3.078452	-0.926847
75	1	0	-7.055957	-3.947475	-1.406382
76	6	0	0.548469	-3.571115	-0.650613
77	6	0	-0.287128	-3.885990	-1.740561
78	1	0	-1.279807	-3.450715	-1.821208
79	6	0	0.145678	-4.794897	-2.715785
80	1	0	-0.508903	-5.048997	-3.544230
81	6	0	1.414281	-5.390216	-2.612188
82	1	0	1.744350	-6.102212	-3.362694
83	6	0	2.247077	-5.075203	-1.526885
84	1	0	3.222883	-5.543247	-1.436250
85	6	0	1.818861	-4.165774	-0.547612
86	1	0	2.461944	-3.950988	0.299513
87	6	0	0.736754	-2.748874	2.251838
88	6	0	0.974265	-4.084184	2.636741
89	1	0	0.780234	-4.908409	1.956323
90	6	0	1.474933	-4.359783	3.917599
91	1	0	1.658692	-5.388938	4.211622
92	6	0	1.732141	-3.312170	4.818470
93	1	0	2.115331	-3.531136	5.810807
94	6	0	1.495043	-1.983040	4.432725
95	1	0	1.697748	-1.171139	5.124670
96	6	0	1.000154	-1.700333	3.151008
97	1	0	0.827706	-0.669604	2.852620
98	6	0	-3.501437	0.382783	-1.016938
99	6	0	-3.702301	-0.565570	-2.039452
100	1	0	-3.248417	-1.551161	-1.977334
101	6	0	-4.522095	-0.254434	-3.132989
102	1	0	-4.688777	-0.994944	-3.909696
103	6	0	-5.142174	1.003858	-3.214623
104	1	0	-5.785898	1.239875	-4.056682
105	6	0	-4.940858	1.948960	-2.196304
106	1	0	-5.428737	2.917794	-2.249262

107	6	0	-4.120254	1.643103	-1.099743
108	1	0	-3.993176	2.373688	-0.307703
109	6	0	-2.961466	0.871749	1.916689
110	6	0	-4.330363	1.119983	2.144786
111	1	0	-5.081319	0.849881	1.407847
112	6	0	-4.735840	1.731170	3.340351
113	1	0	-5.790611	1.922827	3.513806
114	6	0	-3.785046	2.088449	4.311664
115	1	0	-4.104714	2.557098	5.237780
116	6	0	-2.422159	1.840518	4.081871
117	1	0	-1.684761	2.120021	4.828318
118	6	0	-2.009514	1.235088	2.885774
119	1	0	-0.953027	1.053328	2.707015
120	6	0	8.013695	10.287818	0.449778
121	6	0	7.807614	8.920737	0.109670
122	6	0	8.782543	7.964069	0.175137
123	6	0	10.119483	8.221414	0.591610
124	6	0	3.544539	4.517037	-1.320124
125	6	0	2.204816	4.163596	-1.116634
126	1	0	1.446446	4.941391	-1.125620
127	6	0	1.857226	2.825562	-0.844349
128	6	0	2.854616	1.846788	-0.765591
129	6	0	4.203970	2.200026	-0.963685
130	1	0	4.991641	1.459815	-0.855676
131	6	0	4.551883	3.526896	-1.245270
132	6	0	2.802814	7.343194	-0.782015
133	6	0	2.898496	7.598413	0.600261
134	1	0	3.734831	7.205229	1.171845
135	6	0	1.947379	8.418690	1.225893
136	1	0	2.041708	8.639729	2.285139
137	6	0	0.907680	8.993419	0.475101
138	1	0	0.190573	9.653035	0.955833
139	6	0	0.819227	8.746965	-0.905261
140	1	0	0.035918	9.217174	-1.493262
141	6	0	1.766002	7.923862	-1.537011
142	1	0	1.717712	7.772205	-2.612116
143	6	0	3.963058	6.598478	-3.420904
144	6	0	4.703699	7.672589	-3.954045
145	1	0	5.351920	8.263648	-3.311027
146	6	0	4.603665	7.976790	-5.320428
147	1	0	5.172616	8.807419	-5.727082
148	6	0	3.780119	7.205874	-6.158117
149	1	0	3.711226	7.440124	-7.216612
150	6	0	3.052498	6.127847	-5.626788
151	1	0	2.422817	5.524588	-6.275157
152	6	0	3.139243	5.823967	-4.258469
153	1	0	2.576358	4.982605	-3.861965
154	6	0	6.767831	3.832141	-3.221369
155	6	0	7.853183	4.567562	-3.738554
156	1	0	8.379371	5.282457	-3.109979
157	6	0	8.252938	4.375110	-5.070030
158	1	0	9.092093	4.940327	-5.464183
159	6	0	7.566273	3.463766	-5.889816
160	1	0	7.874566	3.322871	-6.921871
161	6	0	6.476765	2.740800	-5.375600
162	1	0	5.939048	2.042332	-6.011059
163	6	0	6.077495	2.920312	-4.041311

164	1	0	5.228733	2.359340	-3.658012
165	6	0	7.342420	2.878193	-0.459007
166	6	0	7.987642	1.799450	-1.093267
167	1	0	7.913682	1.670770	-2.169900
168	6	0	8.774741	0.914991	-0.337436
169	1	0	9.295119	0.099433	-0.831914
170	6	0	8.921102	1.107206	1.046630
171	1	0	9.552861	0.437839	1.623912
172	6	0	8.282717	2.189054	1.676712
173	1	0	8.426648	2.363552	2.739048
174	6	0	7.498167	3.078452	0.926847
175	1	0	7.055957	3.947475	1.406382
176	6	0	-0.548469	3.571115	0.650613
177	6	0	0.287128	3.885990	1.740561
178	1	0	1.279807	3.450715	1.821208
179	6	0	-0.145678	4.794897	2.715785
180	1	0	0.508903	5.048997	3.544230
181	6	0	-1.414281	5.390216	2.612188
182	1	0	-1.744350	6.102212	3.362694
183	6	0	-2.247077	5.075203	1.526885
184	1	0	-3.222883	5.543247	1.436250
185	6	0	-1.818861	4.165774	0.547612
186	1	0	-2.461944	3.950988	-0.299513
187	6	0	-0.736754	2.748874	-2.251838
188	6	0	-0.974265	4.084184	-2.636741
189	1	0	-0.780234	4.908409	-1.956323
190	6	0	-1.474933	4.359783	-3.917599
191	1	0	-1.658692	5.388938	-4.211622
192	6	0	-1.732141	3.312170	-4.818470
193	1	0	-2.115331	3.531136	-5.810807
194	6	0	-1.495043	1.983040	-4.432725
195	1	0	-1.697748	1.171139	-5.124670
196	6	0	-1.000154	1.700333	-3.151008
197	1	0	-0.827706	0.669604	-2.852620
198	6	0	3.501437	-0.382783	1.016938
199	6	0	3.702301	0.565570	2.039452
200	1	0	3.248417	1.551161	1.977334
201	6	0	4.522095	0.254434	3.132989
202	1	0	4.688777	0.994944	3.909696
203	6	0	5.142174	-1.003858	3.214623
204	1	0	5.785898	-1.239875	4.056682
205	6	0	4.940858	-1.948960	2.196304
206	1	0	5.428737	-2.917794	2.249262
207	6	0	4.120254	-1.643103	1.099743
208	1	0	3.993176	-2.373688	0.307703
209	6	0	2.961466	-0.871749	-1.916689
210	6	0	4.330363	-1.119983	-2.144786
211	1	0	5.081319	-0.849881	-1.407847
212	6	0	4.735840	-1.731170	-3.340351
213	1	0	5.790611	-1.922827	-3.513806
214	6	0	3.785046	-2.088449	-4.311664
215	1	0	4.104714	-2.557098	-5.237780
216	6	0	2.422159	-1.840518	-4.081871
217	1	0	1.684761	-2.120021	-4.828318
218	6	0	2.009514	-1.235088	-2.885774
219	1	0	0.953027	-1.053328	-2.707015

Table S8. C_1 complex [**1**]$^{2+}$
B3PW91/LANL2DZ-optimized
E = -5237.602645 au

Center Number	Atomic Number	Atomic Type	Coordinates (Angstroms) X	Y	Z
1	78	0	-0.000019	-0.056911	0.000030
2	28	0	-8.958103	0.079909	-0.447998
3	28	0	8.958124	0.079913	0.448033
4	16	0	10.345395	-1.478527	1.194757
5	16	0	-10.345383	-1.478528	-1.194739
6	16	0	10.301546	1.719055	1.097436
7	16	0	-10.301546	1.719050	-1.097382
8	15	0	-1.779798	1.573066	-0.161702
9	15	0	1.779815	1.573073	0.161739
10	15	0	-1.802304	-1.678289	0.000516
11	15	0	1.802313	-1.678297	-0.000556
12	15	0	-7.491997	1.590221	0.411851
13	15	0	7.492004	1.590225	-0.411823
14	15	0	7.542271	-1.523553	-0.321425
15	15	0	-7.542268	-1.523559	0.321437
16	7	0	13.661175	2.377551	2.630989
17	7	0	-13.661177	2.377554	-2.630927
18	7	0	13.720849	-1.949026	2.762752
19	7	0	-13.720827	-1.949042	-2.762705
20	6	0	1.946825	2.213669	1.906173
21	6	0	-1.946773	2.213678	-1.906135
22	6	0	-1.856315	3.014878	1.013004
23	6	0	1.856253	3.014879	-1.012979
24	6	0	-1.859237	-2.751039	1.525220
25	6	0	1.859252	-2.750943	-1.525333
26	6	0	2.009997	-2.761531	1.499172
27	6	0	-2.009999	-2.761457	-1.499260
28	6	0	-3.415642	-0.725349	0.108306
29	6	0	3.415646	-0.725339	-0.108302
30	6	0	-3.395145	0.674240	0.142927
31	6	0	3.395148	0.674252	-0.142915
32	6	0	-5.841455	-0.701263	0.320900
33	6	0	5.841460	-0.701255	-0.320888
34	6	0	-4.597678	1.392361	0.289629
35	6	0	4.597685	1.392370	-0.289604
36	6	0	-4.640705	-1.413345	0.199558
37	6	0	4.640708	-1.413335	-0.199551
38	6	0	-5.818941	0.711522	0.373952
39	6	0	5.818946	0.711530	-0.373932
40	6	0	7.284155	-3.130407	0.589285
41	6	0	-7.284165	-3.130417	-0.589263
42	6	0	-7.866202	-1.985648	2.101431
43	6	0	7.866217	-1.985631	-2.101411
44	6	0	7.168547	3.225050	0.429206
45	6	0	-7.168551	3.225039	-0.429192
46	6	0	-7.810903	1.992783	2.206912
47	6	0	7.810930	1.992780	-2.206883
48	6	0	-2.025507	5.143423	2.839644
49	6	0	2.025226	5.143401	-2.839666

50	6	0	-2.292186	3.829680	3.264103
51	6	0	2.292005	3.829670	-3.264104
52	6	0	-8.433111	2.660148	4.864618
53	6	0	8.433157	2.660130	-4.864591
54	6	0	-7.158472	-2.542139	4.361230
55	6	0	7.158505	-2.542103	-4.361226
56	6	0	7.187945	-4.313919	2.712901
57	6	0	-7.187942	-4.313925	-2.712884
58	6	0	-1.392283	-2.980064	3.902714
59	6	0	1.392394	-2.979777	-3.902867
60	6	0	-2.124221	-4.178881	3.931202
61	6	0	2.124304	-4.178610	-3.931420
62	6	0	2.338185	-4.337177	3.802897
63	6	0	-2.338265	-4.337025	-3.803025
64	6	0	-1.673951	5.388439	1.502929
65	6	0	1.673670	5.388411	-1.502954
66	6	0	2.282481	3.003749	4.583196
67	6	0	-2.282301	3.003821	-4.583154
68	6	0	-8.499066	-2.738911	4.733445
69	6	0	8.499101	-2.738875	-4.733433
70	6	0	1.321641	2.035007	4.252346
71	6	0	-1.321483	2.035064	-4.252285
72	6	0	6.993491	-5.525862	2.028325
73	6	0	-6.993508	-5.525873	-2.028309
74	6	0	-7.106516	2.369982	4.504424
75	6	0	7.106559	2.369953	-4.504405
76	6	0	-9.449272	2.611409	3.895558
77	6	0	9.449309	2.611410	-3.895521
78	6	0	2.850192	-3.029265	3.766597
79	6	0	-2.850127	-3.029057	-3.766726
80	6	0	-9.144272	2.271499	2.568547
81	6	0	9.144300	2.271506	-2.568508
82	6	0	-6.792219	2.039700	3.176248
83	6	0	6.792257	2.039679	-3.176231
84	6	0	-2.208771	2.766533	2.355255
85	6	0	2.208696	2.766533	-2.355237
86	6	0	3.075732	3.576680	3.574280
87	6	0	-3.075582	3.576746	-3.574258
88	6	0	6.779032	5.669354	1.760323
89	6	0	-6.779050	5.669338	-1.760326
90	6	0	7.266450	3.284860	1.833498
91	6	0	-7.266489	3.284846	-1.833484
92	6	0	1.152479	1.641414	2.916156
93	6	0	-1.152381	1.641442	-2.916095
94	6	0	7.067286	4.506306	2.495623
95	6	0	-7.067332	4.506291	-2.495617
96	6	0	6.951237	-5.540041	0.624089
97	6	0	-6.951266	-5.540054	-0.624073
98	6	0	7.339791	-3.116813	1.997103
99	6	0	-7.339784	-3.116821	-1.997084
100	6	0	2.688074	-2.239873	2.618804
101	6	0	-2.687976	-2.239704	-2.618909
102	6	0	1.653668	-4.851880	2.689757
103	6	0	-1.653859	-4.851826	-2.689862
104	6	0	-1.256671	-2.268762	2.701878
105	6	0	1.256748	-2.268566	-2.701979
106	6	0	-2.720585	-4.664016	2.755119

107	6	0	2.720612	-4.663848	-2.755347
108	6	0	-6.838913	-2.168972	3.045786
109	6	0	6.838939	-2.168948	-3.045781
110	6	0	-9.523179	-2.554570	3.789555
111	6	0	9.523207	-2.554543	-3.789532
112	6	0	2.915320	3.183052	2.238025
113	6	0	-2.915230	3.183091	-2.238004
114	6	0	-1.583585	4.327071	0.587198
115	6	0	1.583410	4.327052	-0.587199
116	6	0	9.212116	-2.171832	-2.475479
117	6	0	-9.212097	-2.171850	2.475500
118	6	0	6.887134	4.390137	-0.309374
119	6	0	-6.887113	4.390127	0.309376
120	6	0	6.689996	5.609887	0.359050
121	6	0	-6.689982	5.609874	-0.359054
122	6	0	7.097383	-4.344341	-0.098507
123	6	0	-7.097407	-4.344356	0.098524
124	6	0	1.481735	-4.065303	1.541281
125	6	0	-1.481882	-4.065287	-1.541366
126	6	0	-2.596584	-3.951528	1.552154
127	6	0	2.596581	-3.951444	-1.552335
128	6	0	11.741541	0.842819	1.763031
129	6	0	-11.741532	0.842820	-1.762995
130	6	0	12.840831	-1.286543	2.331175
131	6	0	-12.840818	-1.286536	-2.331161
132	6	0	11.760024	-0.523873	1.804581
133	6	0	-11.760011	-0.523872	-1.804552
134	6	0	12.800361	1.665680	2.241295
135	6	0	-12.800354	1.665678	-2.241260
136	1	0	-2.569185	3.637759	4.296463
137	1	0	2.568993	3.637758	-4.296469
138	1	0	-2.098869	5.967509	3.543057
139	1	0	2.098501	5.967478	-3.543099
140	1	0	-8.674570	2.916680	5.892170
141	1	0	8.674618	2.916654	-5.892144
142	1	0	0.708149	1.590091	5.030496
143	1	0	-0.707955	1.590165	-5.030416
144	1	0	2.415123	3.309007	5.616962
145	1	0	-2.414891	3.309110	-5.616917
146	1	0	7.259617	-4.305271	3.796600
147	1	0	-7.259602	-4.305272	-3.796584
148	1	0	6.906242	-6.455570	2.583663
149	1	0	-6.906263	-6.455580	-2.583647
150	1	0	3.386798	-2.629511	4.621880
151	1	0	-3.386654	-2.629230	-4.622024
152	1	0	-2.228658	-4.731135	4.860514
153	1	0	2.228767	-4.730792	-4.860771
154	1	0	2.478425	-4.951808	4.687146
155	1	0	-2.478539	-4.951628	-4.687288
156	1	0	-0.923826	-2.606411	4.808236
157	1	0	0.923986	-2.606032	-4.808373
158	1	0	-6.365983	-2.678242	5.092221
159	1	0	6.366020	-2.678195	-5.092221
160	1	0	10.477076	2.829328	-4.169696
161	1	0	-10.477041	2.829320	4.169751
162	1	0	-8.744500	-3.029090	5.751093
163	1	0	8.744543	-3.029046	-5.751081

164	1	0	7.180670	4.556364	3.574829
165	1	0	-7.180742	4.556347	-3.574815
166	1	0	6.658627	6.620441	2.271845
167	1	0	-6.658650	6.620424	-2.271850
168	1	0	7.548081	-2.191471	2.527332
169	1	0	-7.548056	-2.191476	-2.527315
170	1	0	-1.480307	6.403651	1.168975
171	1	0	1.479933	6.403614	-1.169009
172	1	0	-6.322017	2.398881	5.255934
173	1	0	6.322065	2.398834	-5.255921
174	1	0	-2.433529	1.756242	2.689553
175	1	0	2.433519	1.756251	-2.689518
176	1	0	-9.935619	2.221706	1.824011
177	1	0	9.935640	2.221725	-1.823964
178	1	0	-5.797952	-2.015808	2.771807
179	1	0	5.797974	-2.015786	-2.771810
180	1	0	-0.678653	-1.348130	2.681155
181	1	0	0.678754	-1.347921	-2.681212
182	1	0	-3.288414	-5.589563	2.773638
183	1	0	3.288421	-5.589403	-2.773908
184	1	0	3.107674	-1.237424	2.598234
185	1	0	-3.107471	-1.237212	-2.598337
186	1	0	1.257357	-5.862547	2.712953
187	1	0	-1.257667	-5.862540	-2.713056
188	1	0	6.835382	-6.480409	0.092383
189	1	0	-6.835422	-6.480424	-0.092367
190	1	0	7.544772	2.400688	2.400184
191	1	0	-7.544833	2.400673	-2.400156
192	1	0	0.406654	0.892651	2.659499
193	1	0	-0.406563	0.892679	-2.659421
194	1	0	3.823970	4.322300	3.823876
195	1	0	-3.823793	4.322388	-3.823868
196	1	0	-10.560997	-2.701217	4.073114
197	1	0	10.561027	-2.701190	-4.073085
198	1	0	6.498377	6.512894	-0.213661
199	1	0	-6.498343	6.512879	0.213654
200	1	0	-5.763252	1.810803	2.909888
201	1	0	5.763289	1.810775	-2.909875
202	1	0	-4.672145	-2.497809	0.139901
203	1	0	4.672149	-2.497799	-0.139897
204	1	0	-4.591712	2.478350	0.318326
205	1	0	4.591722	2.478360	-0.318282
206	1	0	10.007652	-2.019912	-1.749518
207	1	0	-10.007641	-2.019921	1.749550
208	1	0	3.548143	3.634071	1.479502
209	1	0	-3.548065	3.634119	-1.479497
210	1	0	6.857869	4.359909	-1.395306
211	1	0	-6.857822	4.359901	1.395307
212	1	0	7.104079	-4.367592	-1.184969
213	1	0	-7.104106	-4.367607	1.184986
214	1	0	0.951485	-4.478858	0.689198
215	1	0	-0.951727	-4.478919	-0.689263
216	1	0	-3.075026	-4.336261	0.656015
217	1	0	3.074985	-4.336253	-0.656209
218	1	0	-1.317876	4.531101	-0.445185
219	1	0	1.317691	4.531087	0.445179

Table S9. C_2 complex [**1**]$^{2+}$
B3PW91/LANL2DZ-optimized
E = -5237.602268 au

Center Number	Atomic Number	Atomic Type	Coordinates (Angstroms) X	Y	Z
1	78	0	0.000000	0.000000	0.079936
2	28	0	-2.561944	-8.564580	-0.679161
3	28	0	2.561944	8.564580	-0.679161
4	16	0	1.457967	10.274221	-1.556828
5	16	0	-1.457967	-10.274221	-1.556828
6	16	0	4.521972	9.389376	-1.305338
7	16	0	-4.521972	-9.389376	-1.305338
8	15	0	2.061129	1.272000	0.047600
9	15	0	-2.061129	-1.272000	0.047600
10	15	0	-1.070758	2.174316	0.056468
11	15	0	1.070758	-2.174316	0.056468
12	15	0	0.612682	7.672647	0.078042
13	15	0	-0.612682	-7.672647	0.078042
14	15	0	3.593659	6.798670	0.313953
15	15	0	-3.593659	-6.798670	0.313953
16	7	0	1.961305	13.568475	-3.279706
17	7	0	-1.961305	-13.568475	-3.279706
18	7	0	6.107527	12.369278	-2.941755
19	7	0	-6.107527	-12.369278	-2.941755
20	6	0	2.937666	1.180645	-1.595166
21	6	0	-2.937666	-1.180645	-1.595166
22	6	0	-2.101519	2.566945	1.558930
23	6	0	2.101519	-2.566945	1.558930
24	6	0	3.301896	1.014790	1.412178
25	6	0	-3.301896	-1.014790	1.412178
26	6	0	-2.045757	2.606142	-1.469274
27	6	0	2.045757	-2.606142	-1.469274
28	6	0	0.276042	3.477590	0.117182
29	6	0	-0.276042	-3.477590	0.117182
30	6	0	1.616095	3.085882	0.208678
31	6	0	-1.616095	-3.085882	0.208678
32	6	0	0.946638	5.817154	0.202335
33	6	0	-0.946638	-5.817154	0.202335
34	6	0	2.299808	5.420709	0.313841
35	6	0	-2.299808	-5.420709	0.313841
36	6	0	-0.058917	4.845726	0.114899
37	6	0	0.058917	-4.845726	0.114899
38	6	0	2.628350	4.059400	0.316920
39	6	0	-2.628350	-4.059400	0.316920
40	6	0	-0.970480	7.798215	-0.899952
41	6	0	0.970480	-7.798215	-0.899952
42	6	0	3.998820	7.104447	2.111207
43	6	0	-3.998820	-7.104447	2.111207
44	6	0	5.116120	6.008922	-0.422916
45	6	0	-5.116120	-6.008922	-0.422916
46	6	0	0.189363	8.215188	1.814176
47	6	0	-0.189363	-8.215188	1.814176
48	6	0	-3.454741	3.230724	-3.819065
49	6	0	3.454741	-3.230724	-3.819065

50	6	0	5.122591	0.669882	3.525886
51	6	0	-5.122591	-0.669882	3.525886
52	6	0	-2.057323	3.371260	-3.778468
53	6	0	2.057323	-3.371260	-3.778468
54	6	0	3.944808	1.410729	3.725795
55	6	0	-3.944808	-1.410729	3.725795
56	6	0	-3.301896	8.074395	-2.443184
57	6	0	3.301896	-8.074395	-2.443184
58	6	0	4.710831	7.681680	4.767703
59	6	0	-4.710831	-7.681680	4.767703
60	6	0	5.266995	6.024304	-1.823546
61	6	0	-5.266995	-6.024304	-1.823546
62	6	0	-0.284888	7.330578	2.800620
63	6	0	0.284888	-7.330578	2.800620
64	6	0	2.230312	0.678254	-2.703039
65	6	0	-2.230312	-0.678254	-2.703039
66	6	0	-1.830973	1.873651	2.752966
67	6	0	1.830973	-1.873651	2.752966
68	6	0	6.421888	5.480661	-2.407121
69	6	0	-6.421888	-5.480661	-2.407121
70	6	0	4.096491	6.457737	4.454231
71	6	0	-4.096491	-6.457737	4.454231
72	6	0	4.604152	8.337817	2.425280
73	6	0	-4.604152	-8.337817	2.425280
74	6	0	-2.215131	7.974350	-0.266245
75	6	0	2.215131	-7.974350	-0.266245
76	6	0	3.743044	6.165195	3.127065
77	6	0	-3.743044	-6.165195	3.127065
78	6	0	-0.607662	7.817021	4.077760
79	6	0	0.607662	-7.817021	4.077760
80	6	0	-2.057323	7.906397	-3.074032
81	6	0	2.057323	-7.906397	-3.074032
82	6	0	4.964546	8.618865	3.752191
83	6	0	-4.964546	-8.618865	3.752191
84	6	0	-2.814981	-0.699651	-3.977682
85	6	0	2.814981	0.699651	-3.977682
86	6	0	2.494920	-2.230466	3.935822
87	6	0	-2.494920	2.230466	3.935822
88	6	0	-4.108019	-1.220096	-4.149281
89	6	0	4.108019	1.220096	-4.149281
90	6	0	3.433440	-3.275746	3.928602
91	6	0	-3.433440	3.275746	3.928602
92	6	0	-0.464524	9.184016	4.369533
93	6	0	0.464524	-9.184016	4.369533
94	6	0	7.429325	4.928343	-1.597243
95	6	0	-7.429325	-4.928343	-1.597243
96	6	0	-3.378994	8.110833	-1.040809
97	6	0	3.378994	-8.110833	-1.040809
98	6	0	-0.890299	7.774396	-2.306443
99	6	0	0.890299	-7.774396	-2.306443
100	6	0	-1.350551	3.059736	-2.608003
101	6	0	1.350551	-3.059736	-2.608003
102	6	0	3.033912	1.582619	2.674218
103	6	0	-3.033912	-1.582619	2.674218
104	6	0	-3.706625	3.964456	2.734824
105	6	0	3.706625	-3.964456	2.734824
106	6	0	-4.145217	2.773498	-2.684847

107	6	0	4.145217	-2.773498	-2.684847
108	6	0	5.384566	0.098894	2.270379
109	6	0	-5.384566	-0.098894	2.270379
110	6	0	4.814997	1.720163	-3.042716
111	6	0	-4.814997	-1.720163	-3.042716
112	6	0	7.280466	4.918971	-0.199645
113	6	0	-7.280466	-4.918971	-0.199645
114	6	0	4.233827	1.707011	-1.766208
115	6	0	-4.233827	-1.707011	-1.766208
116	6	0	-3.040279	3.617746	1.549574
117	6	0	3.040279	-3.617746	1.549574
118	6	0	-3.444070	2.455611	-1.512289
119	6	0	3.444070	-2.455611	-1.512289
120	6	0	4.475670	0.266333	1.213838
121	6	0	-4.475670	-0.266333	1.213838
122	6	0	0.009986	10.065399	3.383723
123	6	0	-0.009986	-10.065399	3.383723
124	6	0	0.344150	9.584763	2.108099
125	6	0	-0.344150	-9.584763	2.108099
126	6	0	6.126854	5.461472	0.390363
127	6	0	-6.126854	-5.461472	0.390363
128	6	0	2.776481	11.351652	-2.177398
129	6	0	-2.776481	-11.351652	-2.177398
130	6	0	4.086163	10.973414	-2.070358
131	6	0	-4.086163	-10.973414	-2.070358
132	6	0	2.349083	12.567455	-2.782557
133	6	0	-2.349083	-12.567455	-2.782557
134	6	0	5.178261	11.749610	-2.551776
135	6	0	-5.178261	-11.749610	-2.551776
136	1	0	1.520536	-3.735734	-4.649288
137	1	0	3.742270	1.859315	4.694015
138	1	0	4.000941	-3.484431	-4.722683
139	1	0	5.832553	0.546411	4.338294
140	1	0	4.200052	-8.206527	-3.040026
141	1	0	4.985035	7.906055	5.794569
142	1	0	2.267506	0.308606	-4.829998
143	1	0	2.285364	-1.693678	4.856234
144	1	0	4.560664	1.239196	-5.136319
145	1	0	3.948329	-3.551305	4.844330
146	1	0	-1.989600	7.916878	-4.157972
147	1	0	-5.434311	-9.568549	3.989641
148	1	0	-4.200052	8.206527	-3.040026
149	1	0	-4.985035	-7.906055	5.794569
150	1	0	-1.520536	3.735734	-4.649288
151	1	0	-3.742270	-1.859315	4.694015
152	1	0	-4.560664	-1.239196	-5.136319
153	1	0	-3.948329	3.551305	4.844330
154	1	0	-4.000941	3.484431	-4.722683
155	1	0	-5.832553	-0.546411	4.338294
156	1	0	-2.267506	-0.308606	-4.829998
157	1	0	-2.285364	1.693678	4.856234
158	1	0	-6.550797	-5.526877	-3.484639
159	1	0	-0.968153	7.133104	4.841457
160	1	0	5.434311	9.568549	3.989641
161	1	0	1.989600	-7.916878	-4.157972
162	1	0	-8.338535	-4.540414	-2.048650
163	1	0	-0.715958	9.559101	5.357537

164	1	0	6.550797	5.526877	-3.484639
165	1	0	0.968153	-7.133104	4.841457
166	1	0	8.338535	4.540414	-2.048650
167	1	0	0.715958	-9.559101	5.357537
168	1	0	0.076195	7.703860	-2.798103
169	1	0	-4.790406	-9.071160	1.643888
170	1	0	5.225843	-2.669500	-2.710751
171	1	0	6.296777	-0.468056	2.110073
172	1	0	4.335488	-8.274801	-0.552277
173	1	0	3.892822	5.736278	5.241049
174	1	0	0.272198	-3.194542	-2.585202
175	1	0	2.133257	2.170031	2.836555
176	1	0	-0.076195	-7.703860	-2.798103
177	1	0	4.790406	9.071160	1.643888
178	1	0	-4.517087	-6.500026	-2.449289
179	1	0	-0.395843	6.269880	2.589269
180	1	0	-1.232868	-0.265888	-2.572144
181	1	0	-1.110418	1.058878	2.760141
182	1	0	-5.811207	-2.131787	-3.170285
183	1	0	-4.430942	4.773568	2.725783
184	1	0	-0.272198	3.194542	-2.585202
185	1	0	-2.133257	-2.170031	2.836555
186	1	0	-5.225843	2.669500	-2.710751
187	1	0	-6.296777	0.468056	2.110073
188	1	0	-4.335488	8.274801	-0.552277
189	1	0	-3.892822	-5.736278	5.241049
190	1	0	4.517087	6.500026	-2.449289
191	1	0	0.395843	-6.269880	2.589269
192	1	0	1.232868	0.265888	-2.572144
193	1	0	1.110418	-1.058878	2.760141
194	1	0	5.811207	2.131787	-3.170285
195	1	0	4.430942	-4.773568	2.725783
196	1	0	-8.071437	-4.521590	0.430222
197	1	0	0.126638	11.121948	3.605271
198	1	0	8.071437	4.521590	0.430222
199	1	0	-0.126638	-11.121948	3.605271
200	1	0	2.275941	-8.045056	0.816515
201	1	0	3.263340	5.217068	2.896736
202	1	0	-3.672359	-3.766297	0.380976
203	1	0	-1.092567	5.161898	0.005009
204	1	0	1.092567	-5.161898	0.005009
205	1	0	3.672359	3.766297	0.380976
206	1	0	0.721198	10.267721	1.350215
207	1	0	-6.038931	-5.491345	1.473168
208	1	0	4.795520	2.110831	-0.928962
209	1	0	3.259062	-4.166581	0.637956
210	1	0	6.038931	5.491345	1.473168
211	1	0	-0.721198	-10.267721	1.350215
212	1	0	-2.275941	8.045056	0.816515
213	1	0	-3.263340	-5.217068	2.896736
214	1	0	-3.991350	2.113645	-0.639709
215	1	0	-4.701292	0.163325	0.242999
216	1	0	-4.795520	-2.110831	-0.928962
217	1	0	-3.259062	4.166581	0.637956
218	1	0	3.991350	-2.113645	-0.639709
219	1	0	4.701292	-0.163325	0.242999

Table S10. D_2 complex tetracyano-[**6**]$^{1+}$
B3PW91/LANL2DZ-optimized
E = -5254.128435 au

Center Number	Atomic Number	Atomic Type	Coordinates (Angstroms) X	Y	Z
1	1	0	1.724905	1.785112	4.728164
2	6	0	0.967387	1.005928	4.722102
3	6	0	-0.967387	-1.005928	4.722102
4	6	0	0.503665	0.493669	3.493222
5	6	0	0.469355	0.525817	5.941632
6	6	0	-0.469355	-0.525817	5.941632
7	6	0	-0.503665	-0.493669	3.493222
8	15	0	1.221615	1.188645	1.887582
9	15	0	0.950237	1.260504	7.609036
10	15	0	-0.950237	-1.260504	7.609036
11	15	0	-1.221615	-1.188645	1.887582
12	1	0	-1.724905	-1.785112	4.728164
13	16	0	1.403596	-0.792281	-10.751493
14	6	0	0.584918	-0.357063	-12.305771
15	6	0	-0.989549	-3.035695	2.145993
16	6	0	-1.240752	0.785047	-13.495905
17	15	0	-1.221615	1.188645	-1.887582
18	6	0	-0.584918	0.357063	-12.305771
19	6	0	0.989549	-3.035695	-2.145993
20	6	0	1.240752	-0.785047	-13.495905
21	16	0	1.403596	0.792281	10.751493
22	6	0	-0.503665	0.493669	-3.493222
23	6	0	0.469355	-0.525817	-5.941632
24	6	0	-0.967387	1.005928	-4.722102
25	6	0	0.503665	-0.493669	-3.493222
26	6	0	0.967387	-1.005928	-4.722102
27	6	0	-0.469355	0.525817	-5.941632
28	1	0	-1.724905	1.785112	-4.728164
29	15	0	1.221615	-1.188645	-1.887582
30	1	0	1.724905	-1.785112	-4.728164
31	15	0	-0.950237	1.260504	-7.609036
32	15	0	0.950237	-1.260504	-7.609036
33	6	0	3.062948	-0.902233	-2.061946
34	79	0	0.000000	0.000000	0.000000
35	6	0	-3.062948	-0.902233	2.061946
36	16	0	-1.403596	0.792281	-10.751493
37	28	0	-0.000000	0.000000	9.218113
38	6	0	0.989549	3.035695	2.145993
39	16	0	-1.403596	-0.792281	10.751493
40	6	0	-1.240752	-0.785047	13.495905
41	6	0	0.584918	0.357063	12.305771
42	6	0	-0.584918	-0.357063	12.305771
43	6	0	1.240752	0.785047	13.495905
44	28	0	0.000000	0.000000	-9.218113
45	6	0	3.062948	0.902233	2.061946
46	6	0	-3.062948	0.902233	-2.061946
47	6	0	-0.989549	3.035695	-2.145993
48	6	0	-2.792421	1.531800	-7.519386
49	6	0	-0.171893	2.966106	-7.608104

50	6	0	2.792421	-1.531800	-7.519386
51	6	0	0.171893	-2.966106	-7.608104
52	6	0	-2.792421	-1.531800	7.519386
53	6	0	-0.171893	-2.966106	7.608104
54	6	0	2.792421	1.531800	7.519386
55	6	0	0.171893	2.966106	7.608104
56	7	0	1.807358	1.157870	14.465688
57	7	0	-1.807358	-1.157870	14.465688
58	7	0	-1.807358	1.157870	-14.465688
59	7	0	1.807358	-1.157870	-14.465688
60	6	0	-0.483670	-5.774494	2.557385
61	6	0	-1.973191	-3.854412	2.732448
62	6	0	0.245075	-3.593565	1.762913
63	6	0	0.499381	-4.957181	1.974199
64	6	0	-1.719469	-5.221332	2.931621
65	1	0	-2.933283	-3.439563	3.027265
66	1	0	1.000274	-2.967667	1.292496
67	1	0	1.452635	-5.381552	1.671882
68	1	0	-2.482744	-5.849619	3.382173
69	1	0	-0.291111	-6.832096	2.714555
70	6	0	-0.483670	5.774494	-2.557385
71	6	0	0.245075	3.593565	-1.762913
72	6	0	-1.973191	3.854412	-2.732448
73	6	0	-1.719469	5.221332	-2.931621
74	6	0	0.499381	4.957181	-1.974199
75	1	0	1.000274	2.967667	-1.292496
76	1	0	-2.933283	3.439563	-3.027265
77	1	0	-2.482744	5.849619	-3.382173
78	1	0	1.452635	5.381552	-1.671882
79	1	0	-0.291111	6.832096	-2.714555
80	6	0	5.844957	-0.467661	-2.190917
81	6	0	3.590856	0.047176	-2.956147
82	6	0	3.934478	-1.613917	-1.211808
83	6	0	5.318357	-1.402810	-1.283235
84	6	0	4.978010	0.260468	-3.020120
85	1	0	2.937639	0.611891	-3.614731
86	1	0	3.541291	-2.333217	-0.497253
87	1	0	5.981131	-1.963269	-0.630267
88	1	0	5.376285	0.984562	-3.725297
89	1	0	6.918139	-0.310123	-2.250991
90	6	0	5.844957	0.467661	2.190917
91	6	0	3.934478	1.613917	1.211808
92	6	0	3.590856	-0.047176	2.956147
93	6	0	4.978010	-0.260468	3.020120
94	6	0	5.318357	1.402810	1.283235
95	1	0	3.541291	2.333217	0.497253
96	1	0	2.937639	-0.611891	3.614731
97	1	0	5.376285	-0.984562	3.725297
98	1	0	5.981131	1.963269	0.630267
99	1	0	6.918139	0.310123	2.250991
100	6	0	-5.844957	-0.467661	2.190917
101	6	0	-3.590856	0.047176	2.956147
102	6	0	-3.934478	-1.613917	1.211808
103	6	0	-5.318357	-1.402810	1.283235
104	6	0	-4.978010	0.260468	3.020120
105	1	0	-2.937639	0.611891	3.614731
106	1	0	-3.541291	-2.333217	0.497253

107	1	0	-5.981131	-1.963269	0.630267
108	1	0	-5.376285	0.984562	3.725297
109	1	0	-6.918139	-0.310123	2.250991
110	6	0	0.483670	-5.774494	-2.557385
111	6	0	1.973191	-3.854412	-2.732448
112	6	0	-0.245075	-3.593565	-1.762913
113	6	0	-0.499381	-4.957181	-1.974199
114	6	0	1.719469	-5.221332	-2.931621
115	1	0	2.933283	-3.439563	-3.027265
116	1	0	-1.000274	-2.967667	-1.292496
117	1	0	-1.452635	-5.381552	-1.671882
118	1	0	2.482744	-5.849619	-3.382173
119	1	0	0.291111	-6.832096	-2.714555
120	6	0	-5.844957	0.467661	-2.190917
121	6	0	-3.934478	1.613917	-1.211808
122	6	0	-3.590856	-0.047176	-2.956147
123	6	0	-4.978010	-0.260468	-3.020120
124	6	0	-5.318357	1.402810	-1.283235
125	1	0	-3.541291	2.333217	-0.497253
126	1	0	-2.937639	-0.611891	-3.614731
127	1	0	-5.376285	-0.984562	-3.725297
128	1	0	-5.981131	1.963269	-0.630267
129	1	0	-6.918139	0.310123	-2.250991
130	6	0	0.483670	5.774494	2.557385
131	6	0	-0.245075	3.593565	1.762913
132	6	0	1.973191	3.854412	2.732448
133	6	0	1.719469	5.221332	2.931621
134	6	0	-0.499381	4.957181	1.974199
135	1	0	-1.000274	2.967667	1.292496
136	1	0	2.933283	3.439563	3.027265
137	1	0	2.482744	5.849619	3.382173
138	1	0	-1.452635	5.381552	1.671882
139	1	0	0.291111	6.832096	2.714555
140	6	0	-5.579461	-1.863411	7.444928
141	6	0	-3.341811	-2.750141	7.078729
142	6	0	-3.635709	-0.484365	7.937660
143	6	0	-5.028292	-0.651256	7.893775
144	6	0	-4.735944	-2.912314	7.041822
145	1	0	-2.692259	-3.575243	6.796942
146	1	0	-3.211281	0.437310	8.327201
147	1	0	-5.676944	0.150603	8.234462
148	1	0	-5.160806	-3.858211	6.717198
149	1	0	-6.657614	-1.997520	7.430254
150	6	0	0.990750	-5.525530	7.768145
151	6	0	0.492363	-3.510419	6.493623
152	6	0	-0.245736	-3.703543	8.808295
153	6	0	0.328063	-4.981253	8.881746
154	6	0	1.072961	-4.787242	6.577123
155	1	0	0.571807	-2.955427	5.563851
156	1	0	-0.732450	-3.278970	9.683062
157	1	0	0.267852	-5.540644	9.810683
158	1	0	1.586660	-5.199351	5.712887
159	1	0	1.443529	-6.511164	7.832246
160	6	0	-0.990750	5.525530	7.768145
161	6	0	0.245736	3.703543	8.808295
162	6	0	-0.492363	3.510419	6.493623
163	6	0	-1.072961	4.787242	6.577123

164	6	0	-0.328063	4.981253	8.881746
165	1	0	0.732450	3.278970	9.683062
166	1	0	-0.571807	2.955427	5.563851
167	1	0	-1.586660	5.199351	5.712887
168	1	0	-0.267852	5.540644	9.810683
169	1	0	-1.443529	6.511164	7.832246
170	6	0	5.579461	1.863411	7.444928
171	6	0	3.635709	0.484365	7.937660
172	6	0	3.341811	2.750141	7.078729
173	6	0	4.735944	2.912314	7.041822
174	6	0	5.028292	0.651256	7.893775
175	1	0	3.211281	-0.437310	8.327201
176	1	0	2.692259	3.575243	6.796942
177	1	0	5.160806	3.858211	6.717198
178	1	0	5.676944	-0.150603	8.234462
179	1	0	6.657614	1.997520	7.430254
180	6	0	-0.990750	-5.525530	-7.768145
181	6	0	-0.492363	-3.510419	-6.493623
182	6	0	0.245736	-3.703543	-8.808295
183	6	0	-0.328063	-4.981253	-8.881746
184	6	0	-1.072961	-4.787242	-6.577123
185	1	0	-0.571807	-2.955427	-5.563851
186	1	0	0.732450	-3.278970	-9.683062
187	1	0	-0.267852	-5.540644	-9.810683
188	1	0	-1.586660	-5.199351	-5.712887
189	1	0	-1.443529	-6.511164	-7.832246
190	6	0	5.579461	-1.863411	-7.444928
191	6	0	3.341811	-2.750141	-7.078729
192	6	0	3.635709	-0.484365	-7.937660
193	6	0	5.028292	-0.651256	-7.893775
194	6	0	4.735944	-2.912314	-7.041822
195	1	0	2.692259	-3.575243	-6.796942
196	1	0	3.211281	0.437310	-8.327201
197	1	0	5.676944	0.150603	-8.234462
198	1	0	5.160806	-3.858211	-6.717198
199	1	0	6.657614	-1.997520	-7.430254
200	6	0	-5.579461	1.863411	-7.444928
201	6	0	-3.635709	0.484365	-7.937660
202	6	0	-3.341811	2.750141	-7.078729
203	6	0	-4.735944	2.912314	-7.041822
204	6	0	-5.028292	0.651256	-7.893775
205	1	0	-3.211281	-0.437310	-8.327201
206	1	0	-2.692259	3.575243	-6.796942
207	1	0	-5.160806	3.858211	-6.717198
208	1	0	-5.676944	-0.150603	-8.234462
209	1	0	-6.657614	1.997520	-7.430254
210	6	0	0.990750	5.525530	-7.768145
211	6	0	-0.245736	3.703543	-8.808295
212	6	0	0.492363	3.510419	-6.493623
213	6	0	1.072961	4.787242	-6.577123
214	6	0	0.328063	4.981253	-8.881746
215	1	0	-0.732450	3.278970	-9.683062
216	1	0	0.571807	2.955427	-5.563851
217	1	0	1.586660	5.199351	-5.712887
218	1	0	0.267852	5.540644	-9.810683
219	1	0	1.443529	6.511164	-7.832246

Table S11. C_1 complex tetracyano-[**6**]$^{1+}$
B3PW91/LANL2DZ-optimized
E = -5254.130774 au

Center Number	Atomic Number	Atomic Type	Coordinates (Angstroms) X	Y	Z
1	79	0	-0.000003	0.349852	0.000020
2	28	0	9.102870	-0.087754	-0.278446
3	28	0	-9.102879	-0.087740	0.278394
4	16	0	-10.636057	1.434251	-0.235842
5	16	0	10.636051	1.434264	0.235697
6	16	0	-10.470855	-1.131945	1.682939
7	16	0	10.470816	-1.132003	-1.682987
8	15	0	1.797914	-0.960294	-1.243101
9	15	0	-1.797930	-0.960235	1.243192
10	15	0	1.992232	1.793589	0.741174
11	15	0	-1.992224	1.793577	-0.741183
12	15	0	7.465454	-1.610867	-0.694253
13	15	0	-7.465465	-1.610830	0.694292
14	15	0	-7.647088	0.863523	-1.184864
15	15	0	7.647104	0.863557	1.184807
16	7	0	-14.004464	-1.099281	2.950745
17	7	0	14.004464	-1.099292	-2.950833
18	7	0	-14.228519	2.342273	0.370708
19	7	0	14.228551	2.342214	-0.371030
20	6	0	-1.981744	-0.776670	3.099401
21	6	0	1.981717	-0.776803	-3.099318
22	6	0	1.935175	-2.811160	-0.948229
23	6	0	-1.935198	-2.811113	0.948391
24	6	0	2.242128	2.333956	2.518268
25	6	0	-2.242110	2.333885	-2.518296
26	6	0	-2.284616	3.350631	0.263571
27	6	0	2.284628	3.350609	-0.263633
28	6	0	3.523664	0.758894	0.325257
29	6	0	-3.523663	0.758902	-0.325241
30	6	0	3.440346	-0.370409	-0.518570
31	6	0	-3.440356	-0.370372	0.518628
32	6	0	5.932054	0.389821	0.566088
33	6	0	-5.932049	0.389817	-0.566093
34	6	0	4.606926	-1.110843	-0.806579
35	6	0	-4.606940	-1.110794	0.806649
36	6	0	4.775982	1.123329	0.860426
37	6	0	-4.775973	1.123316	-0.860441
38	6	0	5.848754	-0.736734	-0.278743
39	6	0	-5.848760	-0.736705	0.278782
40	6	0	-7.579660	2.715090	-1.400487
41	6	0	7.579672	2.715132	1.400361
42	6	0	7.773292	0.146643	2.905373
43	6	0	-7.773237	0.146543	-2.905406
44	6	0	-7.210031	-2.282654	2.416636
45	6	0	7.209987	-2.282757	-2.416566
46	6	0	7.537400	-3.100146	0.431829
47	6	0	-7.537382	-3.100152	-0.431735
48	6	0	2.167115	-5.555661	-0.363553
49	6	0	-2.167144	-5.555635	0.363817

50	6	0	1.864712	-4.640651	0.660104
51	6	0	-1.864699	-4.640669	-0.659867
52	6	0	7.777875	-5.369824	2.072362
53	6	0	-7.777814	-5.369894	-2.072186
54	6	0	6.809594	-0.642144	4.995451
55	6	0	-6.809492	-0.642311	-4.995437
56	6	0	-7.701150	4.920621	-0.379841
57	6	0	7.701128	4.920625	0.379631
58	6	0	1.616489	2.040026	4.855900
59	6	0	-1.616446	2.039889	-4.855913
60	6	0	2.517285	3.050970	5.227988
61	6	0	-2.517250	3.050810	-5.228041
62	6	0	-2.616817	5.684074	1.811527
63	6	0	2.616841	5.684000	-1.811664
64	6	0	2.350068	-5.096174	-1.677255
65	6	0	-2.350136	-5.096094	1.677495
66	6	0	-2.106685	-0.532330	5.907473
67	6	0	2.106646	-0.532591	-5.907402
68	6	0	8.098497	-0.883073	5.500335
69	6	0	-8.098384	-0.883270	-5.500337
70	6	0	-0.976521	-1.107687	5.299768
71	6	0	0.976509	-1.107973	-5.299669
72	6	0	-7.544517	5.514005	-1.644453
73	6	0	7.544519	5.514056	1.644224
74	6	0	6.520440	-4.777002	1.871073
75	6	0	-6.520385	-4.777059	-1.870893
76	6	0	8.916328	-4.823955	1.455685
77	6	0	-8.916281	-4.824005	-1.455553
78	6	0	-3.137186	4.459345	2.260170
79	6	0	3.137171	4.459247	-2.260286
80	6	0	8.801500	-3.687257	0.640813
81	6	0	-8.801475	-3.687276	-0.640722
82	6	0	6.396631	-3.644344	1.050079
83	6	0	-6.396597	-3.644369	-1.049940
84	6	0	1.742900	-3.274913	0.367940
85	6	0	-1.742885	-3.274920	-0.367754
86	6	0	-3.168436	-0.078513	5.110187
87	6	0	3.168375	-0.078686	-5.110139
88	6	0	-6.891951	-3.233877	5.041333
89	6	0	6.891856	-3.234080	-5.041221
90	6	0	-7.483305	-1.437230	3.509842
91	6	0	7.483230	-1.437372	-3.509810
92	6	0	-0.910367	-1.220741	3.903383
93	6	0	0.910361	-1.220963	-3.903279
94	6	0	-7.317973	-1.913127	4.819243
95	6	0	7.317873	-1.913319	-4.819190
96	6	0	-7.414163	4.706718	-2.786252
97	6	0	7.414194	4.706811	2.786056
98	6	0	-7.727996	3.523911	-0.256517
99	6	0	7.727979	3.523911	0.256358
100	6	0	-2.973415	3.297650	1.489885
101	6	0	2.973394	3.297578	-1.489963
102	6	0	-1.922622	5.737889	0.591556
103	6	0	1.922681	5.737865	-0.591675
104	6	0	1.476511	1.685435	3.504645
105	6	0	-1.476476	1.685343	-3.504645
106	6	0	3.273379	3.710095	4.243418

107	6	0	-3.273362	3.709956	-4.243500
108	6	0	6.644222	-0.125829	3.700220
109	6	0	-6.644150	-0.125946	-3.700222
110	6	0	9.224510	-0.612255	4.705160
111	6	0	-9.224414	-0.612433	-4.705193
112	6	0	-3.110383	-0.203319	3.711611
113	6	0	3.110329	-0.203429	-3.711557
114	6	0	2.238504	-3.726297	-1.972041
115	6	0	-2.238569	-3.726206	1.972229
116	6	0	-9.066200	-0.103072	-3.406841
117	6	0	9.066267	-0.102942	3.406793
118	6	0	-6.791453	-3.607687	2.637170
119	6	0	6.791415	-3.607802	-2.637042
120	6	0	-6.632494	-4.079757	3.950451
121	6	0	6.632430	-4.079922	-3.950301
122	6	0	-7.430380	3.306877	-2.667783
123	6	0	7.430416	3.306965	2.667639
124	6	0	-1.747439	4.576424	-0.176832
125	6	0	1.747491	4.576427	0.176750
126	6	0	3.138319	3.355899	2.892551
127	6	0	-3.138312	3.355804	-2.892621
128	6	0	-12.019011	-0.207306	1.515158
129	6	0	12.018972	-0.207351	-1.515275
130	6	0	-13.266612	1.671580	0.530081
131	6	0	13.266587	1.671576	-0.530294
132	6	0	-12.089537	0.886972	0.695734
133	6	0	12.089512	0.886957	-0.695892
134	6	0	-13.113983	-0.680906	2.293049
135	6	0	13.113928	-0.680976	-2.293173
136	1	0	1.711541	-4.991700	1.676773
137	1	0	-1.711497	-4.991760	-1.676517
138	1	0	2.257225	-6.614855	-0.139205
139	1	0	-2.257256	-6.614838	0.139508
140	1	0	7.871469	-6.246139	2.707925
141	1	0	-7.871390	-6.246234	-2.707717
142	1	0	-0.152702	-1.468515	5.909554
143	1	0	0.152707	-1.468868	-5.909438
144	1	0	-2.159418	-0.443410	6.988822
145	1	0	2.159374	-0.443719	-6.988755
146	1	0	-7.830405	5.541589	0.501766
147	1	0	7.830360	5.541561	-0.502003
148	1	0	-7.547094	6.596279	-1.740709
149	1	0	7.547092	6.596334	1.740439
150	1	0	-3.674504	4.408171	3.203018
151	1	0	3.674463	4.408034	-3.203146
152	1	0	2.620980	3.332123	6.272338
153	1	0	-2.620939	3.331929	-6.272401
154	1	0	-2.753249	6.584987	2.403010
155	1	0	2.753279	6.584893	-2.403175
156	1	0	1.013056	1.540338	5.608125
157	1	0	-1.012999	1.540184	-5.608117
158	1	0	5.936470	-0.853802	5.606758
159	1	0	-5.936354	-0.853982	-5.606720
160	1	0	-9.892168	-5.274235	-1.611841
161	1	0	9.892219	-5.274174	1.611970
162	1	0	8.224483	-1.281883	6.503163
163	1	0	-8.224347	-1.282118	-6.503152

164	1	0	-7.553146	-1.266935	5.660180
165	1	0	7.553022	-1.267157	-5.660155
166	1	0	-6.786018	-3.607449	6.056177
167	1	0	6.785904	-3.607691	-6.056048
168	1	0	-7.899129	3.070263	0.715978
169	1	0	7.899093	3.070228	-0.716123
170	1	0	2.586789	-5.798132	-2.472092
171	1	0	-2.586890	-5.798018	2.472352
172	1	0	5.638588	-5.192973	2.350716
173	1	0	-5.638521	-5.193045	-2.350502
174	1	0	1.494616	-2.572130	1.160134
175	1	0	-1.494568	-2.572171	-1.159967
176	1	0	9.686207	-3.259407	0.174703
177	1	0	-9.686193	-3.259412	-0.174647
178	1	0	5.641754	0.051803	3.320198
179	1	0	-5.641690	0.051709	-3.320188
180	1	0	0.757739	0.922442	3.217513
181	1	0	-0.757697	0.922368	-3.217481
182	1	0	3.963006	4.501119	4.523464
183	1	0	-3.962997	4.500962	-4.523578
184	1	0	-3.393785	2.361745	1.846246
185	1	0	3.393734	2.361653	-1.846308
186	1	0	-1.521277	6.682154	0.234086
187	1	0	1.521367	6.682151	-0.234221
188	1	0	-7.322229	5.162339	-3.768426
189	1	0	7.322278	5.162469	3.768215
190	1	0	-7.864681	-0.433711	3.341580
191	1	0	7.864602	-0.433843	-3.341594
192	1	0	-0.027583	-1.660493	3.443695
193	1	0	0.027599	-1.660736	-3.443569
194	1	0	-4.047287	0.361883	5.572173
195	1	0	4.047205	0.361730	-5.572145
196	1	0	10.223074	-0.800593	5.088350
197	1	0	-10.222969	-0.800796	-5.088395
198	1	0	-6.327267	-5.108784	4.119122
199	1	0	6.327207	-5.108957	-4.118927
200	1	0	5.418433	-3.192842	0.908369
201	1	0	-5.418404	-3.192858	-0.908226
202	1	0	4.858060	1.998642	1.497015
203	1	0	-4.858041	1.998604	-1.497067
204	1	0	4.545669	-1.976329	-1.461549
205	1	0	-4.545691	-1.976259	1.461649
206	1	0	-9.938519	0.098799	-2.789436
207	1	0	9.938573	0.098914	2.789364
208	1	0	-3.952462	0.134279	3.115301
209	1	0	3.952392	0.134239	-3.115263
210	1	0	-6.616388	-4.276097	1.798496
211	1	0	6.616374	-4.276181	-1.798338
212	1	0	-7.352060	2.689093	-3.558023
213	1	0	7.352118	2.689214	3.557903
214	1	0	-1.200153	4.633916	-1.113427
215	1	0	1.200233	4.633958	1.113359
216	1	0	3.711638	3.894682	2.142761
217	1	0	-3.711647	3.894603	-2.142853
218	1	0	2.395266	-3.383703	-2.990814
219	1	0	-2.395363	-3.383570	2.990983

Table S12. C_2 complex tetracyano-[**6**]$^{1+}$
B3PW91/LANL2DZ-optimized
E = -5254.136544 au

Center Number	Atomic Number	Atomic Type	Coordinates (Angstroms) X	Y	Z
1	79	0	-0.000000	0.000000	-1.198041
2	28	0	-0.290557	-8.842871	0.875813
3	28	0	0.290557	8.842871	0.875813
4	16	0	-0.452842	10.632235	-0.210464
5	16	0	0.452842	-10.632235	-0.210464
6	16	0	1.795340	10.030899	1.998585
7	16	0	-1.795340	-10.030899	1.998585
8	15	0	1.619718	1.609867	-0.037349
9	15	0	-1.619718	-1.609867	-0.037349
10	15	0	-0.810546	2.196373	-2.242394
11	15	0	0.810546	-2.196373	-2.242394
12	15	0	-1.247503	7.539380	-0.178179
13	15	0	1.247503	-7.539380	-0.178179
14	15	0	0.928340	6.956285	1.969694
15	15	0	-0.928340	-6.956285	1.969694
16	7	0	-0.050834	14.360462	-0.400463
17	7	0	0.050834	-14.360462	-0.400463
18	7	0	2.965379	13.552474	2.566582
19	7	0	-2.965379	-13.552474	2.566582
20	6	0	3.170894	2.042311	-1.001420
21	6	0	-3.170894	-2.042311	-1.001420
22	6	0	-2.634292	2.577119	-2.468691
23	6	0	2.634292	-2.577119	-2.468691
24	6	0	2.175454	1.387997	1.735583
25	6	0	-2.175454	-1.387997	1.735583
26	6	0	-0.008989	2.705851	-3.854613
27	6	0	0.008989	-2.705851	-3.854613
28	6	0	-0.286120	3.521856	-1.004183
29	6	0	0.286120	-3.521856	-1.004183
30	6	0	0.704021	3.261202	-0.030760
31	6	0	-0.704021	-3.261202	-0.030760
32	6	0	-0.545526	5.791729	-0.130906
33	6	0	0.545526	-5.791729	-0.130906
34	6	0	0.435653	5.526674	0.846661
35	6	0	-0.435653	-5.526674	0.846661
36	6	0	-0.904052	4.788141	-1.041686
37	6	0	0.904052	-4.788141	-1.041686
38	6	0	1.049051	4.267637	0.892510
39	6	0	-1.049051	-4.267637	0.892510
40	6	0	-1.670462	7.832298	-1.971070
41	6	0	1.670462	-7.832298	-1.971070
42	6	0	0.000000	6.688330	3.568967
43	6	0	0.000000	-6.688330	3.568967
44	6	0	2.724362	6.640026	2.365347
45	6	0	-2.724362	-6.640026	2.365347
46	6	0	-2.876194	7.449066	0.733317
47	6	0	2.876194	-7.449066	0.733317
48	6	0	1.176566	3.352680	-6.327591
49	6	0	-1.176566	-3.352680	-6.327591

50	6	0	2.927485	0.894191	4.407409
51	6	0	-2.927485	-0.894191	4.407409
52	6	0	1.444162	4.131326	-5.191240
53	6	0	-1.444162	-4.131326	-5.191240
54	6	0	1.598280	1.232483	4.098976
55	6	0	-1.598280	-1.232483	4.098976
56	6	0	-2.229597	8.335993	-4.677642
57	6	0	2.229597	-8.335993	-4.677642
58	6	0	-1.359169	6.410368	6.014359
59	6	0	1.359169	-6.410368	6.014359
60	6	0	3.700778	7.127081	1.474557
61	6	0	-3.700778	-7.127081	1.474557
62	6	0	-3.610229	6.255128	0.861628
63	6	0	3.610229	-6.255128	0.861628
64	6	0	3.422481	1.363815	-2.206523
65	6	0	-3.422481	-1.363815	-2.206523
66	6	0	-3.515972	2.185162	-1.440956
67	6	0	3.515972	-2.185162	-1.440956
68	6	0	5.061019	6.911387	1.743724
69	6	0	-5.061019	-6.911387	1.743724
70	6	0	-1.333890	5.346048	5.098480
71	6	0	1.333890	-5.346048	5.098480
72	6	0	-0.035563	7.762297	4.481284
73	6	0	0.035563	-7.762297	4.481284
74	6	0	-2.983077	7.686336	-2.454993
75	6	0	2.983077	-7.686336	-2.454993
76	6	0	-0.652763	5.481319	3.877539
77	6	0	0.652763	-5.481319	3.877539
78	6	0	-4.838915	6.259780	1.541254
79	6	0	4.838915	-6.259780	1.541254
80	6	0	-0.920045	8.488747	-4.190400
81	6	0	0.920045	-8.488747	-4.190400
82	6	0	-0.708584	7.616822	5.703793
83	6	0	0.708584	-7.616822	5.703793
84	6	0	-4.553452	-1.685584	-2.974969
85	6	0	4.553452	1.685584	-2.974969
86	6	0	4.888393	-2.452486	-1.549766
87	6	0	-4.888393	2.452486	-1.549766
88	6	0	-5.435470	-2.687625	-2.542484
89	6	0	5.435470	2.687625	-2.542484
90	6	0	5.392237	-3.100502	-2.691423
91	6	0	-5.392237	3.100502	-2.691423
92	6	0	-5.338537	7.453139	2.089248
93	6	0	5.338537	-7.453139	2.089248
94	6	0	5.449807	6.219636	2.903951
95	6	0	-5.449807	-6.219636	2.903951
96	6	0	-3.259100	7.938901	-3.808914
97	6	0	3.259100	-7.938901	-3.808914
98	6	0	-0.639501	8.245050	-2.837981
99	6	0	0.639501	-8.245050	-2.837981
100	6	0	0.850809	3.813643	-3.957692
101	6	0	-0.850809	-3.813643	-3.957692
102	6	0	1.220018	1.468569	2.769154
103	6	0	-1.220018	-1.468569	2.769154
104	6	0	-4.515953	3.484641	-3.718848
105	6	0	4.515953	-3.484641	-3.718848
106	6	0	0.317783	2.243606	-6.226566

107	6	0	-0.317783	-2.243606	-6.226566
108	6	0	3.875634	0.792019	3.376946
109	6	0	-3.875634	-0.792019	3.376946
110	6	0	5.183114	3.371510	-1.339423
111	6	0	-5.183114	-3.371510	-1.339423
112	6	0	4.475158	5.744028	3.796990
113	6	0	-4.475158	-5.744028	3.796990
114	6	0	4.053845	3.054239	-0.570941
115	6	0	-4.053845	-3.054239	-0.570941
116	6	0	-3.138466	3.227956	-3.609293
117	6	0	3.138466	-3.227956	-3.609293
118	6	0	-0.265138	1.914276	-4.994115
119	6	0	0.265138	-1.914276	-4.994115
120	6	0	3.503372	1.037259	2.044697
121	6	0	-3.503372	-1.037259	2.044697
122	6	0	-4.603990	8.643816	1.961710
123	6	0	4.603990	-8.643816	1.961710
124	6	0	-3.371539	8.644931	1.290017
125	6	0	3.371539	-8.644931	1.290017
126	6	0	3.112071	5.953975	3.531236
127	6	0	-3.112071	-5.953975	3.531236
128	6	0	0.511384	11.983811	0.513008
129	6	0	-0.511384	-11.983811	0.513008
130	6	0	1.470259	11.727877	1.455818
131	6	0	-1.470259	-11.727877	1.455818
132	6	0	0.217192	13.288926	0.024320
133	6	0	-0.217192	-13.288926	0.024320
134	6	0	2.278765	12.737346	2.052074
135	6	0	-2.278765	-12.737346	2.052074
136	1	0	-2.108675	-4.987709	-5.261029
137	1	0	0.858451	1.314430	4.890402
138	1	0	-1.629892	-3.605958	-7.281778
139	1	0	3.220120	0.714633	5.438176
140	1	0	2.449023	-8.546528	-5.720798
141	1	0	-1.887237	6.304989	6.958083
142	1	0	4.741622	1.155228	-3.903898
143	1	0	5.563300	-2.146647	-0.755175
144	1	0	6.310808	2.937019	-3.135840
145	1	0	6.456281	-3.301385	-2.779200
146	1	0	-0.127380	8.824916	-4.852563
147	1	0	0.732008	-8.446768	6.403876
148	1	0	-2.449023	8.546528	-5.720798
149	1	0	1.887237	-6.304989	6.958083
150	1	0	2.108675	4.987709	-5.261029
151	1	0	-0.858451	-1.314430	4.890402
152	1	0	-6.310808	-2.937019	-3.135840
153	1	0	-6.456281	3.301385	-2.779200
154	1	0	1.629892	3.605958	-7.281778
155	1	0	-3.220120	-0.714633	5.438176
156	1	0	-4.741622	-1.155228	-3.903898
157	1	0	-5.563300	2.146647	-0.755175
158	1	0	-5.812221	-7.310373	1.068043
159	1	0	-5.403277	5.336453	1.640886
160	1	0	-0.732008	8.446768	6.403876
161	1	0	0.127380	-8.824916	-4.852563
162	1	0	-6.504221	-6.073067	3.121906
163	1	0	-6.289981	7.454989	2.614023

164	1	0	5.812221	7.310373	1.068043
165	1	0	5.403277	-5.336453	1.640886
166	1	0	6.504221	6.073067	3.121906
167	1	0	6.289981	-7.454989	2.614023
168	1	0	0.363003	8.413619	-2.453947
169	1	0	-0.449514	-8.704565	4.236503
170	1	0	-0.102288	-1.640295	-7.104178
171	1	0	4.904750	0.530537	3.607202
172	1	0	4.276933	-7.843572	-4.176836
173	1	0	-1.846365	4.416068	5.330286
174	1	0	-1.063843	-4.430828	-3.090303
175	1	0	0.184175	1.709956	2.542146
176	1	0	-0.363003	-8.413619	-2.453947
177	1	0	0.449514	8.704565	4.236503
178	1	0	-3.405213	-7.703883	0.602617
179	1	0	-3.233637	5.324487	0.445646
180	1	0	-2.737092	-0.591595	-2.545563
181	1	0	-3.134029	1.670830	-0.561495
182	1	0	-5.860108	-4.150922	-1.001749
183	1	0	-4.898860	3.987695	-4.602282
184	1	0	1.063843	4.430828	-3.090303
185	1	0	-0.184175	-1.709956	2.542146
186	1	0	0.102288	1.640295	-7.104178
187	1	0	-4.904750	-0.530537	3.607202
188	1	0	-4.276933	7.843572	-4.176836
189	1	0	1.846365	-4.416068	5.330286
190	1	0	3.405213	7.703883	0.602617
191	1	0	3.233637	-5.324487	0.445646
192	1	0	2.737092	0.591595	-2.545563
193	1	0	3.134029	-1.670830	-0.561495
194	1	0	5.860108	4.150922	-1.001749
195	1	0	4.898860	-3.987695	-4.602282
196	1	0	-4.772967	-5.226702	4.704661
197	1	0	-4.982662	9.568582	2.386950
198	1	0	4.772967	5.226702	4.704661
199	1	0	4.982662	-9.568582	2.386950
200	1	0	3.789347	-7.403369	-1.783983
201	1	0	-0.647244	4.654697	3.172129
202	1	0	-1.812400	-4.074596	1.641430
203	1	0	-1.652786	4.998309	-1.801186
204	1	0	1.652786	-4.998309	-1.801186
205	1	0	1.812400	4.074596	1.641430
206	1	0	-2.800260	9.565945	1.199643
207	1	0	-2.364274	-5.606648	4.239167
208	1	0	3.875006	3.595630	0.353886
209	1	0	2.469981	-3.540052	-4.406326
210	1	0	2.364274	5.606648	4.239167
211	1	0	2.800260	-9.565945	1.199643
212	1	0	-3.789347	7.403369	-1.783983
213	1	0	0.647244	-4.654697	3.172129
214	1	0	-0.920159	1.048585	-4.924825
215	1	0	-4.250242	-0.966591	1.259005
216	1	0	-3.875006	-3.595630	0.353886
217	1	0	-2.469981	3.540052	-4.406326
218	1	0	0.920159	-1.048585	-4.924825
219	1	0	4.250242	0.966591	1.259005

Table S13. D_{2h} complex [**1**]$^{2+}$
B3PW91/Def2SVP-optimized
E = -12142.070835 au

Center Number	Atomic Number	Atomic Type	Coordinates (Angstroms) X	Y	Z
1	78	0	0.000000	0.000000	0.000000
2	28	0	8.987682	-0.000000	-0.000000
3	28	0	-8.987682	0.000000	-0.000000
4	16	0	-10.507209	1.551097	0.000000
5	16	0	10.507209	-1.551097	-0.000000
6	16	0	-10.507209	-1.551097	-0.000000
7	16	0	10.507209	1.551097	0.000000
8	15	0	1.761819	-1.637578	0.000000
9	15	0	1.761819	1.637578	0.000000
10	15	0	-1.761819	-1.637578	-0.000000
11	15	0	-1.761819	1.637578	0.000000
12	15	0	-7.437143	-1.552613	0.000000
13	15	0	-7.437143	1.552613	0.000000
14	15	0	7.437143	-1.552613	0.000000
15	15	0	7.437143	1.552613	0.000000
16	7	0	14.149770	-2.158164	0.000000
17	7	0	14.149770	2.158164	0.000000
18	7	0	-14.149770	-2.158164	-0.000000
19	7	0	-14.149770	2.158164	0.000000
20	6	0	3.341706	-0.699179	0.000000
21	6	0	5.777559	-0.704719	0.000000
22	6	0	5.777559	0.704719	0.000000
23	6	0	3.341706	0.699179	0.000000
24	6	0	-3.341706	-0.699179	-0.000000
25	6	0	-5.777559	0.704719	0.000000
26	6	0	-3.341706	0.699179	0.000000
27	6	0	-5.777559	-0.704719	-0.000000
28	6	0	4.560279	-1.391165	0.000000
29	6	0	4.560279	1.391165	0.000000
30	6	0	-13.207957	-1.476574	0.000000
31	6	0	13.207957	1.476574	0.000000
32	6	0	13.207957	-1.476574	0.000000
33	6	0	-13.207957	1.476574	0.000000
34	6	0	12.025167	-0.684517	0.000000
35	6	0	12.025167	0.684517	0.000000
36	6	0	-12.025167	-0.684517	-0.000000
37	6	0	-12.025167	0.684517	0.000000
38	6	0	-4.560279	-1.391165	-0.000000
39	6	0	-4.560279	1.391165	0.000000
40	6	0	1.933254	2.648454	1.534323
41	6	0	-1.933254	2.648454	-1.534323
42	6	0	-1.933254	2.648454	1.534323
43	6	0	1.933254	2.648454	-1.534323
44	6	0	1.933254	-2.648454	-1.534323
45	6	0	1.933254	-2.648454	1.534323
46	6	0	-1.933254	-2.648454	-1.534323
47	6	0	-1.933254	-2.648454	1.534323
48	6	0	-7.396761	-2.593022	-1.517211
49	6	0	-7.396761	-2.593022	1.517211

50	6	0	-7.396761	2.593022	1.517211
51	6	0	-7.396761	2.593022	-1.517211
52	6	0	7.396761	2.593022	-1.517211
53	6	0	7.396761	2.593022	1.517211
54	6	0	7.396761	-2.593022	1.517211
55	6	0	7.396761	-2.593022	-1.517211
56	6	0	2.345611	3.969226	3.996091
57	6	0	-2.345611	-3.969226	3.996091
58	6	0	-2.345611	3.969226	3.996091
59	6	0	2.345611	-3.969226	3.996091
60	6	0	2.345611	3.969226	-3.996091
61	6	0	-2.345611	3.969226	-3.996091
62	6	0	-2.345611	-3.969226	-3.996091
63	6	0	2.345611	-3.969226	-3.996091
64	6	0	1.867069	4.667765	2.890371
65	6	0	-1.867069	-4.667765	2.890371
66	6	0	-1.867069	4.667765	2.890371
67	6	0	1.867069	-4.667765	2.890371
68	6	0	1.867069	4.667765	-2.890371
69	6	0	-1.867069	4.667765	-2.890371
70	6	0	-1.867069	-4.667765	-2.890371
71	6	0	1.867069	-4.667765	-2.890371
72	6	0	1.652572	4.015308	1.674875
73	6	0	-1.652572	-4.015308	1.674875
74	6	0	-1.652572	4.015308	1.674875
75	6	0	1.652572	-4.015308	1.674875
76	6	0	1.652572	4.015308	-1.674875
77	6	0	-1.652572	-4.015308	-1.674875
78	6	0	1.652572	-4.015308	-1.674875
79	6	0	-1.652572	4.015308	-1.674875
80	6	0	2.612683	2.604882	3.877875
81	6	0	-2.612683	-2.604882	3.877875
82	6	0	-2.612683	2.604882	3.877875
83	6	0	2.612683	-2.604882	3.877875
84	6	0	2.612683	2.604882	-3.877875
85	6	0	-2.612683	2.604882	-3.877875
86	6	0	-2.612683	-2.604882	-3.877875
87	6	0	2.612683	-2.604882	-3.877875
88	6	0	2.411386	1.958067	2.662361
89	6	0	-2.411386	-1.958067	2.662361
90	6	0	-2.411386	1.958067	2.662361
91	6	0	2.411386	-1.958067	2.662361
92	6	0	2.411386	1.958067	-2.662361
93	6	0	-2.411386	1.958067	-2.662361
94	6	0	-2.411386	-1.958067	-2.662361
95	6	0	2.411386	-1.958067	-2.662361
96	6	0	7.502671	4.032227	-3.931810
97	6	0	7.502671	-4.032227	-3.931810
98	6	0	7.502671	4.032227	3.931810
99	6	0	7.502671	-4.032227	3.931810
100	6	0	-7.502671	4.032227	-3.931810
101	6	0	-7.502671	4.032227	3.931810
102	6	0	-7.502671	-4.032227	-3.931810
103	6	0	-7.502671	-4.032227	3.931810
104	6	0	6.568872	3.709599	-1.721934
105	6	0	-6.568872	3.709599	-1.721934
106	6	0	6.568872	-3.709599	1.721934

107	6	0	-6.568872	3.709599	1.721934
108	6	0	-6.568872	-3.709599	-1.721934
109	6	0	6.568872	-3.709599	-1.721934
110	6	0	-6.568872	-3.709599	1.721934
111	6	0	6.568872	3.709599	1.721934
112	6	0	8.274196	2.210133	-2.544214
113	6	0	8.274196	-2.210133	-2.544214
114	6	0	-8.274196	2.210133	-2.544214
115	6	0	8.274196	2.210133	2.544214
116	6	0	-8.274196	-2.210133	2.544214
117	6	0	-8.274196	2.210133	2.544214
118	6	0	-8.274196	-2.210133	-2.544214
119	6	0	8.274196	-2.210133	2.544214
120	6	0	8.323738	2.922280	-3.742919
121	6	0	8.323738	-2.922280	-3.742919
122	6	0	8.323738	2.922280	3.742919
123	6	0	8.323738	-2.922280	3.742919
124	6	0	-8.323738	2.922280	-3.742919
125	6	0	-8.323738	2.922280	3.742919
126	6	0	-8.323738	-2.922280	-3.742919
127	6	0	-8.323738	-2.922280	3.742919
128	6	0	6.625332	4.423048	-2.918027
129	6	0	6.625332	4.423048	2.918027
130	6	0	6.625332	-4.423048	-2.918027
131	6	0	6.625332	-4.423048	2.918027
132	6	0	-6.625332	4.423048	-2.918027
133	6	0	-6.625332	4.423048	2.918027
134	6	0	-6.625332	-4.423048	-2.918027
135	6	0	-6.625332	-4.423048	2.918027
136	1	0	4.547770	2.482003	0.000000
137	1	0	-4.547770	-2.482003	-0.000000
138	1	0	-4.547770	2.482003	-0.000000
139	1	0	4.547770	-2.482003	-0.000000
140	1	0	1.271783	4.607913	0.849921
141	1	0	-1.271783	-4.607913	0.849921
142	1	0	-1.271783	4.607913	0.849921
143	1	0	1.271783	-4.607913	0.849921
144	1	0	1.271783	4.607913	-0.849921
145	1	0	-1.271783	4.607913	-0.849921
146	1	0	-1.271783	-4.607913	-0.849921
147	1	0	1.271783	-4.607913	-0.849921
148	1	0	2.996261	2.042304	4.731877
149	1	0	-2.996261	-2.042304	4.731877
150	1	0	-2.996261	2.042304	4.731877
151	1	0	2.996261	-2.042304	4.731877
152	1	0	2.996261	2.042304	-4.731877
153	1	0	-2.996261	2.042304	-4.731877
154	1	0	-2.996261	-2.042304	-4.731877
155	1	0	2.996261	-2.042304	-4.731877
156	1	0	-1.657608	-5.737334	-2.965931
157	1	0	-1.657608	5.737334	-2.965931
158	1	0	1.657608	5.737334	2.965931
159	1	0	-1.657608	-5.737334	2.965931
160	1	0	-1.657608	5.737334	2.965931
161	1	0	1.657608	5.737334	-2.965931
162	1	0	1.657608	-5.737334	2.965931
163	1	0	1.657608	-5.737334	-2.965931

164	1	0	2.515066	-4.484797	4.944184
165	1	0	-2.515066	4.484797	-4.944184
166	1	0	2.515066	4.484797	4.944184
167	1	0	-2.515066	-4.484797	4.944184
168	1	0	-2.515066	4.484797	4.944184
169	1	0	2.515066	4.484797	-4.944184
170	1	0	2.515066	-4.484797	-4.944184
171	1	0	-2.515066	-4.484797	-4.944184
172	1	0	2.659002	0.900788	2.587629
173	1	0	-2.659002	-0.900788	2.587629
174	1	0	-2.659002	0.900788	2.587629
175	1	0	2.659002	-0.900788	2.587629
176	1	0	2.659002	0.900788	-2.587629
177	1	0	-2.659002	0.900788	-2.587629
178	1	0	-2.659002	-0.900788	-2.587629
179	1	0	2.659002	-0.900788	-2.587629
180	1	0	8.952031	1.369221	-2.385147
181	1	0	8.952031	1.369221	2.385147
182	1	0	-8.952031	1.369221	-2.385147
183	1	0	-8.952031	1.369221	2.385147
184	1	0	-8.952031	-1.369221	-2.385147
185	1	0	-8.952031	-1.369221	2.385147
186	1	0	8.952031	-1.369221	2.385147
187	1	0	8.952031	-1.369221	-2.385147
188	1	0	5.880354	4.055660	-0.951544
189	1	0	5.880354	4.055660	0.951544
190	1	0	5.880354	-4.055660	-0.951544
191	1	0	5.880354	-4.055660	0.951544
192	1	0	-5.880354	4.055660	0.951544
193	1	0	-5.880354	4.055660	-0.951544
194	1	0	-5.880354	-4.055660	-0.951544
195	1	0	-5.880354	-4.055660	0.951544
196	1	0	5.988860	5.301049	3.051988
197	1	0	5.988860	5.301049	-3.051988
198	1	0	5.988860	-5.301049	-3.051988
199	1	0	5.988860	-5.301049	3.051988
200	1	0	-5.988860	5.301049	-3.051988
201	1	0	-5.988860	5.301049	3.051988
202	1	0	-5.988860	-5.301049	-3.051988
203	1	0	-5.988860	-5.301049	3.051988
204	1	0	7.554609	4.602128	4.862850
205	1	0	7.554609	4.602128	-4.862850
206	1	0	7.554609	-4.602128	-4.862850
207	1	0	7.554609	-4.602128	4.862850
208	1	0	-7.554609	4.602128	-4.862850
209	1	0	-7.554609	4.602128	4.862850
210	1	0	-7.554609	-4.602128	-4.862850
211	1	0	-7.554609	-4.602128	4.862850
212	1	0	9.025682	2.615970	-4.522036
213	1	0	9.025682	2.615970	4.522036
214	1	0	9.025682	-2.615970	4.522036
215	1	0	9.025682	-2.615970	-4.522036
216	1	0	-9.025682	2.615970	-4.522036
217	1	0	-9.025682	2.615970	4.522036
218	1	0	-9.025682	-2.615970	-4.522036
219	1	0	-9.025682	-2.615970	4.522036

Table S14. D_2 complex [**1**]$^{2+}$
B3PW91/Def2SVP-optimized
E = -12142.164330 au

Center Number	Atomic Number	Atomic Type	Coordinates (Angstroms) X	Y	Z
1	1	0	0.508218	-2.436581	4.578647
2	6	0	0.284172	-1.367642	4.573644
3	6	0	-0.284172	1.367642	4.573644
4	6	0	0.104921	-0.693657	3.360562
5	6	0	0.174184	-0.680414	5.787722
6	6	0	-0.174184	0.680414	5.787722
7	6	0	-0.104921	0.693657	3.360562
8	15	0	-0.089595	-1.595594	1.773749
9	15	0	0.445197	-1.473260	7.446551
10	15	0	-0.445197	1.473260	7.446551
11	15	0	0.089595	1.595594	1.773749
12	1	0	-0.508218	2.436581	4.578647
13	16	0	1.063747	1.143489	-10.445927
14	6	0	0.444917	0.523302	-11.955825
15	6	0	-1.125128	2.949143	1.872294
16	6	0	-0.928966	-1.141325	-13.144264
17	15	0	0.089595	-1.595594	-1.773749
18	6	0	-0.444917	-0.523302	-11.955825
19	6	0	1.125128	2.949143	-1.872294
20	6	0	0.928966	1.141325	-13.144264
21	16	0	1.063747	-1.143489	10.445927
22	6	0	-0.104921	-0.693657	-3.360562
23	6	0	0.174184	0.680414	-5.787722
24	6	0	-0.284172	-1.367642	-4.573644
25	6	0	0.104921	0.693657	-3.360562
26	6	0	0.284172	1.367642	-4.573644
27	6	0	-0.174184	-0.680414	-5.787722
28	1	0	-0.508218	-2.436581	-4.578647
29	15	0	-0.089595	1.595594	-1.773749
30	1	0	0.508218	2.436581	-4.578647
31	15	0	-0.445197	-1.473260	-7.446551
32	15	0	0.445197	1.473260	-7.446551
33	6	0	-1.763675	2.316239	-1.926830
34	78	0	0.000000	0.000000	0.000000
35	6	0	1.763675	2.316239	1.926830
36	16	0	-1.063747	-1.143489	-10.445927
37	28	0	0.000000	-0.000000	8.955145
38	6	0	1.125128	-2.949143	1.872294
39	16	0	-1.063747	1.143489	10.445927
40	6	0	-0.928966	1.141325	13.144264
41	6	0	0.444917	-0.523302	11.955825
42	6	0	-0.444917	0.523302	11.955825
43	6	0	0.928966	-1.141325	13.144264
44	28	0	-0.000000	-0.000000	-8.955145
45	6	0	-1.763675	-2.316239	1.926830
46	6	0	1.763675	-2.316239	-1.926830
47	6	0	-1.125128	-2.949143	-1.872294
48	6	0	0.713702	-2.890967	-7.463796
49	6	0	-2.124716	-2.176657	-7.333396

50	6	0	-0.713702	2.890967	-7.463796
51	6	0	2.124716	2.176657	-7.333396
52	6	0	0.713702	2.890967	7.463796
53	6	0	-2.124716	2.176657	7.333396
54	6	0	-0.713702	-2.890967	7.463796
55	6	0	2.124716	-2.176657	7.333396
56	7	0	1.347690	-1.676642	14.087507
57	7	0	-1.347690	1.676642	14.087507
58	7	0	-1.347690	-1.676642	-14.087507
59	7	0	1.347690	1.676642	-14.087507
60	6	0	-3.048214	4.974266	2.064053
61	6	0	-0.785131	4.274506	1.564748
62	6	0	-2.436904	2.644316	2.274032
63	6	0	-3.390559	3.655098	2.371738
64	6	0	-1.748028	5.280932	1.661353
65	1	0	0.234587	4.528358	1.269363
66	1	0	-2.713412	1.618147	2.532153
67	1	0	-4.403631	3.414666	2.702007
68	1	0	-1.475360	6.314231	1.433411
69	1	0	-3.795574	5.766795	2.148896
70	6	0	-3.048214	-4.974266	-2.064053
71	6	0	-2.436904	-2.644316	-2.274032
72	6	0	-0.785131	-4.274506	-1.564748
73	6	0	-1.748028	-5.280932	-1.661353
74	6	0	-3.390559	-3.655098	-2.371738
75	1	0	-2.713412	-1.618147	-2.532153
76	1	0	0.234587	-4.528358	-1.269363
77	1	0	-1.475360	-6.314231	-1.433411
78	1	0	-4.403631	-3.414666	-2.702007
79	1	0	-3.795574	-5.766795	-2.148896
80	6	0	-4.383293	3.246157	-2.282052
81	6	0	-2.052241	3.256598	-2.931099
82	6	0	-2.800104	1.847472	-1.108262
83	6	0	-4.104404	2.310842	-1.286018
84	6	0	-3.355313	3.718724	-3.102072
85	1	0	-1.269529	3.633139	-3.592424
86	1	0	-2.582616	1.118490	-0.324192
87	1	0	-4.905056	1.941233	-0.641287
88	1	0	-3.568475	4.446433	-3.888229
89	1	0	-5.404455	3.608608	-2.423106
90	6	0	-4.383293	-3.246157	2.282052
91	6	0	-2.800104	-1.847472	1.108262
92	6	0	-2.052241	-3.256598	2.931099
93	6	0	-3.355313	-3.718724	3.102072
94	6	0	-4.104404	-2.310842	1.286018
95	1	0	-2.582616	-1.118490	0.324192
96	1	0	-1.269529	-3.633139	3.592424
97	1	0	-3.568475	-4.446433	3.888229
98	1	0	-4.905056	-1.941233	0.641287
99	1	0	-5.404455	-3.608608	2.423106
100	6	0	4.383293	3.246157	2.282052
101	6	0	2.052241	3.256598	2.931099
102	6	0	2.800104	1.847472	1.108262
103	6	0	4.104404	2.310842	1.286018
104	6	0	3.355313	3.718724	3.102072
105	1	0	1.269529	3.633139	3.592424
106	1	0	2.582616	1.118490	0.324192

107	1	0	4.905056	1.941233	0.641287
108	1	0	3.568475	4.446433	3.888229
109	1	0	5.404455	3.608608	2.423106
110	6	0	3.048214	4.974266	-2.064053
111	6	0	0.785131	4.274506	-1.564748
112	6	0	2.436904	2.644316	-2.274032
113	6	0	3.390559	3.655098	-2.371738
114	6	0	1.748028	5.280932	-1.661353
115	1	0	-0.234587	4.528358	-1.269363
116	1	0	2.713412	1.618147	-2.532153
117	1	0	4.403631	3.414666	-2.702007
118	1	0	1.475360	6.314231	-1.433411
119	1	0	3.795574	5.766795	-2.148896
120	6	0	4.383293	-3.246157	-2.282052
121	6	0	2.800104	-1.847472	-1.108262
122	6	0	2.052241	-3.256598	-2.931099
123	6	0	3.355313	-3.718724	-3.102072
124	6	0	4.104404	-2.310842	-1.286018
125	1	0	2.582616	-1.118490	-0.324192
126	1	0	1.269529	-3.633139	-3.592424
127	1	0	3.568475	-4.446433	-3.888229
128	1	0	4.905056	-1.941233	-0.641287
129	1	0	5.404455	-3.608608	-2.423106
130	6	0	3.048214	-4.974266	2.064053
131	6	0	2.436904	-2.644316	2.274032
132	6	0	0.785131	-4.274506	1.564748
133	6	0	1.748028	-5.280932	1.661353
134	6	0	3.390559	-3.655098	2.371738
135	1	0	2.713412	-1.618147	2.532153
136	1	0	-0.234587	-4.528358	1.269363
137	1	0	1.475360	-6.314231	1.433411
138	1	0	4.403631	-3.414666	2.702007
139	1	0	3.795574	-5.766795	2.148896
140	6	0	2.571314	4.981590	7.581371
141	6	0	0.458220	4.099546	6.795677
142	6	0	1.898217	2.742826	8.199667
143	6	0	2.825899	3.783929	8.250916
144	6	0	1.386241	5.139641	6.856816
145	1	0	-0.484094	4.248979	6.261256
146	1	0	2.079130	1.817345	8.755017
147	1	0	3.739757	3.665180	8.837873
148	1	0	1.171967	6.088861	6.358653
149	1	0	3.287355	5.805057	7.643585
150	6	0	-4.774414	3.086763	7.434433
151	6	0	-3.126816	1.527070	6.594134
152	6	0	-2.467219	3.273723	8.140634
153	6	0	-3.786446	3.726053	8.183714
154	6	0	-4.442647	1.985652	6.641681
155	1	0	-2.886933	0.648033	5.989843
156	1	0	-1.709359	3.769778	8.750925
157	1	0	-4.042904	4.576113	8.820244
158	1	0	-5.218012	1.470007	6.069211
159	1	0	-5.808094	3.438257	7.479701
160	6	0	4.774414	-3.086763	7.434433
161	6	0	2.467219	-3.273723	8.140634
162	6	0	3.126816	-1.527070	6.594134
163	6	0	4.442647	-1.985652	6.641681

164	6	0	3.786446	-3.726053	8.183714
165	1	0	1.709359	-3.769778	8.750925
166	1	0	2.886933	-0.648033	5.989843
167	1	0	5.218012	-1.470007	6.069211
168	1	0	4.042904	-4.576113	8.820244
169	1	0	5.808094	-3.438257	7.479701
170	6	0	-2.571314	-4.981590	7.581371
171	6	0	-1.898217	-2.742826	8.199667
172	6	0	-0.458220	-4.099546	6.795677
173	6	0	-1.386241	-5.139641	6.856816
174	6	0	-2.825899	-3.783929	8.250916
175	1	0	-2.079130	-1.817345	8.755017
176	1	0	0.484094	-4.248979	6.261256
177	1	0	-1.171967	-6.088861	6.358653
178	1	0	-3.739757	-3.665180	8.837873
179	1	0	-3.287355	-5.805057	7.643585
180	6	0	4.774414	3.086763	-7.434433
181	6	0	3.126816	1.527070	-6.594134
182	6	0	2.467219	3.273723	-8.140634
183	6	0	3.786446	3.726053	-8.183714
184	6	0	4.442647	1.985652	-6.641681
185	1	0	2.886933	0.648033	-5.989843
186	1	0	1.709359	3.769778	-8.750925
187	1	0	4.042904	4.576113	-8.820244
188	1	0	5.218012	1.470007	-6.069211
189	1	0	5.808094	3.438257	-7.479701
190	6	0	-2.571314	4.981590	-7.581371
191	6	0	-0.458220	4.099546	-6.795677
192	6	0	-1.898217	2.742826	-8.199667
193	6	0	-2.825899	3.783929	-8.250916
194	6	0	-1.386241	5.139641	-6.856816
195	1	0	0.484094	4.248979	-6.261256
196	1	0	-2.079130	1.817345	-8.755017
197	1	0	-3.739757	3.665180	-8.837873
198	1	0	-1.171967	6.088861	-6.358653
199	1	0	-3.287355	5.805057	-7.643585
200	6	0	2.571314	-4.981590	-7.581371
201	6	0	1.898217	-2.742826	-8.199667
202	6	0	0.458220	-4.099546	-6.795677
203	6	0	1.386241	-5.139641	-6.856816
204	6	0	2.825899	-3.783929	-8.250916
205	1	0	2.079130	-1.817345	-8.755017
206	1	0	-0.484094	-4.248979	-6.261256
207	1	0	1.171967	-6.088861	-6.358653
208	1	0	3.739757	-3.665180	-8.837873
209	1	0	3.287355	-5.805057	-7.643585
210	6	0	-4.774414	-3.086763	-7.434433
211	6	0	-2.467219	-3.273723	-8.140634
212	6	0	-3.126816	-1.527070	-6.594134
213	6	0	-4.442647	-1.985652	-6.641681
214	6	0	-3.786446	-3.726053	-8.183714
215	1	0	-1.709359	-3.769778	-8.750925
216	1	0	-2.886933	-0.648033	-5.989843
217	1	0	-5.218012	-1.470007	-6.069211
218	1	0	-4.042904	-4.576113	-8.820244
219	1	0	-5.808094	-3.438257	-7.479701

Table S15. C_i complex [**1**]$^{2+}$
B3PW91/Def2SVP-optimized
E = -12142.172096 au

Center Number	Atomic Number	Atomic Type	Coordinates (Angstroms) X	Y	Z
1	78	0	0.000000	0.000000	0.000000
2	28	0	6.164772	6.203969	-0.901885
3	28	0	-6.164772	-6.203969	0.901885
4	16	0	6.023083	8.316555	-0.462340
5	16	0	-6.023083	-8.316555	0.462340
6	16	0	8.241510	6.138569	-0.302369
7	16	0	-8.241510	-6.138569	0.302369
8	15	0	4.099585	6.198271	-1.610673
9	15	0	-4.099585	-6.198271	1.610673
10	15	0	6.242030	4.092461	-1.451054
11	15	0	-6.242030	-4.092461	1.451054
12	15	0	-0.065586	-2.322447	0.546603
13	15	0	0.065586	2.322447	-0.546603
14	15	0	2.360576	0.077301	-0.347958
15	15	0	-2.360576	-0.077301	0.347958
16	7	0	7.986224	11.252954	0.608954
17	7	0	-7.986224	-11.252954	-0.608954
18	7	0	11.060185	8.234853	0.831120
19	7	0	-11.060185	-8.234853	-0.831120
20	6	0	-7.857985	-10.127663	-0.346493
21	6	0	-7.645897	-8.759037	-0.015586
22	6	0	-8.622204	-7.800464	-0.086056
23	6	0	-9.959361	-8.064459	-0.498315
24	6	0	-3.497821	-4.464960	1.343655
25	6	0	-2.166566	-4.117285	1.122354
26	1	0	-1.401964	-4.897456	1.149822
27	6	0	-1.819821	-2.788276	0.824353
28	6	0	-2.816104	-1.810852	0.744947
29	6	0	-4.158811	-2.161192	0.967249
30	1	0	-4.950042	-1.413354	0.874158
31	6	0	-4.502570	-3.477977	1.266819
32	6	0	-2.827691	-7.245249	0.820926
33	6	0	-2.901574	-7.454141	-0.565830
34	1	0	-3.718382	-7.012266	-1.142148
35	6	0	-1.963886	-8.267481	-1.200005
36	1	0	-2.040675	-8.444999	-2.275440
37	6	0	-0.955964	-8.886802	-0.457536
38	1	0	-0.237784	-9.544676	-0.953450
39	6	0	-0.884413	-8.690638	0.922592
40	1	0	-0.112760	-9.195473	1.509174
41	6	0	-1.818313	-7.874943	1.564038
42	1	0	-1.775929	-7.749817	2.648651
43	6	0	-3.933293	-6.489903	3.408202
44	6	0	-4.665848	-7.561005	3.945365
45	1	0	-5.315078	-8.158073	3.297386
46	6	0	-4.561305	-7.864917	5.302189
47	1	0	-5.129155	-8.703659	5.711792
48	6	0	-3.743921	-7.096568	6.133471
49	1	0	-3.670960	-7.333034	7.197896

50	6	0	-3.025780	-6.022073	5.605627
51	1	0	-2.391871	-5.414850	6.256702
52	6	0	-3.116353	-5.717744	4.246690
53	1	0	-2.549963	-4.872834	3.847547
54	6	0	-6.668158	-3.796773	3.204305
55	6	0	-7.757160	-4.517584	3.720858
56	1	0	-8.288975	-5.232987	3.085855
57	6	0	-8.162283	-4.316616	5.039889
58	1	0	-9.014427	-4.876087	5.433025
59	6	0	-7.477647	-3.413486	5.855374
60	1	0	-7.793359	-3.264676	6.890953
61	6	0	-6.385615	-2.706019	5.349392
62	1	0	-5.844072	-2.004510	5.988988
63	6	0	-5.980263	-2.893232	4.027274
64	1	0	-5.123038	-2.333819	3.644839
65	6	0	-7.245386	-2.906164	0.490172
66	6	0	-7.949444	-1.859663	1.103918
67	1	0	-7.911613	-1.734191	2.188663
68	6	0	-8.727828	-0.994864	0.332332
69	1	0	-9.290882	-0.193983	0.818074
70	6	0	-8.812760	-1.172521	-1.049515
71	1	0	-9.441425	-0.508172	-1.647785
72	6	0	-8.119634	-2.218209	-1.663452
73	1	0	-8.210345	-2.378435	-2.740507
74	6	0	-7.343150	-3.087418	-0.899038
75	1	0	-6.843375	-3.934378	-1.375985
76	6	0	0.482921	-3.476651	-0.758351
77	6	0	-0.398655	-3.768561	-1.812378
78	1	0	-1.405817	-3.345964	-1.822812
79	6	0	-0.007717	-4.623803	-2.840901
80	1	0	-0.707045	-4.856098	-3.647453
81	6	0	1.265592	-5.196709	-2.829978
82	1	0	1.566452	-5.877438	-3.629905
83	6	0	2.148722	-4.907218	-1.789305
84	1	0	3.142893	-5.359520	-1.771856
85	6	0	1.763682	-4.048152	-0.760080
86	1	0	2.459305	-3.846563	0.055229
87	6	0	0.761582	-2.771414	2.109858
88	6	0	1.071112	-4.105242	2.426918
89	1	0	0.917126	-4.905711	1.700099
90	6	0	1.581638	-4.421443	3.685674
91	1	0	1.821366	-5.460205	3.924963
92	6	0	1.778877	-3.419044	4.637771
93	1	0	2.174401	-3.673461	5.624150
94	6	0	1.471111	-2.093286	4.328417
95	1	0	1.626706	-1.306656	5.069941
96	6	0	0.967451	-1.769493	3.069613
97	1	0	0.737001	-0.729287	2.827508
98	6	0	-3.405219	0.317871	-1.097709
99	6	0	-3.607535	-0.669521	-2.076156
100	1	0	-3.185428	-1.668786	-1.948460
101	6	0	-4.376486	-0.394376	-3.205555
102	1	0	-4.539789	-1.174606	-3.952634
103	6	0	-4.952519	0.866667	-3.371187
104	1	0	-5.566320	1.076925	-4.250302
105	6	0	-4.752812	1.853822	-2.405487
106	1	0	-5.208801	2.839116	-2.525962

107	6	0	-3.979209	1.585110	-1.276499
108	1	0	-3.846898	2.361989	-0.522746
109	6	0	-2.965146	0.877275	1.780367
110	6	0	-4.325980	1.189458	1.941329
111	1	0	-5.051783	0.957653	1.158882
112	6	0	-4.765581	1.802900	3.114196
113	1	0	-5.824703	2.043816	3.232380
114	6	0	-3.860402	2.101415	4.134783
115	1	0	-4.211439	2.577607	5.053545
116	6	0	-2.508201	1.791740	3.980249
117	1	0	-1.797427	2.026177	4.775673
118	6	0	-2.061154	1.184816	2.807744
119	1	0	-1.000727	0.951438	2.686170
120	6	0	7.857985	10.127663	0.346493
121	6	0	7.645897	8.759037	0.015586
122	6	0	8.622204	7.800464	0.086056
123	6	0	9.959361	8.064459	0.498315
124	6	0	3.497821	4.464960	-1.343655
125	6	0	2.166566	4.117285	-1.122354
126	1	0	1.401964	4.897456	-1.149822
127	6	0	1.819821	2.788276	-0.824353
128	6	0	2.816104	1.810852	-0.744947
129	6	0	4.158811	2.161192	-0.967249
130	1	0	4.950042	1.413354	-0.874158
131	6	0	4.502570	3.477977	-1.266819
132	6	0	2.827691	7.245249	-0.820926
133	6	0	2.901574	7.454141	0.565830
134	1	0	3.718382	7.012266	1.142148
135	6	0	1.963886	8.267481	1.200005
136	1	0	2.040675	8.444999	2.275440
137	6	0	0.955964	8.886802	0.457536
138	1	0	0.237784	9.544676	0.953450
139	6	0	0.884413	8.690638	-0.922592
140	1	0	0.112760	9.195473	-1.509174
141	6	0	1.818313	7.874943	-1.564038
142	1	0	1.775929	7.749817	-2.648651
143	6	0	3.933293	6.489903	-3.408202
144	6	0	4.665848	7.561005	-3.945365
145	1	0	5.315078	8.158073	-3.297386
146	6	0	4.561305	7.864917	-5.302189
147	1	0	5.129155	8.703659	-5.711792
148	6	0	3.743921	7.096568	-6.133471
149	1	0	3.670960	7.333034	-7.197896
150	6	0	3.025780	6.022073	-5.605627
151	1	0	2.391871	5.414850	-6.256702
152	6	0	3.116353	5.717744	-4.246690
153	1	0	2.549963	4.872834	-3.847547
154	6	0	6.668158	3.796773	-3.204305
155	6	0	7.757160	4.517584	-3.720858
156	1	0	8.288975	5.232987	-3.085855
157	6	0	8.162283	4.316616	-5.039889
158	1	0	9.014427	4.876087	-5.433025
159	6	0	7.477647	3.413486	-5.855374
160	1	0	7.793359	3.264676	-6.890953
161	6	0	6.385615	2.706019	-5.349392
162	1	0	5.844072	2.004510	-5.988988
163	6	0	5.980263	2.893232	-4.027274

164	1	0	5.123038	2.333819	-3.644839
165	6	0	7.245386	2.906164	-0.490172
166	6	0	7.949444	1.859663	-1.103918
167	1	0	7.911613	1.734191	-2.188663
168	6	0	8.727828	0.994864	-0.332332
169	1	0	9.290882	0.193983	-0.818074
170	6	0	8.812760	1.172521	1.049515
171	1	0	9.441425	0.508172	1.647785
172	6	0	8.119634	2.218209	1.663452
173	1	0	8.210345	2.378435	2.740507
174	6	0	7.343150	3.087418	0.899038
175	1	0	6.843375	3.934378	1.375985
176	6	0	-0.482921	3.476651	0.758351
177	6	0	0.398655	3.768561	1.812378
178	1	0	1.405817	3.345964	1.822812
179	6	0	0.007717	4.623803	2.840901
180	1	0	0.707045	4.856098	3.647453
181	6	0	-1.265592	5.196709	2.829978
182	1	0	-1.566452	5.877438	3.629905
183	6	0	-2.148722	4.907218	1.789305
184	1	0	-3.142893	5.359520	1.771856
185	6	0	-1.763682	4.048152	0.760080
186	1	0	-2.459305	3.846563	-0.055229
187	6	0	-0.761582	2.771414	-2.109858
188	6	0	-1.071112	4.105242	-2.426918
189	1	0	-0.917126	4.905711	-1.700099
190	6	0	-1.581638	4.421443	-3.685674
191	1	0	-1.821366	5.460205	-3.924963
192	6	0	-1.778877	3.419044	-4.637771
193	1	0	-2.174401	3.673461	-5.624150
194	6	0	-1.471111	2.093286	-4.328417
195	1	0	-1.626706	1.306656	-5.069941
196	6	0	-0.967451	1.769493	-3.069613
197	1	0	-0.737001	0.729287	-2.827508
198	6	0	3.405219	-0.317871	1.097709
199	6	0	3.607535	0.669521	2.076156
200	1	0	3.185428	1.668786	1.948460
201	6	0	4.376486	0.394376	3.205555
202	1	0	4.539789	1.174606	3.952634
203	6	0	4.952519	-0.866667	3.371187
204	1	0	5.566320	-1.076925	4.250302
205	6	0	4.752812	-1.853822	2.405487
206	1	0	5.208801	-2.839116	2.525962
207	6	0	3.979209	-1.585110	1.276499
208	1	0	3.846898	-2.361989	0.522746
209	6	0	2.965146	-0.877275	-1.780367
210	6	0	4.325980	-1.189458	-1.941329
211	1	0	5.051783	-0.957653	-1.158882
212	6	0	4.765581	-1.802900	-3.114196
213	1	0	5.824703	-2.043816	-3.232380
214	6	0	3.860402	-2.101415	-4.134783
215	1	0	4.211439	-2.577607	-5.053545
216	6	0	2.508201	-1.791740	-3.980249
217	1	0	1.797427	-2.026177	-4.775673
218	6	0	2.061154	-1.184816	-2.807744
219	1	0	1.000727	-0.951438	-2.686170

Table S16. C_1 complex [**1**]$^{2+}$
B3PW91/Def2SVP-optimized
E = -12142.175578 au

Center Number	Atomic Number	Atomic Type	Coordinates (Angstroms) X	Y	Z
1	78	0	-0.006750	-0.055393	-0.454482
2	28	0	-8.800704	0.051828	0.127173
3	28	0	8.683893	0.050062	0.948242
4	16	0	9.953247	-1.512820	1.738201
5	16	0	-10.234668	-1.502748	-0.322454
6	16	0	9.888765	1.596750	1.861412
7	16	0	-10.209220	1.609362	-0.391044
8	15	0	-1.797911	1.487049	-0.771169
9	15	0	1.719921	1.552326	-0.164025
10	15	0	-1.791044	-1.657879	-0.479318
11	15	0	1.779095	-1.630399	-0.465767
12	15	0	-7.314038	1.555561	0.669650
13	15	0	7.407832	1.564705	0.030973
14	15	0	7.462249	-1.442073	-0.076015
15	15	0	-7.352306	-1.454067	0.754482
16	7	0	12.973010	2.185698	3.801265
17	7	0	-13.707451	2.216765	-1.399383
18	7	0	13.061931	-2.124374	3.631912
19	7	0	-13.743341	-2.095738	-1.303401
20	6	0	1.724363	2.225291	1.534265
21	6	0	-2.067965	1.738561	-2.564053
22	6	0	-1.838541	3.135785	0.004252
23	6	0	1.860600	2.930752	-1.348619
24	6	0	-1.764569	-2.878678	0.876807
25	6	0	1.909186	-2.479104	-2.078026
26	6	0	1.898896	-2.868754	0.866854
27	6	0	-2.098639	-2.547201	-2.041644
28	6	0	-3.340224	-0.728811	-0.132132
29	6	0	3.360838	-0.702709	-0.341009
30	6	0	-3.323355	0.671629	-0.174366
31	6	0	3.336213	0.693897	-0.286375
32	6	0	-5.692171	-0.682055	0.463346
33	6	0	5.786842	-0.666764	-0.248655
34	6	0	-4.485103	1.397872	0.129853
35	6	0	4.540825	1.415163	-0.223503
36	6	0	-4.526704	-1.402121	0.191719
37	6	0	4.591413	-1.380614	-0.320705
38	6	0	-5.671091	0.725974	0.436191
39	6	0	5.761788	0.742962	-0.203488
40	6	0	7.134785	-3.069434	0.685981
41	6	0	-7.249289	-3.069883	-0.090019
42	6	0	-7.402974	-1.826089	2.543919
43	6	0	8.001910	-1.795085	-1.787532
44	6	0	7.018670	3.123237	0.902344
45	6	0	-7.171493	3.101971	-0.293932
46	6	0	-7.356928	2.063059	2.425507
47	6	0	7.944296	2.057597	-1.646505
48	6	0	-2.021812	5.625000	1.267313
49	6	0	2.064163	4.978526	-3.250370

50	6	0	-1.785405	4.473825	2.024493
51	6	0	2.611717	3.721113	-3.517313
52	6	0	-7.581238	2.896183	5.087771
53	6	0	8.912279	2.870256	-4.143841
54	6	0	-6.380554	-2.237011	4.701794
55	6	0	7.609539	-2.185084	-4.146851
56	6	0	6.760497	-4.360805	2.702673
57	6	0	-7.392555	-4.322288	-2.160497
58	6	0	-1.361241	-3.308638	3.228980
59	6	0	1.489815	-2.427815	-4.467581
60	6	0	-1.964646	-4.562460	3.103834
61	6	0	2.271800	-3.572013	-4.631581
62	6	0	2.099856	-4.702616	2.974810
63	6	0	-2.556412	-3.872570	-4.467526
64	6	0	-2.169842	5.531680	-0.117164
65	6	0	1.410178	5.209062	-2.040002
66	6	0	1.887794	3.051055	4.206480
67	6	0	-2.436217	2.175880	-5.308426
68	6	0	-7.641396	-2.454396	5.260506
69	6	0	8.975266	-2.400415	-4.340771
70	6	0	1.193189	1.884825	3.878836
71	6	0	-1.150780	1.995555	-4.795752
72	6	0	6.702230	-5.531734	1.943512
73	6	0	-7.160499	-5.508331	-1.459944
74	6	0	-6.327078	2.595347	4.553359
75	6	0	7.559877	2.562830	-3.985189
76	6	0	-8.724134	2.776184	4.294933
77	6	0	9.782798	2.766944	-3.057177
78	6	0	2.559883	-3.394889	3.140942
79	6	0	-3.233190	-2.686493	-4.180373
80	6	0	-8.618308	2.353044	2.970491
81	6	0	9.306984	2.353684	-1.813385
82	6	0	-6.211415	2.181332	3.225801
83	6	0	7.073689	2.159533	-2.740863
84	6	0	-1.696078	3.232254	1.398178
85	6	0	2.512727	2.700465	-2.573667
86	6	0	2.505923	3.799011	3.202758
87	6	0	-3.537845	2.140363	-4.451089
88	6	0	6.500068	5.478622	2.322480
89	6	0	-7.035752	5.429932	-1.842327
90	6	0	6.879830	3.089624	2.299360
91	6	0	-7.404608	3.045095	-1.678340
92	6	0	1.111623	1.473528	2.549116
93	6	0	-0.965059	1.776447	-3.430639
94	6	0	6.616986	4.264120	3.002987
95	6	0	-7.328715	4.205289	-2.447566
96	6	0	6.863850	-5.474773	0.558300
97	6	0	-6.978863	-5.477979	-0.076409
98	6	0	6.981856	-3.133294	2.080594
99	6	0	-7.445555	-3.107088	-1.480338
100	6	0	2.461396	-2.479685	2.094376
101	6	0	-3.009639	-2.024710	-2.973164
102	6	0	1.529434	-5.091315	1.761914
103	6	0	-1.643300	-4.392915	-3.548146
104	6	0	-1.258725	-2.470924	2.120809
105	6	0	1.305488	-1.884395	-3.196688
106	6	0	-2.465979	-4.974968	1.868743

107	6	0	2.871529	-4.172429	-3.521889
108	6	0	-6.257684	-1.925345	3.347191
109	6	0	7.120592	-1.884731	-2.874833
110	6	0	-8.783856	-2.353222	4.464428
111	6	0	9.855906	-2.308414	-3.261323
112	6	0	2.432667	3.389395	1.871221
113	6	0	-3.359746	1.922324	-3.084821
114	6	0	-2.076354	4.292002	-0.752909
115	6	0	1.300024	4.190709	-1.091354
116	6	0	9.376708	-1.998584	-1.988954
117	6	0	-8.670655	-2.031741	3.112275
118	6	0	6.905103	4.345538	0.223247
119	6	0	-6.885230	4.334330	0.310321
120	6	0	6.642042	5.517910	0.934044
121	6	0	-6.816337	5.493096	-0.465594
122	6	0	7.083304	-4.248613	-0.071964
123	6	0	-7.024038	-4.263488	0.610758
124	6	0	1.421719	-4.178942	0.712787
125	6	0	-1.406761	-3.731004	-2.344768
126	6	0	-2.372993	-4.137760	0.755536
127	6	0	2.698708	-3.629327	-2.248844
128	6	0	11.209813	0.722643	2.602119
129	6	0	-11.681781	0.741718	-0.760385
130	6	0	12.254471	-1.439098	3.152347
131	6	0	-12.835660	-1.415171	-1.049387
132	6	0	11.237880	-0.645966	2.548078
133	6	0	-11.692921	-0.627896	-0.730047
134	6	0	12.193857	1.507133	3.268276
135	6	0	-12.811372	1.532912	-1.114811
136	1	0	-1.675125	4.543694	3.108997
137	1	0	3.128672	3.537869	-4.462260
138	1	0	-2.097339	6.596964	1.761026
139	1	0	2.152821	5.781265	-3.986327
140	1	0	-7.669151	3.220611	6.127593
141	1	0	9.290560	3.186541	-5.119053
142	1	0	0.715153	1.293147	4.663301
143	1	0	-0.285212	2.029303	-5.461425
144	1	0	1.953300	3.374720	5.248133
145	1	0	-2.581089	2.347250	-6.377865
146	1	0	6.666176	-4.406958	3.790313
147	1	0	-7.564706	-4.347351	-3.239189
148	1	0	6.554526	-6.495846	2.436635
149	1	0	-7.144829	-6.462717	-1.992287
150	1	0	3.013408	-3.088147	4.086309
151	1	0	-3.950439	-2.276734	-4.895175
152	1	0	-2.046187	-5.219295	3.973312
153	1	0	2.413734	-4.000397	-5.626846
154	1	0	2.192212	-5.422799	3.791322
155	1	0	-2.744049	-4.395856	-5.408237
156	1	0	-0.967090	-2.984058	4.194524
157	1	0	1.012535	-1.960148	-5.331615
158	1	0	-5.486086	-2.309934	5.325575
159	1	0	6.920015	-2.250258	-4.992341
160	1	0	10.843004	3.001851	-3.177490
161	1	0	-9.708270	3.005973	4.710105
162	1	0	-7.734789	-2.698921	6.321513
163	1	0	9.355690	-2.636100	-5.337749

164	1	0	6.537227	4.234746	4.092420
165	1	0	-7.535714	4.159462	-3.519615
166	1	0	6.323749	6.402654	2.879288
167	1	0	-7.003520	6.342080	-2.443553
168	1	0	7.076709	-2.226377	2.683130
169	1	0	-7.677592	-2.188117	-2.024753
170	1	0	-2.365877	6.428317	-0.709884
171	1	0	0.985088	6.192229	-1.825525
172	1	0	-5.432875	2.682391	5.175770
173	1	0	6.879914	2.636170	-4.837724
174	1	0	-1.522558	2.337334	2.000276
175	1	0	2.956277	1.724799	-2.789724
176	1	0	-9.516395	2.249706	2.353479
177	1	0	9.993257	2.262676	-0.965639
178	1	0	-5.264684	-1.758081	2.922907
179	1	0	6.049414	-1.717999	-2.737466
180	1	0	-0.786831	-1.490344	2.222106
181	1	0	0.679380	-0.996401	-3.070897
182	1	0	-2.943480	-5.952523	1.768509
183	1	0	3.483535	-5.068864	-3.647164
184	1	0	2.845388	-1.465941	2.233338
185	1	0	-3.558083	-1.105451	-2.758382
186	1	0	1.171400	-6.114292	1.626499
187	1	0	-1.112685	-5.322199	-3.767180
188	1	0	6.844848	-6.392689	-0.034485
189	1	0	-6.824521	-6.407642	0.477018
190	1	0	7.017784	2.150299	2.840554
191	1	0	-7.690523	2.098874	-2.145264
192	1	0	0.568982	0.559480	2.290651
193	1	0	0.045853	1.651401	-3.038370
194	1	0	3.059868	4.705692	3.455200
195	1	0	-4.545951	2.286671	-4.845837
196	1	0	-9.772994	-2.518215	4.898112
197	1	0	10.926049	-2.471836	-3.408950
198	1	0	6.574671	6.469985	0.401826
199	1	0	-6.612856	6.453632	0.014097
200	1	0	-5.224151	1.948352	2.819510
201	1	0	6.013101	1.921919	-2.628533
202	1	0	-4.554984	-2.494612	0.217954
203	1	0	4.623098	-2.473045	-0.327076
204	1	0	-4.475313	2.490985	0.097528
205	1	0	4.530554	2.506453	-0.161741
206	1	0	10.069561	-1.918412	-1.145543
207	1	0	-9.567724	-1.943807	2.491524
208	1	0	2.935331	3.984162	1.105877
209	1	0	-4.235754	1.901521	-2.433208
210	1	0	7.047325	4.388678	-0.859207
211	1	0	-6.738766	4.395860	1.391286
212	1	0	7.240803	-4.214293	-1.152637
213	1	0	-6.906529	-4.249244	1.696833
214	1	0	0.982069	-4.503568	-0.231631
215	1	0	-0.690213	-4.151310	-1.637534
216	1	0	-2.788240	-4.468497	-0.198707
217	1	0	3.182639	-4.109598	-1.395613
218	1	0	-2.194138	4.229807	-1.836482
219	1	0	0.786395	4.388943	-0.149266

Table S17. C_2 complex [**1**]$^{2+}$
B3PW91/Def2SVP-optimized
E = -12142.174724 au

Center Number	Atomic Number	Atomic Type	Coordinates (Angstroms) X	Y	Z
1	78	0	0.000000	0.000000	0.127374
2	28	0	-4.419742	-7.570103	-0.732348
3	28	0	4.419742	7.570103	-0.732348
4	16	0	3.761133	9.422071	-1.634691
5	16	0	-3.761133	-9.422071	-1.634691
6	16	0	6.461494	7.896787	-1.368034
7	16	0	-6.461494	-7.896787	-1.368034
8	15	0	2.266533	0.739030	0.127144
9	15	0	-2.266533	-0.739030	0.127144
10	15	0	-0.515489	2.328636	0.088299
11	15	0	0.515489	-2.328636	0.088299
12	15	0	2.405820	7.215607	0.031611
13	15	0	-2.405820	-7.215607	0.031611
14	15	0	5.009651	5.725750	0.275855
15	15	0	-5.009651	-5.725750	0.275855
16	7	0	4.949711	12.444731	-3.388264
17	7	0	-4.949711	-12.444731	-3.388264
18	7	0	8.691236	10.328746	-3.022117
19	7	0	-8.691236	-10.328746	-3.022117
20	6	0	3.135657	0.405027	-1.441086
21	6	0	-3.135657	-0.405027	-1.441086
22	6	0	-1.414424	2.948677	1.549594
23	6	0	1.414424	-2.948677	1.549594
24	6	0	3.314062	0.219638	1.527500
25	6	0	-3.314062	-0.219638	1.527500
26	6	0	-1.335041	2.934553	-1.423464
27	6	0	1.335041	-2.934553	-1.423464
28	6	0	1.062557	3.264924	0.147527
29	6	0	-1.062557	-3.264924	0.147527
30	6	0	2.271900	2.571260	0.241945
31	6	0	-2.271900	-2.571260	0.241945
32	6	0	2.261351	5.374871	0.198123
33	6	0	-2.261351	-5.374871	0.198123
34	6	0	3.481314	4.675314	0.310127
35	6	0	-3.481314	-4.675314	0.310127
36	6	0	1.061253	4.669796	0.125311
37	6	0	-1.061253	-4.669796	0.125311
38	6	0	3.481327	3.281596	0.333261
39	6	0	-3.481327	-3.281596	0.333261
40	6	0	0.934710	7.698684	-0.937091
41	6	0	-0.934710	-7.698684	-0.937091
42	6	0	5.470112	5.958072	2.030700
43	6	0	-5.470112	-5.958072	2.030700
44	6	0	6.298307	4.640394	-0.432160
45	6	0	-6.298307	-4.640394	-0.432160
46	6	0	2.129373	7.872380	1.716074
47	6	0	-2.129373	-7.872380	1.716074
48	6	0	-2.526924	3.800437	-3.806151
49	6	0	2.526924	-3.800437	-3.806151

50	6	0	4.848595	-0.529657	3.749408
51	6	0	-4.848595	0.529657	3.749408
52	6	0	-1.143259	3.624653	-3.740942
53	6	0	1.143259	-3.624653	-3.740942
54	6	0	3.912076	0.500517	3.862399
55	6	0	-3.912076	-0.500517	3.862399
56	6	0	-1.250010	8.475759	-2.503645
57	6	0	1.250010	-8.475759	-2.503645
58	6	0	6.307680	6.411064	4.662617
59	6	0	-6.307680	-6.411064	4.662617
60	6	0	6.391757	4.540939	-1.829807
61	6	0	-6.391757	-4.540939	-1.829807
62	6	0	1.498899	7.146897	2.737308
63	6	0	-1.498899	-7.146897	2.737308
64	6	0	2.361261	0.168380	-2.587356
65	6	0	-2.361261	-0.168380	-2.587356
66	6	0	-1.265873	2.250825	2.757910
67	6	0	1.265873	-2.250825	2.757910
68	6	0	7.387495	3.755405	-2.408699
69	6	0	-7.387495	-3.755405	-2.408699
70	6	0	5.438575	5.356328	4.377863
71	6	0	-5.438575	-5.356328	4.377863
72	6	0	6.333243	7.026556	2.322934
73	6	0	-6.333243	-7.026556	2.322934
74	6	0	-0.214014	8.228335	-0.331083
75	6	0	0.214014	-8.228335	-0.331083
76	6	0	5.020590	5.126551	3.066489
77	6	0	-5.020590	-5.126551	3.066489
78	6	0	1.303169	7.730618	3.989673
79	6	0	-1.303169	-7.730618	3.989673
80	6	0	-0.104937	7.955860	-3.111451
81	6	0	0.104937	-7.955860	-3.111451
82	6	0	6.754772	7.242953	3.634409
83	6	0	-6.754772	-7.242953	3.634409
84	6	0	-2.970233	-0.042946	-3.834853
85	6	0	2.970233	0.042946	-3.834853
86	6	0	1.809590	-2.761502	3.935404
87	6	0	-1.809590	2.761502	3.935404
88	6	0	-4.357635	-0.146971	-3.947228
89	6	0	4.357635	0.146971	-3.947228
90	6	0	2.509729	-3.969378	3.915356
91	6	0	-2.509729	3.969378	3.915356
92	6	0	1.730196	9.038052	4.229031
93	6	0	-1.730196	-9.038052	4.229031
94	6	0	8.300539	3.072541	-1.601335
95	6	0	-8.300539	-3.072541	-1.601335
96	6	0	-1.303169	8.612220	-1.115541
97	6	0	1.303169	-8.612220	-1.115541
98	6	0	0.987762	7.574016	-2.334975
99	6	0	-0.987762	-7.574016	-2.334975
100	6	0	-0.546232	3.194444	-2.556938
101	6	0	0.546232	-3.194444	-2.556938
102	6	0	3.146718	0.875062	2.759551
103	6	0	-3.146718	-0.875062	2.759551
104	6	0	-2.663457	4.667145	2.715827
105	6	0	2.663457	-4.667145	2.715827
106	6	0	-3.315893	3.536715	-2.685537

107	6	0	3.315893	-3.536715	-2.685537
108	6	0	5.011056	-1.191254	2.531824
109	6	0	-5.011056	1.191254	2.531824
110	6	0	5.134482	0.381015	-2.810706
111	6	0	-5.134482	-0.381015	-2.810706
112	6	0	8.214357	3.171528	-0.211228
113	6	0	-8.214357	-3.171528	-0.211228
114	6	0	4.530828	0.516700	-1.560922
115	6	0	-4.530828	-0.516700	-1.560922
116	6	0	-2.115290	4.165796	1.534846
117	6	0	2.115290	-4.165796	1.534846
118	6	0	-2.726371	3.098409	-1.500289
119	6	0	2.726371	-3.098409	-1.500289
120	6	0	4.244794	-0.823899	1.425099
121	6	0	-4.244794	0.823899	1.425099
122	6	0	2.361261	9.762004	3.215663
123	6	0	-2.361261	-9.762004	3.215663
124	6	0	2.570256	9.182370	1.964741
125	6	0	-2.570256	-9.182370	1.964741
126	6	0	7.219747	3.956465	0.374973
127	6	0	-7.219747	-3.956465	0.374973
128	6	0	5.232721	10.141874	-2.246276
129	6	0	-5.232721	-10.141874	-2.246276
130	6	0	6.421059	9.470282	-2.129521
131	6	0	-6.421059	-9.470282	-2.129521
132	6	0	5.101833	11.410611	-2.879358
133	6	0	-5.101833	-11.410611	-2.879358
134	6	0	7.659686	9.964403	-2.628792
135	6	0	-7.659686	-9.964403	-2.628792
136	1	0	0.521118	-3.837824	-4.613343
137	1	0	3.784502	1.023243	4.813380
138	1	0	2.990724	-4.150800	-4.731352
139	1	0	5.457247	-0.813080	4.611413
140	1	0	2.095328	-8.798216	-3.116745
141	1	0	6.634282	6.588495	5.690243
142	1	0	2.359988	-0.140523	-4.721863
143	1	0	1.689069	-2.213279	4.872387
144	1	0	4.836254	0.048613	-4.924624
145	1	0	2.937211	-4.369485	4.837992
146	1	0	-0.049335	7.877202	-4.199857
147	1	0	-7.431638	-8.072313	3.852817
148	1	0	-2.095328	8.798216	-3.116745
149	1	0	-6.634282	-6.588495	5.690243
150	1	0	-0.521118	3.837824	-4.613343
151	1	0	-3.784502	-1.023243	4.813380
152	1	0	-4.836254	-0.048613	-4.924624
153	1	0	-2.937211	4.369485	4.837992
154	1	0	-2.990724	4.150800	-4.731352
155	1	0	-5.457247	0.813080	4.611413
156	1	0	-2.359988	0.140523	-4.721863
157	1	0	-1.689069	2.213279	4.872387
158	1	0	-7.470954	-3.704686	-3.497027
159	1	0	0.815291	7.160394	4.784228
160	1	0	7.431638	8.072313	3.852817
161	1	0	0.049335	-7.877202	-4.199857
162	1	0	-9.099182	-2.481976	-2.057815
163	1	0	1.575302	9.493143	5.210416

164	1	0	7.470954	3.704686	-3.497027
165	1	0	-0.815291	-7.160394	4.784228
166	1	0	9.099182	2.481976	-2.057815
167	1	0	-1.575302	-9.493143	5.210416
168	1	0	1.900108	7.214498	-2.817661
169	1	0	-6.677492	-7.685477	1.519735
170	1	0	4.398114	-3.677479	-2.731311
171	1	0	5.745433	-1.994415	2.437800
172	1	0	2.187754	-9.043400	-0.640241
173	1	0	5.083363	4.708976	5.183631
174	1	0	-0.540007	-3.080865	-2.516300
175	1	0	2.427840	1.692964	2.857907
176	1	0	-1.900108	-7.214498	-2.817661
177	1	0	6.677492	7.685477	1.519735
178	1	0	-5.710391	-5.111380	-2.465797
179	1	0	1.160243	6.122502	2.564164
180	1	0	-1.275467	-0.078574	-2.498841
181	1	0	-0.722223	1.302092	2.774270
182	1	0	-6.219411	-0.472219	-2.894963
183	1	0	-3.208862	5.613699	2.697816
184	1	0	0.540007	3.080865	-2.516300
185	1	0	-2.427840	-1.692964	2.857907
186	1	0	-4.398114	3.677479	-2.731311
187	1	0	-5.745433	1.994415	2.437800
188	1	0	-2.187754	9.043400	-0.640241
189	1	0	-5.083363	-4.708976	5.183631
190	1	0	5.710391	5.111380	-2.465797
191	1	0	-1.160243	-6.122502	2.564164
192	1	0	1.275467	0.078574	-2.498841
193	1	0	0.722223	-1.302092	2.774270
194	1	0	6.219411	0.472219	-2.894963
195	1	0	3.208862	-5.613699	2.697816
196	1	0	-8.941907	-2.657839	0.422062
197	1	0	2.701262	10.783907	3.399490
198	1	0	8.941907	2.657839	0.422062
199	1	0	-2.701262	-10.783907	3.399490
200	1	0	0.249874	-8.365737	0.752253
201	1	0	4.340933	4.297297	2.855852
202	1	0	-4.433668	-2.749320	0.396337
203	1	0	0.123967	5.222067	0.020435
204	1	0	-0.123967	-5.222067	0.020435
205	1	0	4.433668	2.749320	0.396337
206	1	0	3.073408	9.748122	1.174428
207	1	0	-7.179394	-4.056556	1.462249
208	1	0	5.157925	0.715988	-0.689602
209	1	0	2.235253	-4.732499	0.609016
210	1	0	7.179394	4.056556	1.462249
211	1	0	-3.073408	-9.748122	1.174428
212	1	0	-0.249874	8.365737	0.752253
213	1	0	-4.340933	-4.297297	2.855852
214	1	0	-3.355336	2.903270	-0.630672
215	1	0	-4.394281	1.340971	0.476130
216	1	0	-5.157925	-0.715988	-0.689602
217	1	0	-2.235253	4.732499	0.609016
218	1	0	3.355336	-2.903270	-0.630672
219	1	0	4.394281	-1.340971	0.476130
